# Derivatization of 2,1,3-Benzothiadiazole via Regioselective C–H Functionalization and Aryne Reactivity

**DOI:** 10.1021/acs.joc.4c00122

**Published:** 2024-04-22

**Authors:** Susanna Kunz, Fredrik Barnå, Mauricio Posada Urrutia, Fredric J. L. Ingner, Andrea Martínez-Topete, Andreas Orthaber, Paul J. Gates, Lukasz T. Pilarski, Christine Dyrager

**Affiliations:** † Department of Chemistry—BMC, Uppsala University, Box 576, Uppsala 75123, Sweden; ‡ Department of Chemistry—Ångström, Uppsala University, Box 523, Uppsala 75120, Sweden; § School of Chemistry, University of Bristol, Cantock’s Close, Clifton, Bristol BS8 1TS, U.K.

## Abstract

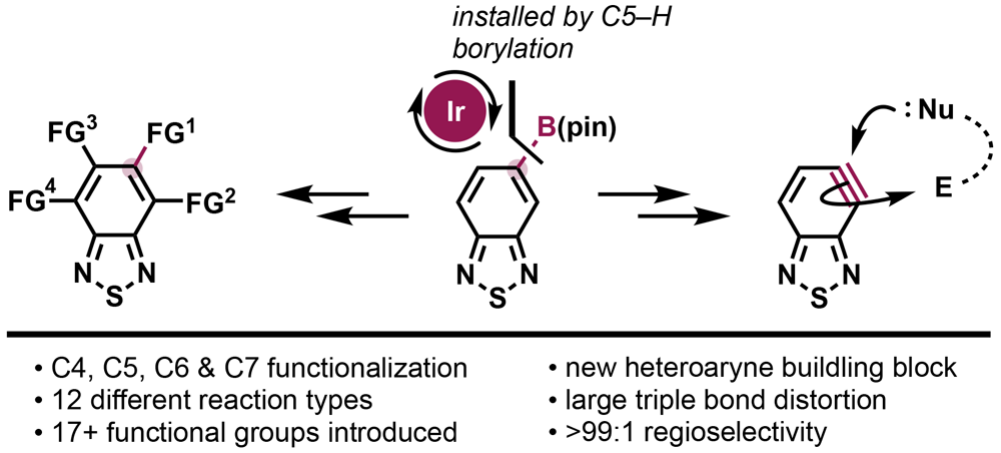

Despite growing interest in 2,1,3-benzothiadiazole (BTD) as an integral component of many functional molecules, methods for the functionalization of its benzenoid ring have remained limited, and many even simply decorated BTDs have required *de novo* synthesis. We show that regioselective Ir-catalyzed C–H borylation allows access to versatile 5-boryl or 4,6-diboryl BTD building blocks, which undergo functionalization at the C4, C5, C6, and C7 positions. The optimization and regioselectivity of C–H borylation are discussed. A broad reaction scope is presented, encompassing *ipso* substitution at the C–B bond, the first examples of *ortho*-directed C–H functionalization of BTD, ring closing reactions to generate fused ring systems, as well as the generation and capture reactions of novel BTD-based heteroarynes. The regioselectivity of the latter is discussed with reference to the Aryne Distortion Model.

## Introduction

**Figure 1:**
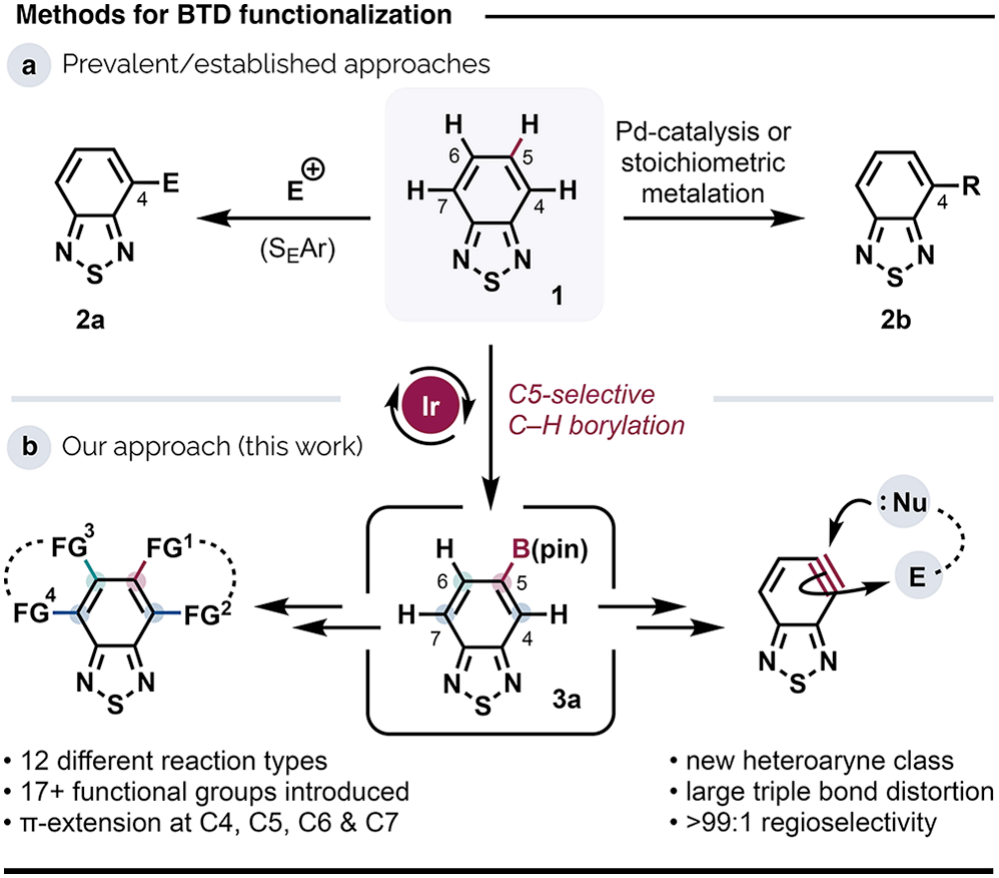
Functionalizing BTD: established approaches versus the C–H functionalization approach explored in this study.

2,1,3-Benzothiadiazole (BTD, **1**, Figure 1) is a privileged electron acceptor unit^1^ found at the core of numerous fluorescent probes^2^ and phototheranostics^3^ as well as covalent organic frameworks,^4^ and optoelectronic devices^5^ (including in polymers,^6^ OLEDs,^7^ solar cells,^8^ and transistors^9^). BTD has also recently found new roles in catalytic hydrogen production.^10^ The growing interest in BTD derivatives makes user-friendly approaches to functionalization a highly attractive prospect. Despite this, options for derivatizing BTD directly have remained remarkably few. Foremost among these are: (i) electrophilic aromatic substitution (to **2a**), which typically requires harsh conditions due to BTD’s electron-poor nature, and which can deliver mixtures of C4- and C7-substituted products,^11^ and (ii) tactics based on stoichiometric metalation (to **2b**).^12^ The C5–H or C6–H positions have remained essentially unaddressed. In practice, C5- and/or C6-substituted BTDs almost invariably require *de novo* assembly of the thiadiazoloid ring, usually from toxic SOCl_2_ and substituted aryl-1,2-diamines,^13^ which can themselves be tedious to prepare. This has restricted access to many potentially valuable BTD-based scaffolds and the exploration of their properties and of the contexts in which these can be exploited.

Recent years have seen C–H functionalization^14^ emerge as a transformative technology for expediting synthesis through previously impossible reactions and increasingly sustainable protocols.^15^ However, reports on the catalytic C–H functionalization of BTD have been sparse and overwhelmingly focused on Pd-catalyzed C4/C7 arylation,^16^ where CMD-type processes most easily take effect.^17^

Our teams’ interest in C–H functionalization^18^ and the use of BTD derivatives for bioimaging^11b,19^ led us to envisage Ir-catalyzed C–H borylation^20^ as a route to C5-boryl building block **3a**, in which the versatility of the C–B bond could be leveraged for decorating C5 directly and, importantly, for manipulating the neighboring C4 and C6 positions. Here, we describe the application of this strategy as an entry point to numerous novel BTD-based structures via C4–, C5–, and C6–H activation and even via unprecedented BTD-based heteroarynes.^21^

## Results and Discussion

### Boryl BTD via C–H Borylation

**Scheme 1:**
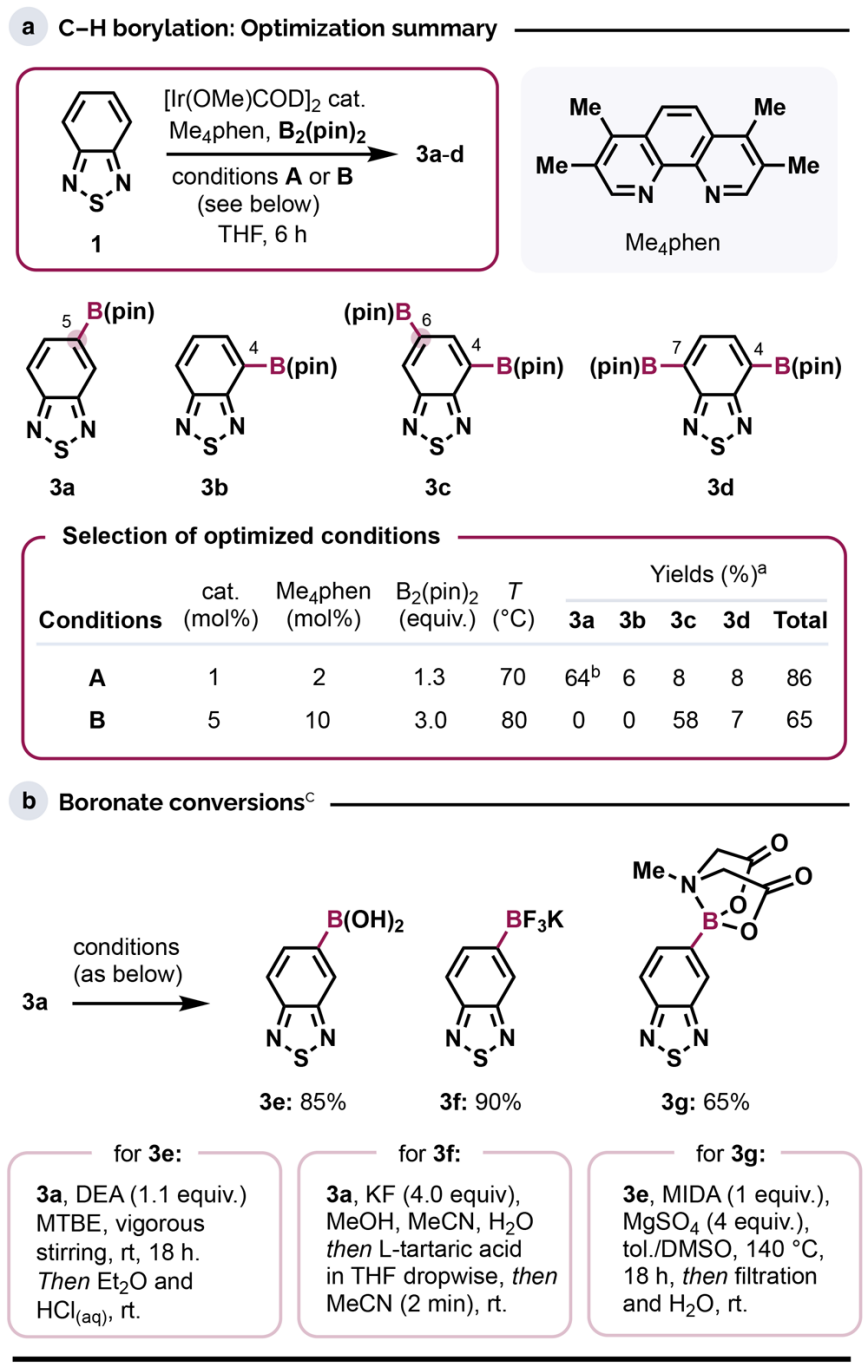
Generation of BTD-Based Boronates Yield determined by ^1^H NMR spectroscopy using 1,3,5-trimethoxybenzene as an internal standard. Scale: 0.5 mmol BTD. 1 g scale reaction: **3a** formed in 56% spectroscopic yield (see the Supporting Information for more details). For boronate interconversions: DEA = diethanolamine; MTBE = methyl-*tert-*butylether; and MIDA = methyliminodiacetic acid.

At the outset, we explored the Ir-catalyzed C–H borylation of **1** using B_2_(pin)_2_ and [Ir(OMe)COD]_2_ as the precatalyst. Two sets of optimized conditions are shown in Scheme 1a (see Table S1 for more details). Conditions A gave borylated derivatives **3** in 86% yield, with a strong preference for the desired building block **3a** (64%), with 4-boryl (**3b**, 6%), 4,6-diboryl (**3c**, 8%), and 4,7-diboryl (**3d**, 8%) congeners forming in minor amounts. For heteroarenes lacking sterically hindering substituents, Ir-catalyzed C–H borylation is often selective for the most acidic position,^20b^ but suppressing multiple borylations can be challenging. In BTD, C4–H is the most acidic proton;^12^ the high C5–H regioselectivity observed here is probably accounted for by the inhibitory effect of the N3 lone pair, by analogy with pyridines and quinolines.^22^ In situ instability of C4-boryl species **3b**–**d** is unlikely to explain the high levels of C5H borylation observed. Subjecting **3b** to conditions A resulted in a mixture of the diboryl products (**3c**/**3d** = 1:1), with no loss of B(pin) from the C4 position—*i.e*., **3b** is stable under the borylation conditions. Additionally, compounds **3a**–**d** proved indefinitely bench stable under air.

More forcing conditions (conditions B), involving higher catalyst, ligand and B_2_(pin)_2_ loadings, as well as a higher temperature, gave exclusively the diborylated products with a strong preference for 4,6-diboryl BTD derivative **3c**, which can arise from a second borylation of either **3a** at C7–H or **3b** at C6–H. We anticipate that this very direct route to **3c** will help challenge the conspicuous paucity of unsymmetrically substituted BTDs in the research literature, the properties of which are poorly explored.

More generally, we tentatively ascribe the need for elevated temperatures (only traces of borylated products formed at temperatures <40 °C) to the coordination by the sulfur atom in **1** to the catalyst’s Ir center, as has been invoked for thiophene substrates.^22a^ Throughout, the ligand Me_4_phen gave higher yields and regioselectivity than did other commonly used ligands for C–H borylation (dtbpy, dmbpy, dMeObpy, and SiO_2_-SMAP^23^).

As the reactivity profile of organoboron reagents may be honed or modulated for different applications through the substituents at boron,^24^ we also examined the conversion of **3a** to its relatives **3e**–**g** (Scheme 1b). Deprotection of **3a** to the corresponding boronic acid **3e** using diethanolamine (DEA) and acid hydrolysis^18h,25^ occurred in very good yield (85%), as did conversion to the trifluoroborate salt **3f** (90%) under the mild conditions developed by the group of Lloyd-Jones.^26^

The MIDA-protecting group can act as a valuable stabilizer of otherwise labile C–B bonds,^27^ and B(MIDA) boronates themselves have earned prominence as valuable building blocks in iterative^24d,24j,28^ and even automated^29^ synthesis.^30^ We obtained novel BTD-based B(MIDA) boronate **3g** in 65% yield through ligation of the B center in **3e** with methyliminodiacetic acid and concomitant water abstraction.^24i^

### *Ipso* Substitutions

**Scheme 2:**
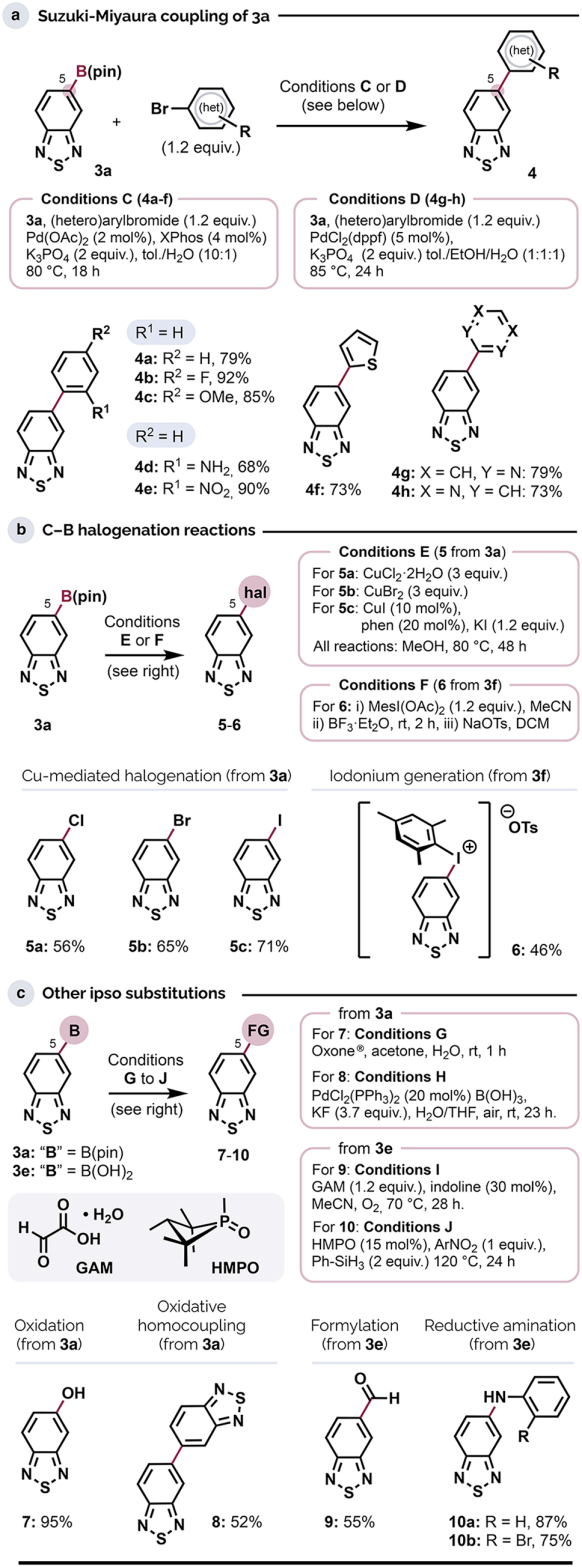
*Ipso* Substitutions of the C–B Bond in Boronates **3** GAM = glyoxylic acid monohydrate; HMPO = *anti-*1,2,2,3,4,4-Hexamethylphosphetane 1-oxide.

As an initial foray into leveraging the versatility of the C–B bond in building blocks **3**, we explored a range of *ipso* substitutions, beginning with extending BTD’s π-system via Suzuki-Miyaura cross-coupling of **3a** (Scheme 2a).^31^ Simple Pd(OAc)_2_/XPhos or PdCl_2_(dppf)-based systems gave biaryls **4a**–**h** in good-to-excellent yields from a range of electronically diverse (hetero)aryl bromides. Notably few 5-aryl BTD derivatives have been reported to date, and the influence of (hetero)aryl substituents at C5, e.g., on the photophysical properties of BTD-based compounds, has been explored only very sparingly, usually as one-off examples.^2d,32^ The pyrimidyl group of **4g** was installed as a directing group to enable extensions of the π-system through subsequent C4–H and C6–H functionalizations (see Scheme 4).

Next, we expanded the reaction scope using Cu-catalyzed^33^ and mediated^34^*ipso* halogenation, which provided the corresponding 5-chloro (**5a**), -bromo (**5b**), and -iodo (**5c**) analogues (Scheme 2b). The novel iodonium salt^35^**6** could be accessed via transfer of the BTD moiety from **3f** to the I^III^ center of diacetoxy(mesityl)-λ^3^-iodane, MesI(OAc)_2_, prior to anion exchange with NaOTs (Scheme 2b). To the best of our knowledge, only a single example of a BTD-based iodonium salt has been reported previously, which Zhang and Liang used to effect nucleophilic substitutions.^16k^ As described below, **6** gave new options for derivatizing the BTD benzenoid ring by acting as a precursor to the corresponding 4,5-benzothiadiazolyne (see Scheme 5). Scheme 2c shows four further functionalizations of **3a** and **3e**. Oxidation of **3a** using Oxone gave phenolic derivative **7** in near-quantitative yield, which we expect to serve as a convenient basis for generating new BTD-based push–pull systems. Boronic ester **3a** also underwent oxidative Pd-catalyzed homocoupling^36^ to the heterobiaryl system **8**, which has been of interest recently as a component of electrochromic polymers.^37^ The synthesis of **8** previously proceeded by condensation of SOCl_2_ and 3,3′,4,4′-tetraaminobiphenyl. Boronic acid **3e** was amenable to organocatalytic formylation recently reported by Mariano, Xie, and Wang,^38^ furnishing aldehyde **9**. Existing syntheses of **9** have relied on oxidation of the corresponding alcohol, in turn, obtained from the benzylic bromide, which is derived from a methyl group. Our route to **9** thus shortcuts a tedious multistep sequence.^39^ Finally, diaryl amines **10a** and **10b** were generated via a phosphacycle-catalyzed intermolecular reductive amination using nitroarenes as coupling partners.^40^ The high yields of these reactions are particularly pleasing, as efforts to generate 5-aryl BTDs via Chan-Lam^41^ or Buchwald-Hartwig^32f,42^ aminations (from 5-Br-BTD) gave only low-to-moderate yields (see the Supporting Information for details).^32d,43^

### Fused BTD Motifs

**Scheme 3:**
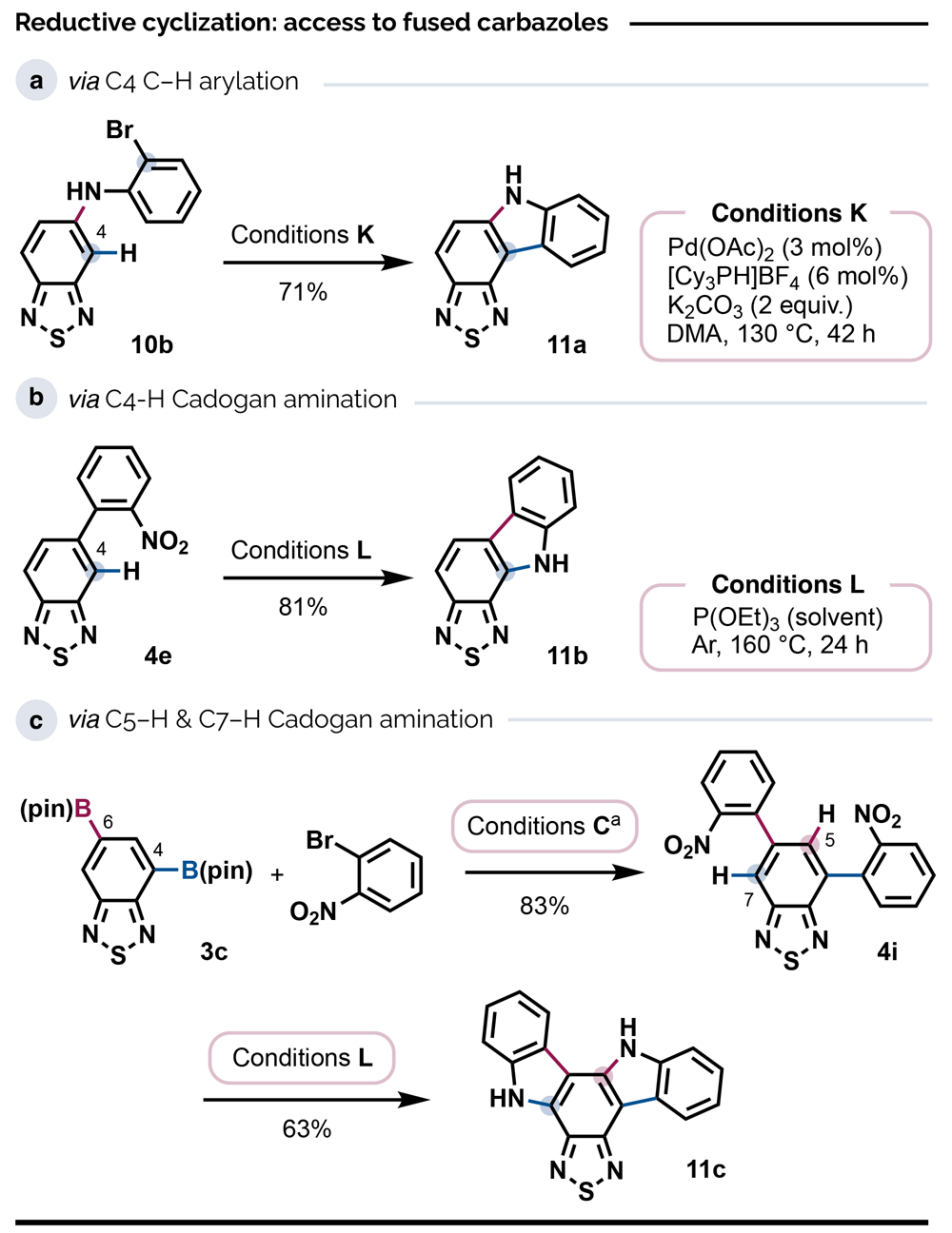
Access to Tetracyclic and Hexacyclic Thiadiazolocarbazoles via Derivatization of 5-Boryl or 4,6-Diboryl BTDs Modifications: Pd(OAc)_2_ (4 mol %), XPhos (4 mol %), bromoarene (2.4 equiv.), K_3_PO_4_ (3.0 equiv.), 80 °C, 21 h.

Fused carbazoles are key components of innumerable organic solar cells and other optoelectronic devices.^8a,44^ Tetracycles **11a** and **11b** (Scheme 3) specifically represent a class of fused thiadiazolocarbazoles that have shown promise as electron transporters in electroluminescent materials.^45^ We obtained **11a** from **10b** via intramolecular Pd-catalyzed C4–H arylation^46^ (Conditions K) with complete regioselectivity, which is accounted for by the greater ease with which C4–H activates under base-assisted palladation.^16,17^ To the best of our knowledge, this marks the first example of direct C–H arylation as a route to fused BTD-based systems.

The regioisomer **11b** could be accessed directly by reductive cyclization of nitroarene **4e** (Scheme 3) in 81% yield via the Cadogan reaction^35b,47^ (Conditions L). The cyclization occurred with exclusive regioselectivity, which is especially rare for Cadogan reactions of substrates with multiple sterically unhindered sites. In the case of **11b**, we attribute this to a significant difference in nucleophilicity between the C4 and C6 positions of the BTD unit. This difference notwithstanding, the novel hexacyclic system **11c** was also accessible via the Cadogan reaction of dinitro arene **4i** (obtained from diboronate **3c**), which demonstrates that both aromatic positions of the BTD framework may be addressed this way. By contrast, efforts to cyclize the aniline **4d** to **11b** using an oxidative Ir-catalyzed C–H amination^48^ gave a much lower yield of 25%, possibly due to inhibitive coordination to Ir and/or the Cu oxidant by the nitrogen or sulfur lone pairs.

### Directed C–H Functionalizations

**Scheme 4:**
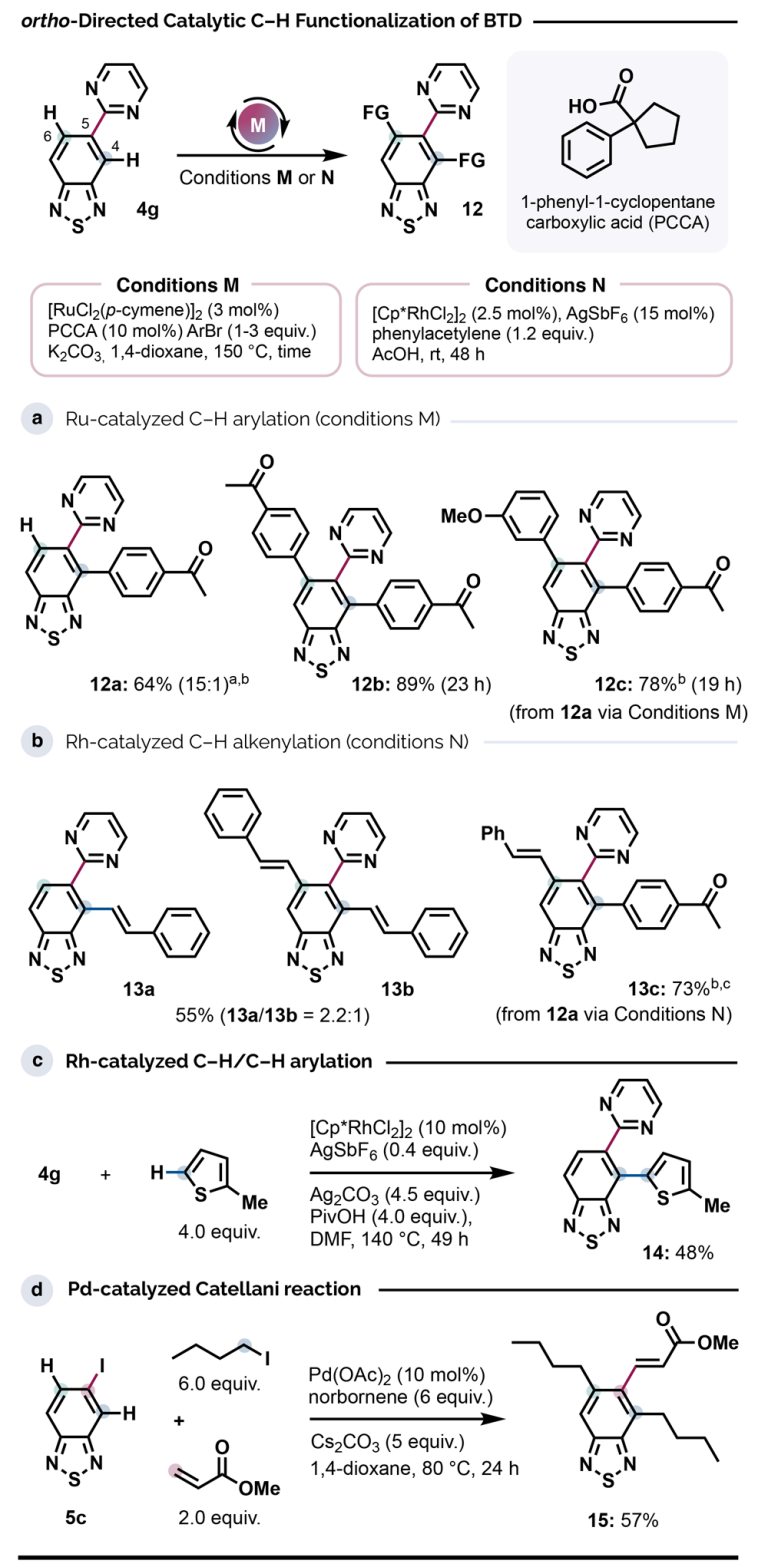
Directed C–H Functionalization Reactions 4-Bromoacetophenone (1.0 equiv.) added in two portions (16 h, then 25 h). Ratio of C4/C6 arylation. Major regioisomer shown. *rt* to 60 °C. Scale: 0.5 mmol BTD.

The use of catalytic C–H functionalization as a tactic to extend the BTD π-system is in its infancy. To complement the range of reactions reported to date,^16^ as well as to explore the prospects of addressing *both* C4–H and C6–H positions from a common directing group, we initially examined carboxylate-assisted^17^ Ru-catalyzed arylations and Rh-catalyzed alkenylation using **4g** as a substrate (Conditions M and N, respectively, Scheme 4). With one equivalent of bromoarene, the Ru system gave C4–H-arylated product **12a** with very high regioselectivity (15:1 *rr*), underscoring the greater preference of carboxylate-assisted C–H activation for the C4 position. However, this could be readily adapted to yield the 4,5,6-triaryl BTD system **12b** by using 3 equiv of bromoarene. Otherwise, sequential arylations under Conditions M could be used to introduce two electronically differentiated aromatic units (**12c**). The identities of these regioisomers were verified by using 1D-NOESY NMR spectroscopy, which showed no undirected arylation at C7–H, underscoring the complementarity between directed and undirected C–H arylation approaches. Similarly, the room-temperature Rh-catalyzed C–H alkenylation (Conditions N) was C4–H selective (**13a,b**) but could be used to address C6–H in **12a** to give the 4,5,6-trisubstituted BTD system **13c** in good yield. The C4–H position of **4g** was also amenable to directed Rh-catalyzed oxidative C–H/C–H coupling with 2-methylthiophene (product **14**, Scheme 4c),^49^ which complements the undirected Ru system described by Singh.^16h^ Access to structures such as **4f** and **14** should prove valuable as many BTD-based functional molecules comprise thiophenyl groups.

Subjecting **4h** to Conditions M did not result in any C–H functionalization, confirming the importance of the *ortho*-directing group effect.

Finally, to expand the set of Pd-catalyzed approaches to BTD C–H functionalization, we examined the prospect of generating 4,5,6-trifunctionalized BTD via the Catellani reaction, in which Ar–H bonds *ortho* with respect to an iodide are substituted via catalytic Pd/norbornene cooperation (Scheme 4d).^50^ Iodide **5c** thus underwent C4,C6-dialkylation, prior to a C5–H alkenylation with methyl acrylate to give **15**. The dibutylation of the BTD backbone is significant as the solubility and photophysical properties of BTD-based polymers can be enhanced through the inclusion of alkyl side chains.^51^ However, the paucity of available synthetic methods for modifying the BTD system itself has meant that alkyl groups have been typically attached to pendant rings, such as thiophenes.^51,52^ We anticipate that the Catellani approach described here will offer valuable new options in this context.

To the best of our knowledge, the reactions described here are the first demonstrations that Ir-, Ru-, Pd-, and Rh-catalyzed C–H functionalization is a viable approach for addressing C5 and C6 positions of BTD. As many directing groups are compatible with transition-metal functionalizations of this kind and because of the modularity with which the incoming substituents can be introduced, we anticipate the C–H functionalization approach exemplified here will open valuable new opportunities to incorporate variously modified BTDs into functional molecules. For example, one rare report from Shaoming and co-workers^53^ describes that polymers based on 4,6- rather than 4,7-substituted BTDs exhibit markedly greater solubility, enabling the synthesis of polymer chains with higher molecular weights. Investigations of the photophysical properties arising from novel BTD substitution patterns are ongoing in our laboratory.

### 2,1,3-Benzothiadiazol-4,5-yne

Arynes are remarkably versatile electrophiles able to accept a huge variety of functional groups to two neighboring aromatic carbons in a single procedure.^54^ Accordingly, arynes have secured roles as key intermediates in the syntheses of natural products,^55^ biaryl systems,^56^ polycyclic aromatic hydrocarbons,^57^ and beyond.^58^ A variety of methods for the generation of arynes has been reported,^59^ including deprotonation *ortho* to the I(III) center of diaryliodonium salts,^59b,60^ which is mild and operationally simple. This ejects an iodoarene as the nucleophile and creates a strained triple bond in the aromatic ring, which is trapped by an arynophile. For unsymmetrical (hetero)arynes, the regioselectivity of the trapping step can be rationalized using the Garg and Houk’s Aryne Distortion Model,^61^ which holds that initial nucleophilic attack by an arynophile will occur at the more linear of the two distorted triple bond carbon atoms. The extent of distortion at these positions can be calculated with relative ease for ground-state aryne intermediates, and the difference between the two internal bond angles, Δθ, correlates with expected regioselectivity. Values of Δθ > 4° can be considered as predicting “synthetically useful” levels of regioselectivity;^62^ in practice Δθ > 8° typically results in complete or near-complete selectivity for the “flatter” carbon atom.

Unfortunately, very few heteroaryne building block molecules have been developed, especially those that can be activated under mild conditions. Even simple heteroaryne precursors can be challenging or tedious to synthesize. To the best of our knowledge, indolynes and pyridynes are the only heteroarynes with commercially available mild-to-activate precursors.

**Scheme 5:**
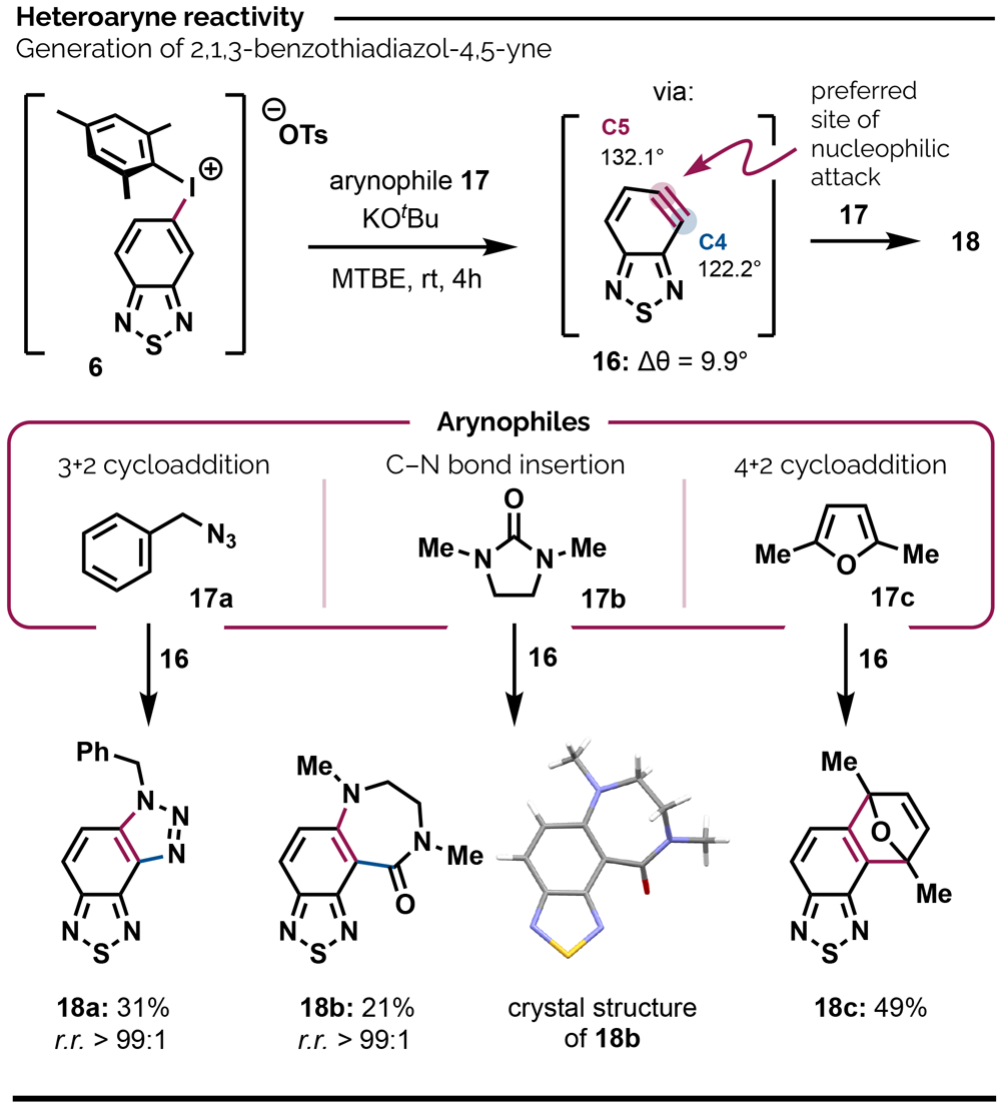
Generation, Distortion, and Trapping of 2,1,3-Benzothiadiazol-4,5-yne MTBE = Methyl *tert*-butylether.

We anticipated that the strongly electronegative N atoms, especially N3, would induce considerable distortion,^63^ in the novel 2,1,3-benzothiadiazol-4,5-yne (**16**, Scheme 5), and that this intermediate could be accessed by regioselective C4–H deprotonation of iodonium salt **6**.^64^ DFT calculations at the B3LYP/6–311++G(d,p) level predicted significant distortion [Δθ = 132.1° (C5) – 122.2° (C4) = 9.9°] of the triple bond in **16** in favor of C5-selective nucleophilic attack. To test this prediction and the viability of exploiting BTD-based arynes in synthesis, we subjected **6** to KO^t^Bu in MTBE in the presence of three different arynophiles: azide **17a**, cyclic urea **17b**, and furan **17c**. In each case, deprotonation occurred exclusively at the more acidic C4–H position, leading to products **18a–c**. For products **18a** and **18b**, we observed only the formation of the shown regioisomers, both of which result from nucleophilic attack at C5, as predicted by DFT and the Aryne Distortion Model. The structure of **18b** was further confirmed by using X-ray crystallography. Products **18a–b** were the only isolable species from otherwise intractable mixtures. The [4 + 2] cycloaddition with **17c** gave **18c** in a fair yield. Attempts to generate aryne **16** via C5–H deprotonation of the 4-iodaneyl BTD isomer of **6** (see compound **6′** in Supporting Information) in the presence of **17c** gave only traces of **18c**. More broadly, aryne generation from aryliodonium salts can give lower yields than, for example, fluoride-activated *ortho*-silylaryl triflates,^59a^ but the latter can be more demanding to prepare. The modest yields of **18a–b** notwithstanding, the reactions in Scheme 5 confirm the viability of BTD-based arynes and the theoretical prediction of the regioselectivity with which they react. They also expand the range of approaches available for decorating BTD’s benzenoid ring as well as the portfolio of accessible heteroaryne synthons.

## Conclusions

This methodology study addresses several long-standing challenges around functionalizing the BTD core. We have demonstrated a new route to diversely functionalized BTDs, which allows predictable and systematic substitutions at the C4, C5, C6, and C7 positions. These include a broad set of substitutions at C5, the first examples of directed and sequential C–H functionalization at the C4, C6, and C7 positions, and a demonstration that BTD-based arynes can be generated and captured with excellent levels of chemo- and regioselectivity.

At the root of these new possibilities is access to BTD-based organoboronate building blocks via mild and selective catalytic C–H borylation. We expect our description of their reactivity to serve as a platform for the synthesis of new functional molecules based on BTD’s unique properties. Work on the exploration of new substituent effects on the photophysical properties of BTD derivatives is ongoing in our laboratory.

## Data Availability

The data underlying this study are available in the published article and its Supporting Information.

## Supporting Information Available

General information, detailed experimental procedures, characterization data, ^1^H NMR and ^13^C NMR spectra, DFT calculations, and crystallographic data (PDF)
