# Supplementary material for: Derivatization of 2,1,3-Benzothiadiazole via Regioselective C–H Functionalization and Aryne Reactivity
Source: J Org Chem. 2024 Apr 22;89(9):6138–48. doi: 10.1021/acs.joc.4c00122 (PMC11077497; doi:10.1021/acs.joc.4c00122)
Supplement: Supplementary file 1 — jo4c00122_si_001.pdf [file jo4c00122_si_001.pdf]

Supporting Information

Derivatization of 2,1,3-Benzothiadiazole via Regioselective C–H Functionalization and Aryne Reactivity

Susanna V. Kunz,[a] Fredrik Barnå,[a] Mauricio Posada Urrutia,[a] Fredric J. L. Ingner,[a] Andrea Martínez-Topete,[a] Andreas Orthaber,[b] Paul J. Gates,[c] Lukasz T. Pilarski*[a] and Christine Dyrager*[a]

[a] Department of Chemistry – BMC, Uppsala University, Box 576, 75123, Uppsala, Sweden.

[b] Department of Chemistry – Ångström, Uppsala University, Box 523, 75120, Uppsala, Sweden.

[c] School of Chemistry, University of Bristol, Cantock's Close, Clifton, Bristol, BS8 1TS, United Kingdom.

Contents

I. General information S3

Abbreviations S3

Materials S4

Chromatography S4

Characterization S4

Crystallographic data S5

II. Synthesis S5

1) C–H Borylation of BTD (**1**) S5

2) Ipso substitutions S12

Alternative Buchwald-Hartwig amination procedure for **10b** S20

3) Fused BTD motifs S21

Alternative oxidative Ir-catalyzed C–H amination for **11b** S22

4) Directed C–H functionalizations S23

5) Generation and capture of 3,1,2-Benzothiadiazol-4,5-yne S28

III. Crystallographic details for **18b** S30

IV. DFT Calculation S32

V. References S33

VI. NMR Spectra S36

I. General information

Abbreviations

$B_2(pin)_2$	bis(pinacolato)diboron
B(pin)	pinacol boronic ester
BTD	2,1,3-benzothiadiazole
dmbpy	4,4'-dimethyl-2,2'-bipyridyl
dMeObpy	4-4′-dimethoxy-2-2′-bipyridine
dppf	1,1′-ferrocenediyl-bis(diphenylphosphine)
dtbpy	4,4′-di-*tert*-butyl-2,2′-dipyridyl
HMBC	heteronuclear multiple bond correlation
$[IrCp^*Cl_2]_2$	di-*μ*-chloro-bis[chloro(pentamethylcyclopentadienyl)iridium(III)]
$[Ir(OMe)COD]_2$	bis(1,5-cyclooctadiene)di-*μ*-methoxydiiridium(I)
Me_4phen	3,4,7,8-tetramethyl-1,10-phenanthroline
mida	*N*-methyliminodiacetic acid
MTBE	methyl-*tert*-butylether
NMP	*N*-methyl-2-pyrrolidone
o.n.	overnight
PEPPSI-IPr	[1,3-bis(2,6-diisopropylphenyl)imidazol-2-ylidene](3-chloropyridyl) palladium(II) dichloride
rt	room temperature
$[RhCp^*Cl_2]_2$	pentamethylcyclopentadienylrhodium(III) chloride dimer
$[RuCl_2(p\text{-cymene})]_2$	dichloro(*p*-cymene)ruthenium(II) dimer
XPhos	dicyclohexyl[2′,4′,6′-tris-(propan-2-yl) [1,1′-biphenyl]-2-yl]phosphane
Silica-SMAP	silica supported phosphine ligand[1]

Materials

All reagents were purchased from Sigma-Aldrich, Fluorochem or TCI and used without further purification unless specified. Solvents were obtained from VWR Chemicals and Sigma-Aldrich and used without further purification unless specified. Commercial $B_2(pin)_2$ was dried by heating at 50 °C under reduced pressure overnight. Commercial Me_4phen was recrystallized from benzene. Anhydrous THF and dioxane were passed over activated alumina, stored over molecular sieves and degassed by purging with argon prior to utilization.

Unless otherwise stated, glassware was dried in an oven overnight prior to use. Reactions were conducted in microwave vials (0.5-2 mL, 2-5 mL or 5-20 mL capacity) sealed with Teflon capped aluminum lids. Reactions under argon were placed under inert atmosphere through three or more cycles of evacuating the reaction vessel and refilling with argon.

Chromatography

Purification by flash column chromatography was performed on a Biotage Isolera One flash chromatography system. The separation was carried out on Biotage Sfär Silica HC High Capacity 20 µm cartridges through dryload on Celite. Manual column chromatography was carried out using silica gel 60 (particle size 0.063−0.100 mm). Borylation products were purified by column chromatography using boric acid impregnated silica as previously described in the literature.[2] Thin-layer chromatography (TLC) was performed on aluminum backed silica plates (silica gel 60, 0.20 mm, UV 254) and visualized under UV light (λ = 254 nm or 365 nm). Borylation products were visualized on TLC with *p*-anisaldehyde staining solution.

Characterization

^{1}H, ^{13}C and ^{19}F NMR spectra were recorded on a Varian Unity 400 MHz (^{1}H: 400 MHz, ^{13}C: 101 MHz, ^{19}F: 376 MHz). Chemical shifts are referenced to residual solvent signals (^{1}H: $CDCl_3$ at 7.26 ppm, acetone-d_6 at 2.05 ppm, CD_2Cl_2 at 5.32 ppm, DMSO-d_6 at 2.50 ppm; ^{13}C: $CDCl_3$ at 77.16 ppm, acetone-d_6 at 29.84 ppm, CD_2Cl_2 at 53.84 ppm, DMSO-d_6 at 39.52 ppm) and reported in ppm. Multiplicities are reported as follows: s = singlet, br s = broad singlet, d = doublet, t = triplet, q = quartet, m = multiplet. Structural assignments were made with additional information from gCOSY, gHSQC, gHMBC and (1D-)NOESY experiments.

High-resolution mass spectrometry (HRMS) data (ESI-TOF) was determined at the School of Chemistry, University of Bristol (Bristol, UK) or at the Division of Mass Spectrometry, Department of Chemistry, Imperial College (London, UK). High-resolution electrospray ionisation mass spectrometry was performed on a micrOTOF II Focus instrument, nanospray ionization was performed on a Synapt G2S instrument using a Triversa chip based nanospray source and electron ionisation mass spectrometry was performed at 70eV on a QExactive GC Orbitrap instrument (70 eV, Thermo Scientific).

Crystallographic data

Measurements were performed using graphite-monochromatized Mo Kα radiation at 180 K using a Bruker D8 APEX-II equipped with a CCD camera. The structure was solved by direct methods (SHELXS-2014) and refined by full-matrix least-squares techniques against F2 (SHELXL-2018). The non-hydrogen atoms were refined with anisotropic displacement parameters. The H atoms of the CH_2 / CH groups were refined with common isotropic displacement parameters for the H atoms of the same group, and idealized geometry. The H atoms of the methyl groups were refined with common isotropic displacement parameters for the H atoms of the same group, and idealized staggered geometry.

CCDC 2324934 contains the supplementary crystallographic data for this paper. The data can be obtained free of charge from The Cambridge Crystallographic Data Centre via www.ccdc.cam.ac.uk/structures.

Procedure for crystal growth of **18b**:

10 mg of **18b** was dissolved in 500 µL $CHCl_3$ in a cylindrical vial (inner diameter x height: 6 x 40 mm), open at one end. It was placed in a 20 mL vial filled with *n*-pentane (4 mL). The larger vial was capped and placed at -18 °C for 3 weeks. The smaller vial was removed from the larger one, and the solution was removed from the formed crystal with a pipette.

II. Synthesis

1) C–H Borylation of BTD (1)

General procedure for the Ir-catalyzed C–H borylation of BTD (Table S1)

Modified literature procedure.[8] In an oven-dried microwave vial equipped with a stirrer bar dried $B_2(pin)_2$ (1.3 equiv.), $[Ir(OMe)COD]_2$ (1 mol%) and Me_4phen (2 mol%) were weighed, sealed and placed under argon atmosphere. Degassed anhydrous THF (1.15 mL per mmol B_2pin_2) was added and the mixture was placed in an oil bath at 70 °C. After stirring for 1 h the solution was allowed to reach rt and was transferred via syringe to a second dry microwave vial containing 2,1,3-benzothiadiazole **1** (1.0 equiv.) under argon. Residues from the first vial were then added by rinsing with additional THF (such that the concentration noted in Table S1 was obtained). The reaction mixture was heated at 70 °C for the noted time. The reaction was allowed to reach rt and quenched by addition of H_2O during which hydrogen gas evolution was observed. The mixture was diluted with H_2O, extracted with CH_2Cl_2 and the combined organic phases were dried ($MgSO_4$) and filtered. 1,3,5-Trimethoxybenzene was added to the filtrate as an internal ^{1}H NMR standard. The solvent was removed under reduced pressure and the yield was determined via ^{1}H NMR spectroscopy.

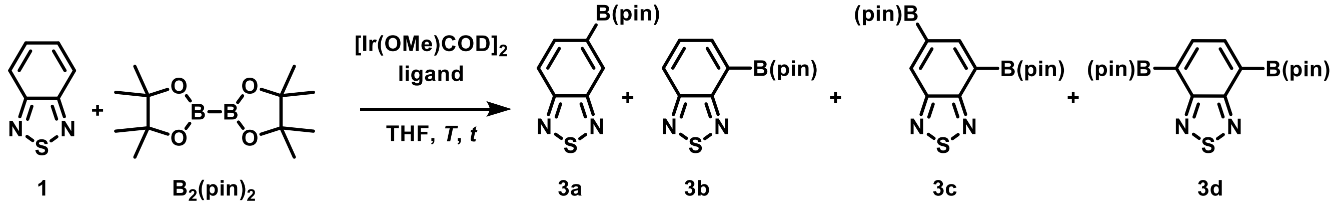

Table S1. Optimization of conditions for the iridium-catalyzed C–H borylation of BTD (**1**).

Entry	$B_2(pin)_2$ (equiv.)	$[Ir(OMe)COD]_2$ (mol%)	ligand	ligand (mol%)	conc. BTD (M)	*T* (°C)	*t* (h)	Yields (%)[a] 3a	3b	3c	3d
1	1.05	1	Me_4phen	2	0.5	80	o.n.	59	7	11	4
2	1.05	1	**dtbpy**	2	0.5	80	o.n.	44	11	6	2
3	1.05	1	Me_4phen	2	0.5	80	**2**	58	11	7	3
4	1.05	1	Me_4phen	2	0.5	80	**3**	59	11	8	3
5	1.05	1	Me_4phen	2	0.5	80	**6**	57	11	7	3
6	1.05	1	Me_4phen	2	0.5	80	**24**	53	10	7	3
7	1.05	1	Me_4phen	2	0.5	**40**	2	6	2	0	0
8	1.05	1	Me_4phen	2	0.5	**60**	2	31	8	1	0
9	1.05	1	Me_4phen	2	0.5	60	**6**	25	8	1	0
10	1.05	1	Me_4phen	2	0.5	**65**	6	57	10	8	2
11	1.05	1	Me_4phen	2	0.5	**70**	2	59	10	7	3
12	**0.8**	1	Me_4phen	2	0.5	70	2	50	12	4	1
13	0.8	1	Me_4phen	2	0.5	**65**	**6**	49	10	4	2
14	**1.05**	1	Me_4phen	2	**0.25**	**70**	6	51	10	3	2
15	**1.3**	1	Me_4phen	2	**0.5**	70	6	60	6	13	4
16	1.3	1	Me_4phen	2	**0.25**	70	6	64 (59)	6	8	8
17[b]	1.3	1	Me_4phen	2	**0.25**	70	6	56 (52)	3	16	3
18	1.3	1	Me_4phen	2	**0.1**	70	**o.n.**	61	5	14	3
19	1.3	1	Me_4phen	2	**0.75**	70	**4**	55	12	5	2
20	1.3	1	**dMeObpy**	2	0.25	70	4	57	10	10	2
21	1.3	1	**dmbpy**	2	0.25	70	4	59	8	11	3
22	1.3	1	**Silica-SMAP**	2	0.25	70	4	0	0	0	0
23	1.3	**0.5**	Me_4phen	**1**	0.25	70	4	38	9	1	0
24	1.3	**3**	Me_4phen	**6**	0.25	70	4	57	5	14	4
25	1.3	**5**	Me_4phen	**10**	0.25	70	4	39	4	22	5
26	**1.5**	1	Me_4phen	2	0.25	70	4	62	10	8	2
27	**2**	1	Me_4phen	2	0.25	70	4	62	8	12	3
28	**3**	**5**	Me_4phen	**10**	0.25	70	4	15	0	53	6
29	3	5	Me_4phen	10	0.25	**80**	**6**	0	0	58 (47)	7

Reaction scale: 0.5 mmol BTD. [a] Yields determined by ^{1}H NMR spectroscopy using 1,3,5-trimethoxybenzene as an internal standard; isolated yields in brackets. [b] Reaction scale: 1 g BTD. Abbreviations: conc., concentration; *T*, temperature; *t*, time.

^{1}H NMR-assignment of BTD and regioselectivity of C–H-borylation

The ^{1}H NMR spectrum of BTD shows AA'BB'-type higher order multiplets, which have previously been assigned in the literature:[3,4] the more deshielded multiplets at 7.97 ppm were assigned to C4–H and C7–H, whilst the multiplets with the lower chemical shift at 7.54 ppm were assigned to C5–H and C6–H. Our measurements of the T1 relaxation time (inversion recovery measured with d1 = 26 s) as well as a ^{15}N HMBC spectrum (Figure S1) confirm these earlier assignments.

T1(7.97 ppm) = 5.45 s; T1(7.54 ppm) = 4.77 s

Steel, Marder and co-workers have previously described that the regioselectivity of C–H borylation of pyridines and quinolines can be predicted to occur at the most sterically accessible C–H bond with the highest chemical shift in the ^{1}H NMR spectrum.[5] Even though in BTD the C4–H and C7–H protons are more acidic and show the higher chemical shift in the ^{1}H NMR spectrum, C–H borylation takes place predominantly at C5. We attribute this to inhibition of C4–H borylation by the lone pairs of N3, through either repulsion of the bulky catalyst or coordination to the Ir center. Marder and Steel described analogous effects in their study of heterocyclic systems such as quinolines and pyridines.[5]

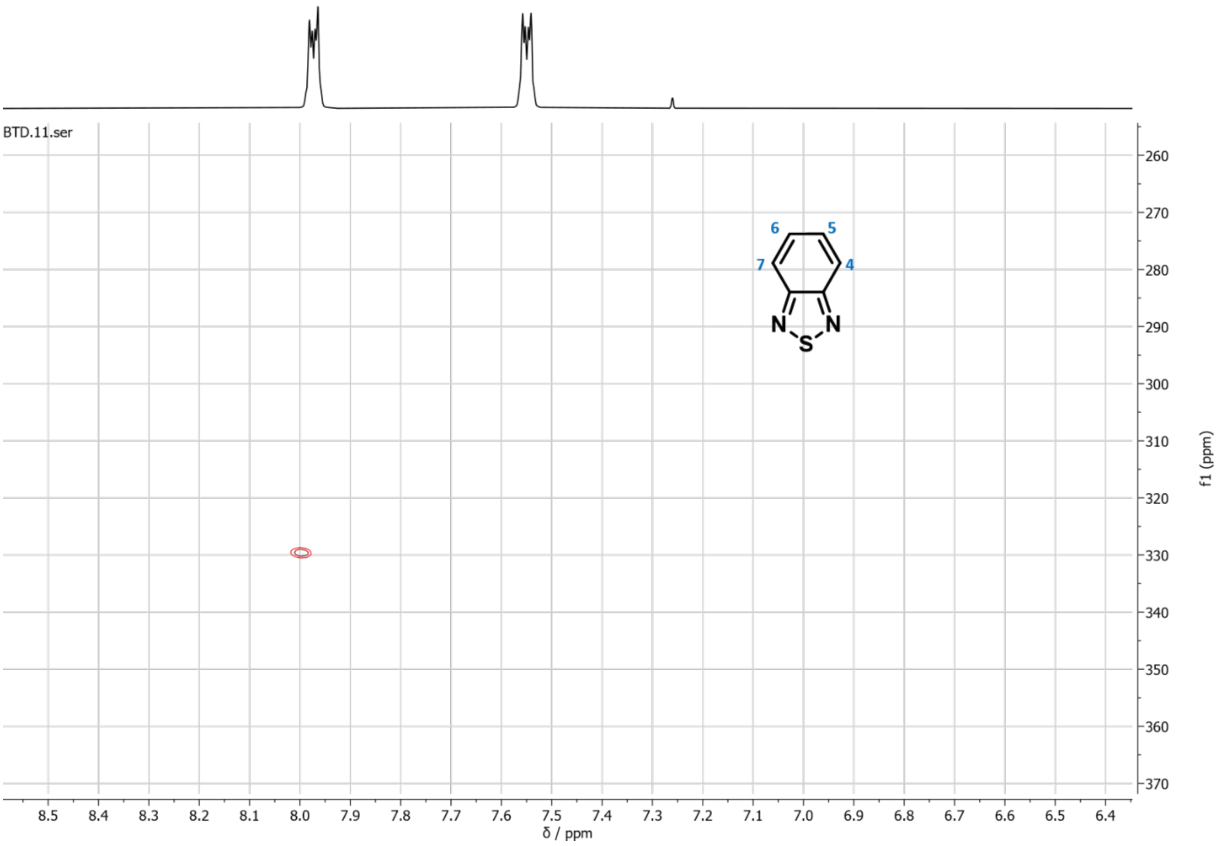

Figure S1: ^{15}N-HMBC spectrum of BTD in $CDCl_3$ showing the coupling of C4/C7 to the adjacent N3/1.

Diborylation experiments starting from 5-Bpin-BTD compared to 4-Bpin-BTD

A second borylation starting from monoborylated 5-B(pin)-BTD **3a** was investigated. The conditions for the second borylation followed the general borylation procedure with the modifications listed below.

Even with 2.5 equivalents of $B_2(pin)_2$ and higher catalyst loadings of 2 mol% $[Ir(OMe)COD]_2$ (*i.e.*, 4 mol% with respect to Ir) and 4 mol% Me_4phen, 5-B(pin)-BTD barely formed any diborylated product **3c**. The crude NMR of the borylation mixture reveals a conversion of only 7% (Figure S2a). Under the same conditions the borylation of 4-B(pin)-BTD **3b** yielded a mixture with a ratio of approximately 1:1 4,6-$(B(pin))_2$-BTD **3c** and 4,7-$(B(pin))_2$-BTD **3d** with full conversion of the starting material (Figure S2b).

*Note: 4-B(pin)-BTD **3b** and 4,6-$(B(pin))_2$-BTD **3d** are commercially available and were not isolated from the borylation of BTD. Spectral data obtained during the borylation experiments is in accordance with previously reported values.*[6,7]

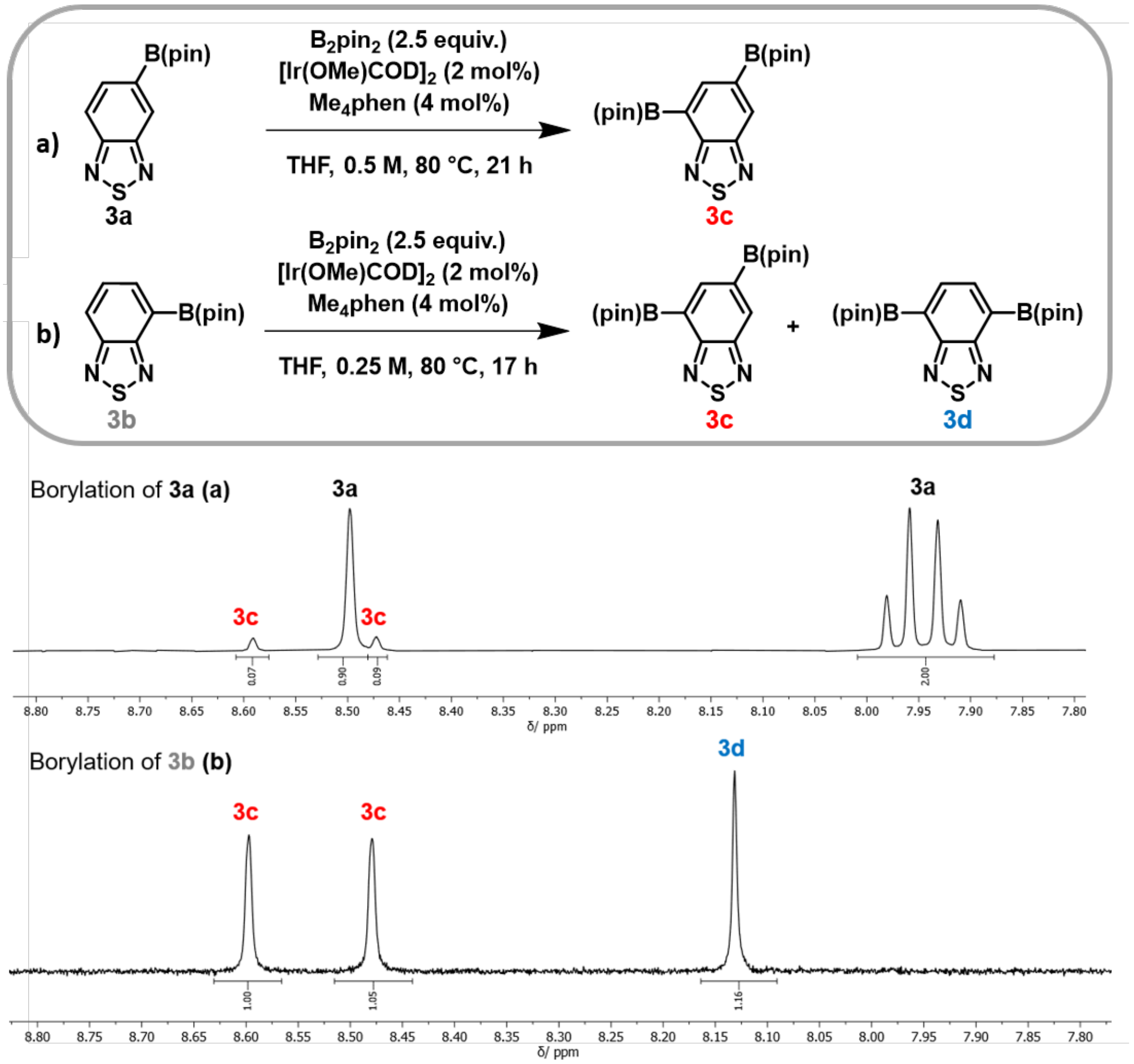

*Figure S2: ^{1}H NMR spectra of crude borylation products of 5-B(pin)-BTD **3a** (top) and 4-B(pin)-BTD **3b** (bottom).*

Optimized procedure for the Ir-catalyzed C–H borylation of BTD (Table S1, Entry 16); synthesis of 5-(4,4,5,5-tetramethyl-1,3,2-dioxaborolan-2-yl)-2,1,3-benzothiadiazole (3a)

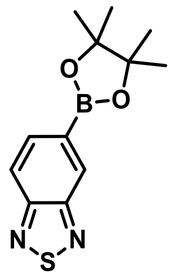

3a

Modified literature procedure.[8] In an oven-dried microwave vial equipped with a stirrer bar dried $B_2(pin)_2$ (165 mg, 0.65 mmol, 1.3 equiv.), $[Ir(OMe)COD]_2$ (1 mol%, 3.3 mg, 5.0 μmol) and Me_4phen (2 mol%, 2.4 mg, 0.01 mmol) were weighed, sealed and placed under argon atmosphere. Degassed anhydrous THF (0.75 mL) was added and the mixture was placed in an oil bath at 70 °C. After stirring for 1 h the solution was allowed to reach rt and was transferred via syringe to a second dry microwave vial containing 2,1,3-benzothiadiazole **1** (68 mg, 0.50 mmol, 1.0 equiv.) under argon. Residues from the first vial were then added by rinsing with additional THF (1.25 mL). The reaction mixture was heated at 70 °C for 6 h. The reaction was allowed to reach rt and quenched by addition of H_2O (0.5 mL) during which hydrogen gas evolution was observed. The mixture was diluted with H_2O (20 mL), extracted with CH_2Cl_2 (3 x 20 mL) and the combined organic phases were dried ($MgSO_4$) and filtered. 1,3,5-Trimethoxybenzene (28.0 mg, 0.167 mmol) was added to the filtrate as an internal ^{1}H NMR standard. The solvent was removed under reduced pressure and the yield was determined via ^{1}H NMR spectroscopy. The crude product was purified by column chromatography using boric acid impregnated silica[2] (eluent: pentane/EtOAc 20:1, TLC stain: *p*-anisaldehyde) to afford **3a** as a colorless solid (78 mg, 0.30 mmol, 59%). R_f = 0.28 (hexane/EtOAc 15:1). ^{1}H NMR (400 MHz, $CDCl_3$) δ 8.50 (s, 1H), 8.04 – 7.85 (m, 2H), 1.39 (s, 12H). $^{13}C\{^1H\}$ NMR (101 MHz, $CDCl_3$) δ 156.3, 154.8, 133.9, 129.6, 120.8, 84.6, 25.1. The *C*–B signal was not observed due to quadrupolar relaxation. HRMS (TOF ES+) calcd for $[C_{12}H_{16}BN_2O_2S + H]^+$ 263.1026, found m/z 263.1015.

Gram scale Ir-catalyzed C–H borylation of BTD (Table S1, Entry 17):

Dried B_2pin_2 (2.42 g, 9.55 mmol, 1.3 equiv.), $[Ir(OMe)COD]_2$ (1 mol%, 48.7 mg, 73.4 μmol) and Me_4phen (2 mol%, 34.7 mg, 147 μmol) were weighed in an oven-dried 100 mL Schlenk tube and placed under N_2 atmosphere. Degassed anhydrous THF (11 mL) was added and the mixture was heated in an oil bath at 70 °C for 1 h. The solution was allowed to cool to room temperature and a solution of BTD (1.00 g, 7.34 mmol, 1 equiv.) in degassed anhydrous THF (18.4 mL) was added. The reaction solution was heated in an oil bath at 70 °C for 6 h. After the reaction solution had cooled down to room temperature H_2O (5 mL) were added and hydrogen gas evolution was observed. Additional H_2O (100 mL) was added and the aqueous phase was extracted with CH_2Cl_2 (3 x 100 mL). The organic phase was washed with brine (100 mL), dried ($MgSO_4$) and filtered. The solution was diluted with CH_2Cl_2 to 500 mL in a volumetric flask and 5 mL were removed. The spectroscopic yield was determined by adding 1,3,5-trimethoxybenzene (3.3 mg, 0.020 mmol) as an internal ^{1}H NMR standard to the 5 mL sample. The solvent was removed and the crude product was purified by column chromatography using boric acid impregnated silica[2] (eluent: pentane/EtOAc 20:1) to afford **3a** as a colorless solid (996 mg, 3.80 mmol, 52%).

Representative procedure for the Ir-catalyzed C–H diborylation of BTD (Table S1, Entry 28); synthesis of 4,6-bis(4,4,5,5-tetramethyl-1,3,2-dioxaborolan-2-yl)-2,1,3-benzothiadiazole (3c)

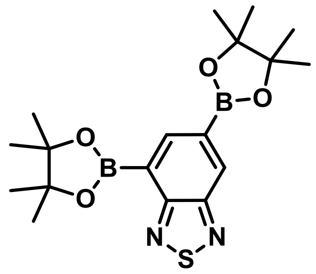

3c

Modified literature procedure.[8] In an oven-dried microwave vial equipped with a stirrer bar dry $B_2(pin)_2$ (381 mg, 1.50 mmol, 3.0 equiv.), $[Ir(OMe)COD]_2$ (6 mol%, 16.6 mg, 25.0 μmol) and Me_4phen (10 mol%, 11.8 mg, 50.0 μmol) were weighed, sealed and placed under argon. Degassed anhydrous THF (0.75 mL) was added and the mixture was placed in an oil bath at 80 °C. After stirring for 1 h the solution was allowed to reach rt and was transferred via syringe to a second dry microwave vial containing 2,1,3-benzothiadiazole **1** (68 mg, 0.50 mmol, 1.0 equiv.) under argon. Residues from the first vial were then added to the second vial by rinsing with additional THF (1.25 mL). The second vial was heated at 80 °C for 6 h. The reaction was allowed to reach rt and quenched with H_2O (1 mL) during which hydrogen gas evolution was observed. The mixture was diluted with H_2O (20 mL), extracted with CH_2Cl_2 (3 x 20 mL) and the combined organic phases were washed with brine (20 mL) and dried ($MgSO_4$). The solution was filtered and 1,3,5-trimethoxybenzene (28.0 mg, 0.167 mmol) was added to the filtrate as an internal ^{1}H NMR standard. The solvent was removed under reduced pressure and the yield was determined via ^{1}H NMR spectroscopy. The crude product was purified by column chromatography on boric acid impregnated silica[2] (eluent: pentane/EtOAc 15:1, TLC stain: *p*-anisaldehyde) to afford **3c** as a colorless solid (92 mg, 0.24 mmol, 47%). ^{1}H NMR (400 MHz, $CDCl_3$) δ 8.60 (d, *J* = 1.1 Hz, 1H), 8.48 (d, *J* = 1.2 Hz, 1H), 1.44 (s, 12H), 1.39 (s, 12H). $^{13}C\{^1H\}$ NMR (101 MHz, $CDCl_3$) δ 158.8, 154.5, 143.2, 132.6, 84.6, 84.4, 25.1, 25.0. The *C*–B signals were not observed due to quadrupolar relaxation. HRMS (EI+) calcd for $[C_{18}H_{26}N_2O_4SB_2]^+$ 388.1794, found m/z 388.1783.

2,1,3-Benzothiadiazol-5-ylboronic acid (3e)

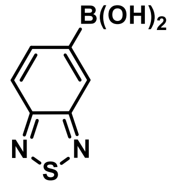

3e

Modified literature procedure.[9,10] To a 100 mL round-bottom flask equipped with several stirrer bars were added boronic acid ester **3a** (200 mg, 0.76 mmol, 1.0 equiv.) and MTBE (20 mL). Diethanolamine (80 µL, 88 mg, 0.84 mmol, 1.1 equiv.) was added in one portion. The solution was very vigorously stirred at rt for 18 h. The resulting colorless precipitate was dissolved in Et_2O (20 mL) and $HCl_{(aq)}$ (1 M, 20 mL). The biphasic mixture was stirred until all solids dissolved. The phases were separated and the aqueous phase was extracted with Et_2O (3 x 20 mL). The combined organic phases were dried (Na_2SO_4), filtered and concentrated under reduced pressure to afford **3e** as a colorless solid (117 mg, 0.65 mmol, 85%). ^{1}H NMR (400 MHz, acetone-d$_6$) δ 8.55 (s, 1H), 8.11 (d, *J* = 8.9, 1H), 7.99 (d, *J* = 8.8 Hz, 1H), 7.58 (s, 2H). $^{13}C\{^1H\}$ NMR (101 MHz, acetone-d$_6$) δ 155.8, 154.9, 134.0, 127.9, 120.1. The *C*–B signal was not observed due to quadrupolar relaxation. HRMS (Nanospray-MS) calcd for $[C_6H_5N_2O_2BS + Cl]^-$ 214.9855, found m/z 214.9859.

5-(Trifluoro-λ[4]-boranyl)-2,1,3-benzothiadiazole, potassium salt (3f)

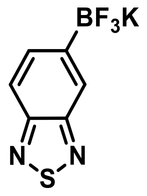

3f

Modified literature procedure.[11] To a 100 mL round-bottom flask equipped with a stirrer bar was added boronic acid ester **3a** (2.11 g, 8.03 mmol, 1.0 equiv.), MeOH (16.1 mL), and MeCN (16.1 mL). KF (1.87 g, 32.1 mmol, 4.0 equiv.) and H_2O (3.2 mL). In a 25 mL round-flask, L-tartaric acid (2.41 g, 16.1 mmol, 2.0 equiv.) was dissolved in THF (12.1 mL) by stirring at 40 °C. The L-tartaric acid solution was added dropwise to the solution of **3a**. A colorless precipitate formed. After stirring the solution for 2 min, MeCN (16 mL) was added. After an additional 2 min of stirring, MeCN (8 mL) was added. The solution was filtered, and the precipitate was washed with MeCN. The filtrate was concentrated under reduced pressure. The solid crude product was triturated with Et_2O, filtered and washed with Et_2O to afford **3f** as a colorless solid (1.75 g, 7.22 mmol, 90%). ^1H NMR (400 MHz, DMSO-d_6) δ 7.82 (s, 1H), 7.79 (d, *J* = 8.8 Hz, 1H), 7.70 (d, *J* = 8.7 Hz, 1H). $^{13}C\{^1H\}$ NMR (101 MHz, DMSO-d_6): δ 155.6, 154.5, 135.9, 122.0, 119.0. The *C*–B signal was not observed due to quadrupolar relaxation. ^{19}F NMR (376 MHz, DMSO-d_6): δ -139.4 (1:1:1:1 q, *J* = 17.8 Hz, ^{19}F bound to ^{10}B, *I* = 3/2), -139.8 – -140.9 (m, ^{19}F bound to ^{11}B). HRMS (Nanospray-MS) calcd for $[C_6H_3N_2BF_3SK\text{-}K]^-$ 203.0063, found m/z 203.0067.

4-(trifluoro-λ[4]-boraneyl)-2,1,3-benzothiadiazole, potassium salt (3f')

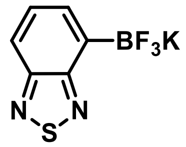

3f'

Modified literature procedure.[11] To a 50 mL flame-dried Schlenk flask equipped with a stirrer bar under argon were added 4-bromo-2,1,3-benzothiadiazole (2.00 g, 9.33 mmol, 1.0 equiv.), KOAc (2.74 g, 27.9 mmol, 3.0 equiv.), $B_2(pin)_2$ (2.84 g, 11.2 mmol, 1.2 equiv.) and anhydrous 1,4-dioxane (30 mL). The resulting suspension was purged with argon for 60 min. $PdCl_2(dppf)$ (6 mol%, 409 mg, 0.56 mmol) was added and the solution was stirred at 110 °C for 100 min, after which it was allowed to reach rt. The solution was filtered through a pad of Celite using Et_2O (120 mL). The filtrate was washed with brine (3 x 80 mL) and H_2O (50 mL). The washed organic phase was dried (Na_2SO_4), filtered and concentrated under reduced pressure, to afford a red/brown oil. The resulting oil was dissolved in MeOH (18.6 mL) and MeCN (18.6 mL). To this solution was added KF (2.17 g, 37.3 mmol, 4.0 equiv.) and H_2O (3.7 mL). In a separate 50 mL round-bottom flask heated to 40 °C, L-tartaric acid (2.80 g, 18.7 mmol, 2 equiv.) was dissolved in THF (13.9 mL). The tartaric acid solution was then added dropwise over 2 min to the main solution under stirring, after which MeCN (18 mL) was added. A brown precipitate formed. After 3 min, MeCN (9 mL) was added. The reaction mixture was filtered. The filtrate was stirred together with activated charcoal (3 g), filtered, concentrated under reduced pressure, and triturated first with Et_2O, then with MeOH, to afford **3f'** as a colorless solid (1.42 g, 5.88 mmol, 63%). ^1H NMR (400 MHz, DMSO-d_6) δ 7.78 (d, *J* = 8.6 Hz, 1H), 7.59 (d, *J* = 6.4 Hz, 1H), 7.54 – 7.42 (m, 1H). $^{13}C\{^1H\}$ NMR (101 MHz, DMSO-d_6) δ 158.7, 155.0, 131.5 (q, *J* = 3.0 Hz), 130.0, 118.6. ^{19}F NMR (376 MHz, DMSO-d_6) δ -137.7 – -138.7 (m, ^{10}B), -139.3 – -139.7 (m, ^{11}B). HRMS (ESI-): m/z calcd for $C_6H_3BF_3N_2S$ $[M\text{-}K]^-$ 203.0062; found 203.0064.

2-(2,1,3-Benzothiadiazol-5-yl)-6-methyl-1,3,6,2-dioxazaborocane-4,8-dione (3g)

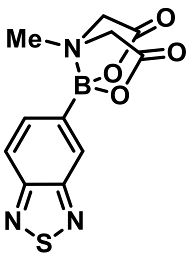

3g

Modified literature procedure.[12] To a 25 mL flame-dried microwave vial equipped with a stirrer bar under argon was added the boronic acid **3e** (40.0 mg, 0.22 mmol, 1.0 equiv.), *N*-methyliminodiacetic acid (32.7 mg, 0.22 mmol, 1.0 equiv.) and flame-dried $MgSO_2$ (107 mg, 0.89 mmol, 4.0 equiv.). The vial was sealed and the atmosphere was exchanged again for argon. Dry toluene (3.7 mL) and dry DMSO (0.37 mL) were added. The mixture was stirred at 140 °C for 18 h. The mixture was allowed to reach rt and filtered. The remaining solids were washed with CH_2Cl_2 and the filtrate was concentrated under reduced pressure. H_2O (15 mL) was added to the crude product, forming a colorless precipitate. The precipitate was filtered off and dried under vacuum to afford **3g** as a colorless solid (42 mg, 0.14 mmol, 65%). ^{1}H NMR (400 MHz, DMSO-d_6): δ 8.10 (s, 1H), 8.05 (d, *J* = 8.9 Hz, 1H), 7.75 (d, *J* = 8.8 Hz, 1H), 4.40 (d, *J* = 17.2 Hz, 2H), 4.20 (d, *J* = 17.2 Hz, 2H), 2.59 (s, 3H). $^{13}C\{^{1}H\}$ NMR (101 MHz, DMSO-d_6) δ 169.8, 155.2, 154.8, 133.8, 126.3, 120.8, 62.6, 48.1. The *C*–B signal was not observed due to quadrupolar relaxation. HRMS (ESI+) calcd for $[C_{11}H_{10}N_3O_4SB + H]^+$ 292.0558, found m/z 292.0553.

2) Ipso substitutions

5-Phenyl-2,1,3-benzothiadiazole (4a)

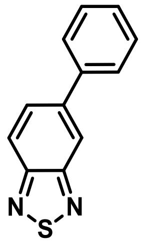

4a

Modified literature procedure.[9] Prior to use, toluene and H_2O were purged with argon for 20 min. Boronic acid ester **3a** (105 mg, 0.40 mmol, 1.0 equiv.), $Pd(OAc)_2$ (2 mol%, 1.8 mg, 8.0 μmol), K_3PO_4 (170 mg, 0.80 mmol, 2.0 equiv.) and XPhos (4 mol%, 7.6 mg, 16.0 μmol) were weighed in a microwave vial equipped with a stirrer bar, sealed and placed under argon atmosphere. Degassed toluene (2.0 mL), bromobenzene (51 μL, 75 mg, 0.48 mmol, 1.2 equiv.) and degassed H_2O (0.2 mL) were added to the vial. The reaction mixture was stirred for 18 h at 80 °C. The solution was allowed to reach rt, diluted with Et_2O (40 mL) and washed with brine (30 mL). The organic phase was dried ($MgSO_4$), filtered and the solvent was removed under reduced pressure. Product **4a** was obtained after purification by flash column chromatography (SiO_2, eluent: hexane/EtOAc gradient from 98:2 to 95:5) as an off-white solid (67 mg, 0.32 mmol, 79%). R_f = 0.37 (hexane/EtOAc 95:5). ^{1}H NMR (400 MHz, $CDCl_3$) δ 8.15 (s, 1H), 8.04 (d, *J* = 9.1 Hz, 1H), 7.86 (d, *J* = 9.2 Hz, 1H), 7.69 (d, *J* = 7.6 Hz, 2H), 7.50 (t, *J* = 7.2 Hz, 2H), 7.43 (t, *J* = 7.2 Hz, 1H).$^{13}C\{^{1}H\}$ NMR (101 MHz, $CDCl_3$) δ 155.5, 154.3, 142.5, 139.6, 130.2, 129.2, 128.5, 127.6, 121.6, 118.6. Spectral data are in accordance with previously reported values.[13]

5-(4-Fluorophenyl)-2,1,3-benzothiadiazole (4b)

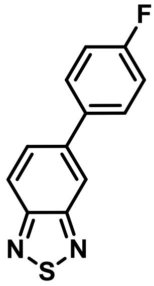

4b

Modified literature procedure.[9] Prior to utilization toluene and H_2O were purged with argon for 20 min. Boronic acid ester **3a** (105 mg, 0.40 mmol, 1.0 equiv.), $Pd(OAc)_2$ (2 mol%, 1.8 mg, 8.0 μmol,), K_3PO_4 (170 mg, 0.80 mmol, 2.0 equiv.) and XPhos (4 mol%, 7.6 mg, 16 μmol) were weighed in a microwave vial equipped with a stirrer bar, sealed and placed under argon atmosphere. Degassed toluene (2.0 mL), 1-bromo-4-fluorobenzene (53 μL, 84 mg, 0.48 mmol, 1.2 equiv.) and degassed H_2O (0.2 mL) were added to the vial. The reaction mixture was stirred for 18 h in an oil bath at 80 °C. The solution was allowed to reach rt, diluted with Et_2O (40 mL) and washed with brine (30 mL). The organic phase was dried ($MgSO_4$), filtered and the solvent was removed under reduced pressure. Purification by flash column chromatography (SiO_2, eluent: hexane/EtOAc gradient from 98:2 to 95:5) afforded the product **4b** as a pale yellow solid (85 mg, 0.37 mmol, 92%). R_f = 0.29 (hexane/EtOAc 95:5). ^{1}H NMR (400 MHz, $CDCl_3$) δ 8.11 (s, 1H), 8.05 (d, J = 9.1 Hz, 1H), 7.82 (dt, J = 9.1, 1.4 Hz, 1H), 7.66 (ddt, J = 8.5, 5.1, 1.6 Hz, 2H), 7.23 – 7.15 (m, 2H). ^{13}C{^{1}H} NMR (101 MHz, $CDCl_3$) δ 163.2 (J_{CF} = 148.4 Hz), 155.5, 154.3, 141.6, 135.8 (J_{CF} = 3.2 Hz), 130.1, 129.3 (J_{CF} = 8.4 Hz), 121.7, 118.6, 116.2 (J_{CF} = 21.8 Hz). ^{19}F NMR (376 MHz, $CDCl_3$) δ -113.7. HRMS (EI+) calcd for $[C_{12}H_7N_2SF]^+$ 230.0308, found m/z 230.0303.

5-(4-Methoxyphenyl)-2,1,3-benzothiadiazole (4c)

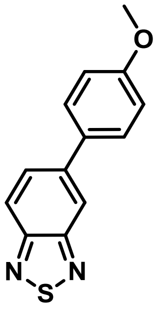

4c

Modified literature procedure.[9] Prior to use, toluene and H_2O were purged with argon for 20 min. Boronic acid ester **3a** (105 mg, 0.40 mmol, 1.0 equiv.), $Pd(OAc)_2$ (2 mol%, 1.8 mg, 8.0 μmol), K_3PO_4 (170 mg, 0.80 mmol, 2.0 equiv.) and XPhos (4 mol%, 7.6 mg, 16 μmol) were weighed in a microwave vial equipped with a stirrer bar, sealed and placed under argon atmosphere. Degassed toluene (2.0 mL), 1-bromo-4-fluorobenzene (60 μL, 90 mg, 0.48 mmol, 1.2 equiv.) and degassed H_2O (0.2 mL) were added to the vial. The reaction mixture was stirred for 18 h in an oil bath at 80 °C. The solution was allowed to reach rt, diluted with Et_2O (40 mL) and washed with brine (30 mL). The organic phase was dried ($MgSO_4$), filtered and the solvent was removed under reduced pressure. Purification of the crude product by flash column chromatography (SiO_2, eluent: hexane/EtOAc gradient from 98:2 to 95:5) yielded the product **4c** as a pale yellow solid (82 mg, 0.34 mmol, 85%). R_f = 0.23 (hexane/EtOAc 95:5). ^{1}H NMR (400 MHz, $CDCl_3$) δ 8.09 (s, 1H), 8.01 (d, J = 9.1 Hz, 1H), 7.85 (d, J = 9.2 Hz, 1H), 7.64 (d, J = 8.8 Hz, 2H), 7.03 (d, J = 8.8 Hz, 2H), 3.87 (s, 3H). ^{13}C{^{1}H} NMR (101 MHz, $CDCl_3$) δ 160.0, 155.5, 154.0, 142.0, 131.9, 130.1, 128.6, 121.3, 117.5, 114.6, 55.4. HRMS (EI+) calcd for $[C_{13}H_{10}N_2OS]^+$ 242.0508, found m/z 242.0503.

2-(2,1,3-Benzothiadiazol-5-yl)aniline (4d)

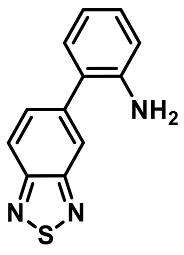

4d

Modified literature procedure.[9] Prior to use, toluene and H_2O were purged with argon for 20 min. Boronic acid ester **3a** (315 mg, 1.2 mmol, 1.0 equiv.), 2-bromoaniline (248 mg, 1.4 mmol, 1.2 equiv.), $Pd(OAc)_2$ (2 mol%, 5.4 mg, 0.024 mmol), K_3PO_4 (509 mg, 2.4 mmol, 2.0 equiv.) and XPhos (4 mol%, 22.9 mg, 0.048 mmol) were weighed in a microwave vial equipped with a stirrer bar, sealed and placed under argon atmosphere. Degassed toluene (6.0 mL) and degassed H_2O (0.6 mL) were added to the vial. The reaction mixture was stirred for 21 h in an oil bath at 80 °C. The solution was allowed to reach rt, diluted with Et_2O (40 mL) and washed with brine (30 mL). The organic phase was dried ($MgSO_4$), filtered and the solvent was removed under reduced pressure. Purification of the crude product by flash column chromatography (SiO_2, eluent: hexane/Et_2O gradient from 85:15 to 50:50) yielded the product **4d** as a yellow solid (185 mg, 0.81 mmol, 68%). ^{1}H NMR (400 MHz, $CDCl_3$) δ 8.09 (s, 1H), 8.06 (d, *J* = 9.1 Hz, 1H), 7.76 (dd, *J* = 9.0, 1.6 Hz, 1H), 7.23 (d, *J* = 7.5 Hz, 2H), 6.89 (t, *J* = 7.6 Hz, 1H), 6.82 (d, *J* = 8.1 Hz, 1H), 3.81 (br s, 2H). ^{13}C{^{1}H} NMR (101 MHz, $CDCl_3$) δ 155.4, 154.2, 143.7, 141.3, 131.9, 130.6, 129.6, 125.9, 121.7, 121.0, 119.1, 116.2. HRMS (ESI+) calcd for $[C_{12}H_9N_3S+H]^+$ 228.0590, found m/z 228.0586.

5-(2-Nitrophenyl)-2,1,3-benzothiadiazole (4e)

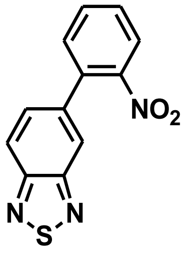

4e

Modified literature procedure.[9] Prior to use, toluene and H_2O were purged with argon for 20 min. Boronic acid ester **3a** (105 mg, 0.40 mmol, 1.0 equiv.), 1-bromo-2-nitrobenzene (97 mg, 0.48 mmol, 1.2 equiv.), $Pd(OAc)_2$ (2 mol%, 1.8 mg, 8.0 μmol), K_3PO_4 (170 mg, 0.80 mmol, 2.0 equiv.) and XPhos (4 mol%, 7.6 mg, 16 μmol) were weighed in a microwave vial equipped with a stirrer bar, sealed and placed under argon atmosphere. Degassed toluene (2.0 mL) and degassed H_2O (0.2 mL) were added to the vial. The reaction mixture was stirred for 18 h in an oil bath at 80 °C. The solution was allowed to reach rt, diluted with Et_2O (40 mL) and washed with brine (30 mL). The organic phase was dried ($MgSO_4$), filtered and the solvent was removed under reduced pressure. Purification of the crude product by flash column chromatography (SiO_2, eluent: hexane/EtOAc gradient from 97:3 to 90:10) yielded the product **4e** as a beige solid (93 mg, 0.36 mmol, 90%). R_f = 0.37 (hexane/EtOAc 90:10). ^{1}H NMR (400 MHz, $CDCl_3$) δ 8.04 (dd, *J* = 8.2, 1.1 Hz, 1H), 8.03 (d, *J* = 9.0 Hz, 1H), 7.97 (dd, *J* = 1.7, 0.8 Hz, 1H), 7.72 (td, *J* = 7.5, 1.3 Hz, 1H), 7.60 (td, *J* = 7.8, 1.5 Hz, 1H), 7.54 (dd, *J* = 7.6, 1.5 Hz, 1H), 7.50 (dd, *J* = 9.1, 1.7 Hz, 1H). ^{13}C{^{1}H} NMR (101 MHz, $CDCl_3$) δ: 154.9, 154.4, 148.8, 139.7, 135.5, 133.2, 132.2, 130.4, 129.4, 124.9, 121.6, 120.3. HRMS (EI+) calcd for $[C_{12}H_7N_3O_2S]^+$ 257.0253, found m/z 257.0250.

5-(Thiophen-2-yl)-2,1,3-benzothiadiazole (4f)

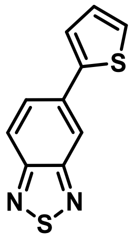

4f

Modified literature procedure.[9] Boronic acid ester **3a** (105 mg, 0.40 mmol, 1.0 equiv.), $Pd(OAc)_2$ (2 mol%, 1.8 mg, 8.0 μmol), K_3PO_4 (170 mg, 0.80 mmol, 2.0 equiv.) and XPhos (4 mol%, 7.6 mg, 16 μmol) were weighed in a microwave vial equipped with a stirrer bar, sealed and placed under argon atmosphere. Degassed toluene (2.0 mL), 2-bromothiophene (47 μL, 78 mg, 0.48 mmol, 1.2 equiv.) and degassed H_2O (0.2 mL) were added to the vial. The reaction mixture was placed in a preheated oil bath at 80 °C and stirred for 18 h. The solution was allowed to reach rt, diluted with Et_2O (40 mL) and washed with brine (30 mL). The organic phase was dried ($MgSO_4$), filtered and the solvent was removed under reduced pressure. The crude product was purified by flash column chromatography (SiO_2, eluent: hexane/EtOAc gradient from 98:2 to 95:5) to afford the product **4f** as a yellow solid (64 mg, 0.29 mmol, 73%). R_f = 0.43 (hexane/EtOAc 95:5). ^{1}H NMR (400 MHz, $CDCl_3$) δ 8.18 (d, *J* = 1.8, 1H), 7.98 (d, *J* = 9.2 Hz, 1H), 7.89 (dd, *J* = 9.2, 1.8 Hz, 1H), 7.49 (d, *J* = 3.7 Hz, 1H), 7.40 (d, *J* = 5.1 Hz, 1H), 7.14 (dd, *J* = 5.0, 3.7 Hz, 1H). $^{13}C\{^1H\}$ NMR (101 MHz, $CDCl_3$) δ 155.4, 154.3, 142.7, 135.7, 129.1, 128.6, 126.8, 125.2, 121.8, 116.6. Spectral data are in accordance with previously reported values.[14]

5-(Pyrimidin-2-yl)-2,1,3-benzothiadiazole (4g)

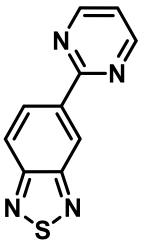

4g

Modified literature procedure.[15] Boronic acid ester **3a** (250 mg, 0.95 mmol, 1.0 equiv.), 2-bromopyrimidine (182 mg, 1.1 mmol, 1.2 equiv.), $PdCl_2(dppf)$ (5 mol%, 35 mg, 0.048 mmol) and K_3PO_4 (607 mg, 2.9 mmol, 3.0 equiv.) were weighed in a microwave vial equipped with a stirrer bar, sealed and placed under argon atmosphere. Degassed toluene (1.6 mL), degassed EtOH (1.6 mL) and degassed H_2O (1.6 mL) were added and the solution was stirred at 85 °C for 24 h. The reaction mixture was allowed to reach rt, diluted with H_2O and extracted with EtOAc (3 x 80 mL). The combined organic phases were washed with brine (80 mL) and dried (Na_2SO_4) and filtered. The solvent was removed under reduced pressure and the residue was purified by flash chromatography (SiO_2, eluent: hexane/EtOAc gradient from 95:5 to 80:20). The title compound **4g** was obtained as a colorless solid (162 mg, 0.76 mmol, 79%). R_f = 0.51 (hexane/EtOAc 80:20). ^{1}H NMR (400 MHz, $CDCl_3$) δ 9.13 (dd, *J* = 1.6, 0.8 Hz, 1H), 8.88 (d, *J* = 4.8 Hz, 2H), 8.73 (dd, *J* = 9.3, 1.6 Hz, 1H), 8.08 (dd, *J* = 9.3, 0.8 Hz, 1H), 7.28 (t, *J* = 4.9 Hz, 1H). $^{13}C\{^1H\}$ NMR (101 MHz, $CDCl_3$) δ 163.7, 157.6, 156.1, 155.4, 138.96, 129.3, 121.8, 121.4, 112.0. HRMS (EI+) calcd for $[C_{10}H_6N_4S]^+$ 214.0308, found m/z 214.0302.

5-(Pyrimidin-5-yl)-2,1,3-benzothiadiazole (4h)

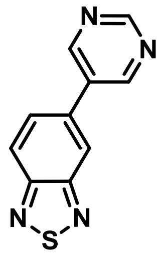

4h

Modified literature procedure.[15] Boronic acid ester **3a** (100 mg, 0.38 mmol, 1.0 equiv.), 5-bromopyrimidine (73 mg, 0.46 mmol, 1.2 equiv.), $PdCl_2$(dppf) (5 mol%, 14.0 mg, 19 μmol) and K_3PO_4 (243 mg, 1.1 mmol, 3.0 equiv.) were weighed in a microwave vial equipped with a stirrer bar, sealed and placed under an argon atmosphere. Degassed toluene (0.6 mL), degassed EtOH (0.6 mL) and degassed H_2O (0.6 mL) were added and the solution was stirred at 85 °C for 16 h. The reaction mixture was allowed to reach rt, diluted with H_2O (50 mL) and extracted with EtOAc (3 x 50 mL). The combined organic phases were washed with brine (50 mL), dried (Na_2SO_4) and filtered. The filtrate was concentrated under reduced pressure and the residue was purified by flash chromatography (SiO_2, eluent: petroleum ether/EtOAc gradient from 80:20 to 60:40). The title compound **4h** was obtained as a colorless solid (60 mg, 0.28 mmol, 73%). R_f = 0.21 (hexane/EtOAc 80:20). ^{1}H NMR (400 MHz, $CDCl_3$) δ 9.30 (s, 1H), 9.09 (s, 2H), 8.24 (dd, *J* = 1.8, 0.9 Hz, 1H), 8.18 (dd, *J* = 9.1, 0.8 Hz, 1H), 7.84 (dd, *J* = 9.1, 1.8 Hz, 1H). $^{13}C\{^1H\}$ NMR (101 MHz, $CDCl_3$) δ 158.5, 155.4, 155.1, 154.7, 135.7, 133.3, 128.7, 123.0, 119.8. HRMS (EI+) calcd for $[C_{10}H_6N_4S]^+$ 214.0308, found m/z 214.0302.

5-Chloro-2,1,3-benzothiadiazole (5a)

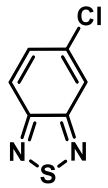

5a

Modified literature procedure.[16] A solution of boronic acid ester **3a** (52 mg, 0.20 mmol, 1.0 equiv.) in MeOH (2.5 mL) was combined with a solution of $CuCl_2·2H_2O$ (102 mg, 0.60 mmol, 3.0 equiv.) in H_2O (2.5 mL) in a microwave vial equipped with a stirrer bar and sealed. The reaction solution was stirred at 80 °C for 48 h. The solution was allowed to reach rt, was extracted with Et_2O (3 x 20 mL) and the combined organic phases were dried ($MgSO_4$) and filtered. The crude product was dry loaded on Celite. After purification by flash column chromatography (SiO_2, eluent: gradient from 100% hexane to 5% EtOAc in hexane) the title compound **5a** was obtained as a colorless solid (19 mg, 0.11 mmol, 56%). ^{1}H NMR (400 MHz, $CDCl_3$) δ 8.03 (dd, *J* = 2.0, 0.7 Hz, 1H), 7.94 (dd, *J* = 9.3, 0.7 Hz, 1H), 7.56 (dd, *J* = 9.3, 2.0 Hz, 1H). $^{13}C\{^1H\}$ NMR (101 MHz, $CDCl_3$) δ: 155.0, 153.4, 136.5, 131.1, 122.2, 120.5. HRMS (EI+) calcd for $[C_6H_3N_2SCl]^+$ 169.9700, found m/z 169.9697.

5-Bromo-2,1,3-benzothiadiazole (5b)

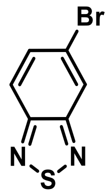

5b

Modified literature procedure.[16] Boronic acid ester **3a** (52 mg, 0.20 mmol, 1.0 equiv.) was dissolved in MeOH (2.5 mL) and combined with a solution of $CuBr_2$ (134 mg, 0.60 mmol, 3.0 equiv.) in H_2O (2.5 mL) in a microwave vial equipped with a stirrer bar and sealed. The solution was stirred at 80 °C for 48 h. The solution was allowed to reach rt, Et_2O (20 mL) was added and the orange precipitate was filtered. The filtrate was extracted with Et_2O (3 x 20 mL) and the combined organic phases were dried ($MgSO_4$). After filtratation, the

solvent was removed under reduced pressure and the residue purified by flash column chromatography (SiO_2, eluent: gradient from 100% hexane to 5% EtOAc in hexane). The title compound **5b** was obtained as a colorless solid (28 mg, 0.13 mmol, 65%). ^{1}H NMR (400 MHz, $CDCl_3$) δ 8.23 (dt, *J* = 1.9, 0.9 Hz, 1H), 7.88 (dt, *J* = 9.2, 0.8 Hz, 1H), 7.68 (ddd, *J* = 9.2, 1.9, 0.9 Hz, 1H). ^{13}C{^{1}H} NMR (101 MHz, $CDCl_3$) δ 155.5, 153.5, 133.4, 124.7, 124.0, 122.4. Spectral data are in accordance with previously reported values.[17]

5-Iodo-2,1,3-benzothiadiazole (5c)

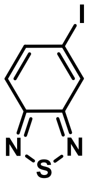

5c

Modified literature procedure.[18] In a microwave vial equipped with a stirrer bar boronic acid ester **3a** (52 mg, 0.20 mmol, 1.0 equiv.), CuI (10 mol%, 3.8 mg, 0.020 mmol), phenanthroline (20 mol%, 7.2 mg, 0.040 mmol) and KI (40 mg, 0.24 mmol, 1.2 equiv.) were dissolved in MeOH (1.6 mL) and stirred at rt before H_2O was added (0.4 mL) and the vial sealed. The reaction solution was stirred at 80 °C for 24 h and after reaching rt, H_2O (10 mL) was added. The solution was extracted with Et_2O (4 x 20 mL) and the combined organic phases were washed with brine (30 mL), dried ($MgSO_4$), filtered and the solvent was removed under reduced pressure. Purification by flash column chromatography (SiO_2, eluent: gradient from 100% hexane to 5% EtOAc in hexane) afforded the title compound **5c** as an off-white solid (37 mg, 0.14 mmol, 71%). ^{1}H NMR (400 MHz, $CDCl_3$) δ 8.48 (dd, *J* = 1.6, 0.7 Hz, 1H), 7.81 (dd, *J* = 9.2, 1.6 Hz, 1H), 7.74 (dd, *J* = 9.1, 0.7 Hz, 1H). ^{1}H NMR (400 MHz, DMSO-d_6) δ 8.63 (dd, *J* = 1.6, 0.7 Hz, 1H), 7.95 (dd, *J* = 9.1, 1.6 Hz, 1H), 7.90 (dd, *J* = 9.2, 0.7 Hz, 1H). ^{13}C{^{1}H} NMR (101 MHz, $CDCl_3$) δ 156.0, 153.8, 138.3, 130.8, 122.5, 96.4. HRMS (EI+) calcd for $[C_6H_3N_2SI]^+$ 261.9056, found m/z 261.9053.

5-(Mesityl(tosyl)-λ^3-iodaneyl)-2,1,3-benzothiadiazole (6)

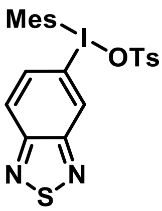

6

To a flame-dried 10 mL microwave vial equipped with a stirrer bar under argon were added trifluoroborate **3f** (48 mg, 0.20 mmol, 1.0 equiv.), 2-(diacetoxyiodo)mesitylene (87 mg, 0.24 mmol, 1.2 equiv.), the vial was sealed and the atmosphere was exchanged again for argon. Dry MeCN (2.5 mL) was added. While the solution was stirring at rt, $BF_3{\cdot}Et_2O$ (74 µL, 0.60 mmol, 3.0 equiv.) was added dropwise. The solution was stirred at rt for 2 h and concentrated under reduced pressure, then redissolved in CH_2Cl_2 (10 mL) and aqueous sodium *p*-toluenesulfonate (2 M, 10 mL). The biphasic mixture was stirred for 10 min, the phases were separated and the aqueous phase was extracted with CH_2Cl_2 (3 x 10 mL). The combined organic phases were dried (Na_2SO_4), filtered and concentrated under reduced pressure. The solid crude product was triturated with Et_2O to afford title compound **6** as a yellow solid (51 mg, 0.93 mmol, 46%). ^{1}H NMR (400 MHz, $CDCl_3$) δ 8.28 (s, 1H), 8.05 (d, *J* = 9.4 Hz, 1H), 7.93 (d, *J* = 9.3 Hz, 1H), 7.43 (d, *J* = 7.9 Hz, 2H), 7.02 (s, 2H), 6.95 (d, *J* = 7.8 Hz, 2H), 2.65 (s, 6H), 2.32 (s, 3H), 2.28 (s, 3H). ^{13}C{^{1}H} NMR (101 MHz, $CDCl_3$) δ 154.9, 154.5, 143.7, 142.2, 139.5, 132.0, 130.0, 128.4,

127.2, 125.9, 123.9, 27.2, 21.3, 21.1. HRMS (TOF MS ES+) calcd for $[C_{22}H_{21}N_2O_3S_2I+Cs]^+$ 684.9093, found m/z 684.9088 (doped with CsI).

2,1,3-Benzothiadiazol-4-yl(mesityl)-λ^3-iodaneyl 4-methylbenzenesulfonate (6')

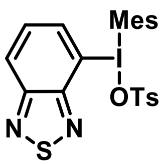

6'

To a flame-dried 50 mL round-bottom flask equipped with a stirrer bar under argon was added the trifluoroborate **3f'** (201 mg, 0.83 mmol, 1.0 equiv.), 2-(diacetoxyiodo)mesitylene (362 mg, 0.99 mmol, 1.2 equiv.) and MeCN (10 mL, 0.08 M). $BF_3 \cdot Et_2O$ (310 µL, 357 mg, 2.51 mmol, 3.0 equiv.) was slowly added dropwise. The solution was stirred at rt for 4 h. The solution was concentrated under reduced pressure, and the residue was redissolved in aqueous sodium *p*-toluenesulfonate solution (2 M, 8.2 mL, 16 mmol, 20 equiv.) and CH_2Cl_2 (10 mL). The biphasic system was stirred for 10 min. The phases were separated, the aqueous phase was extracted with CH_2Cl_2 (3 x 10 mL), the combined organic phases were dried (Na_2SO_4), filtered, and concentrated under reduced pressure. The crude product was purified by trituration with Et_2O followed by recrystallisation from $CHCl_3$/*n*-pentane to afford **6'** as a brown solid (306 mg, 0.55 mmol, 67%). ^1H NMR (400 MHz, $CDCl_3$) δ 8.11 (d, *J* = 8.0 Hz, 2H), 7.55 (t, *J* = 7.7 Hz, 1H), 7.44 (d, *J* = 7.7 Hz, 2H), 7.00 – 6.95 (m, 4H), 2.75 (s, 6H), 2.30 (s, 3H), 2.28 (s, 3H). $^{13}C\{^1H\}$ NMR (101 MHz, $CDCl_3$) δ 153.6, 152.9, 143.7, 142.9, 142.5, 139.5, 137.6, 130.5, 130.1, 128.5, 126.0, 125.4, 122.6, 104.3, 27.3, 21.4, 21.2. HRMS (TOF MS ES+) calcd for $[C_{22}H_{21}N_2O_3S_2I+Cs]^+$ 684.9093, found m/z 684.9088 (doped with CsI).

2,1,3-Benzothiadiazol-5-ol (7)

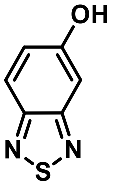

7

A microwave vial equipped with a stirrer bar was loaded with boronic acid ester **3a** (65.5 mg, 0.25 mmol, 1.0 equiv.). Acetone (1.25 mL) and H_2O (1.25 mL) was added to the sealed vial and the suspension was stirred. OXONE® (115 mg, 0.38 mmol, 1.5 equiv.) was added in one portion. The solution was stirred at rt for 1 h after which the solution was diluted with H_2O (10 mL) and extracted with EtOAc (3 x 10 mL). The combined organic phases were dried ($MgSO_4$), filtered and the crude reaction mixture was dry loaded onto SiO_2. The crude reaction mixture was purified by flash column chromatography (SiO_2, eluent: CH_2Cl_2:MeOH 99:1). The title compound **7** was collected as a yellow solid (36 mg, 0.24 mmol, 95%). R_f = 0.17 (CH_2Cl_2:MeOH 99:1) ^1H NMR (400 MHz, acetone-d_6) δ 9.40 (s, 1H), 7.84 (d, *J* = 9.4 Hz, 1H), 7.36 (dd, *J* = 9.3, 2.4 Hz, 1H), 7.21 (d, *J* = 2.4 Hz, 1H). $^{13}C\{^1H\}$ NMR (101 MHz, acetone-d_6) δ 159.8, 157.0, 151.5, 125.5, 122.4, 101.6. Spectral data are in accordance with previously reported values.[19]

5,5'-Bi-2,1,3-benzothiadiazole (8)

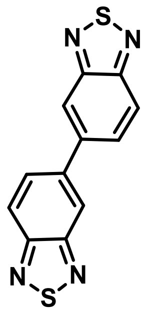

8

Modified literature procedure.[20,21] Boronic acid ester **3a** (79 mg, 0.30 mmol, 1.0 equiv.), $PdCl_2(PPh_3)_2$ (20 mol%, 42 mg, 0.060 mmol) and boric acid (85 mg, 1.4 mmol, 4.6 equiv.) were dissolved in THF (3 mL) in a sealed microwave vial equipped with a stirrer bar and purged with compressed air for 10 min. Subsequently, KF (65 mg, 1.1 mmol, 3.7 equiv.) and H_2O (0.75 mL) were added and the reaction mixture was stirred at rt under air for 23 h. CH_2Cl_2 (50 mL) and H_2O (20 mL) were added, the phases were separated and the aqueous phase extracted with CH_2Cl_2 (3 x 30 mL). The combined organic phases were washed with H_2O (50 mL) and brine (50 mL), dried ($MgSO_4$), filtered and the solvent was removed under reduced pressure. The crude product was purified by flash column chromatography (SiO_2, eluent: gradient from pure CH_2Cl_2 to CH_2Cl_2/MeOH 98:2) to afford the title compound **8** as a colorless solid (21 mg, 0.078 mmol, 52%). R_f = 0.88 (CH_2Cl_2). ^{1}H NMR (400 MHz, DMSO-d_6) δ 8.63 (dd, *J* = 1.8, 0.9 Hz, 2H), 8.31 (dd, *J* = 9.2, 1.8 Hz, 2H), 8.25 (dd, *J* = 9.2, 0.8 Hz, 2H). ^{13}C NMR spectrum could not be measured due to the poor solubility of **8**. Spectral data are in accordance with previously reported values.[22]

2,1,3-Benzothiadiazole-5-carbaldehyde (9)

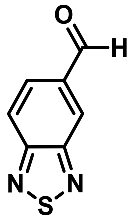

9

Modified literature procedure.[23] Boronic acid **3e** (36 mg, 0.20 mmol, 1.0 equiv.) and glyoxylic acid monohydrate (22 mg, 0.24 mmol, 1.2 equiv.) were weighed in an oven-dried microwave vial equipped with a stirrer bar, sealed, dissolved in anhydrous MeCN (2 mL) and the solution was purged with oxygen. Indoline (7 μL, 7 mg, 0.06 mmol, 0.3 equiv.) was added and the reaction mixture was heated at 70 °C for 28 h. The reaction mixture was concentrated under reduced pressure and the residue was purified by flash column chromatography (SiO_2, eluent: hexane/EtOAc from 20:1 to 9:1) affording the title compound **9** as a colorless solid (18 mg, 0.11 mmol, 55%). ^{1}H NMR (400 MHz, $CDCl_3$) δ 10.22 (s, 1H), 8.51 (s, 1H), 8.12 (d, *J* = 1.2 Hz, 2H). ^{13}C{^{1}H} NMR (101 MHz, $CDCl_3$) δ 191.4, 157.4, 154.6, 137.2, 127.9, 126.1, 122.8. Spectral data are in accordance with previously reported values.[24]

***N*-Phenyl-2,1,3-benzothiadiazol-5-amine (10a)**

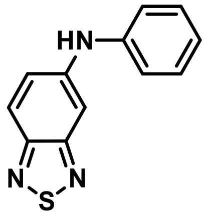

10a

Modified literature procedure.[25] Boronic acid **3e** (30 mg, 0.17 mmol, 1.0 equiv.) and *anti*-1,2,2,3,4,4-Hexamethylphosphetane 1-oxide (4.0 mg, 0.023 mmol, 0.15 equiv.) were weighed in an oven-dried microwave vial equipped with a stirrer bar, sealed, placed under argon atmosphere and dissolved in xylene (0.3 mL) which was previously passed over activated basic alumina and purged with argon. Nitrobenzene (16 μL, 19 mg, 0.15 mmol, 1.0 equiv.) and phenylsilane (37 μL, 33 mg, 0.30 mmol, 2.0 equiv.) were added and the mixture was heated to 120 °C

for 24 h. The solution was allowed to reach rt, H_2O (10 mL) and EtOAc (10 mL) were added. The organic phase was washed with aqueous NaOH (1 M, 20 mL) and brine (30 mL) and dried ($MgSO_4$). After filtration the solvent was removed under reduced pressure. The product **10a** was obtained after purification by column chromatography (eluent: petroleum ether/EtOAc 7:1 with 0.5% triethylamine) as a yellow solid (30 mg, 0.13 mmol, 87%). ^{1}H NMR (400 MHz, $CDCl_3$) δ 7.84 (d, *J* = 9.3 Hz, 1H), 7.44 (s, 1H), 7.38 (t, *J* = 7.1 Hz, 2H), 7.28 (d, *J* = 9.2 Hz, 1H), 7.24 (s, 2H), 7.11 (t, *J* = 7.4 Hz, 1H), 6.05 (s, 1H). ^{13}C{^{1}H} NMR (101 MHz, $CDCl_3$) δ 156.6, 151.2, 145.3, 140.9, 129.8, 125.57, 123.7, 122.0, 120.87, 99.9. HRMS (ESI+) calcd for $[C_{12}H_9N_3S + H]^+$ 228.0590, found m/z 228.0585.

N-(2-bromophenyl)-2,1,3-benzothiadiazol-5-amine (10b)

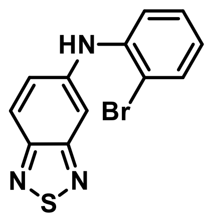

10b

Modified literature procedure.[25] Boronic acid **3e** (50 mg, 0.28 mmol, 1.1 equiv.), 2-nitro-1-bromobenzene (51 mg, 0.25 mmol, 1.0 equiv.) and *trans*-hexamethyl-phosphetane oxide (6.6 mg, 0.038 mmol, 0.15 equiv.) were weighed in an oven-dried microwave vial equipped with a stirrer bar, sealed, placed under argon atmosphere and dissolved in xylene (0.5 mL) which was previously passed over activated basic alumina and purged with argon. Phenylsilane (62 μL, 55 mg, 0.51 mmol, 2.0 equiv.) were added and the mixture was heated to 120 °C for 24 h. After reaching rt, H_2O (10 mL) and EtOAc (10 mL) were added. The organic phase was washed with aqueous NaOH (1 M, 20 mL) as well as brine (30 mL) and dried ($MgSO_4$). After filtration the solvent was removed under reduced pressure. The product **10b** was obtained after purification by column chromatography (eluent: petroleum ether/EtOAc 11:1 with 2% triethylamine) as a yellow solid (58 mg, 0.19 mmol, 75%). ^{1}H NMR (400 MHz, $CDCl_3$) δ 7.88 (d, *J* = 9.3 Hz, 1H), 7.61 (d, *J* = 7.9 Hz, 1H), 7.54 – 7.46 (m, 2H), 7.36 (d, *J* = 9.3 Hz, 1H), 7.29 (t, *J* = 7.9 Hz, 1H), 6.93 (t, *J* = 7.7 Hz, 1H), 6.31 (s, 1H). ^{13}C{^{1}H} NMR (101 MHz, $CDCl_3$) δ 156.3, 151.6, 143.9, 139.2, 133.5, 128.5, 126.0, 123.9, 122.1, 119.9, 115.1, 102.4. HRMS (ESI+) calcd for $[C_{12}H_8N_3SBr + H]^+$ 305.9695, found m/z 305.9690.

Alternative Buchwald-Hartwig amination procedure for 10b

Modified literature procedure.[26] In a microwave vial equipped with a stirrer bar 5-bromo-2,1,3-benzothiadiazole (108 mg, 0.50 mmol, 1.0 equiv.) and 2-bromoaniline (96 mg, 0.56 mmol, 1.1 equiv.) were added to a suspension of *tert*-BuOK (69 mg, 0.62 mmol, 1.2 equiv.) and PEPPSI-IPr (2 mol%, 7 mg, 0.01 mmol) in dry toluene (2.5 mL). The reaction mixture was stirred under argon atmosphere at 120 °C for 18 h. The mixture was allowed to reach rt, filtrated through Celite and the solvent was removed under reduced pressure. The crude product was washed with pentane to obtain title compound **10b** as a brown solid (70 mg, 0.23 mmol, 46%).

3) Fused BTD motifs

6*H*-[1,2,5]thiadiazolo[3,4-*c*]carbazole (11a)

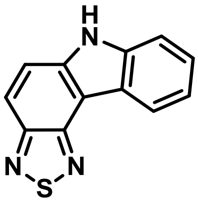

11a

Modified literature procedure.[28] Compound **10b** (40 mg, 0.13 mmol, 1.0 equiv.), $Pd(OAc)_2$ (3 mol%, 0.9 mg, 4 μmol), tricyclohexylphosphine tetrafluoroborate (6 mol%, 2.9 mg, 7.8 μmol) and K_2CO_3 (36 mg, 0.26 mmol, 2.0 equiv.) were weighed in an oven-dried microwave vial equipped with a stirrer bar, sealed and placed under argon atmosphere. Dimethylacetamide (0.65 mL) which was previously passed over activated basic alumina and purged with argon was added to the vial and the mixture was heated to 130 °C for 42 h. Additional $Pd(OAc)_2$ (3 mol%, 0.9 mg, 4 μmol) and tricyclohexylphosphine tetrafluoroborate (6 mol%, 2.9 mg, 7.8 μmol) were added to the reaction mixture and stirred at 140 °C for another 22 h. After reaching rt, the solution was dry loaded onto Celite and the crude product was purified via column chromatography (eluent: hexane/EtOAc from 90:10 to 75:25). The product **11a** was obtained as a yellow solid (21 mg, 0.093 mmol, 71%). R_f = 0.17 (hexane/EtOAc 90:10). ^{1}H NMR (400 MHz, acetone-d_6) δ 11.19 (s, 1H), 8.53 (d, *J* = 7.8 Hz, 1H), 8.03 (d, *J* = 9.3 Hz, 1H), 7.94 (d, *J* = 9.4 Hz, 1H), 7.71 (d, *J* = 8.2 Hz, 1H), 7.47 (t, *J* = 8.2 Hz, 1H), 7.40 (t, *J* = 7.4 Hz, 1H). ^{13}C{^{1}H} NMR (101 MHz, acetone-d_6) δ 153.3, 151.8, 139.7, 139.5, 125.8, 123.9, 122.3, 121.8, 120.1, 119.2, 112.9, 111.1. HRMS (ESI+) calcd for $[C_{12}H_7N_3S + H]^+$ 226.0433, found m/z 226.0430.

10*H*-[1,2,5]thiadiazolo[3,4-*a*]carbazole (11b)

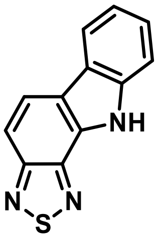

11b

Modified literature procedure.[29] Compound **4e** (34 mg, 0.13 mmol, 1.0 equiv.) was dissolved in a sealed microwave vial equipped with a stirrer bar under argon atmosphere in triethyl phosphate (0.2 mL) that was briefly purged with argon. The mixture was heated to 160 °C for 16 h after which the solution was allowed to reach rt and the product precipitated as a yellow solid. EtOAc (20 mL) and H_2O (20 mL) were added and the organic phase was washed with H_2O (3 x 20 mL) and brine (20 mL), dried ($MgSO_4$), filtered and the solvent removed under reduced pressure. Purification via column chromatography (SiO_2, eluent: petroleum ether/EtOAc 9:1) afforded product **11b** as a yellow solid (24 mg, 0.11 mmol, 81%). R_f = 0.13 (pentane/EtOAc 20:1). ^{1}H NMR (400 MHz, acetone-d_6) δ 11.78 (s, 1H), 8.44 (d, *J* = 9.1 Hz, 1H), 8.23 (d, *J* = 8.0 Hz, 1H), 7.74 (dd, *J* = 13.3, 8.6 Hz, 2H), 7.53 – 7.44 (m, 1H), 7.34 (t, *J* = 7.5 Hz, 1H). ^{13}C{^{1}H} NMR (10 MHz, acetone-d_6) δ: 156.3, 145.8, 140.2, 126.4, 125.4, 124.3, 121.5, 121.0, 120.2, 113.1, 113.1, 113.0. Spectral data are in accordance with previously reported values.[30]

Alternative oxidative Ir-catalyzed C–H amination for 11b

Modified literature procedure.[31] Compound **4d** (20 mg, 0.088 mmol, 1.0 equiv.), $[IrCp^*Cl_2]_2$ (4 mol%, 2.8 mg, 3.5 μmol), anhydrous $Cu(OAc)_2$ (3.2 mg, 0.017 mmol, 0.2 equiv.) and pivalic acid (18.0 mg, 0.18 mmol, 2.0 equiv.) were weighed in an oven-dried microwave vial equipped with a stirrer bar, sealed and NMP (1.1 mL) was added. The reaction mixture was stirred at 120 °C under air for 23 h. After reaching rt, H_2O (40 mL) and EtOAc (50 mL) were added. The organic phase was washed with H_2O (4 x 40 mL) and aq. $NaHCO_3$ solution (40 mL). The organic solution was dried (Na_2SO_4), filtered and the solvent was removed under reduced pressure. The crude product was purified by flash column chromatography (SiO_2, eluent: hexane/EtOAc gradient from 97:3 to 9:1) to afford **11b** as a yellow solid (5 mg, 22.2 μmol, 25%). *Characterization data see above.*

4,6-Bis(2-nitrophenyl)-2,1,3-benzothiadiazole (4i)

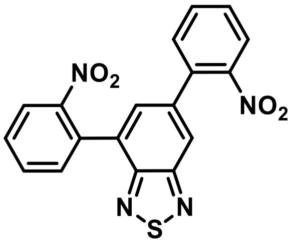

4i

Modified literature procedure.[9] Diboronic acid ester **3c** (100 mg, 0.26 mmol, 1.0 equiv.), 1-bromo-2-nitrobenzene (125 mg, 0.62 mmol, 2.4 equiv.), $Pd(OAc)_2$ (4 mol%, 2.3 mg, 10 μmol), K_3PO_4 (164 mg, 0.77 mmol, 3.0 equiv.) and XPhos (8 mol%, 10 mg, 0.02 mmol) were weighed in a microwave vial equipped with a stirrer bar, sealed and placed under argon atmosphere. Degassed toluene (1.3 mL) and degassed H_2O (0.13 mL) were added to the vial. The reaction mixture was stirred for 21 h in an oil bath at 80 °C. After reaching rt, the solution was diluted with Et_2O (40 mL) and washed with brine (30 mL). The organic phase was dried ($MgSO_4$), filtered and the solvent was removed under reduced pressure. Purification of the crude product by flash column chromatography (SiO_2, eluent: hexane/EtOAc gradient from 95:5 to 80:20) yielded the product **4i** as a brown solid (81 mg, 0.21 mmol, 83%). R_f = 0.40 (hexane/EtOAc 80:20). ^1H NMR (400 MHz, $CDCl_3$) δ 8.17 (d, J = 8.1 Hz, 1H), 8.09 (d, J = 8.1 Hz, 1H), 8.02 (d, J = 1.6 Hz, 1H), 7.75 (t, J = 7.7 Hz, 2H), 7.67-7.57 (m, 4H), 7.52 (d, J = 1.6 Hz, 1H). $^{13}C\{^1H\}$ NMR (101 MHz, $CDCl_3$) δ 154.7, 152.9, 149.3, 148.7, 139.9, 135.2, 133.6, 133.4, 133.0, 132.4, 132.2, 131.8, 129.8, 129.6, 129.0, 125.0, 125.0, 120.3. HRMS (ESI+) calcd for $[C_{18}H_{10}N_4O_4S + H]^+$ 379.0496, found m/z 379.0486; calc for $[C_{18}H_{10}N_4O_4S + Na]^+$ 401.0315, found m/z 401.0306.

8,13-Dihydroindolo[3,2-*a*][1,2,5]thiadiazolo[3,4-*c*]carbazole (11c)

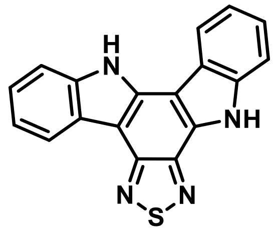

11c

Modified literature procedure.[29] Compound **4i** (40 mg, 0.11 mmol, 1.0 equiv.) was placed in a microwave vial equipped with a stirrer bar and sealed under argon atmosphere. Subsequently, triethyl phosphate (0.4 mL) that was purged with argon was added and the mixture was heated to 160 °C for 23 h. After reaching rt, EtOAc (40 mL) and H_2O (40 mL) were added and the organic phase was washed with H_2O (3 x 40 mL) and brine (40 mL) and dried (Na_2SO_4). The solution was filtered and the solvent was removed under reduced pressure and the residue was purified by flash column chromatography (SiO_2, eluent: petroleum ether/EtOAc gradient from 92:8 to 85:15). The title compound **11c** was obtained as a yellow solid (26 mg, 0.083 mmol, 63%). R_f = 0.21 (hexane/EtOAc 90:10). ^{1}H NMR (400 MHz, DMSO-d$_6$) δ 12.98 (s, 1H), 12.33 (s, 1H), 8.78 (d, *J* = 7.9 Hz, 1H), 8.48 – 8.36 (m, 1H), 7.82 – 7.68 (m, 2H), 7.51 (ddd, *J* = 8.2, 7.0, 1.2 Hz, 1H), 7.47 – 7.30 (m, 3H). ^{13}C{^{1}H} NMR (101 MHz, DMSO-d$_6$) δ 150.8, 142.9, 138.6, 138.1, 135.5, 128.2, 125.1, 123.5, 123.0, 121.4, 121.2, 120.9, 120.6, 119.7, 112.5, 112.1, 108.3, 103.4. HRMS (TOF MS ES+ Nanospray) calcd for $[C_{18}H_{11}N_4S+H]^+$ 315.0704, found m/z 315.0705.

4) Directed C–H functionalizations

1-(4-(5-(Pyrimidin-2-yl)-2,1,3-benzothiadiazol-4-yl)phenyl)ethan-1-one (12a) and 1-(4-(6-(pyrimidin-2-yl)-2,1,3-benzothiadiazol-5-yl)phenyl)ethan-1-one (12a’)

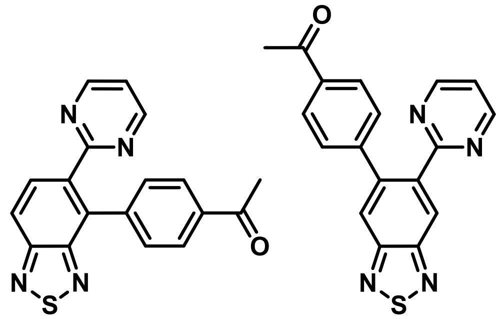

12a **12a'**

Modified literature procedure.[32] Compound **4g** (54 mg, 0.25 mmol, 1.0 equiv.), 4-bromoacetophenone (25 mg, 0.13 mmol, 0.5 equiv.), $[RuCl_2(p\text{-cymene})]_2$ (3 mol%, 3.8 mg, 6.0 μmol), 1-phenyl-1-cyclopentane-carboxylic acid (4.8 mg, 0.025 mmol, 0.1 equiv.) and K_2CO_3 (73 mg, 0.53 mmol, 2.1 equiv.) were weighed in an oven-dried microwave vial equipped with a stirrer bar, sealed and placed under argon atmosphere. Anhydrous dioxane (1 mL) was added and the solution was stirred at 150 °C for 16 h. After that time another portion of 4-bromoacetophenone (25 mg, 0.13 mmol, 0.5 equiv.), $[RuCl_2(p\text{-cymene})]_2$ (2 mol%, 2.6 mg, 4.0 μmol), 1-phenyl-1-cyclopentane-carboxylic acid (7 mol%, 3.2 mg, 0.017 mmol) and K_2CO_3 (35 mg, 0.25 mmol, 1.0 equiv.) was added as a solution in dioxane (1 mL) and the reaction mixture was stirred at 150 °C for another 25 h. Subsequently, the reaction mixture was allowed to reach rt, CH_2Cl_2 (5 mL) was added and the salts were filtered off through a layer of Celite. The solvent was removed under reduced pressure and the residue was purified by flash column chromatography (SiO_2, eluent: hexane/EtOAc gradient from 55:45 to 50:50). The title compound **12a** was obtained as an off-white solid (53 mg, 0.16 mmol, 64%, **12a/12a’** = ~15:1 based on the ^{1}H NMR spectrum). R_f = 0.11 (hexane/EtOAc 50:50). **Major isomer** (**12a**): ^{1}H NMR (400 MHz, $CDCl_3$)

δ_{major} 8.67 (d, J = 4.9 Hz, 2H), 8.17 (d, J = 9.1 Hz, 1H), 8.14 (d, J = 9.1 Hz, 1H), 7.94 (d, J = 8.3 Hz, 2H), 7.45 (d, J = 8.3 Hz, 2H), 7.17 (t, J = 4.9 Hz, 1H), 2.63 (s, 3H). $^{13}C\{^1H\}$ NMR (101 MHz, $CDCl_3$) δ_{major} 198.0, 166.5, 157.1, 155.2, 154.7, 141.9, 138.1, 136.2, 133.4, 132.1, 130.9, 128.1, 121.1, 119.3, 26.8. **Minor isomer** (**12a'**, tentative assignment): ^1H NMR (400 MHz, $CDCl_3$) δ_{minor} 8.51 (s, 1H), 8.10 (s, 1H), 7.87 (d, J = 8.1 Hz, 2H), 7.37 (d, J = 8.1 Hz, 2H), 7.30 (d, J = 8.1 Hz, 3H), 2.60 (s, 3H) (other peaks partially obscured). HRMS (ESI+) calcd for $[C_{18}H_{12}N_4OS + H]^+$ 333.00805, found m/z 333.0797.

1,1'-((5-(Pyrimidin-2-yl)-thiadiazole-4,6-diyl)bis(4,1-phenylene))bis(ethan-1-one) (12b)

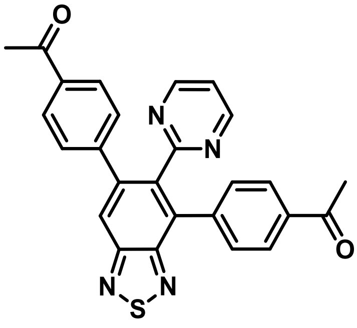

12b

Modified literature procedure.[32] Compound **4g** (21.4 mg, 0.10 mmol, 1.0 equiv.), 4-bromoacetophenone (60 mg, 0.30 mmol, 3.0 equiv.), $[RuCl_2(p\text{-cymene})]_2$ (5 mol%, 3.1 mg, 5.0 μmol), 1-phenyl-1-cyclopentanecarboxylic acid (3 mg, 0.015 mmol, 0.15 equiv.) and K_2CO_3 (42 mg, 0.30 mmol, 3.0 equiv.) were weighed in an oven-dried microwave vial equipped with a stirrer bar, sealed and placed under argon atmosphere. Anhydrous dioxane (0.4 mL) was added and the solution was stirred at 150 °C for 23 h. The reaction mixture was allowed to reach rt, CH_2Cl_2 (5 mL) was added and the salts were filtered off over Celite. The solvent was removed under reduced pressure and the residue was purified by flash column chromatography (SiO_2, eluent: hexane/EtOAc gradient from 55:45 to 50:50). The title compound **12b** was obtained as an off-white solid (40 mg, 0.089 mmol, 89%). R_f = 0.19 (hexane/EtOAc 50:50). ^1H NMR (400 MHz, $CDCl_3$) δ 8.46 (d, J = 5.0 Hz, 2H), 8.14 (s, 1H), 7.91 – 7.85 (m, 2H), 7.84 – 7.77 (m, 2H), 7.46 – 7.40 (m, 2H), 7.33 – 7.28 (m, 2H), 6.98 (t, J = 4.9 Hz, 1H), 2.59 (s, 3H), 2.57 (s, 3H). $^{13}C\{^1H\}$ NMR (101 MHz, $CDCl_3$) δ: 197.9, 197.8, 166.3, 156.5, 154.4, 153.8, 145.1, 142.9, 141.0, 138.9, 136.3, 136.0, 133.3, 130.7, 129.4, 128.2, 128.0, 121.9, 119.1, 26.8, 26.8. HRMS (ESI+) calcd for $[C_{26}H_{18}N_4O_2S + H]^+$ 451.1223, found m/z 451.1214.

1-(4-(6-(3-Methoxyphenyl)-5-(pyrimidin-2-yl)-2,1,3-benzothiadiazol-4-yl)phenyl)ethan-1-one (12c) and 1-(4-(7-(3-methoxyphenyl)-6-(pyrimidin-2-yl)-2,1,3-benzothiadiazol-5-yl)phenyl)ethan-1-one (12c')

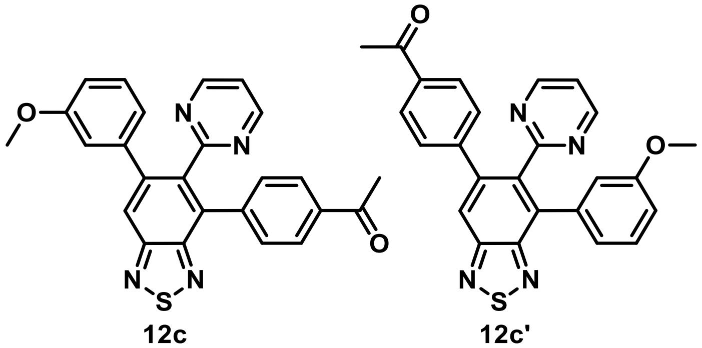

12c 12c'

Modified literature procedure.[32] The mixture of **12a/12a'** (regioisomeric ratio ~15:1, see above) (30 mg, 0.090 mmol, 1.0 equiv.), 3-bromoanisole (18 mg, 0.95 mmol, 1.05 equiv.), $[RuCl_2(p\text{-cymene})]_2$ (3 mol%, 1.7 mg, 3.0 μmol), 1-phenyl-1-cyclo-pentanecarboxylic acid (10 mol%, 1.7 mg, 0.01 mmol) and K_2CO_3

(26 mg, 0.19 mmol, 2.1 equiv.) were weighed in an oven-dried microwave vial equipped with a stirrer bar, sealed and placed under argon atmosphere. Anhydrous dioxane (0.36 mL) was added and the solution was stirred at 150 °C for 19 h. The reaction mixture was allowed to reach rt, CH_2Cl_2 (5 mL) was added and the salts were filtered off through a layer of Celite. The solvent was removed under reduced pressure and the residue was purified by flash column chromatography (SiO_2, eluent: hexane/EtOAc gradient from 55:45 to 50:50). The title compound **12c** was obtained as an off-white solid (31 mg, 0.071 mmol, 78%, **12c/12c'** = ~17:1 based on the ^{1}H NMR spectrum). R_f = 0.21 (hexane/EtOAc 50:50). **Major isomer (12c):** ^{1}H NMR (400 MHz, $CDCl_3$) δ_{major} 8.49 (d, *J* = 5.0 Hz, 2H), 8.14 (s, 1H), 7.88 (d, *J* = 7.7 Hz, 2H), 7.44 (d, *J* = 7.7 Hz, 2H), 7.13 (dd, *J* = 8.2, 7.5 Hz, 1H), 6.99 (t, *J* = 4.9 Hz, 1H), 6.83 – 6.71 (m, 3H), 3.65 (s, 3H), 2.58 (s, 3H). $^{13}C\{^{1}H\}$ NMR (101 MHz, $CDCl_3$) δ_{major} 197.9, 166.7, 159.4, 156.4, 154.6, 153.6, 143.9, 141.4, 141.3, 139.2, 136.2, 132.9, 130.8, 129.3, 128.0, 121.7, 121.7, 118.9, 114.1, 113.9, 55.3, 26.8. **Minor isomer** (**12c'**, tentative assignment): ^{1}H NMR (400 MHz, $CDCl_3$) δ_{minor} 8.09 (s, 1H), 7.78 (d, *J* = 8.5 Hz, 2H), 7.36-7.26 (m, 5H), 7.23-7.17 (m, 3H), 6.92 (d, *J* = 7.5 Hz, 1H), 2.55 (s, 3H) (other peaks partially obscured). HRMS (ESI+) calc for $[C_{25}H_{18}N_4O_2S + H]^+$ 439.1223, found m/z 439.1216.

(*E*)-5-(Pyrimidin-2-yl)-4-styryl-2,1,3-benzothiadiazole (13a) and 5-(pyrimidin-2-yl)-4,6-di((*E*)-styryl)-2,1,3-benzothiadiazole (13b)

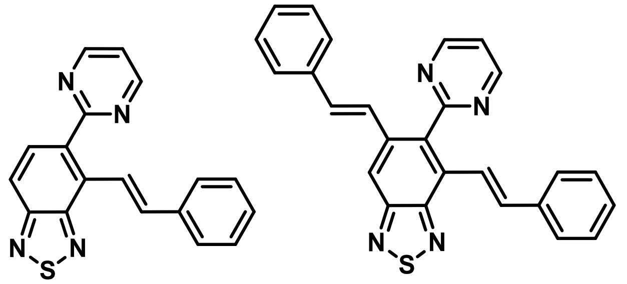

13a **13b**

Modified literature procedure.[33] Compound **4g** (21 mg, 0.10 mmol, 1.0 equiv.), $[RhCp^*Cl_2]_2$ (2.5 mol%, 1.6 mg, 2.5 μmol) and AgF_6Sb (5.2 mg, 15 μmol, 0.15 equiv.) were weighed in an oven-dried microwave vial equipped with a stirrer bar, sealed and placed under argon atmosphere. Acetic acid (0.5 mL), which was previously passed over activated basic alumina, and phenylacetylene (13 μL, 12 mg, 0.12 mmol, 1.2 equiv.) were added to the vial and the solution was stirred at rt for 42 h. The reaction mixture was filtered through Celite and washed with EtOAc before concentrating the crude product under reduced pressure. Flash column chromatography (SiO_2, eluent: hexane/EtOAc gradient from 95:5 to 75:25) afforded the monosubstituted product **13a** (11 mg, 0.038 mmol, 38%) as a yellow solid and the disubstituted product **13b** (7 mg, 0.017 mmol, 17%) as a yellow solid.

Monosubstituted product 13a: ^{1}H NMR (400 MHz, $CDCl_3$) δ 8.97 (d, *J* = 4.9 Hz, 2H), 8.34 (d, *J* = 16.3 Hz, 1H), 8.08 (d, *J* = 9.1 Hz, 1H), 7.98 (d, *J* = 9.1 Hz, 1H), 7.79 (d, *J* = 16.3 Hz, 1H), 7.56 – 7.50 (m, 2H), 7.39 – 7.32 (m, 3H), 7.32 – 7.27 (m, 1H). $^{13}C\{^{1}H\}$ NMR (101 MHz, $CDCl_3$) δ: 167.0, 157.4, 156.0, 154.0, 138.1, 137.9, 137.3, 132.8, 129.6, 128.8, 128.3, 127.2, 123.6, 119.9, 119.3. HRMS (ESI+) calc for $[C_{18}H_{12}N_4S + H]^+$ 317.0855, found m/z 317.0847.

Disubstituted product 13b: ^{1}H NMR (400 MHz, $CDCl_3$) δ 9.00 (d, *J* = 5.0 Hz, 2H), 8.23 (s, 1H), 8.14 (d, *J* = 16.3 Hz, 1H), 7.44 (t, *J* = 5.0 Hz, 1H), 7.39 – 7.34 (m, 2H), 7.34 – 7.28 (m, 6H), 7.25 – 7.20 (m, 2H), 7.12 (d, *J* = 15.9 Hz, 1H), 6.91 (d, *J* = 16.2 Hz, 1H), 6.66 (d, *J* = 15.9 Hz, 1H). $^{13}C\{^{1}H\}$ NMR (101 MHz, $CDCl_3$)

δ:167.2, 157.6, 155.7, 153.0, 139.5, 138.7, 137.8, 137.8, 137.0, 133.2, 128.8, 128.7, 128.7, 128.4, 128.3, 127.1, 127.0, 126.1, 122.9, 119.9, 116.4. HRMS (ESI+) calcd for $[C_{26}H_{18}N_4S + H]^+$ 419.1325, found m/z 419.1317.

(*E*)-1-(4-(5-(Pyrimidin-2-yl)-6-styryl-2,1,3-benzothiadiazol-4-yl)phenyl)ethan-1-one (13c) and (*E*)-1-(4-(6-(pyrimidin-2-yl)-7-styryl-2,1,3-benzothiadiazol-5-yl)phenyl)ethan-1-one

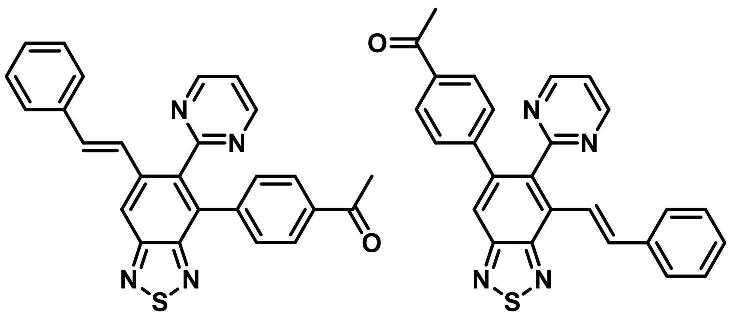

13c **13c'**

Modified literature procedure.[33] The mixture of **12a/12a'** (regioisomeric ratio ~15:1, see above) (20 mg, 0.060 mmol, 1.0 equiv.), $[RhCp^*Cl_2]_2$ (2.5 mol%, 1.1 mg, 1.8 μmol) and AgF_6Sb (3.1 mg, 9.0 μmol, 0.15 equiv.) were weighed in an oven-dried microwave vial equipped with a stirrer bar, sealed and placed under argon atmosphere. Subsequently, acetic acid (0.3 mL), which was passed over activated basic alumina and briefly sparged with argon, and phenylacetylene (8 μL, 7.4 mg, 72 μmol, 1.2 equiv.) were added. The reaction mixture was stirred at rt for 39 h before the solution was transferred to another sealed microwave vial containing another loading of $[RhCp^*Cl_2]_2$ (2.5 mol%, 1.1 mg, 1.8 μmol) and AgF_6Sb (3.1 mg, 9.0 μmol, 0.15 equiv.). Another portion of phenylacetylene (8.0 μL, 7.4 mg, 72 μmol, 1.2 equiv.) was added and the mixture was stirred at 60 °C for another 20 h. After reaching rt, the reaction mixture was filtered over Celite and washed with EtOAc before removal of the solvent under reduced pressure. Purification by flash column chromatography (SiO_2, eluent: hexane/EtOAc gradient from 80:20 to 35:65) afforded the title compound **13c** as an orange solid (19 mg, 44 μmol, 73%, **13c/13c'** = ~15:1 based on the ^1H NMR spectrum). **Major isomer (13c):** ^1H NMR (400 MHz, $CDCl_3$) δ_{major} 8.73 (d, *J* = 5.0 Hz, 2H), 8.39 (s, 1H), 7.88 (d, *J* = 8.8 Hz, 2H), 7.43 (d, *J* = 7.3 Hz, 2H), 7.36 – 7.27 (m, 5H), 7.22 – 7.15 (m, 2H), 6.84 (d, *J* = 16.3 Hz, 1H), 2.59 (s, 3H). $^{13}C\{^1H\}$ NMR (101 MHz, $CDCl_3$) δ_{major} 197.9, 166.4, 157.0, 155.0, 153.7, 141.21, 139.5, 139.1, 136.8, 136.2, 133.7, 132.7, 130.7, 128.8, 128.5, 128.0, 127.1, 125.9, 119.5, 117.4, 26.8. **Minor isomer** (**13c'**, tentative assignment): ^1H NMR (400 MHz, $CDCl_3$) δ_{minor} 8.94 (s, 1H), 8.77 (d, *J* = 4.8 Hz, 2H), 8.24 (d, *J* = 16.4 Hz, 1H), 7.96 (s, 1H), 7.80 (d, *J* = 7.9 Hz, 2H), 7.54-7.45 (m, 5H), 7.07 (d, *J* = 16.4 Hz, 1H) (other peaks partially obscured). HRMS (ESI+) calcd for $[C_{26}H_{18}N_4OS + H]^+$ 435.1274, found m/z 435.1268.

4-(5-Methylthiophen-2-yl)-5-(pyrimidin-2-yl)-2,1,3-benzothiadiazole (14)

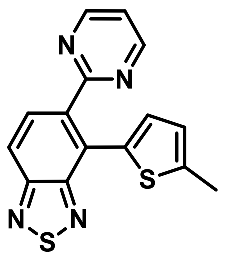

14

Modified literature procedure.[34] Compound **4g** (21 mg, 0.10 mmol, 1.0 equiv.), $[RhCp^*Cl_2]_2$ (10 mol%, 6.2 mg, 10 μmol), AgF_6Sb (14 mg, 40 μmol, 0.4 equiv.), Ag_2CO_3 (124 mg, 0.45 mmol, 4.5 equiv.) and pivalic acid (41 mg, 0.40 mmol, 4.0 equiv.) were weighed in an oven-dried microwave vial equipped with a stirrer bar, sealed and placed under argon atmosphere. Anhydrous DMF (0.8 mL) and methylthiophene (39 μL, 39 mg, 0.4 mmol, 4.0 equiv.) were added. The reaction mixture was stirred at 140 °C for 49 h. After reaching rt, the solvent was partially removed under reduced pressure. The residue was dissolved in CH_2Cl_2, filtered through Celite and washed with CH_2Cl_2. The solvent was removed under reduced pressure and the crude product was purified by flash column chromatography (SiO_2, eluent: hexane/EtOAc gradient from 90:10 to 65:35) to afford the title compound **14** as an orange solid (15 mg, 0.48 mmol, 48%). ^{1}H NMR (400 MHz, $CDCl_3$) δ 8.80 (d, *J* = 4.9 Hz, 2H), 8.03 (d, *J* = 9.0 Hz, 1H), 7.93 (d, *J* = 9.1 Hz, 1H), 7.26 (m, 1H), 6.67 (dt, *J* = 3.4, 1.1 Hz, 1H), 2.47 (s, 3H). ^{13}C{^{1}H} NMR (101 MHz, $CDCl_3$) δ 167.5, 157.3, 155.2, 154.7, 142.7, 137.9, 134.4, 132.2, 130.3, 127.3, 125.5, 120.3, 119.5, 15.5. HRMS (ESI+) calcd for $[C_{15}H_{10}N_4S_2 + H]^+$ 311.0420, found m/z 311.0415.

Methyl (*E*)-3-(4,6-dibutyl-2,1,3-benzothiadiazol-5-yl)acrylate (15)

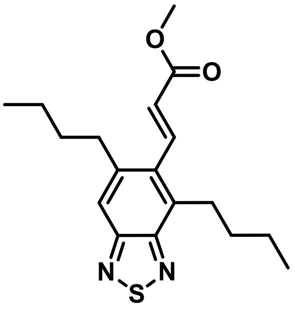

15

Modified literature procedure.[35] Compound **5c** (26 mg, 0.10 mmol, 1.0 equiv.), $Pd(OAc)_2$ (10 mol%, 2.3 mg, 0.010 mmol), tri(2-furyl)phosphine (20 mol%, 4.6 mg, 0.020 mmol), norbornene (57 mg, 0.60 mmol, 6.0 equiv.) and Cs_2CO_3 (163 mg, 0.50 mmol, 5.0 equiv.) were weighed in an oven-dried microwave vial equipped with a stirrer bar, sealed and placed under argon atmosphere. After addition of anhydrous dioxane (1 mL) the solution was stirred at rt for 10 min. Methyl acrylate (18 μL, 17 mg, 0.20 mmol, 2.0 equiv.) and iodobutane (68 μL, 110 mg, 0.60 mmol, 6.0 equiv.) were added and the solution was stirred at 80 °C for 24 h. After reaching rt, the reaction mixture was filtered through Celite and washed with EtOAc. The solvent was removed under reduced pressure and the crude product was purified by flash column chromatography (SiO_2, eluent: 100% hexane, then manual column: eluent: hexane/CH_2Cl_2 98:2 to 90:10 to 100% CH_2Cl_2). The title compound **15** was obtained as a colorless oil (19 mg, 0.57 mmol, 57%). ^{1}H NMR (400 MHz, $CDCl_3$) δ 7.92 (dd, *J* = 16.3, 2.0 Hz, 1H), 7.65 (s, 1H), 6.11 (dd, *J* = 16.2, 2.5 Hz, 1H), 3.86 (s, 3H), 3.22 – 3.00 (m, 2H), 2.80 – 2.58 (m, 2H), 1.72 – 1.55 (m, 4H), 1.50 – 1.35 (m, 4H), 0.94 (ddt, *J* = 8.8, 5.1, 1.8 Hz, 6H). ^{13}C{^{1}H} NMR (101 MHz, $CDCl_3$) δ 166.6, 154.7, 154.1, 143.8, 142.9, 135.2, 133.9, 125.3, 117.6, 52.1, 34.3, 32.8, 32.23, 29.9, 23.1, 22.6, 14.0 (2 CH_3). HRMS (EI+) calcd for $[C_{18}H_{24}N_2O_2S]^+$ 332.1553, found m/z 332.1551.

5) Generation and capture of 3,1,2-Benzothiadiazol-4,5-yne

6-Benzyl-6*H*-[1,2,3]triazolo[4',5':3,4]benzo[1,2-*c*][1,2,5]thiadiazole (18a)

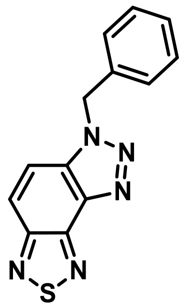

18a

To a flame-dried 10 mL microwave vial equipped with a stirrer bar under argon was added compound **6** (111 mg, 0.20 mmol, 1.0 equiv.), benzyl azide (0.5 M in CH_2Cl_2, 0.80 mL, 0.40 mmol, 2.0 equiv.), the vial was sealed and the atmosphere was exchanged again for argon. MTBE (5 mL) was added. While stirring at rt, *tert*-BuOK (37 mg, 0.33 mmol, 1.5 equiv.) was added in one portion. The heterogenous mixture was stirred at rt for 4 h. Brine (5 mL) was added, and the mixture was extracted with EtOAc (3 x 10 mL), the organic phases were combined, dried (Na_2SO_4), filtered and concentrated under reduced pressure. The crude product was purified by column chromatography (SiO_2, eluent: petroleum ether/EtOAc 4:1) to afford **18a** (17 mg, 0.063 mmol, 31%) as a pale yellow solid. ^1H NMR (400 MHz, $CDCl_3$) δ 7.90 (d, *J* = 9.5 Hz, 1H), 7.54 (d, *J* = 9.5 Hz, 1H), 7.41 – 7.33 (m, 3H), 7.33 – 7.27 (m, 2H), 5.95 (s, 2H). $^{13}C\{^1H\}$ NMR (101 MHz, $CDCl_3$) δ 154.0, 147.1*, 138.4*, 134.1, 133.7*, 129.3, 128.9, 127.5, 121.7, 113.9, 53.0. *signals did not appear in the ^{13}C NMR spectrum due to low concentration but could be detected through HMBC correlations. HRMS (EI+) calcd for $[C_{13}H_9N_5S]^+$ 267.0573, found m/z 267.0571.

6,9-Dimethyl-6,7,8,9-tetrahydro-10*H*-[1,2,5]thiadiazolo[3',4':3,4]benzo[1,2-*e*][1,4]diazepin-10-one (18b)

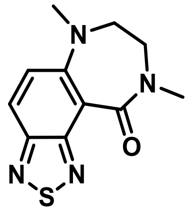

18b

To a flame-dried 25 mL microwave vial equipped with a stirrer bar under argon was added compound **6** (139 mg, 0.25 mmol, 1.0 equiv.), 1,3-dimethyl-2-imidazolidinone (68 µL, 0.50 mmol, 2.0 equiv.), the vial was sealed and the atmosphere was exchanged again for argon. MTBE (6.2 mL) was added. While stirring at rt, *tert*-BuOK (47 mg, 0.38 mmol, 1.5 equiv.) was added in one portion. The heterogenous mixture was stirred at rt for 4 h. Brine (15 mL) was added and the mixture was extracted with EtOAc (3 x 15 mL), the organic phases were combined, dried (Na_2SO_4), filtered and concentrated under reduced pressure. The crude product was purified by column chromatography (SiO_2, eluent: CH_2Cl_2/MeOH 50:1) to afford **18b** (13 mg, 0.052 mmol, 21%) as a yellow solid. ^1H NMR (400 MHz, $CDCl_3$) δ 7.90 (d, *J* = 9.6 Hz, 1H), 7.36 (d, *J* = 9.6 Hz, 1H), 3.63 – 3.52 (m, 4H), 3.27 (s, 3H), 3.03 (s, 3H). $^{13}C\{^1H\}$ NMR (101 MHz, $CDCl_3$) δ 167.3, 154.5, 150.9, 146.5, 123.8, 123.4, 113.0, 57.8, 47.3, 40.5, 34.3. HRMS (EI+) calcd for $[C_{11}H_{12}N_4OS]^+$ 248.0726, found m/z 248.0723.

6,9-Dimethyl-6,9-dihydro-6,9-epoxynaphtho[1,2-*c*][1,2,5]thiadiazole (18c)

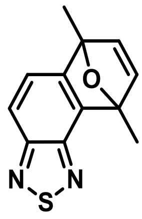

18c

To a flame-dried 10 mL microwave vial equipped with a stirrer bar under argon was added compound **6** (110 mg, 0.20 mmol, 1.0 equiv.), 2,5-dimethylfuran (38 mg, 0.40 mmol, 2.0 equiv.), the vial was sealed and the atmosphere exchanged again for argon. MTBE (5 mL) was added. While stirring at rt, *tert*-BuOK (33 mg, 0.30 mmol, 1.5 equiv.) was added in one portion. The heterogenous mixture was stirred at rt for 4 h. Brine (10 mL) was added and the mixture was extracted with EtOAc (3 x 10 mL), the organic phases were combined, dried (Na_2SO_4), filtered and concentrated under reduced pressure. The crude product was purified by column chromatography (SiO_2, eluent: EtOAc/petroleum ether 1:9) to afford **18c** (23 mg, 0.098 mmol, 49%) as a yellow solid. ^{1}H NMR (400 MHz, $CDCl_3$) δ 7.79 (d, *J* = 8.6 Hz, 1H), 7.58 (d, *J* = 8.6 Hz, 1H), 7.06 (d, *J* = 5.2 Hz, 1H), 6.96 (d, *J* = 5.2 Hz, 1H), 2.30 (s, 3H), 2.02 (s, 3H). ^{13}C{^{1}H} NMR (101 MHz, $CDCl_3$) δ: 156.8, 154.4, 148.9, 148.4, 148.3, 146.1, 121.3, 118.5, 90.2, 89.8, 16.6, 15.4. HRMS (EI+) calcd for $[C_{12}H_{10}N_2OS]^+$ 230.0508, found m/z 230.0506.

III. Crystallographic details for 18b

Table S2. Crystal data and structure refinement for 18b.

Identification code	ao_lp_fba_115_02_1_0m
CCDC No.	2324934
Empirical formula	$C_{12}H_{13}Cl_3N_4OS$
Formula weight	367.67
Temperature/K	180.00
Crystal system	triclinic
Space group	P-1
a/Å	7.585(4)
b/Å	9.734(5)
c/Å	12.082(6)
α/°	72.670(9)
β/°	81.339(9)
γ/°	70.959(9)
Volume/Å^3	803.6(7)
Z	2
ρ_{calc}g/cm^3	1.520
μ/mm^{-1}	0.703
F(000)	376.0
Crystal size/mm^3	0.25 × 0.2 × 0.15
Radiation	MoKα (λ = 0.71073)
2Θ range for data collection/°	3.538 to 52.984
Index ranges	-9 ≤ h ≤ 9, -12 ≤ k ≤ 12, -15 ≤ l ≤ 15
Reflections collected	16851
Independent reflections	3324 [R_{int} = 0.0353, R_{sigma} = 0.0290]
Data/restraints/parameters	3324/18/220
Goodness-of-fit on F^2	1.154
Final R indexes [I>=2σ (I)]	R_1 = 0.0570, wR_2 = 0.1125
Final R indexes [all data]	R_1 = 0.0791, wR_2 = 0.1214
Largest diff. peak/hole / e Å^{-3}	0.26/-0.22

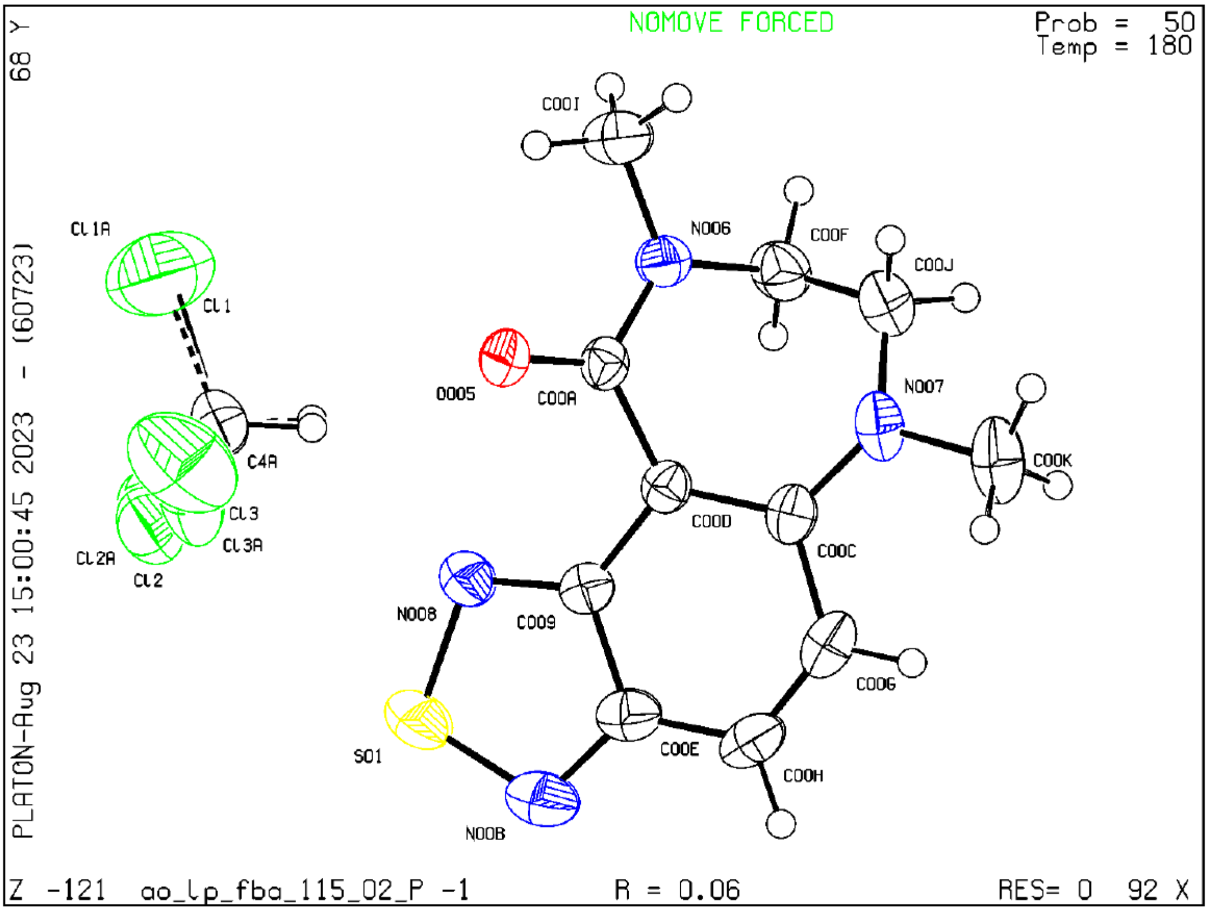

*Figure S3: Thermal ellipsoid plot for the crystal structure of **18b** (thermal ellipsoids are drawn at the 50% probability level).*

IV. DFT Calculation

The geometry calculation of heteroaryne **16** was performed on the Rackham cluster at UPPMAX using the software Gaussian 09 (Revision D.01) at the B3LYP/6-311++G(d,p) level of theory.[36–39]

Energy sums:

Sum of electronic and zero-point Energies = -737.426306 Hartree
Sum of electronic and thermal Energies = -737.420094 Hartree
Sum of electronic and thermal Enthalpies = -737.419149 Hartree
Sum of electronic and thermal Free Energies = -737.457049 Hartree

Calculated geometry for 16:

C	1.22368	-1.50911	0.00013
C	-0.02057	-0.84473	0.00001
C	0.15009	0.62551	0.00001
C	1.44976	1.24290	0.00012
C	2.59796	0.47992	0.00023
C	2.29407	-0.89937	0.00022
N	-1.28251	-1.24190	-0.00011
H	1.50506	2.32472	0.00012
H	3.58914	0.91169	0.00032
N	-1.01472	1.26875	-0.00011
S	-2.19897	0.12531	-0.00021

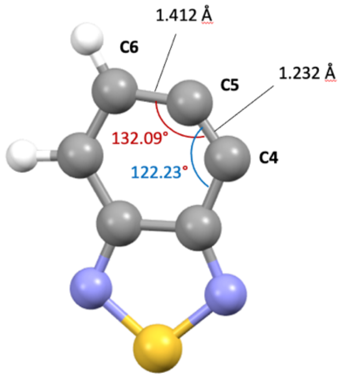

V. References

(1) Kawamorita, S.; Murakami, R.; Iwai, T.; Sawamura, M. Synthesis of Primary and Secondary Alkylboronates through Site-Selective C(Sp3)-H Activation with Silica-Supported Monophosphine-Ir Catalysts. *J. Am. Chem. Soc.* **2013**, *135* (8), 2947–2950.

(2) Hitosugi, S.; Tanimoto, D.; Nakanishi, W.; Isobe, H. A Facile Chromatographic Method for Purification of Pinacol Boronic Esters. *Chem. Lett.* **2012**, *41* (9), 972–973.

(3) Boulton, A. J.; Halls, P. J.; Katritzky, A. R. Applications of Proton Magnetic Resonance Spectroscopy to Structural Problems—XXXIV: Partial Bond Fixation in Bicyclic Heteroaromatic Compounds. *Org. Magn. Reson.* **1969**, *1* (4), 311–317.

(4) Tobiason, F. L.; Goldstein, J. H. The Proton Magnetic Resonance Spectra and Parameters for 2,1,3-Benzoxadiazole, -Benzothiadiazole, and -Benzoselenadiazole. *Spectrochim. Acta Part A Mol. Spectrosc.* **1969**, *25* (5), 1027–1033.

(5) Tajuddin, H.; Harrisson, P.; Bitterlich, B.; Collings, J. C.; Sim, N.; Batsanov, A. S.; Cheung, M. S.; Kawamorita, S.; Maxwell, A. C.; Shukla, L.; et al. Iridium-Catalyzed C-H Borylation of Quinolines and Unsymmetrical 1,2-Disubstituted Benzenes: Insights into Steric and Electronic Effects on Selectivity. *Chem. Sci.* **2012**, *3* (12), 3505–3515.

(6) Anant, P.; Lucas, N. T.; Jacob, J. A Simple Route toward the Synthesis of Bisbenzothiadiazole Derivatives. *Org. Lett.* **2008**, *10* (24), 5533–5536.

(7) Nau, J.; Brüning, V.; Biesen, L.; Knedel, T.; Janiak, C.; Müller, T. J. J. Synthesis and Electronic Properties of Conjugated *Syn* , *Syn* -Dithienothiazine Donor-Acceptor-Donor Dumbbells. *European J. Org. Chem.* **2022**, *2022* (2), e202101398.

(8) Larsen, M. A.; Hartwig, J. F. Iridium-Catalyzed C-H Borylation of Heteroarenes: Scope, Regioselectivity, Application to Late-Stage Functionalization, and Mechanism. *J. Am. Chem. Soc.* **2014**, *136* (11), 4287–4299.

(9) Demory, E.; Devaraj, K.; Orthaber, A.; Gates, P. J.; Pilarski, L. T. Boryl (Hetero)Aryne Precursors as Versatile Arylation Reagents: Synthesis through C-H Activation and Orthogonal Reactivity. *Angew. Chemie Int. Ed.* **2015**, *54* (40), 11765–11769.

(10) Sun, J.; Perfetti, M. T.; Santos, W. L. A Method for the Deprotection of Alkylpinacolyl Boronate Esters. *J. Org. Chem.* **2011**, *76* (9), 3571–3575.

(11) Lennox, A. J. J.; Lloyd-Jones, G. C. Preparation of Organotrifluoroborate Salts: Precipitation-Driven Equilibrium under Non-Etching Conditions. *Angew. Chemie Int. Ed.* **2012**, *51* (37), 9385–9388.

(12) Wollenburg, M.; Moock, D.; Glorius, F. Hydrogenation of Borylated Arenes. *Angew. Chem. Int. Ed.* **2019**, *58* (20), 6549–6553.

(13) Mosleh, I.; Shahsavari, H. R.; Beitle, R.; Beyzavi, M. H. Recombinant Peptide Fusion Protein-Templated Palladium Nanoparticles for Suzuki-Miyaura and Stille Coupling Reactions. *ChemCatChem* **2020**, *12* (11), 2942–2946.

(14) Barnsley, J. E.; Shillito, G. E.; Mapley, J. I.; Larsen, C. B.; Lucas, N. T.; Gordon, K. C. Walking the Emission Tightrope: Spectral and Computational Analysis of Some Dual-Emitting Benzothiadiazole Donor-Acceptor Dyes. *J. Phys. Chem. A* **2018**, *122* (40), 7991–8006.

(15) Gray, N.; Zhang, Tinghu; Kwiatowski, Nicholas; Fan, M.; Che, J.; Lu, W. Inhibitors of

Transcriptional Enhanced Associate Domain (TEAD) And Uses Thereof. WO2021247634 (A1), 2021.

(16) Murphy, J. M.; Liao, X.; Hartwig, J. F. Meta Halogenation of 1,3-Disubstituted Arenes via Iridium-Catalyzed Arene Borylation. *J. Am. Chem. Soc.* **2007**, *129* (50), 15434–15435.

(17) Ma, Y. P.; Wang, G.; Zhao, M.; Shi, Z. F.; Miao, Y.; Cao, X. P.; Zhang, H. L. A Red Thermally Activated Delayed Fluorescence Emitter Based on Benzo[c][1,2,5]Thiadiazole. *Dye. Pigment.* **2023**, *212*, 111084.

(18) Partridge, B. M.; Hartwig, J. F. Sterically Controlled Iodination of Arenes via Iridium-Catalyzed C-H Borylation. *Org. Lett.* **2013**, *15* (1), 140–143.

(19) Nguyen, Y. H.; Lampkin, B. J.; Venkatesh, A.; Ellern, A.; Rossini, A. J.; Vanveller, B. Open-Resonance-Assisted Hydrogen Bonds and Competing Quasiaromaticity. *J. Org. Chem.* **2018**, *83* (17), 9850–9857.

(20) Kopp, J.; Brückner, R. Stereoselective Total Synthesis of the Dimeric Naphthoquinonopyrano-Î-Lactone (-)-Crisamicin A: Introducing the Dimerization Site by a Late-Stage Hartwig Borylation. *Org. Lett.* **2020**, *22* (9), 3607–3612.

(21) Darzi, E. R.; White, B. M.; Loventhal, L. K.; Zakharov, L. N.; Jasti, R. An Operationally Simple and Mild Oxidative Homocoupling of Aryl Boronic Esters To Access Conformationally Constrained Macrocycles. *J. Am. Chem. Soc.* **2017**, *139* (8), 3106–3114.

(22) Liu, S.; He, F.; Yao, L.; Gu, C.; Xu, H.; Xie, Z.; Wu, H.; Ma, Y. Chemistry and Materials Based on 5,5′-Bibenzo[c][1,2,5]Thiadiazole. *Chem. Commun.* **2013**, *49* (51), 5730–5732.

(23) Huang, H.; Yu, C.; Li, X.; Zhang, Y.; Zhang, Y.; Chen, X.; Mariano, P. S.; Xie, H.; Wang, W. Synthesis of Aldehydes by Organocatalytic Formylation Reactions of Boronic Acids with Glyoxylic Acid. *Angew. Chemie Int. Ed.* **2017**, *56* (28), 8201–8205.

(24) Macé, Y.; Bony, E.; Delvaux, D.; Pinto, A.; Mathieu, V.; Kiss, R.; Feron, O.; Quetin-Leclercq, J.; Riant, O. Cytotoxic Activities and Metabolic Studies of New Combretastatin Analogues. *Med. Chem. Res.* **2015**, *24* (8), 3143–3156.

(25) Nykaza, T. V.; Cooper, J. C.; Li, G.; Mahieu, N.; Ramirez, A.; Luzung, M. R.; Radosevich, A. T. Intermolecular Reductive C-N Cross Coupling of Nitroarenes and Boronic Acids by PIII/PV=O Catalysis. *J. Am. Chem. Soc.* **2018**, *140* (45), 15200–15205.

(26) Miyake, N. .; Okada, S. .; Irisawa, J. .; Konishi, T. .; Matsuura, K. Near-Infrared-Absorbing Dye, Optical Filter, and Imaging Device. US2018/0346729A1, 2018.

(27) Vantourout, J. C.; Law, R. P.; Isidro-Llobet, A.; Atkinson, S. J.; Watson, A. J. B. Chan−Evans−Lam Amination of Boronic Acid Pinacol (BPin) Esters: Overcoming the Aryl Amine Problem. *J. Org. Chem* **2016**, *81*, 3950.

(28) Campeau, L. C.; Parisien, M.; Jean, A.; Fagnou, K. Catalytic Direct Arylation with Aryl Chlorides, Bromides, and Iodides: Intramolecular Studies Leading to New Intermolecular Reactions. *J. Am. Chem. Soc.* **2006**, *128* (2), 581–590.

(29) Brunner, K.; Van Dijken, A.; Hofstraat, J. W. .; Boerner, H.; Langeveld-Voss, B. M. W. .; Kiggen, N. M. T. .; Bastiaansen, J. J. A. M. .; Schoo, H. F. M. 9,9′- And 2,2′-Substituted 3,3′-Bicarbazolyl Derivatives For Use In Semiconducting Materials As A Host Matrix For Phosphorescent Emitters. EP1838671 (A2), 2008.

(30) Mataka, S.; Takahashi, K.; Ikezaki, Y.; Hatta, T.; Torm, A.; Tashiro, M. Sulfur Nitride in Organic Chemistry. Part 19.1) Selective Formation of Benzo- and Benzobis[l,2,5]Thiadiazole Skeleton in

the Reaction of Tetrasulfur Tetranitride with Naphthalenols and Related Compounds. *Bull. Chem. Soc. Jpn.* **1991**, *64* (1), 68–73.

(31) Suzuki, C.; Hirano, K.; Satoh, T.; Miura, M. Direct Synthesis of N -H Carbazoles via Iridium(III)-Catalyzed Intramolecular C-H Amination. *Org. Lett.* **2015**, *17* (6), 1597–1600.

(32) Štefane, B.; Fabris, J.; Požgan, F. C-H Bond Functionalization of Arylpyrimidines Catalyzed by an in Situ Generated Ruthenium(II) Carboxylate System and the Construction of Tris(Heteroaryl)-Substituted Benzenes. *European J. Org. Chem.* **2011**, No. 19, 3474–3481.

(33) Duan, C. L.; Liu, X. Y.; Tan, Y. X.; Ding, R.; Yang, S.; Tian, P.; Lin, G. Q. Acetic Acid-Promoted Rhodium(III)-Catalyzed Hydroarylation of Terminal Alkynes. *Synlett* **2019**, *30* (8), 932–938.

(34) Dong, J.; Long, Z.; Song, F.; Wu, N.; Guo, Q.; Lan, J.; You, J. Rhodium or Ruthenium-Catalyzed Oxidative C-H/C-H Cross-Coupling: Direct Access to Extended π-Conjugated Systems. *Angew. Chem. Int. Ed.* **2013**, *52* (2), 580–584.

(35) Fu, J.; Gao, Y.; Qi, X.; Jiang, C. Synthesis of Polysubstituted Pyridines and Indoles by a Palladium-Catalyzed Catellani-Type Alkylation-Alkenylation Sequence. *ChemistrySelect* **2018**, *3* (36), 10164–10168.

(36) Becke, A. D. Density-Functional Thermochemistry. III. The Role of Exact Exchange. *J. Chem. Phys.* **1993**, *98*, 5648.

(37) Lee, C.; Yang, W.; Parr, R. G. Development of the Colle-Salvetti Correlation-Energy Formula into a Functional of the Electron Density. *Phys. Rev. B* **1988**, *37* (2), 785–789.

(38) Vosko, S. H.; Wilk, L.; Nusair, M. Accurate Spin-Dependent Electron Liquid Correlation Energies for Local Spin Density Calculations: A Critical Analysis. *Can. J. Phys.* **1980**, *58*, 1200.

(39) Stephens, P. J.; Devlin, F. J.; Chabalowski, C. F.; Frisch, M. J. Ab Initio Calculation of Vibrational Absorption and Circular Dichroism Spectra Using Density Functional Force Fields. *J. Phys. Chem.* **1994**, *98*, 11623.

VI. NMR Spectra

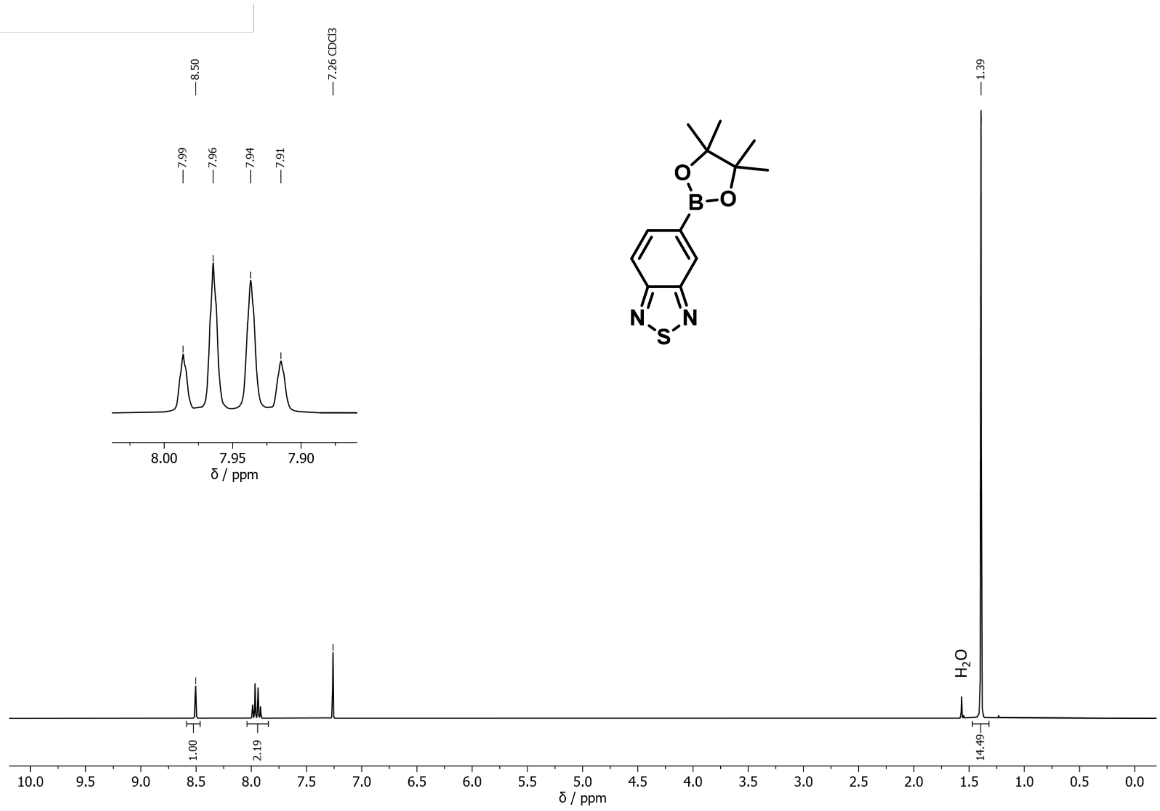

Figure S4: ^{1}H NMR spectrum of ***3a*** *in $CDCl_3$ (400 MHz).*

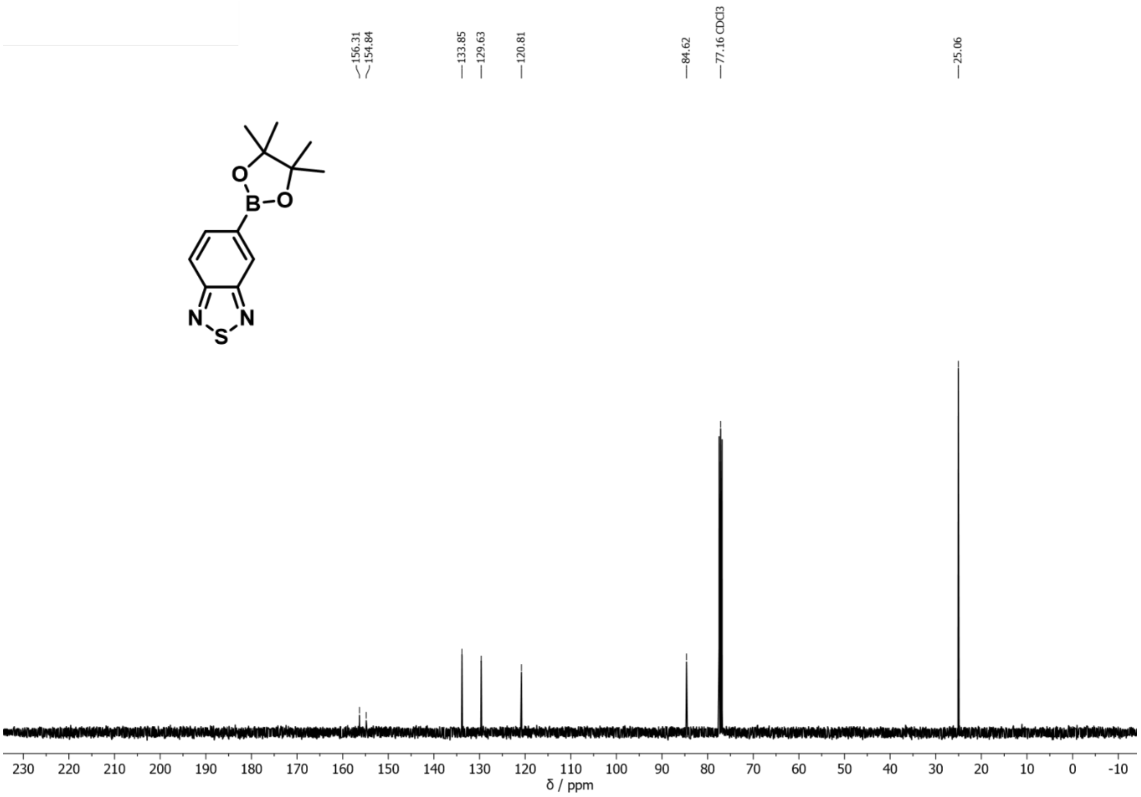

Figure S5: $^{13}C\{^{1}H\}$ NMR spectrum of ***3a*** *in $CDCl_3$ (101 MHz).*

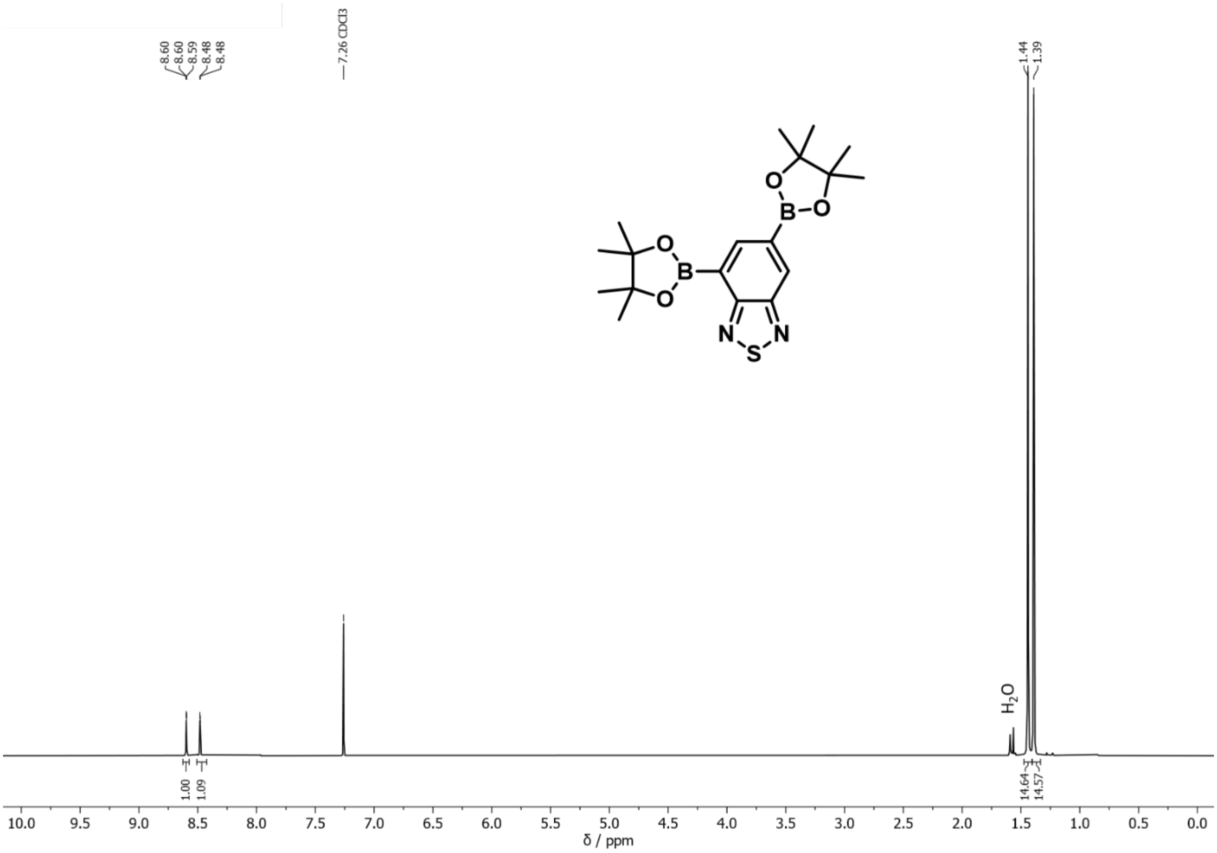

*Figure S6: ^{1}H NMR spectrum of **3c** in $CDCl_3$ (400 MHz).*

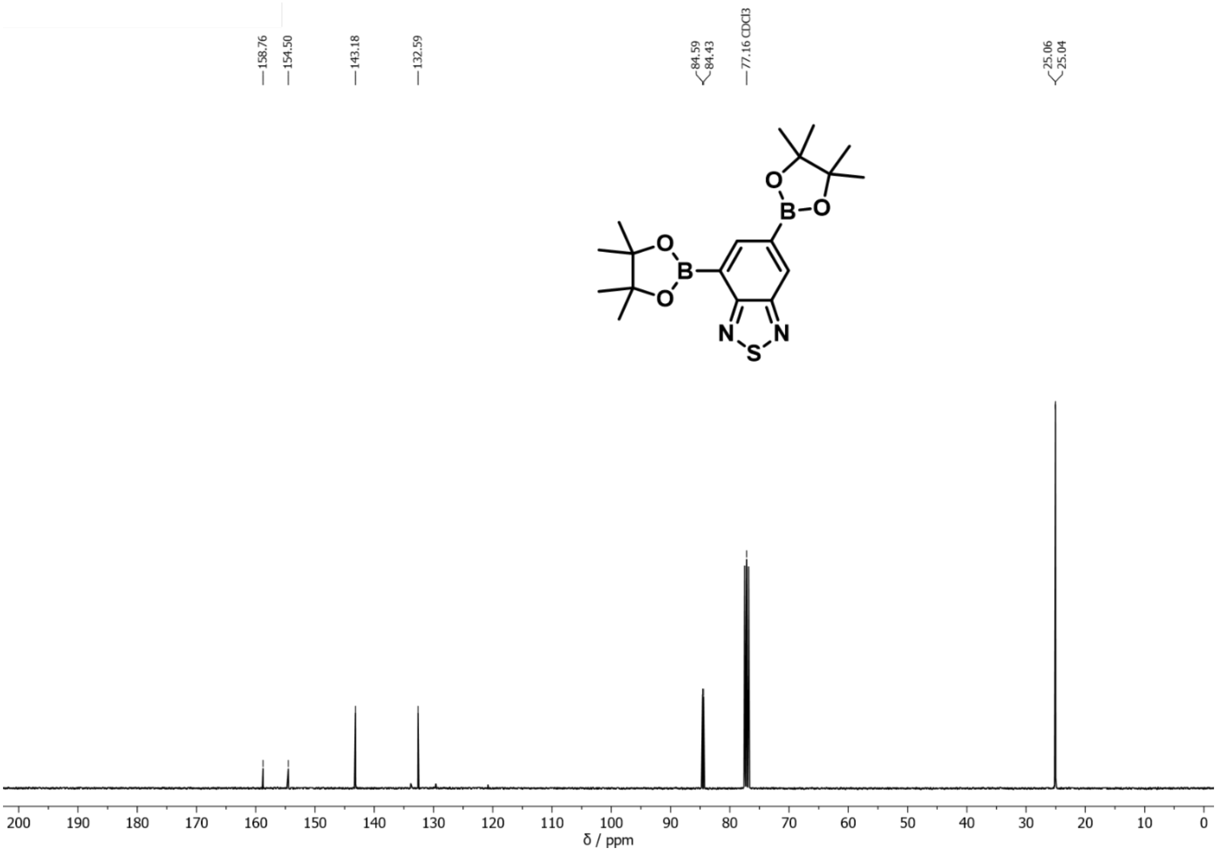

*Figure S7: $^{13}C\{^{1}H\}$ NMR spectrum of **3c** in $CDCl_3$ (101 MHz).*

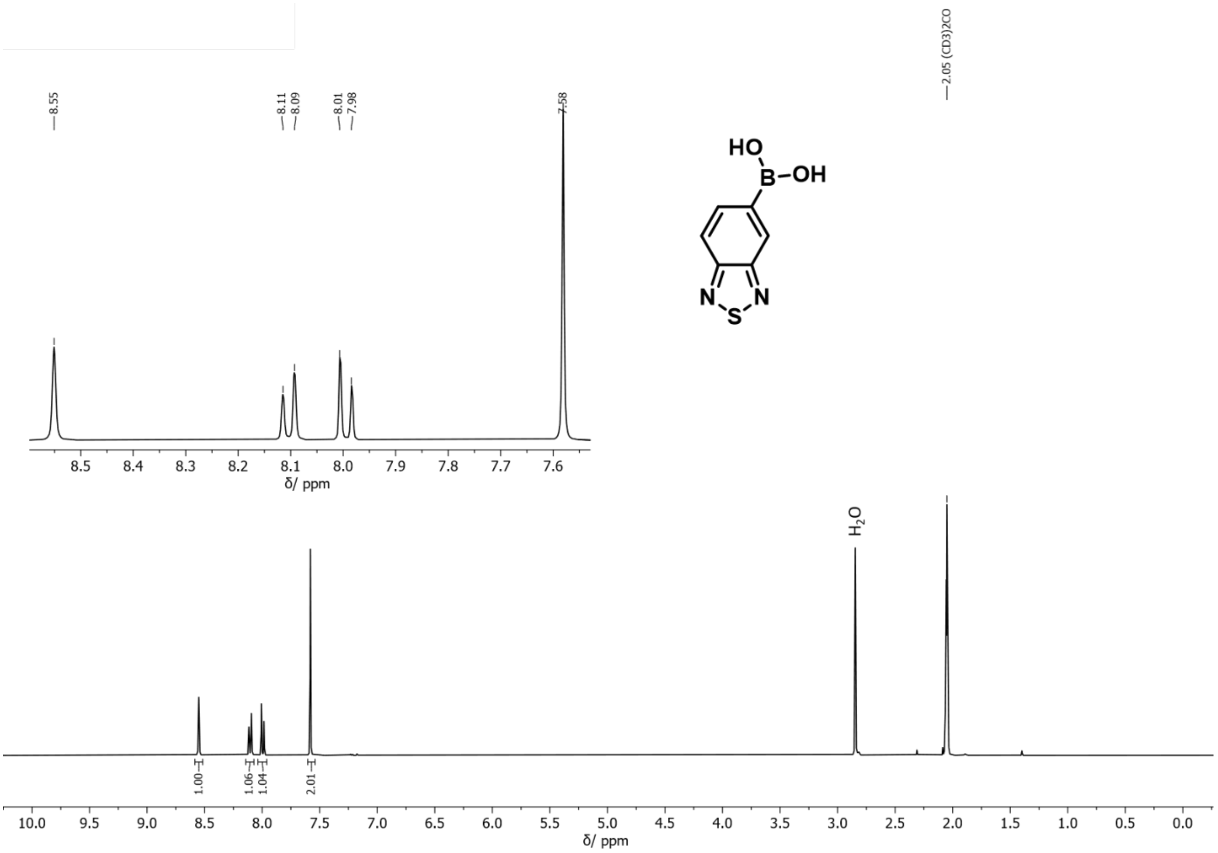

*Figure S8: ^{1}H NMR spectrum of **3e** in acetone-d_6 (400 MHz).*

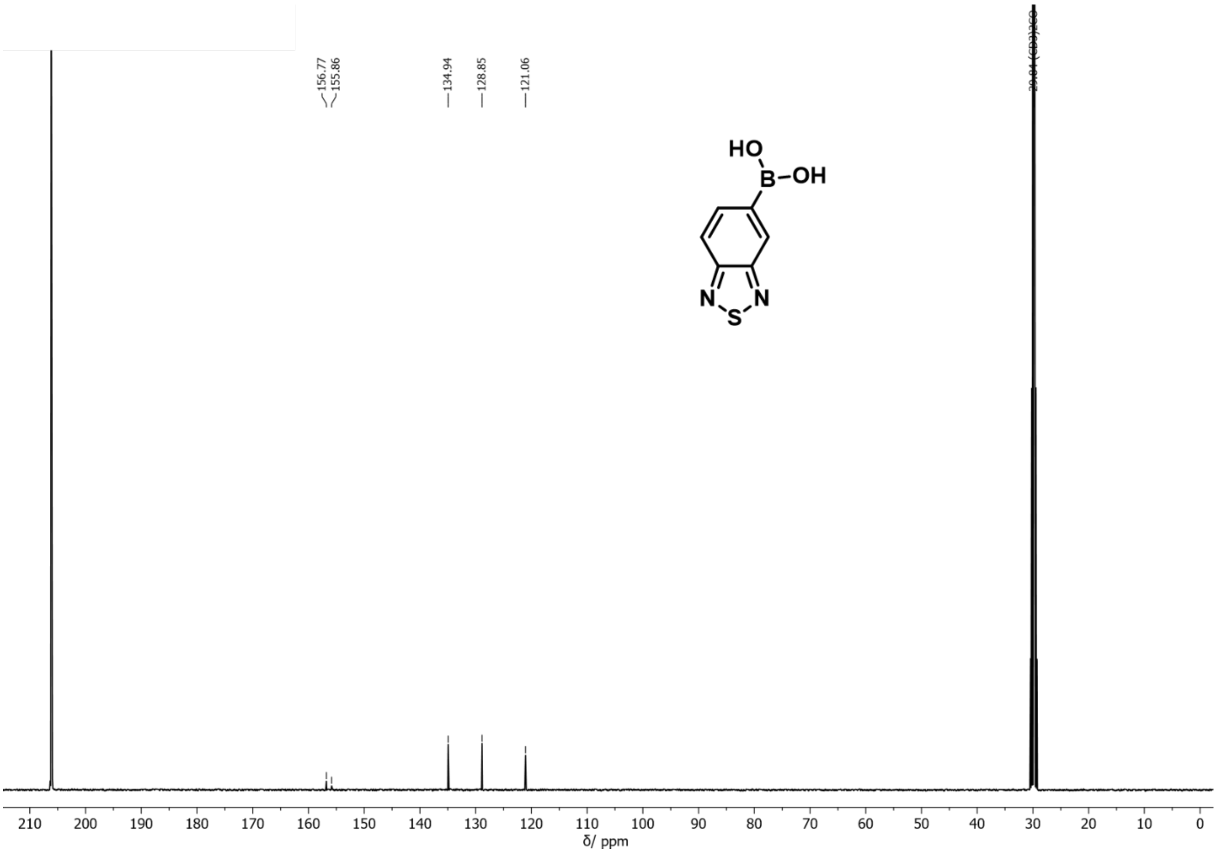

*Figure S9: ^{13}C{^{1}H} NMR spectrum of **3e** in acetone-d_6 (101 MHz).*

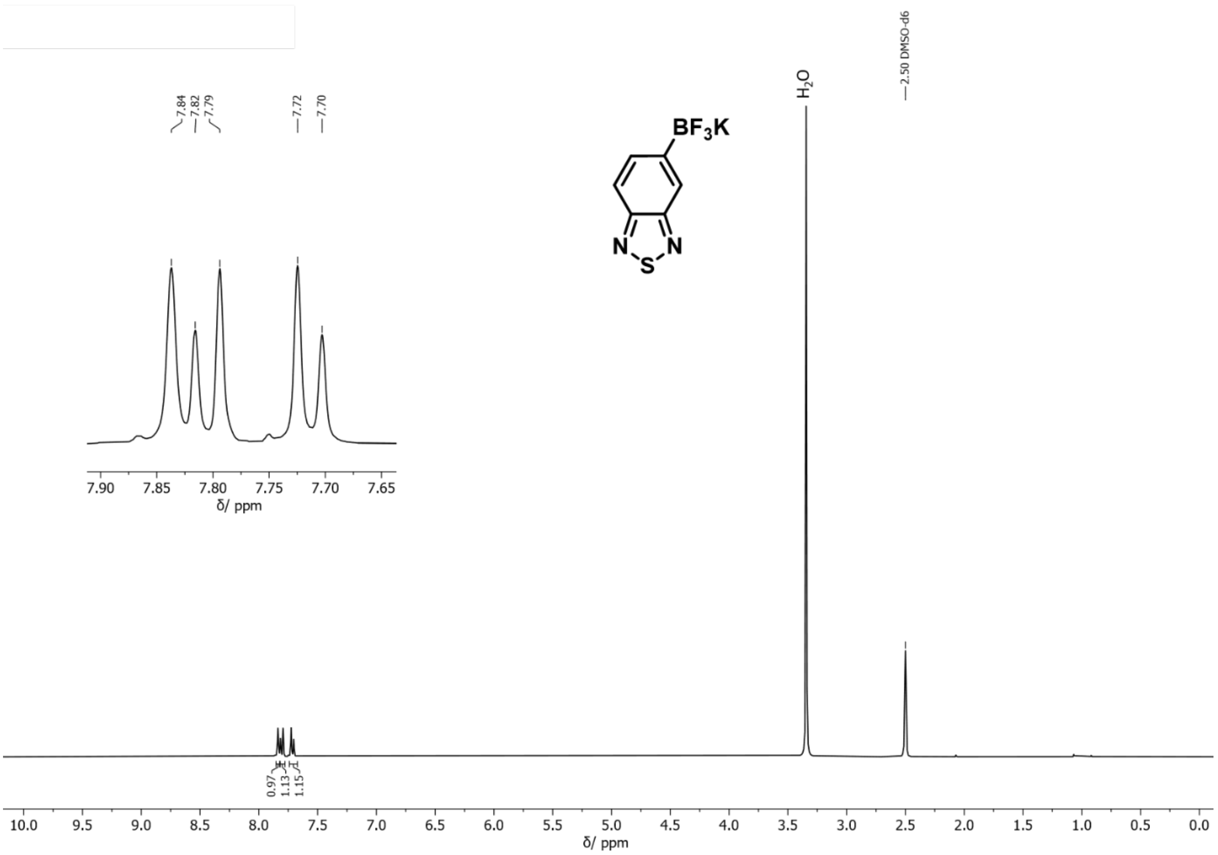

*Figure S10: ^{1}H NMR spectrum of **3f** in DMSO-d_6 (400 MHz).*

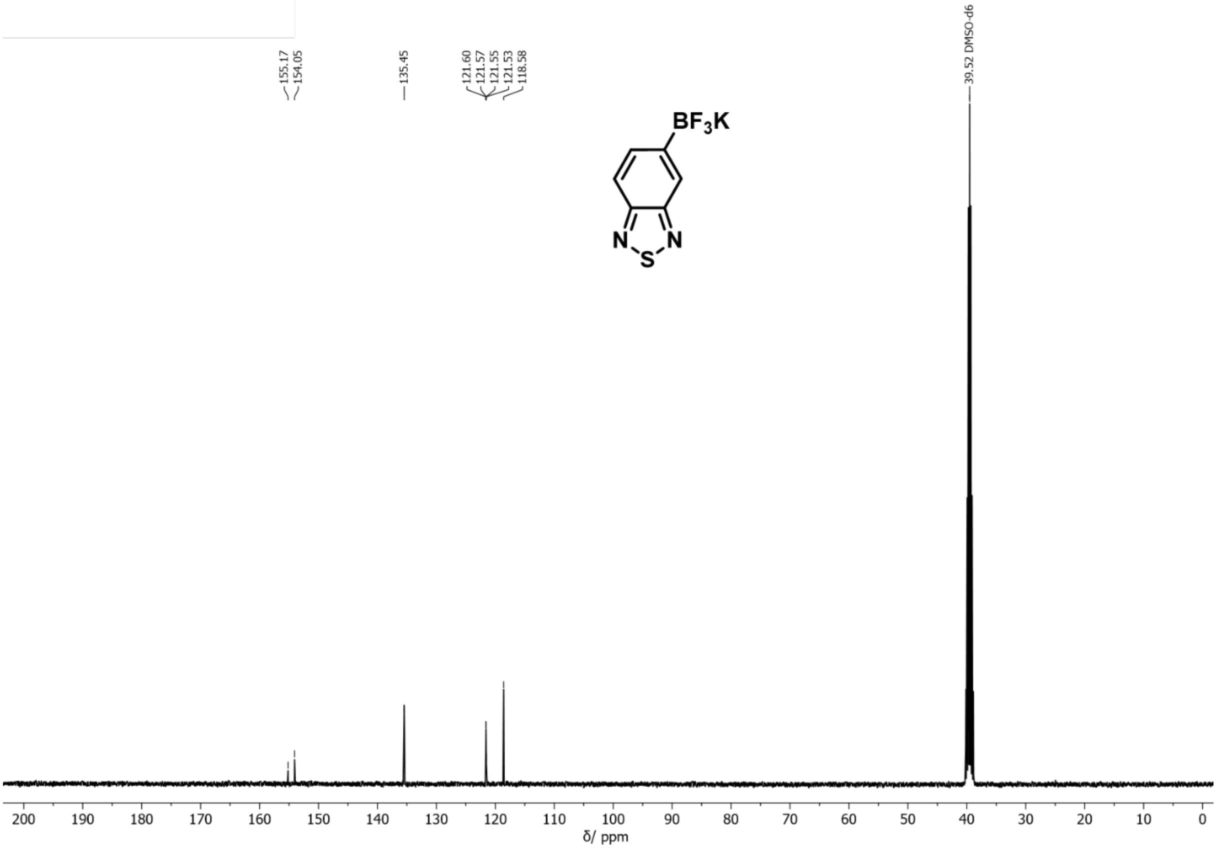

*Figure S11: $^{13}C\{^{1}H\}$ NMR spectrum of **3f** in DMSO-d_6 (101 MHz).*

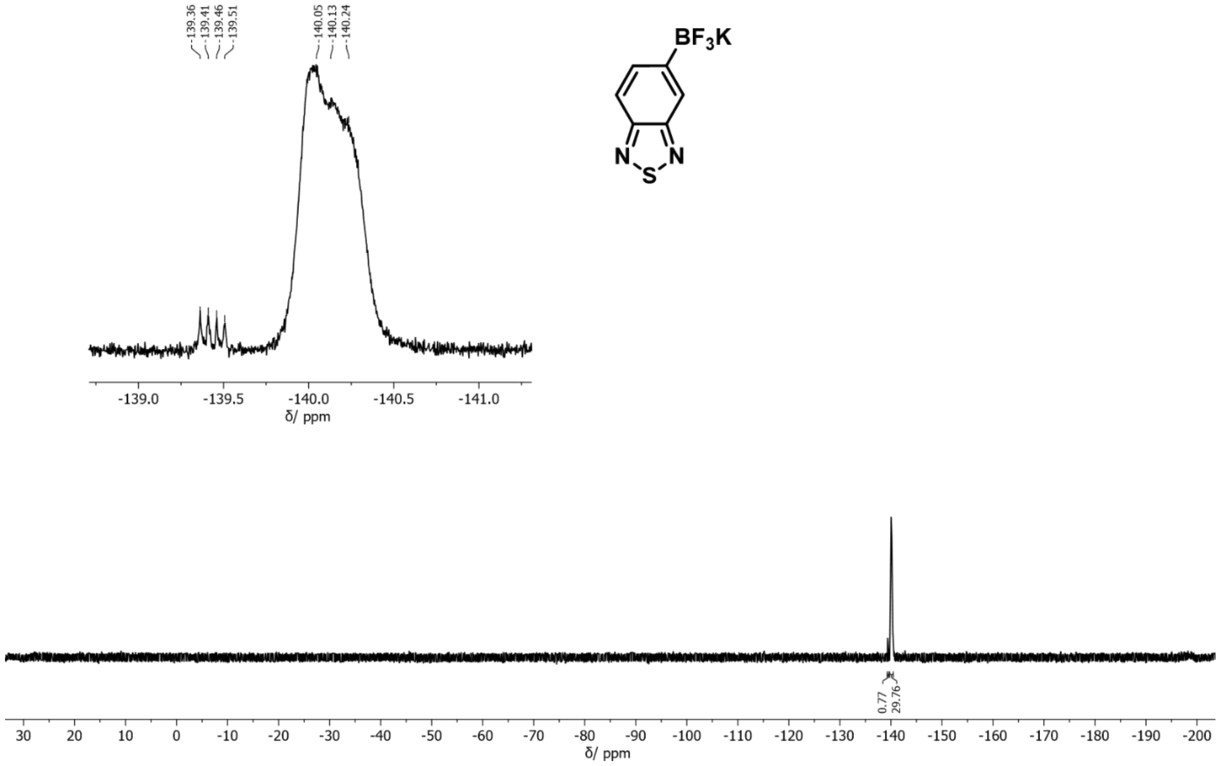

*Figure S12: ^{19}F NMR spectrum of **3f** in DMSO-d$_6$ (376 MHz).*

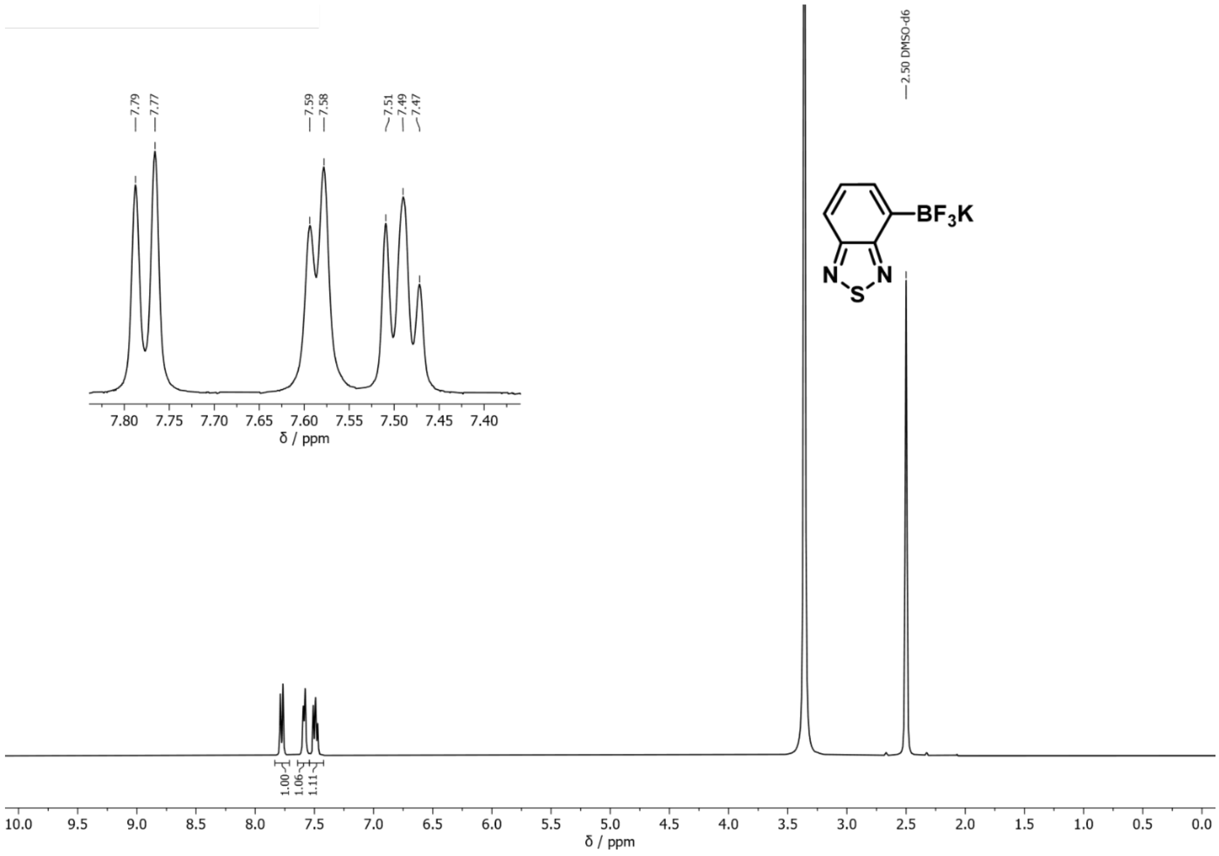

*Figure S13: ^{1}H NMR spectrum of **3f'** in DMSO-d$_6$ (400 MHz).*

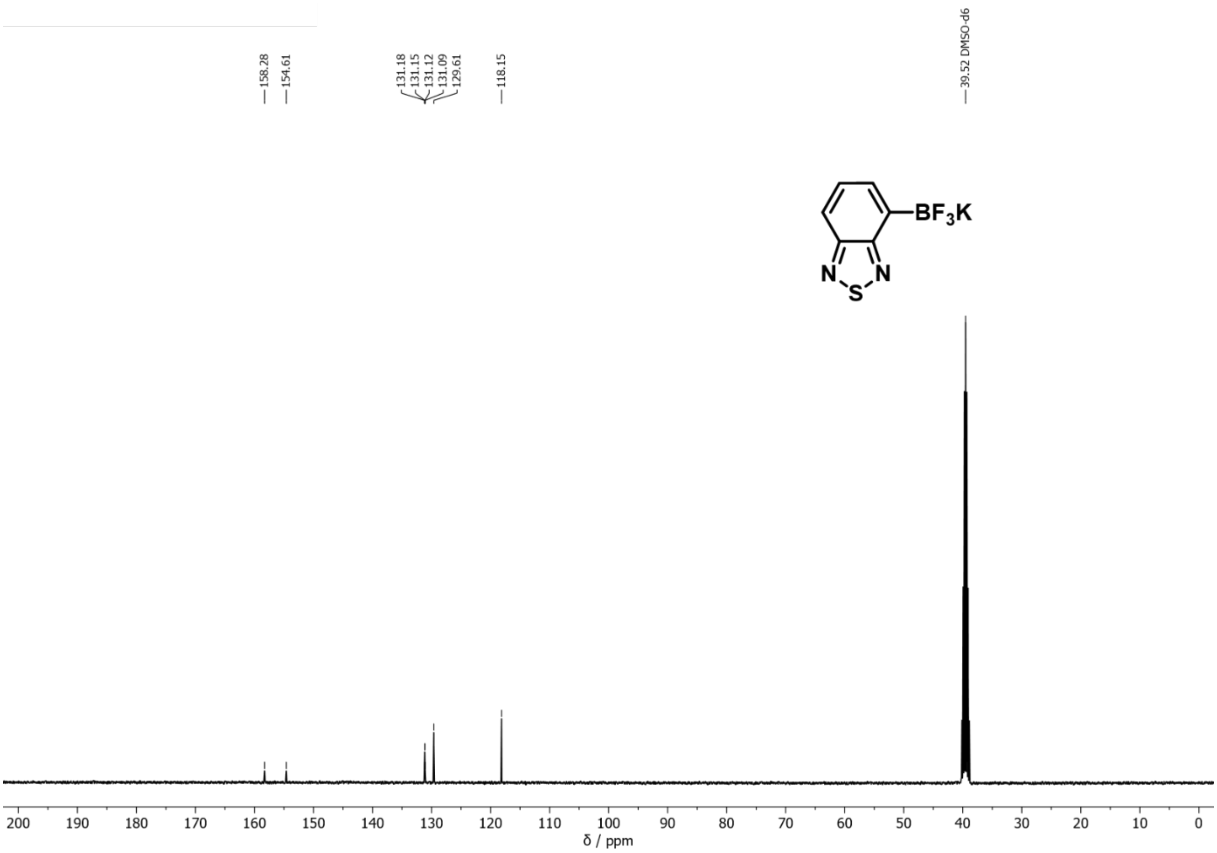

*Figure S14: $^{13}C\{^{1}H\}$ NMR spectrum of **3f'** in DMSO-d_6 (101 MHz).*

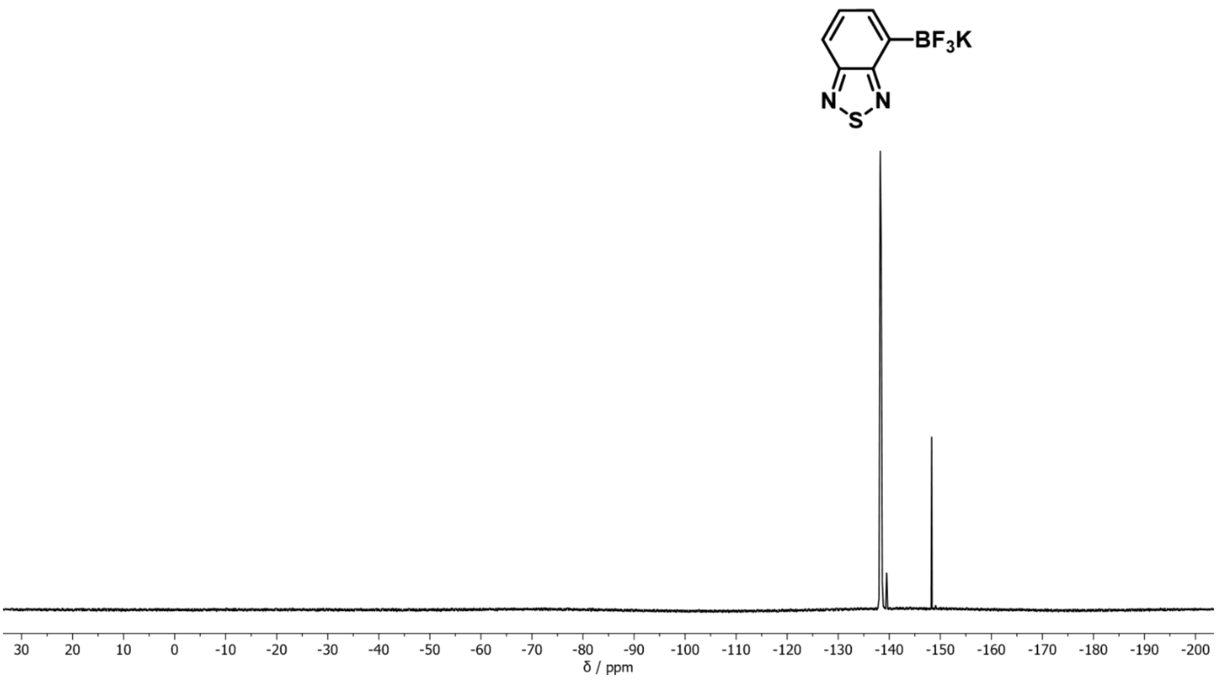

*Figure S15: ^{19}F NMR spectrum of **3f'** in DMSO-d_6 (376 MHz).*

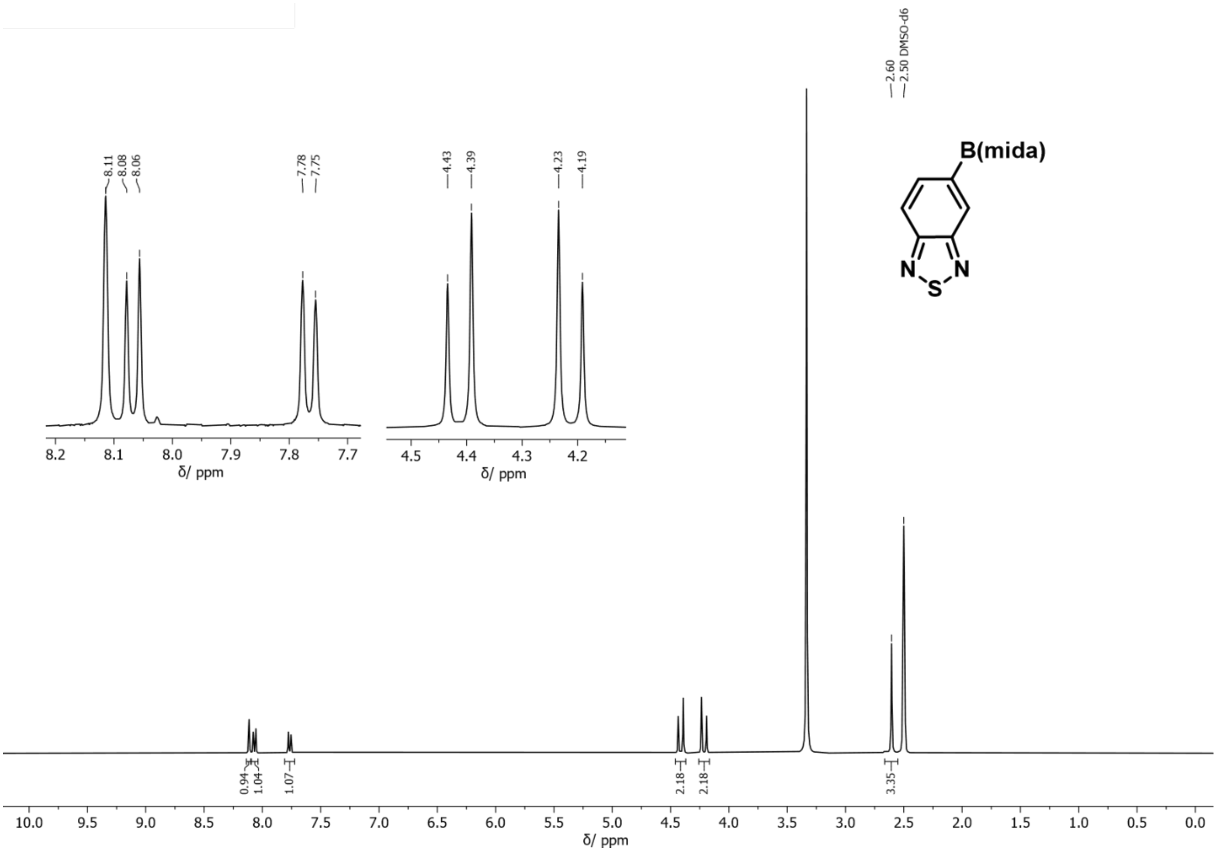

*Figure S16: ^{1}H NMR spectrum of **3g** in DMSO-d_6 (400 MHz).*

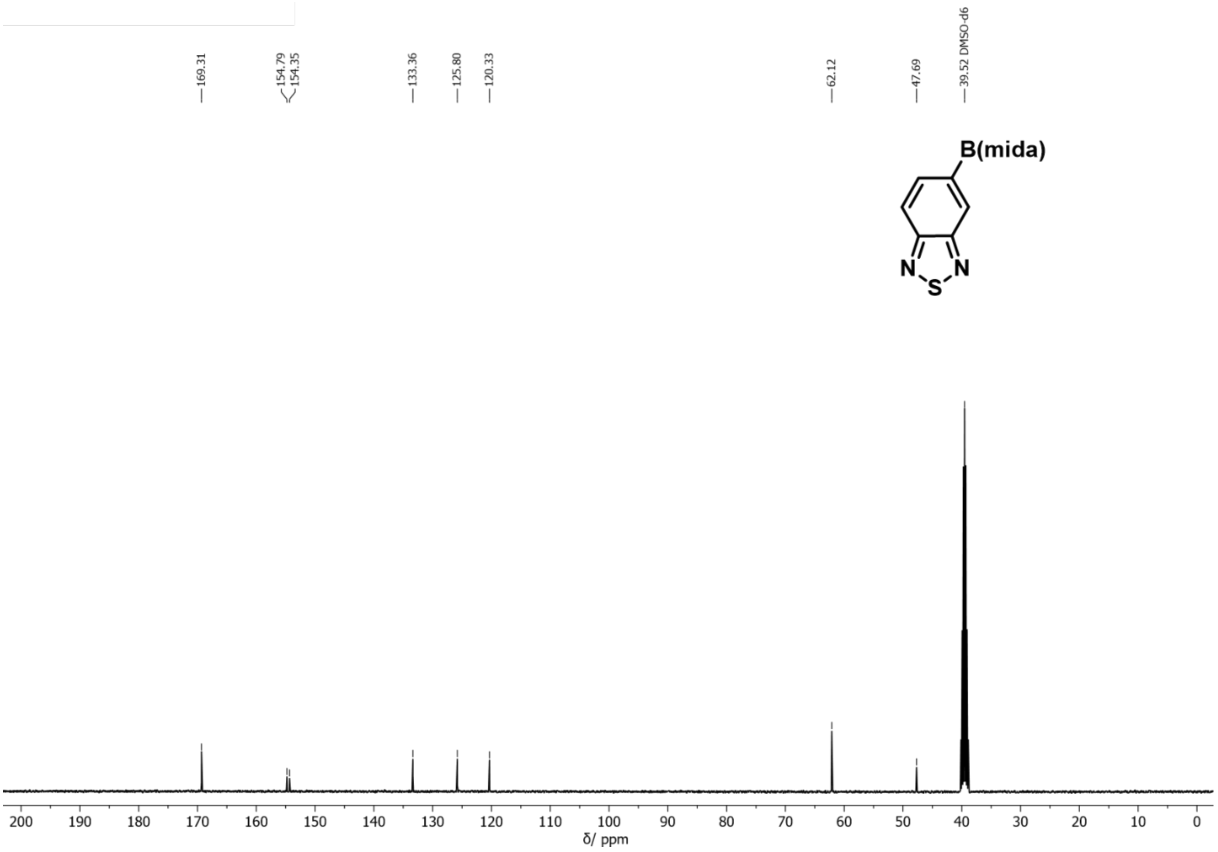

*Figure S17: $^{13}C\{^1H\}$ NMR spectrum of **3g** in DMSO-d_6 (101 MHz).*

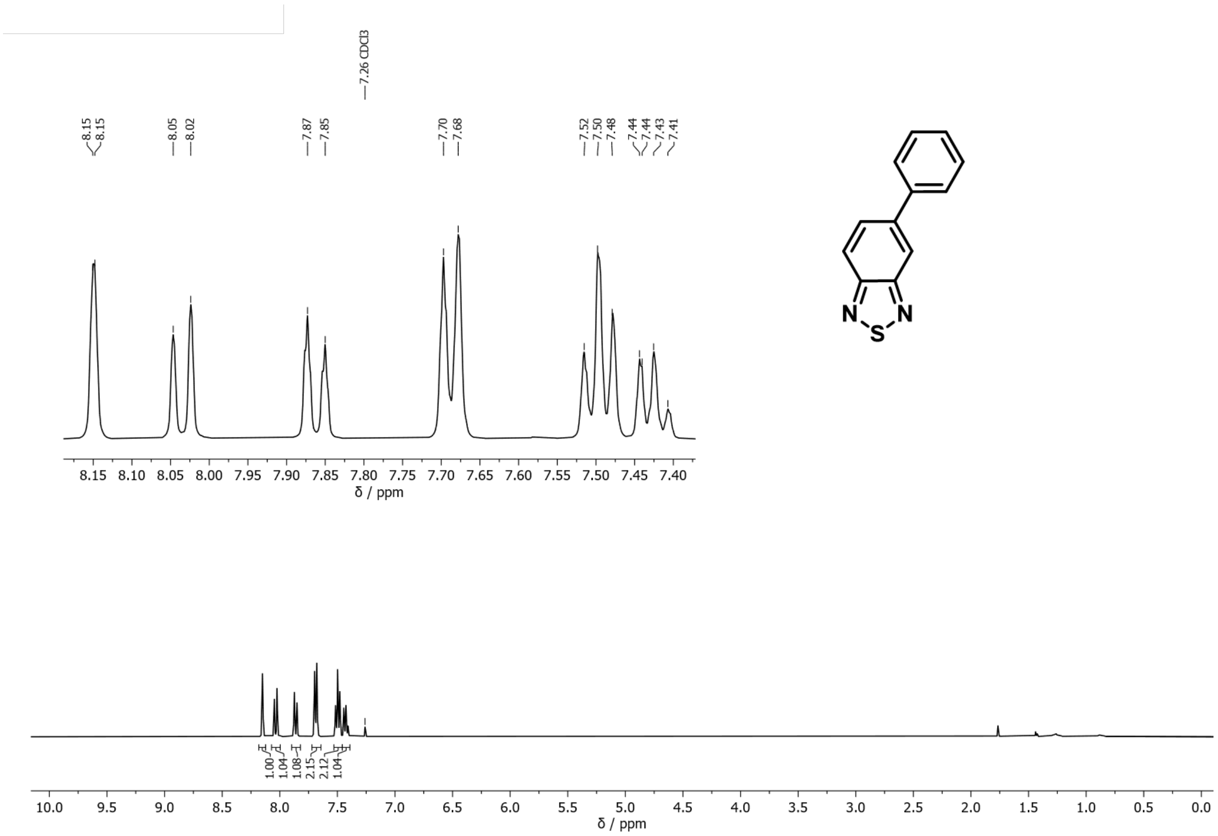

*Figure S18: ^{1}H NMR spectrum of **4a** in $CDCl_3$ (400 MHz).*

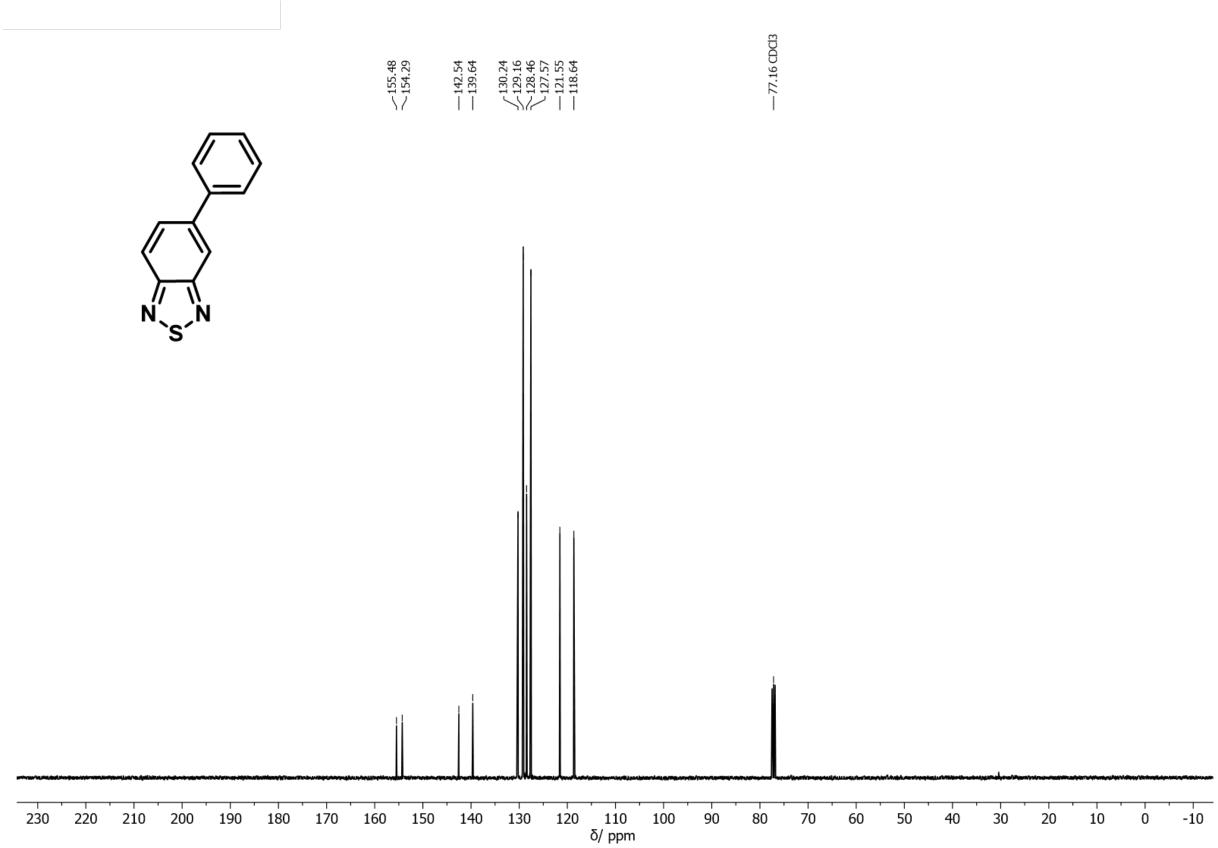

*Figure S19: $^{13}C\{^{1}H\}$ NMR spectrum of **4a** in $CDCl_3$ (101 MHz).*

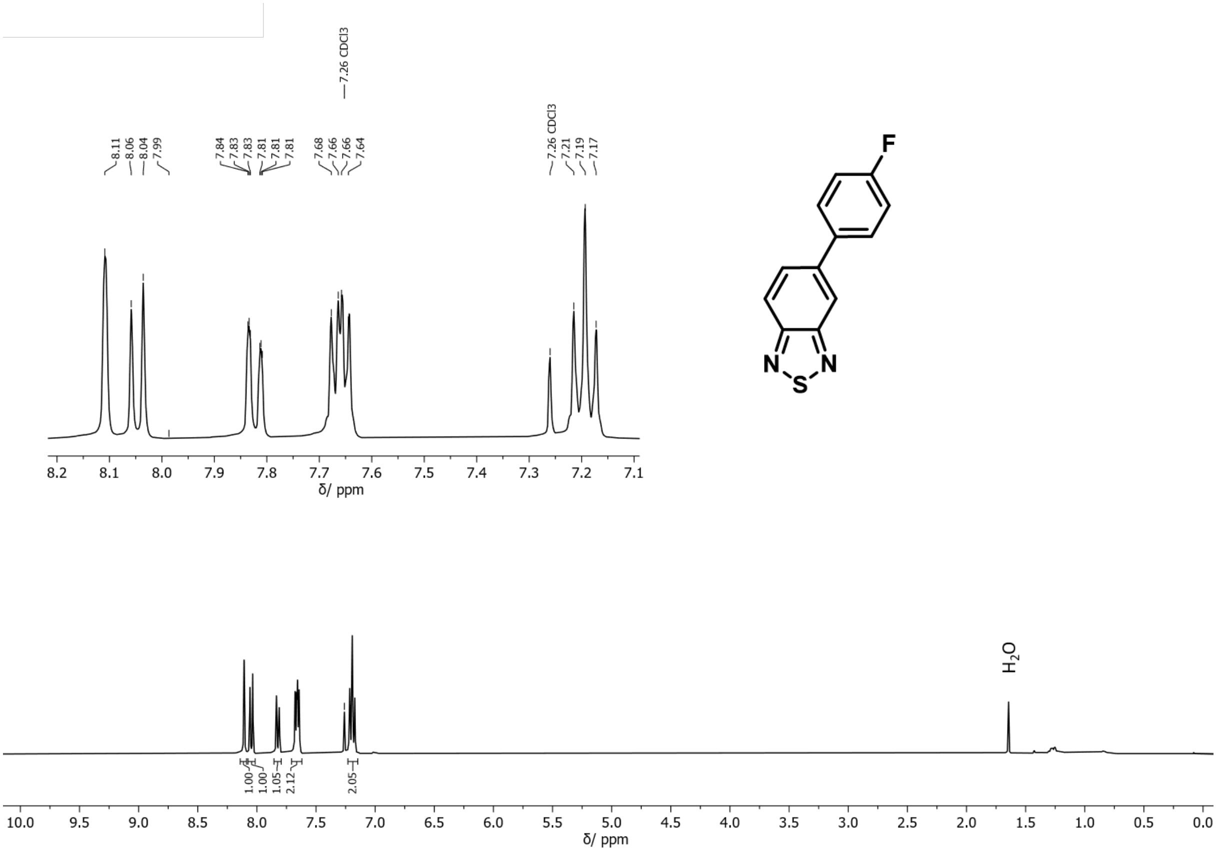

*Figure S20: ^{1}H NMR spectrum of **4b** in $CDCl_3$ (400 MHz).*

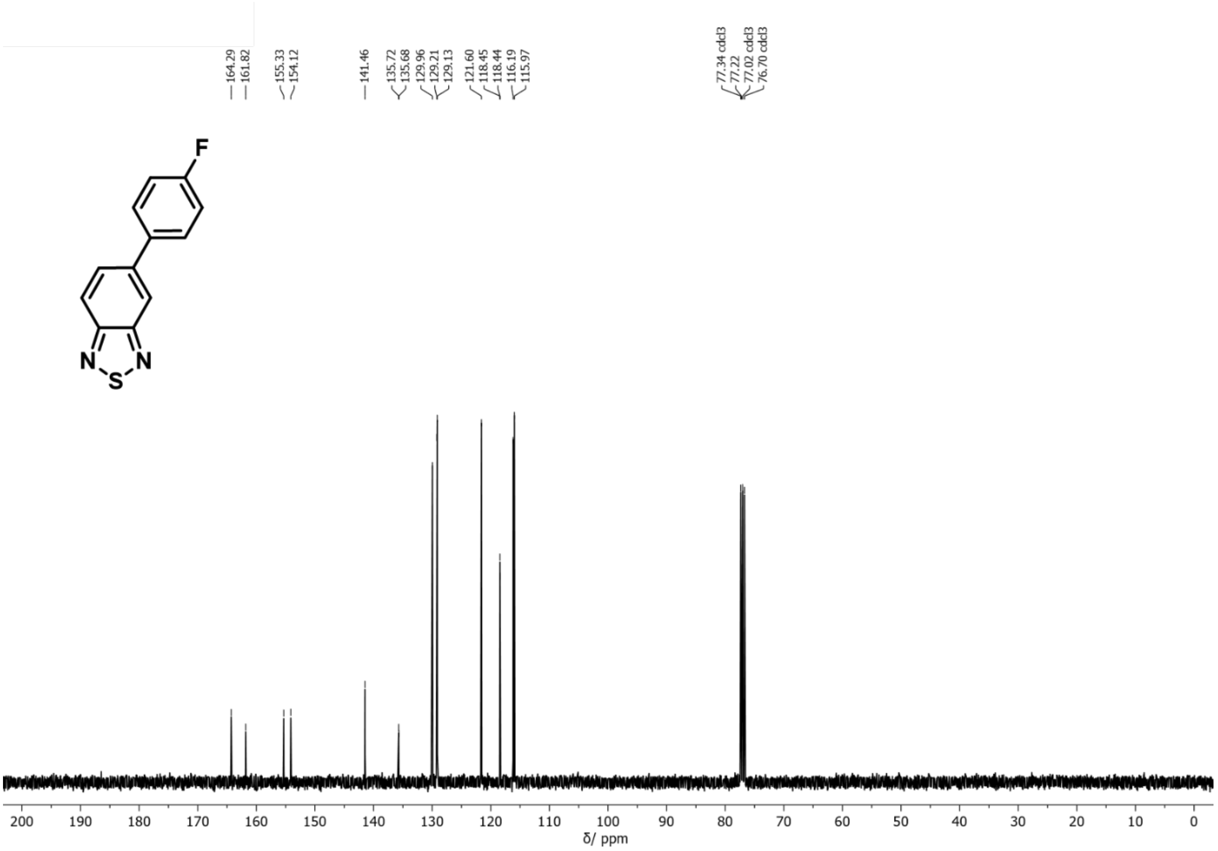

*Figure S21: $^{13}C\{^{1}H\}$ NMR spectrum of **4b** in $CDCl_3$ (101 MHz).*

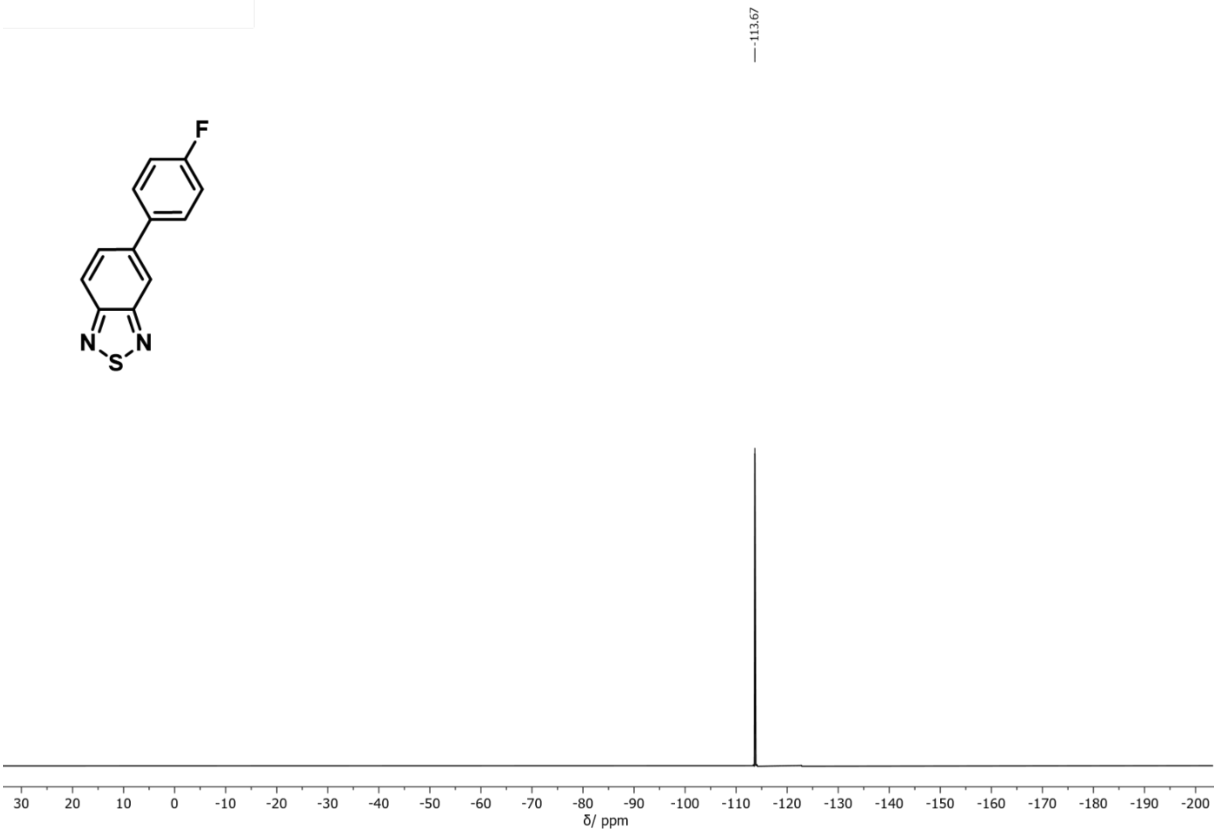

*Figure S22: ^{19}F NMR spectrum of **4b** in $CDCl_3$ (376 MHz).*

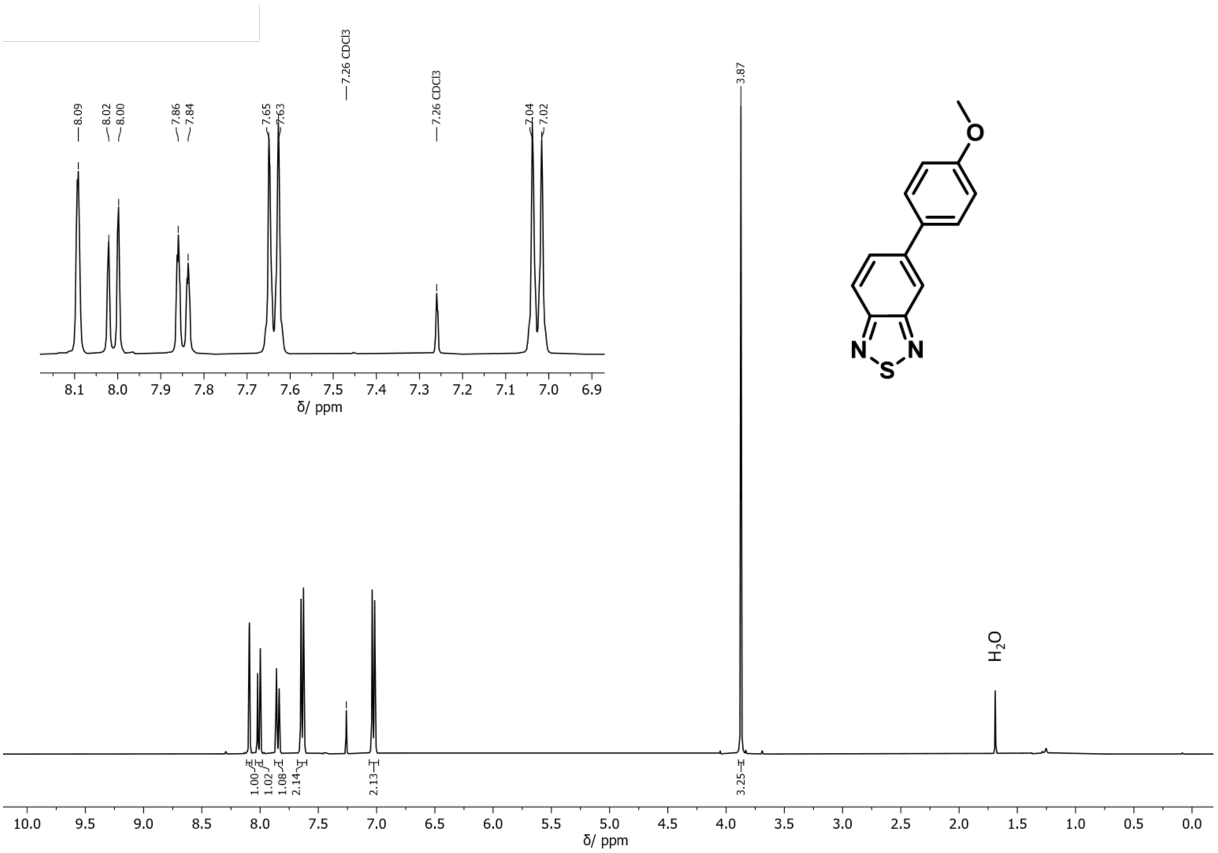

*Figure S23: ^{1}H NMR spectrum of **4c** in $CDCl_3$ (400 MHz).*

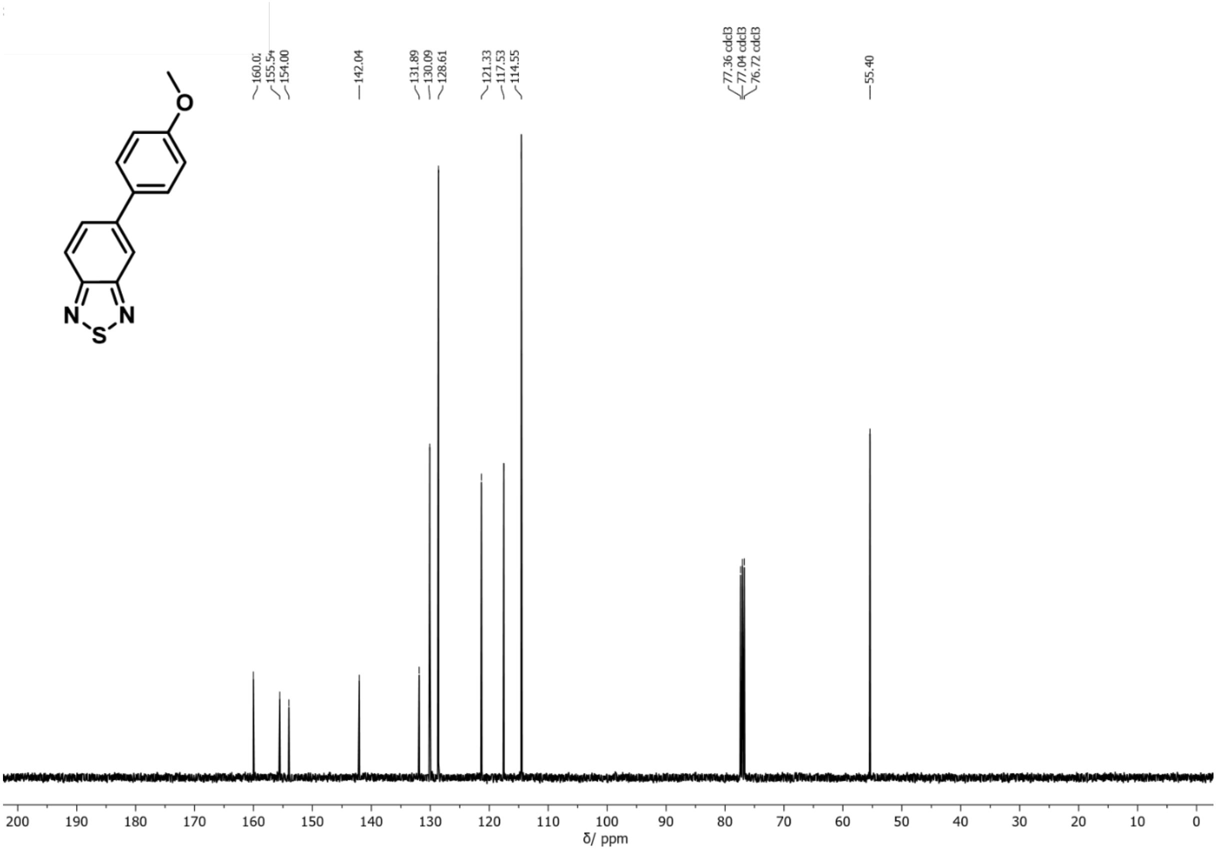

*Figure S24: $^{13}C\{^{1}H\}$ NMR spectrum of **4c** in $CDCl_3$ (101 MHz).*

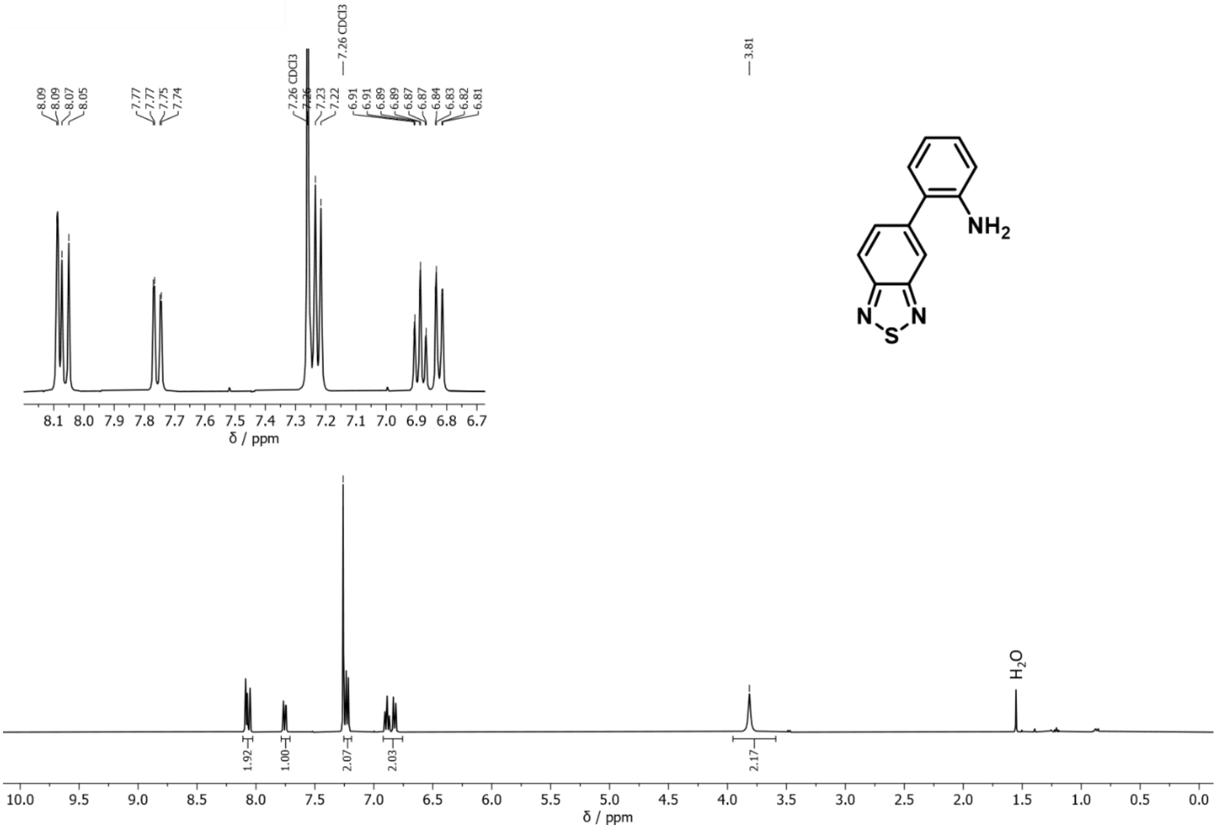

*Figure S25: ^{1}H NMR spectrum of **4d** in $CDCl_3$ (400 MHz).*

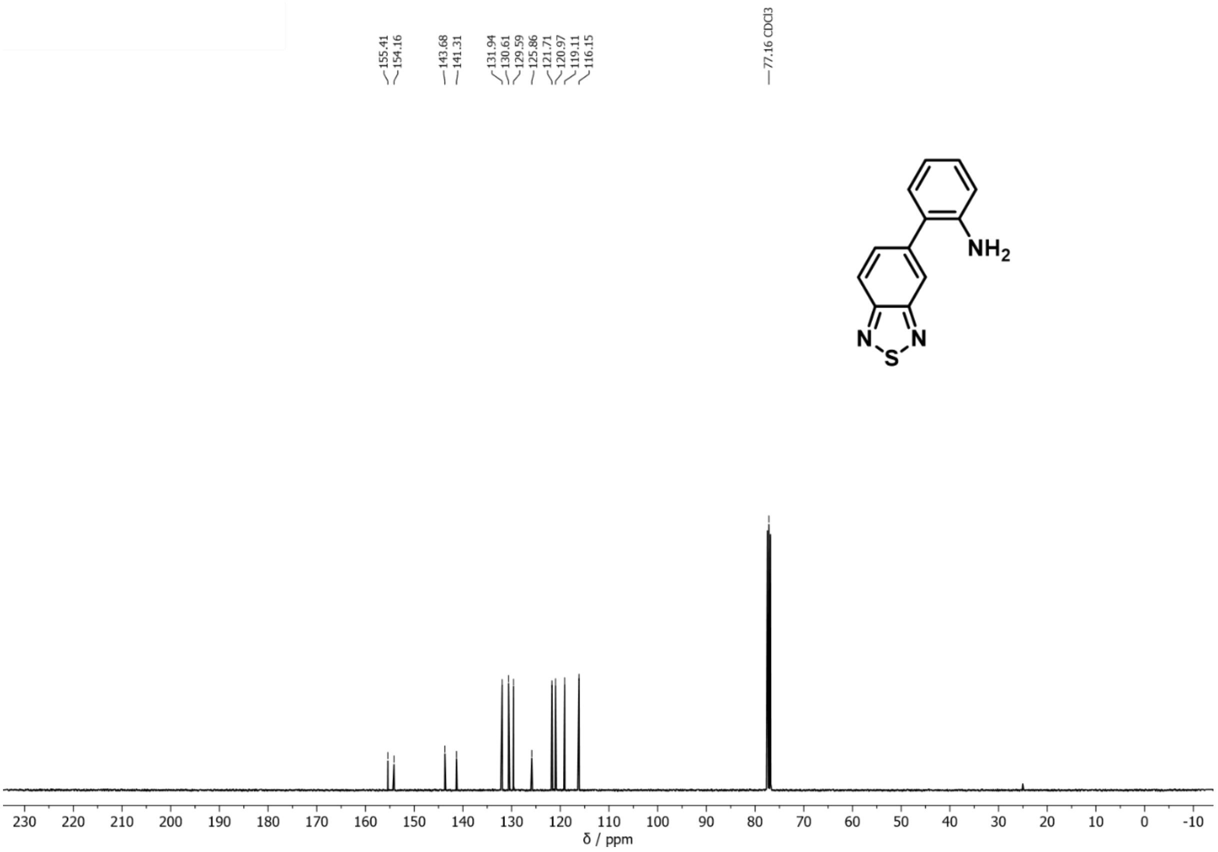

*Figure S1: $^{13}C\{^{1}H\}$ NMR spectrum of **4d** in $CDCl_3$ (101 MHz).*

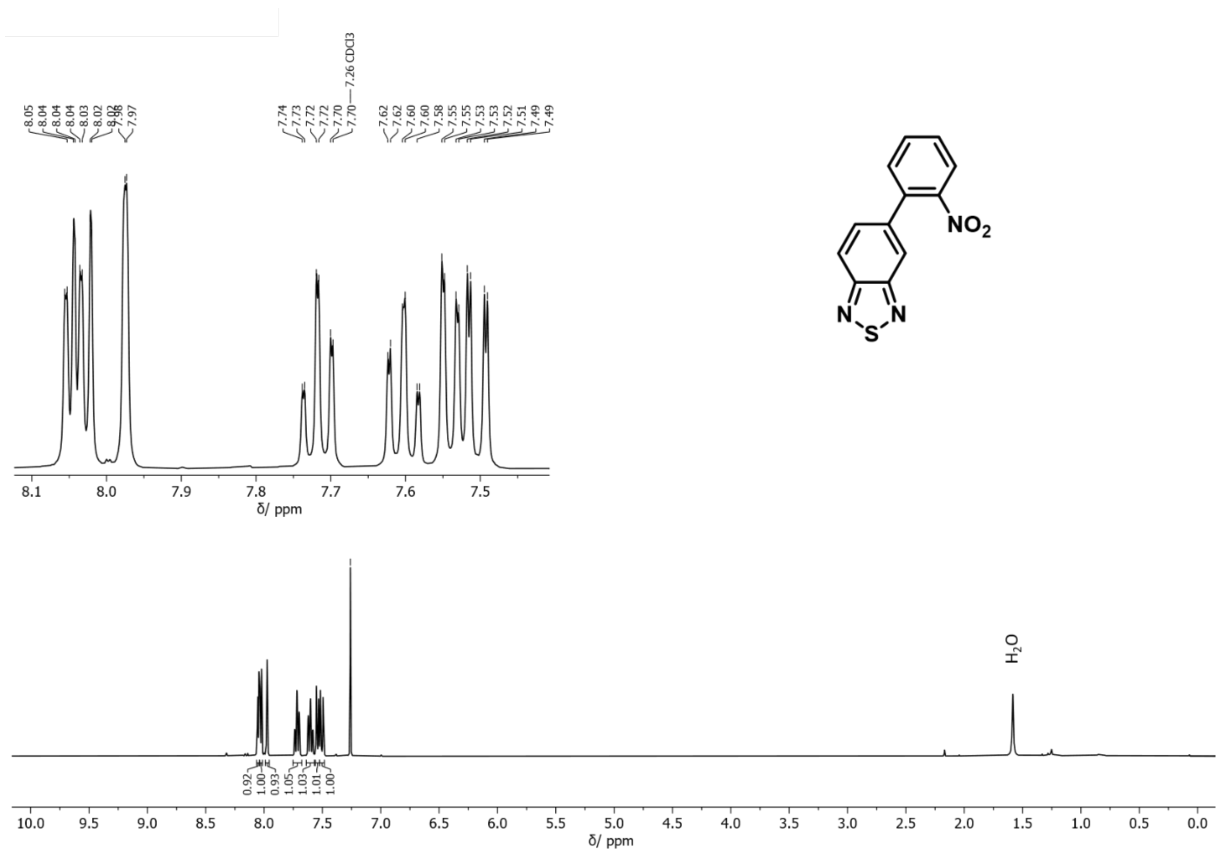

*Figure S2: ^{1}H NMR spectrum of **4e** in $CDCl_3$ (400 MHz).*

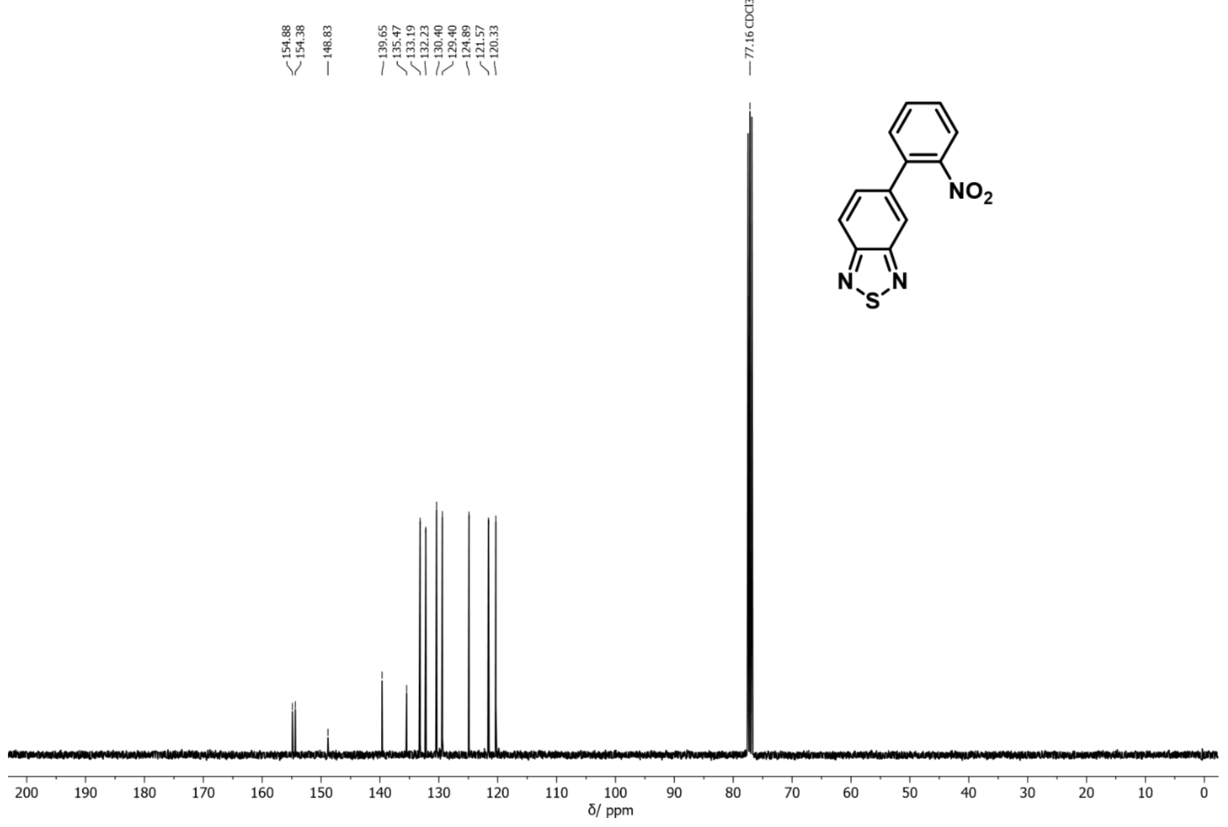

*Figure S3: ^{13}C{^{1}H} NMR spectrum of **4e** in $CDCl_3$ (101 MHz).*

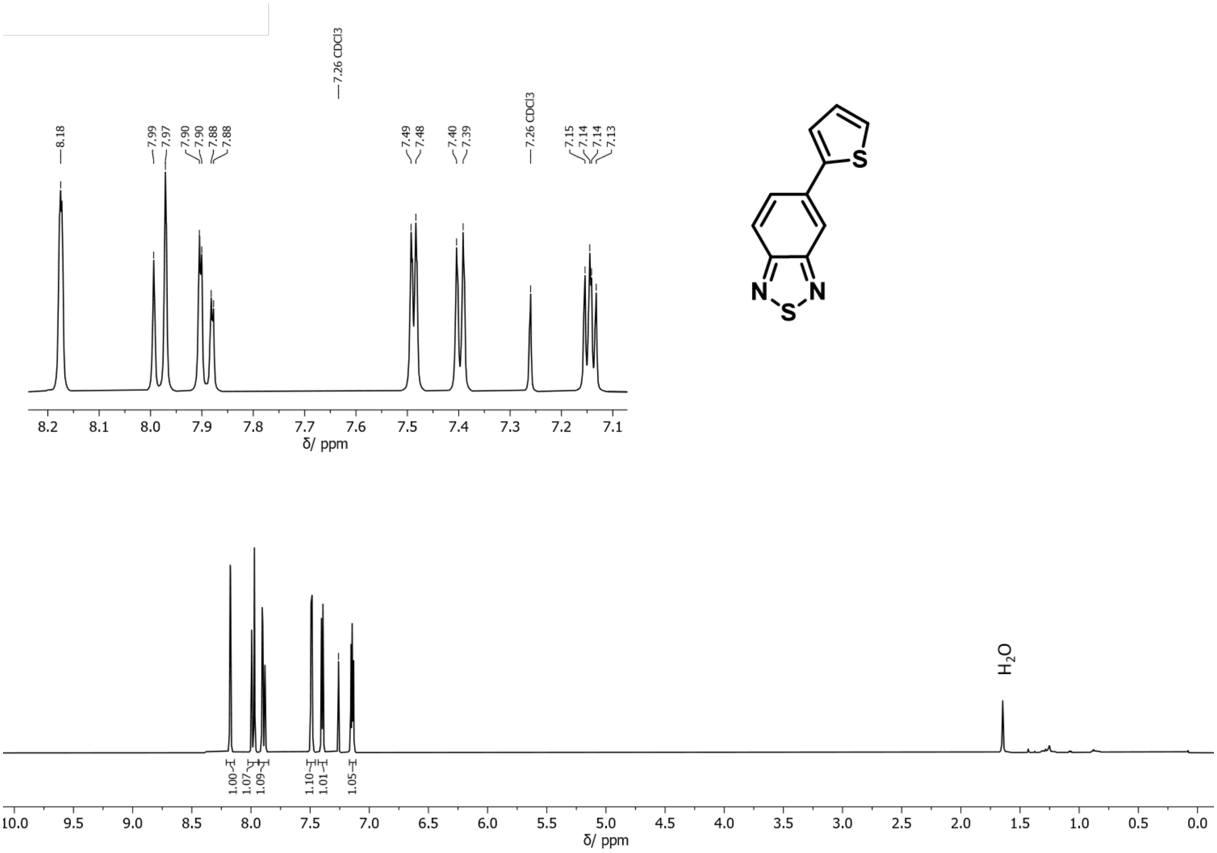

*Figure S4: ^{1}H NMR spectrum of **4f** in $CDCl_3$ (400 MHz).*

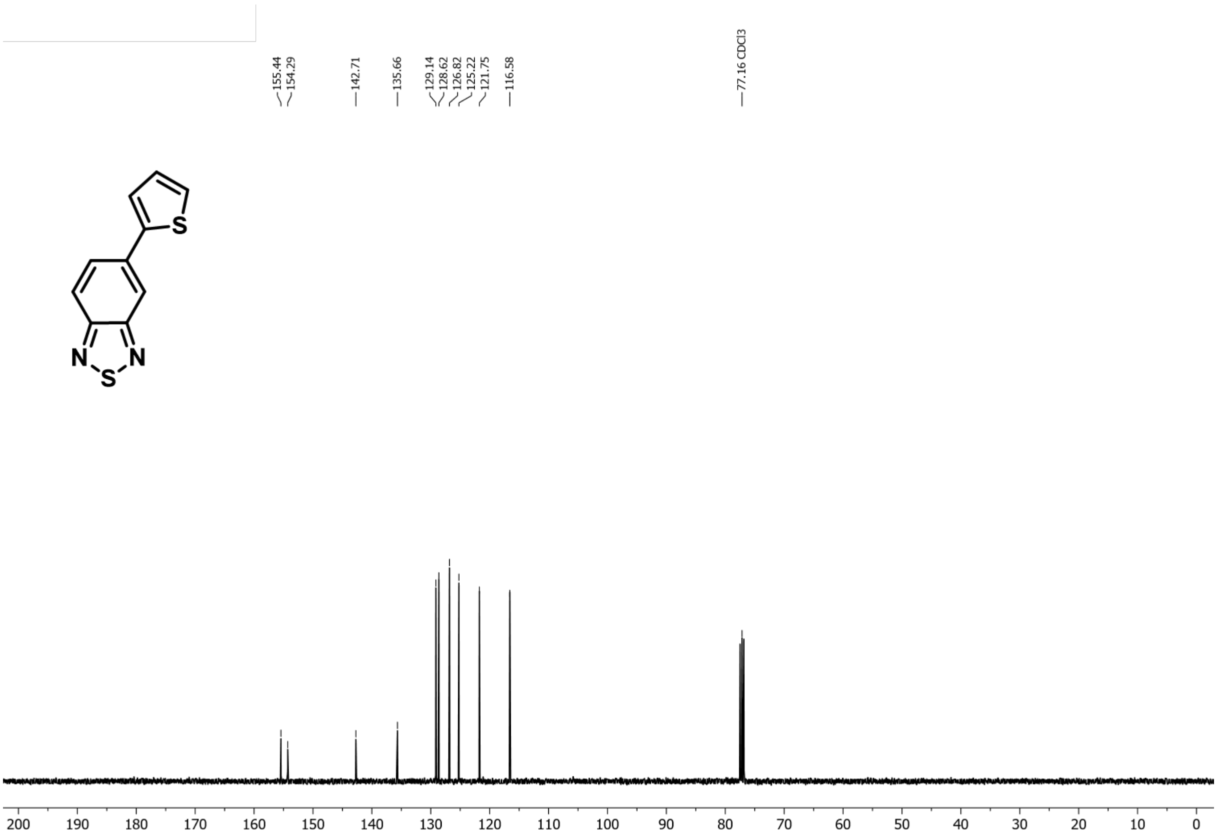

*Figure S30: $^{13}C\{^{1}H\}$ NMR spectrum of **4f** in $CDCl_3$ (101 MHz).*

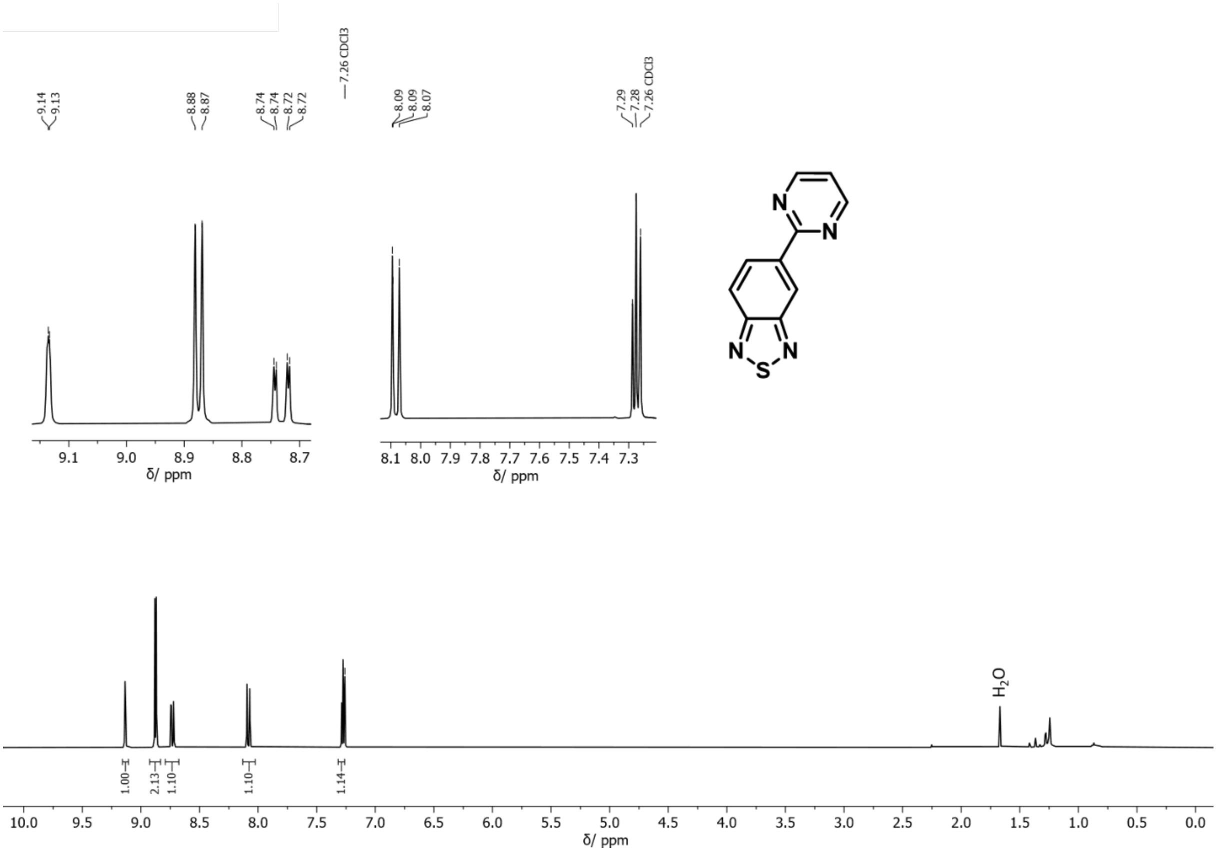

*Figure S31: ^{1}H NMR spectrum of **4g** in $CDCl_3$ (400 MHz).*

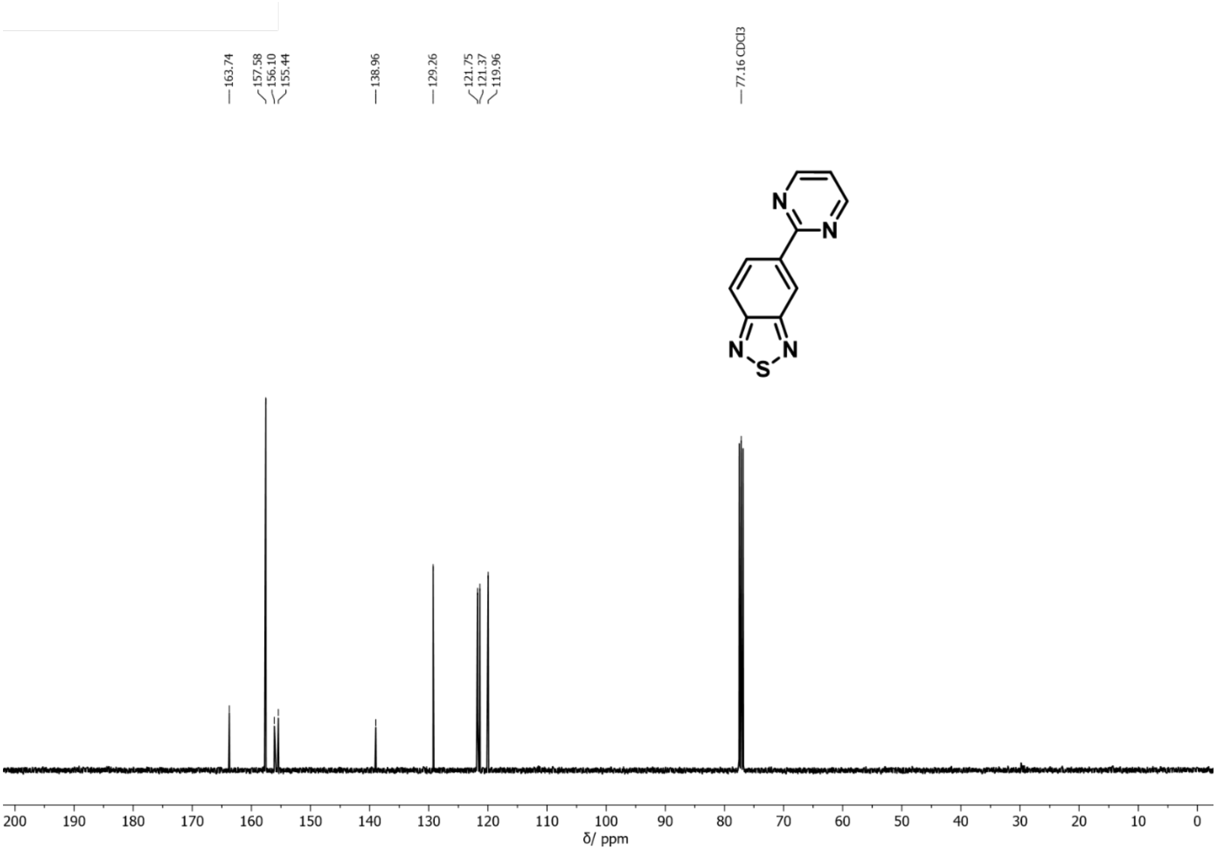

*Figure S32: $^{13}C\{^{1}H\}$ NMR spectrum of **4g** in $CDCl_3$ (101 MHz).*

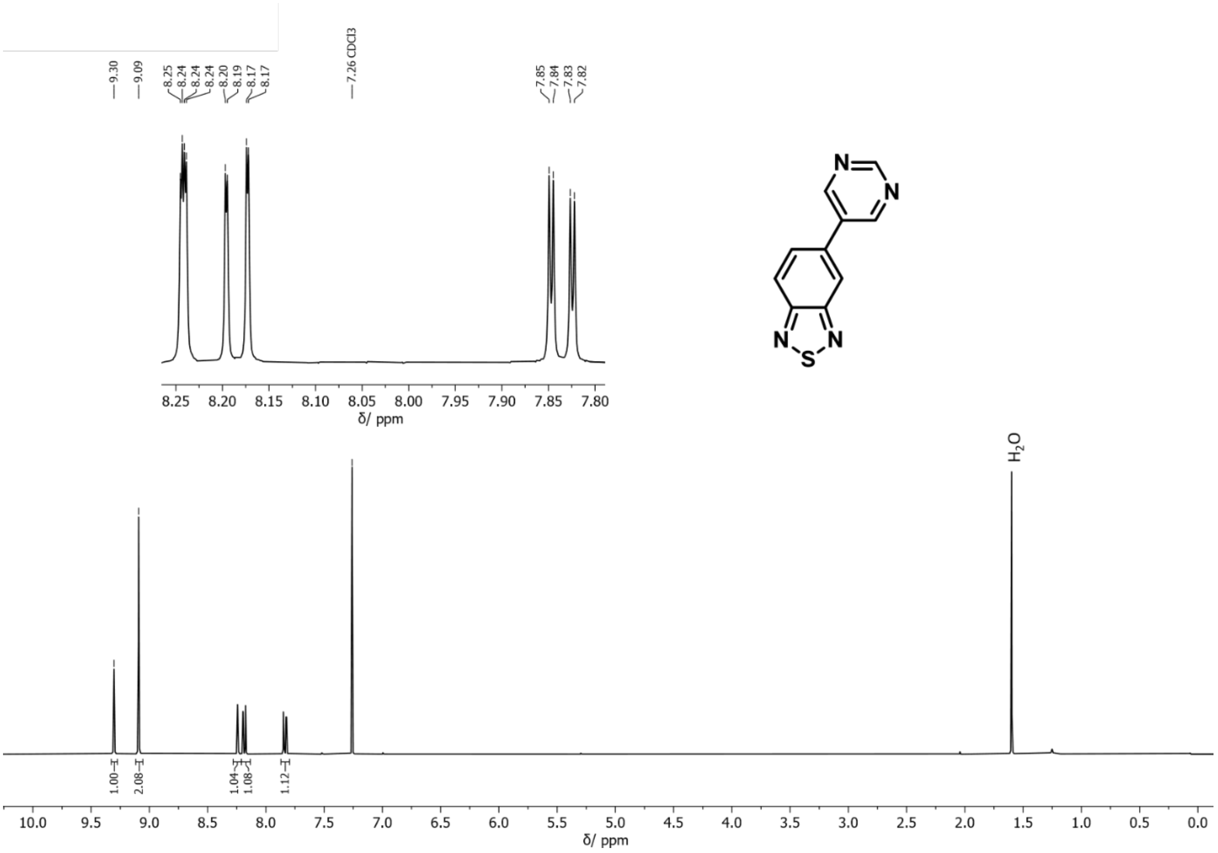

*Figure S33: ^{1}H NMR spectrum of **4h** in $CDCl_3$ (400 MHz).*

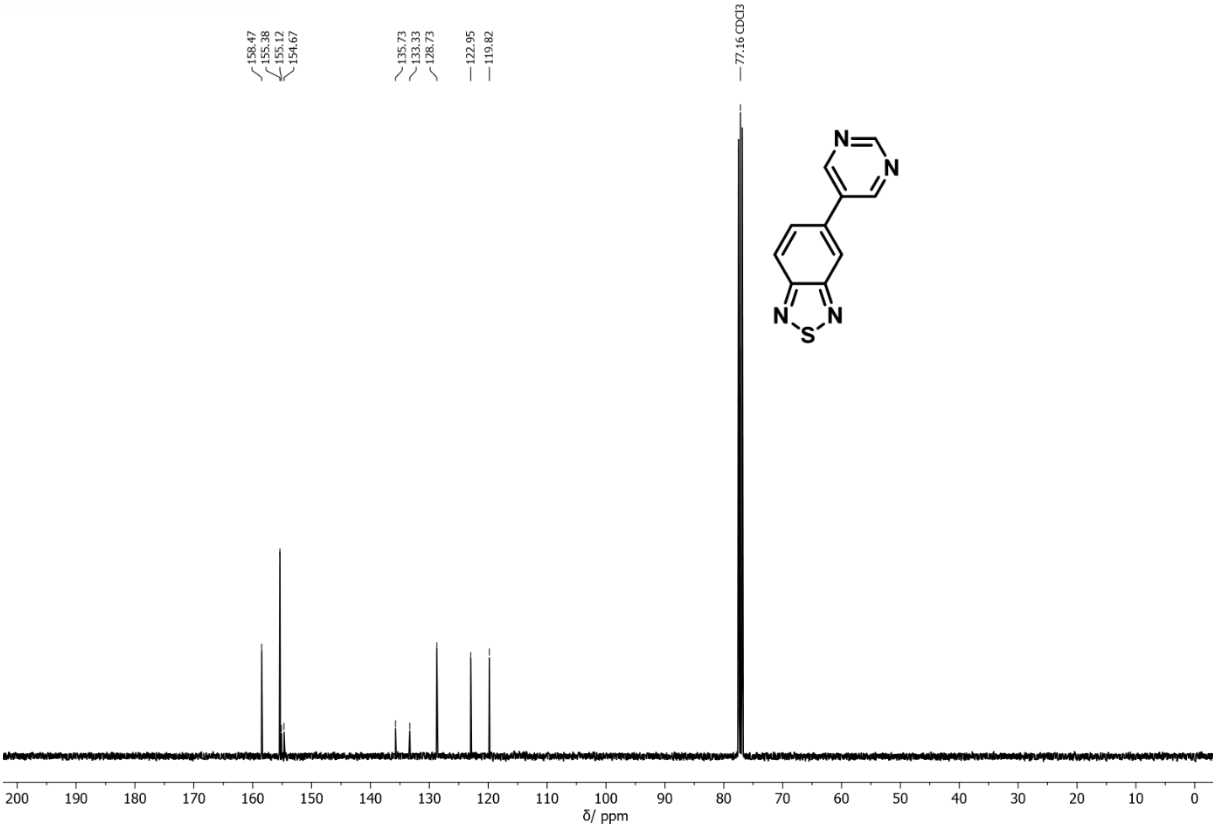

*Figure S5: $^{13}C\{^{1}H\}$ NMR spectrum of **4h** in $CDCl_3$ (101 MHz).*

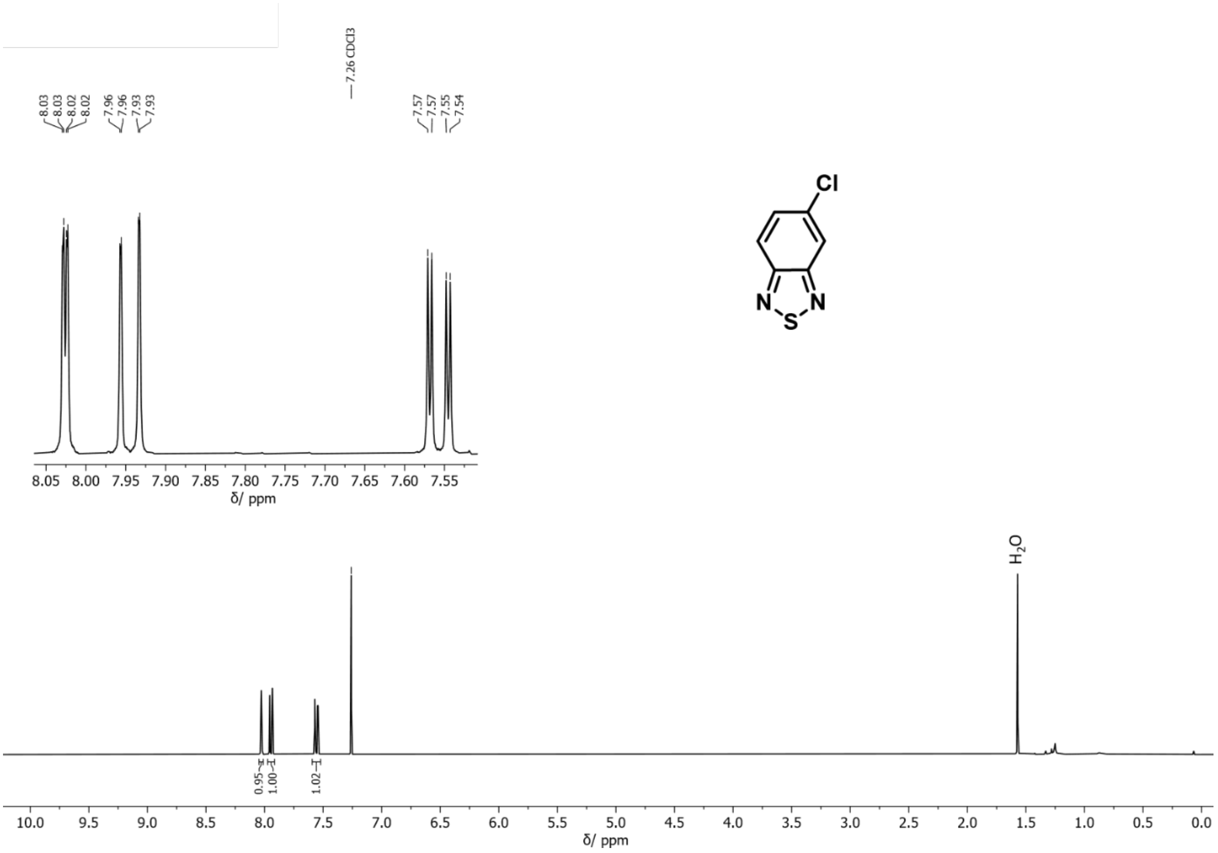

*Figure S35: ^{1}H NMR spectrum of **5a** in $CDCl_3$ (400 MHz).*

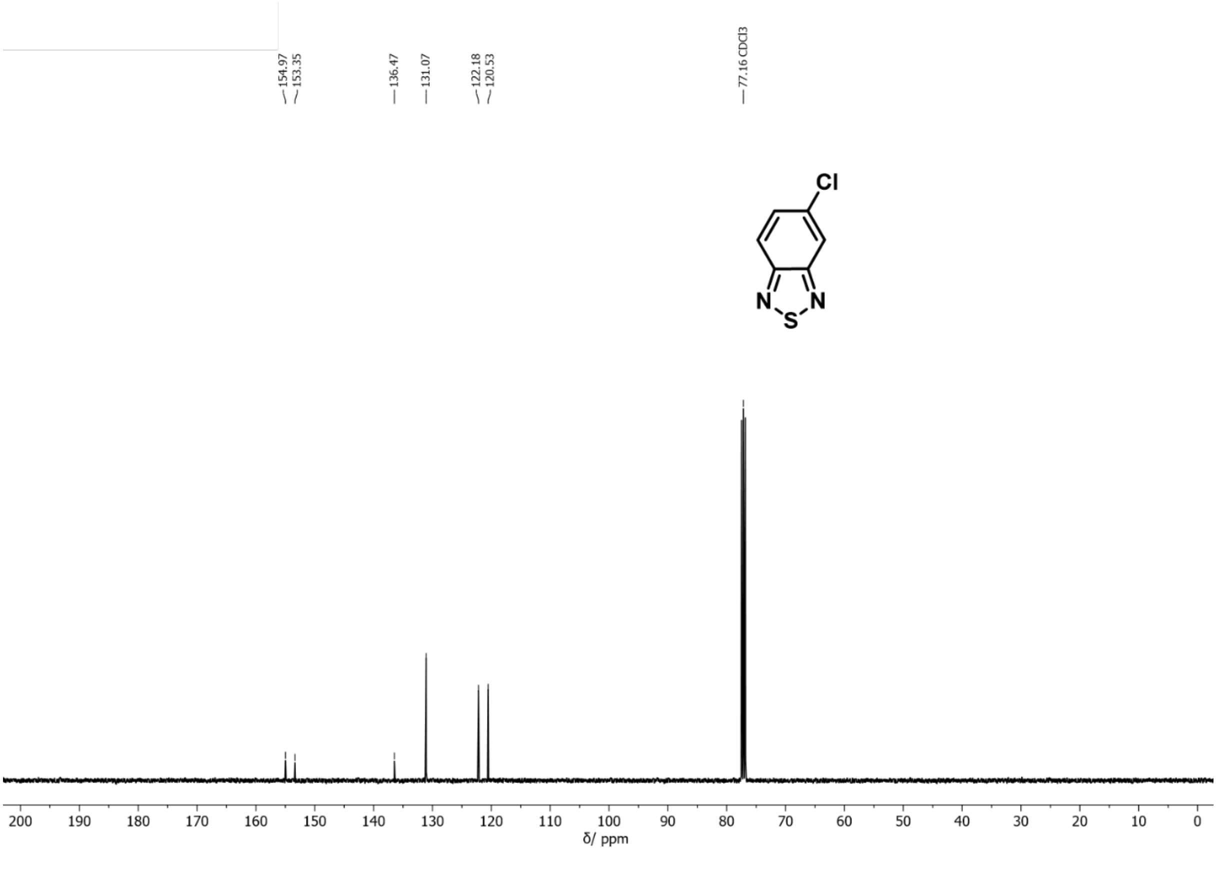

*Figure S6: $^{13}C\{^{1}H\}$ NMR spectrum of **5a** in $CDCl_3$ (101 MHz).*

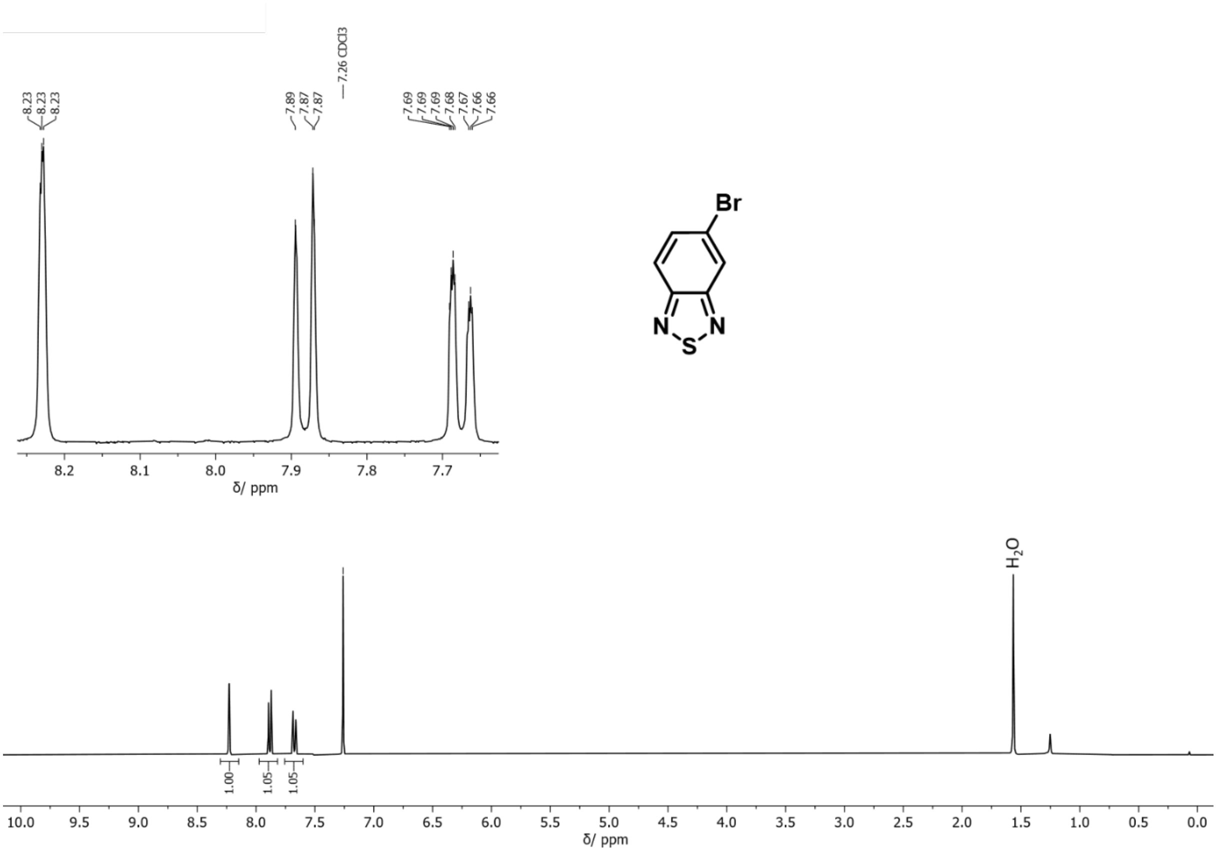

*Figure S7: ^{1}H NMR spectrum of **5b** in $CDCl_3$ (400 MHz).*

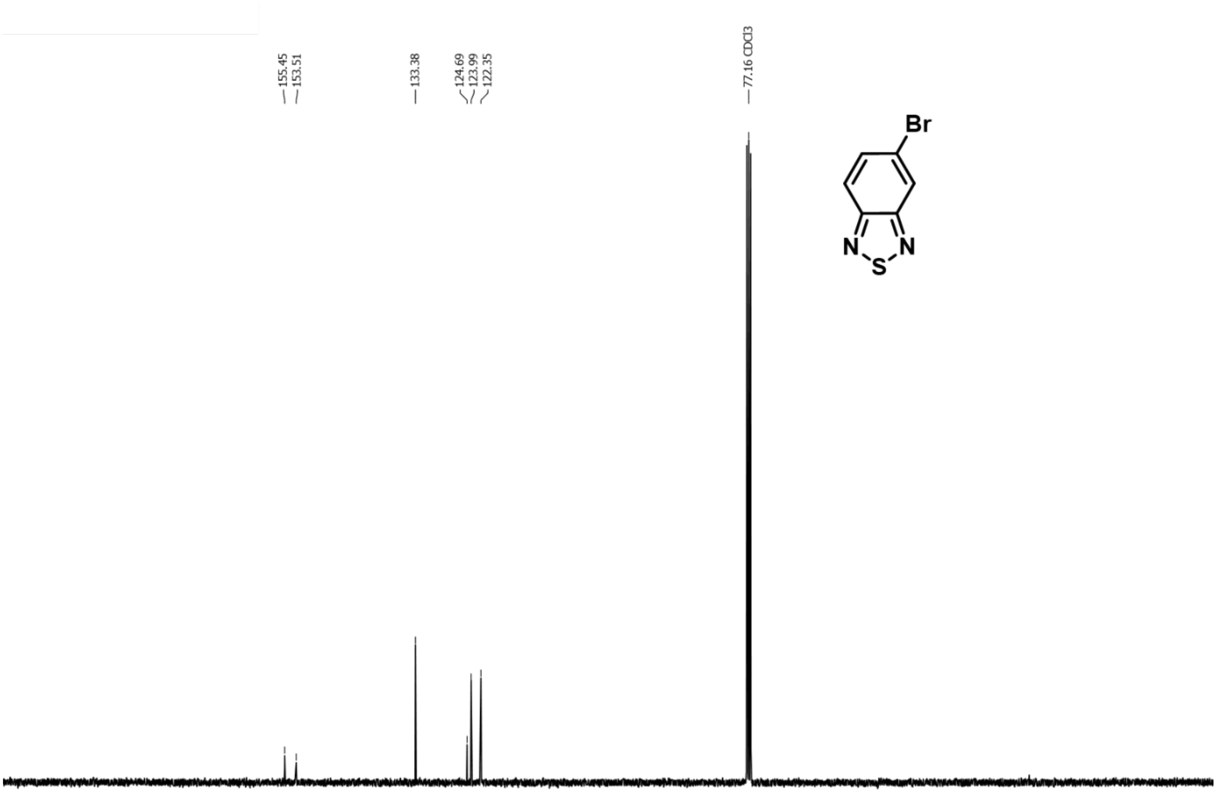

*Figure S8: $^{13}C\{^{1}H\}$ NMR spectrum of **5b** in $CDCl_3$ (101 MHz).*

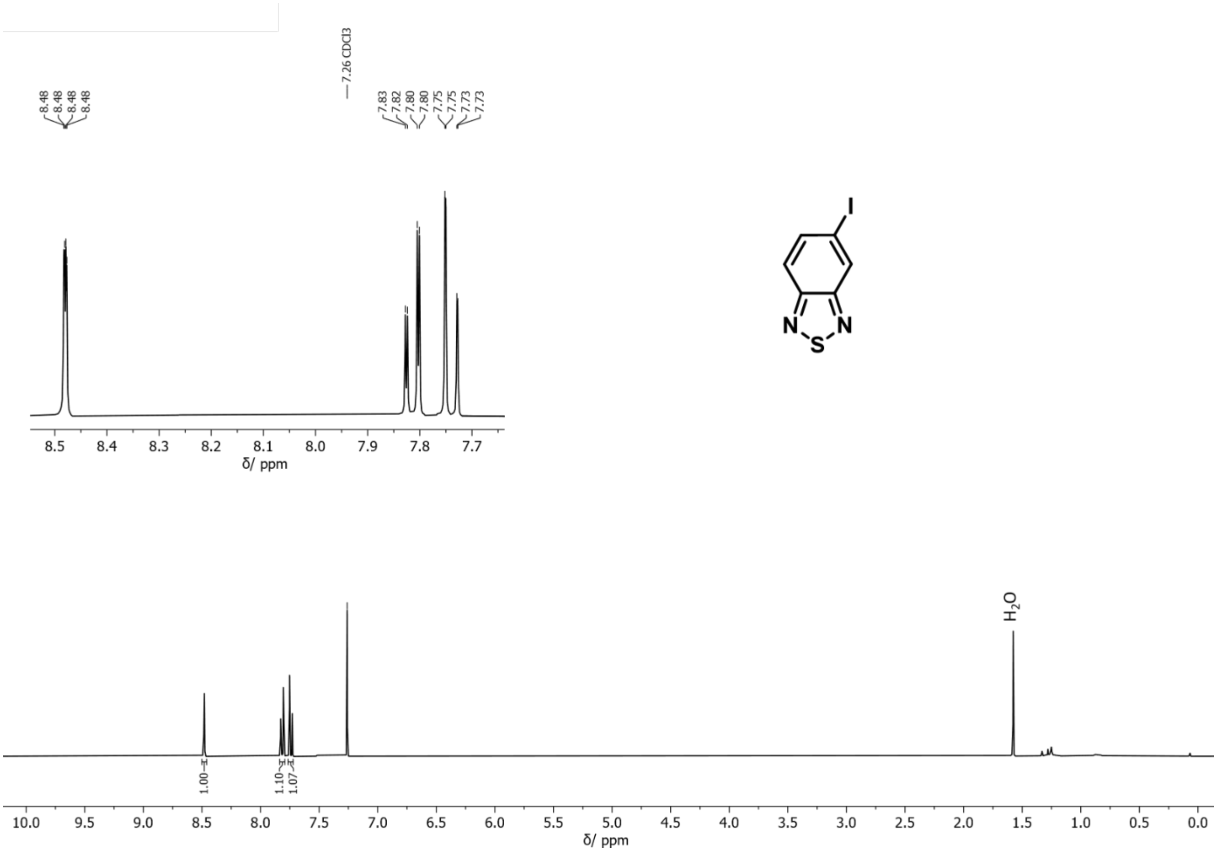

*Figure S39: ^{1}H NMR spectrum of **5c** in $CDCl_3$ (400 MHz).*

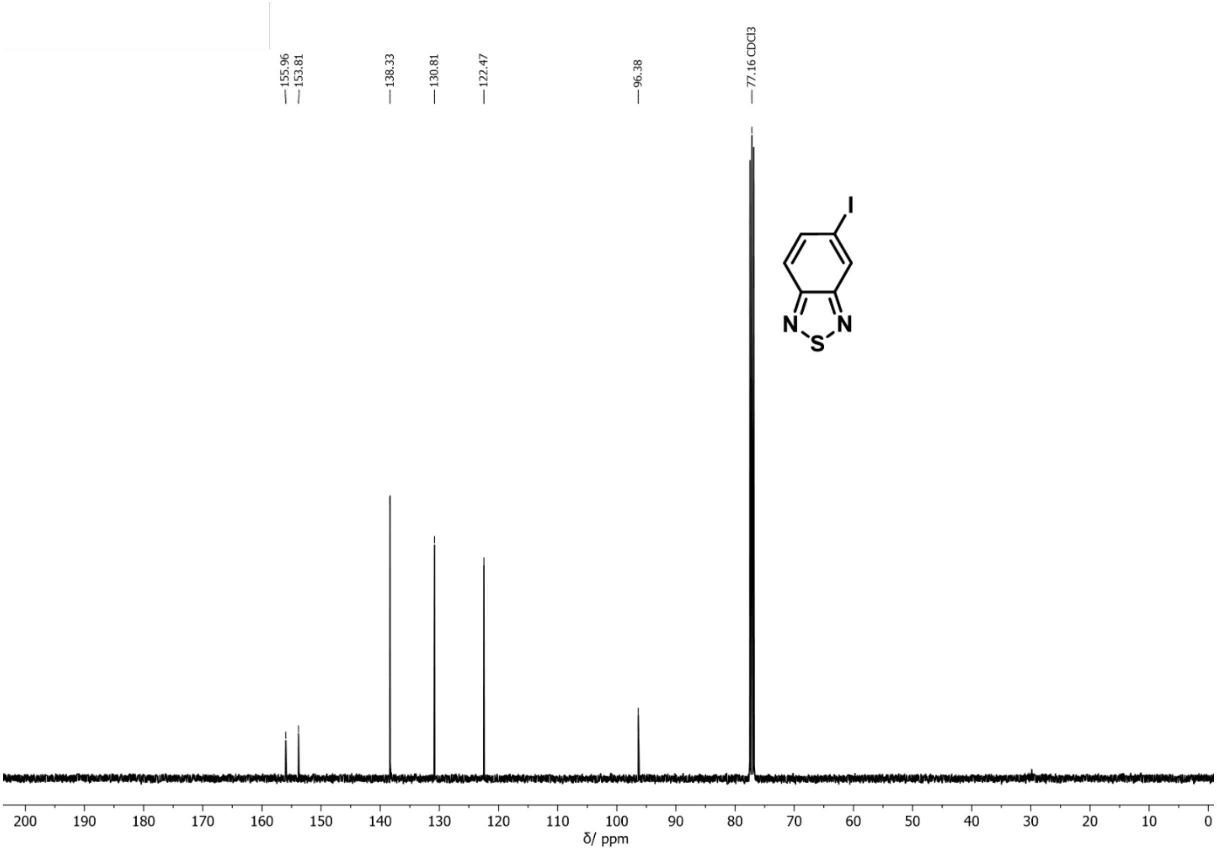

*Figure S40: $^{13}C\{^{1}H\}$ NMR spectrum of **5c** in $CDCl_3$ (101 MHz).*

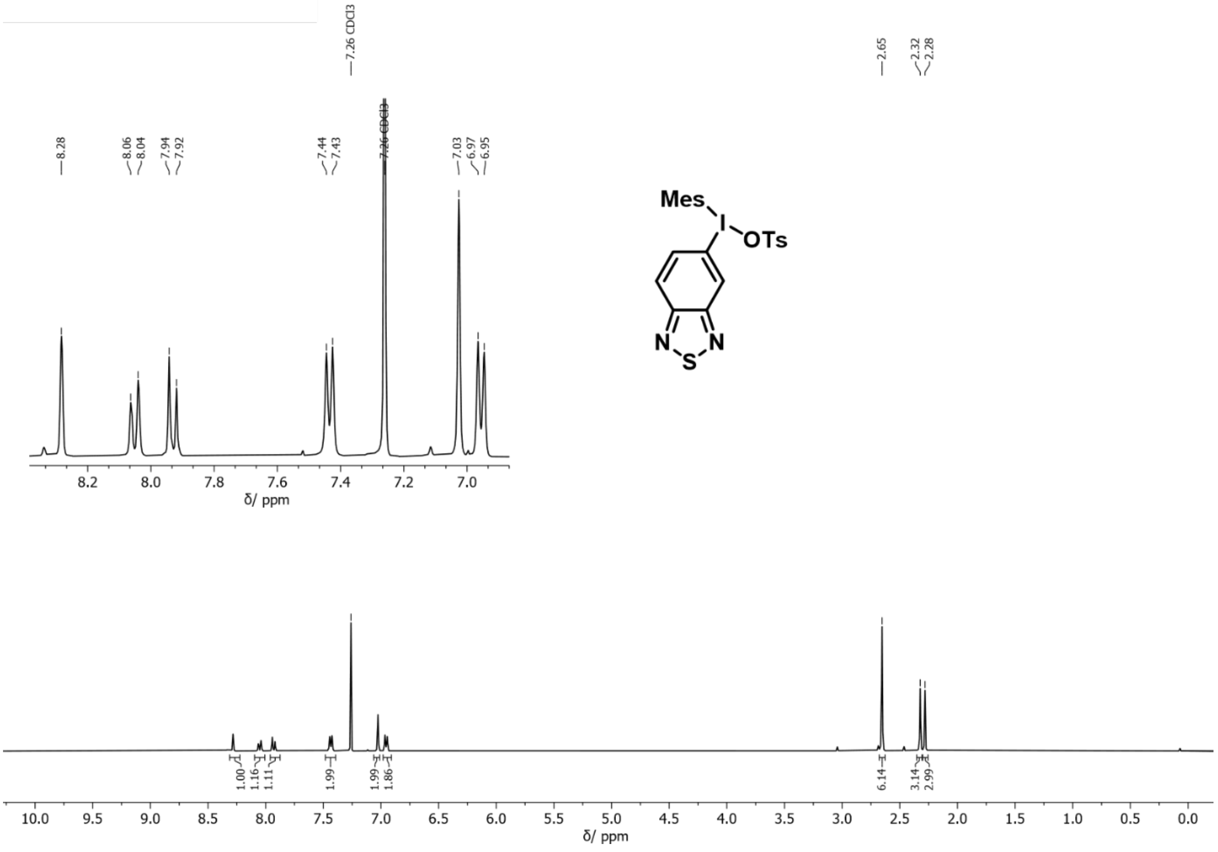

*Figure S41: ^{1}H NMR spectrum of **6** in $CDCl_3$ (400 MHz).*

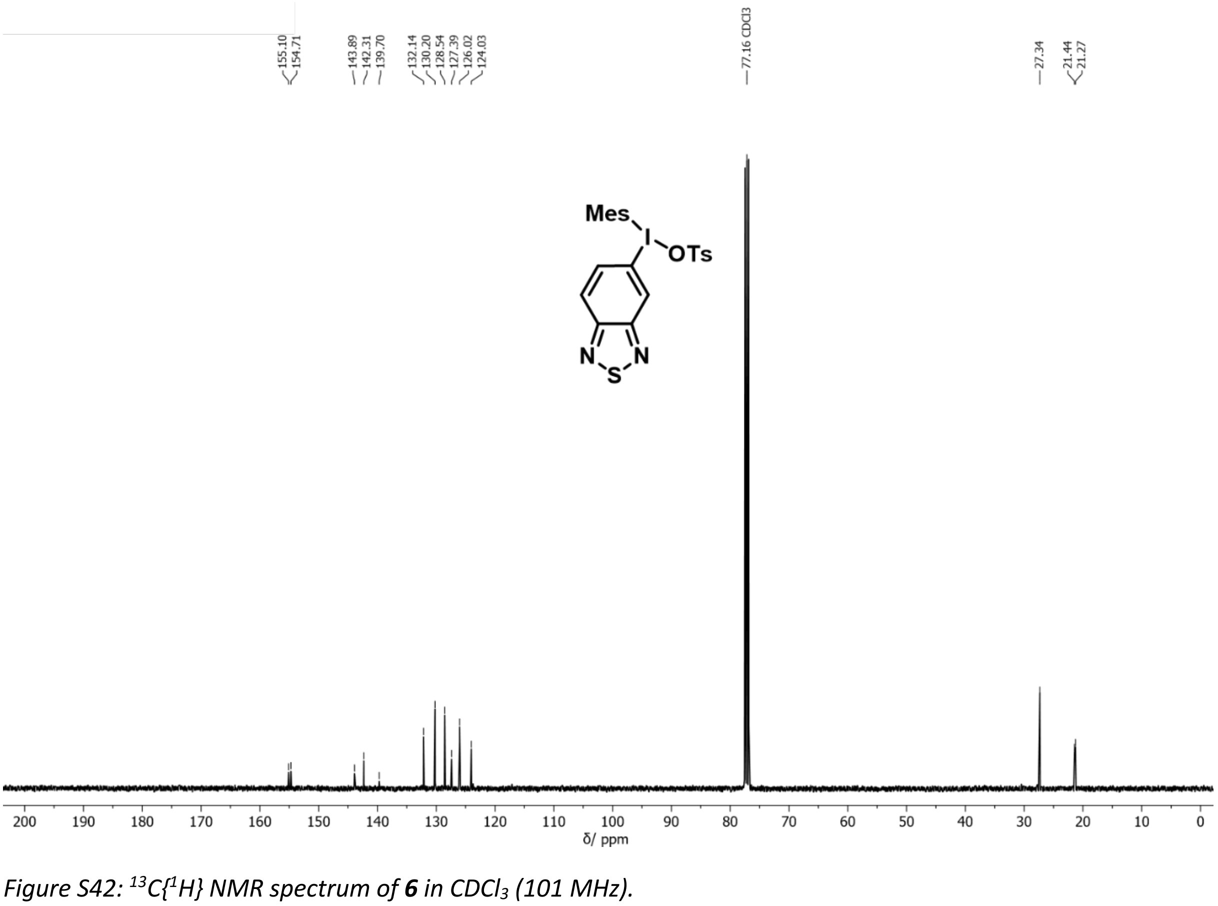

*Figure S42: $^{13}C\{^1H\}$ NMR spectrum of **6** in $CDCl_3$ (101 MHz).*

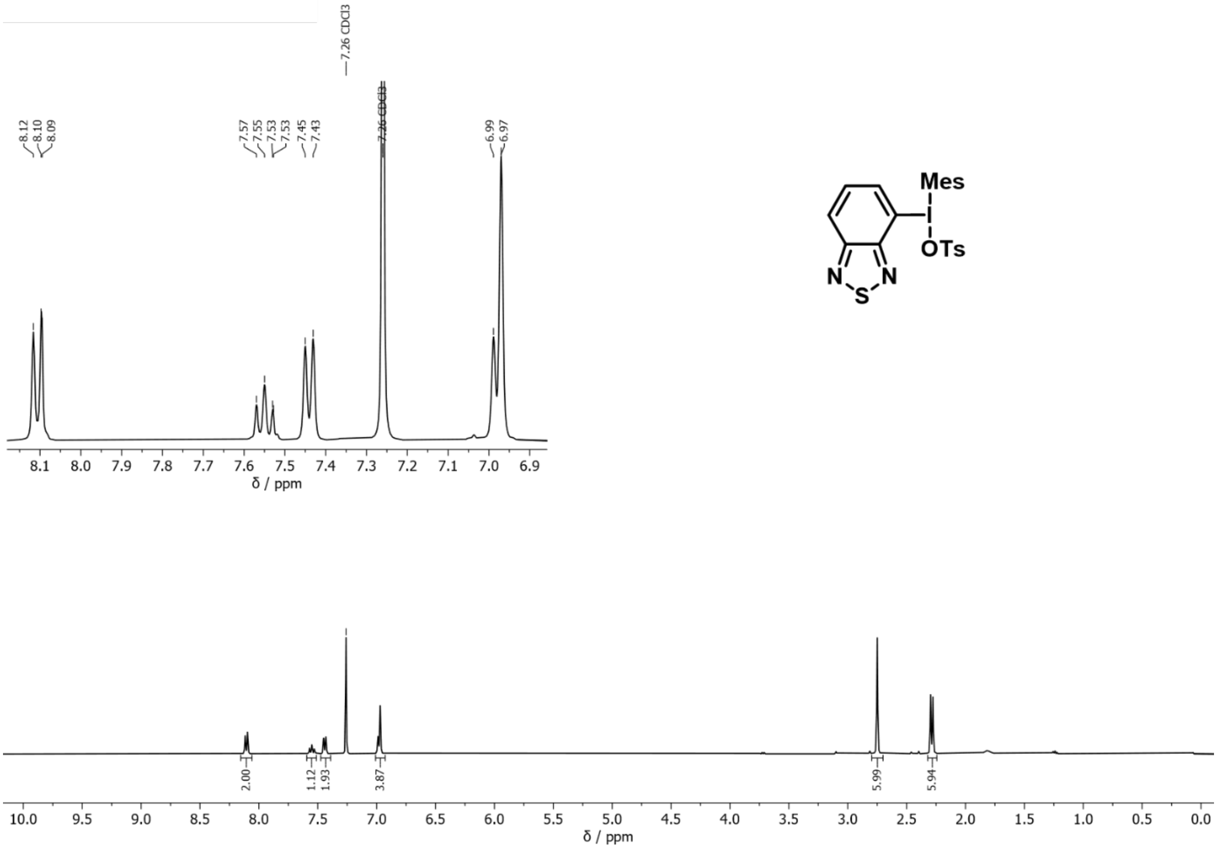

*Figure S43: ^{1}H NMR spectrum of **6’** in $CDCl_3$ (400 MHz).*

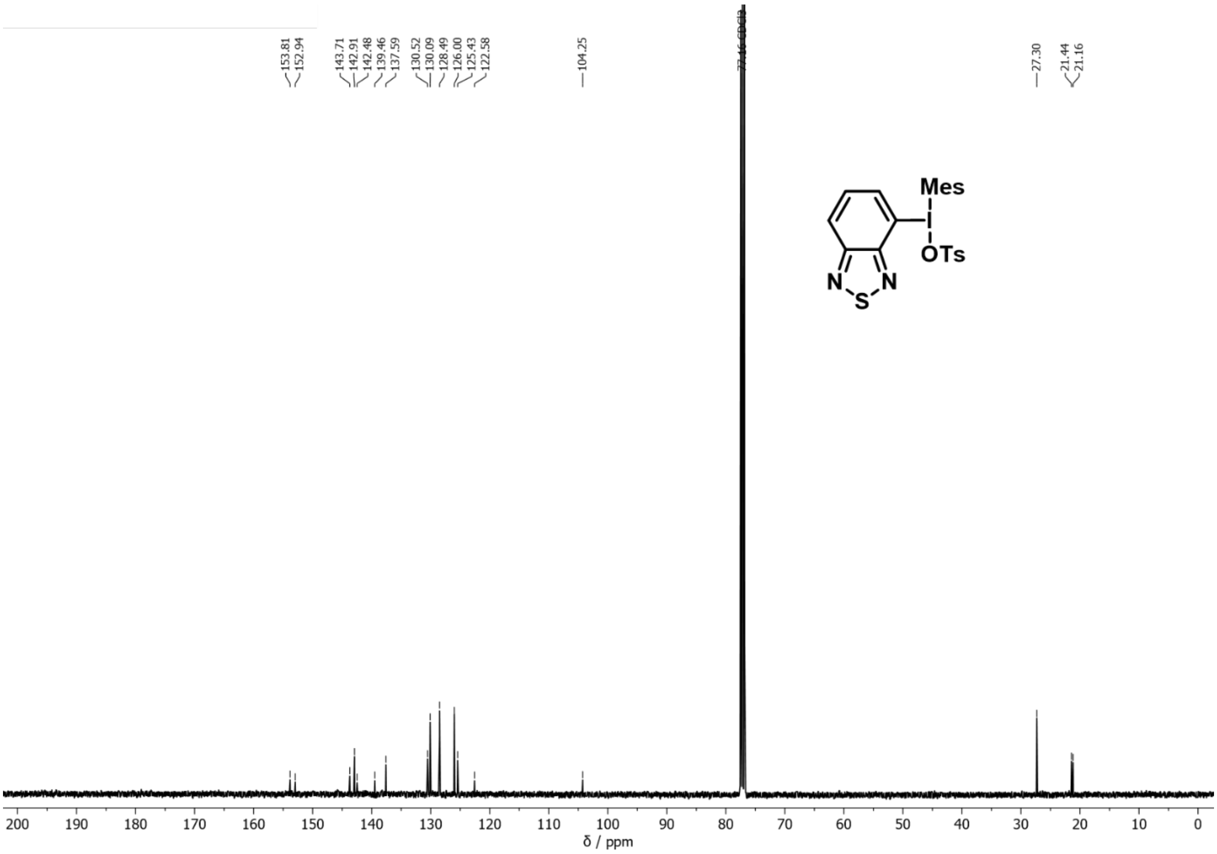

*Figure S44: $^{13}C\{^{1}H\}$ NMR spectrum of **6’** in $CDCl_3$ (101 MHz).*

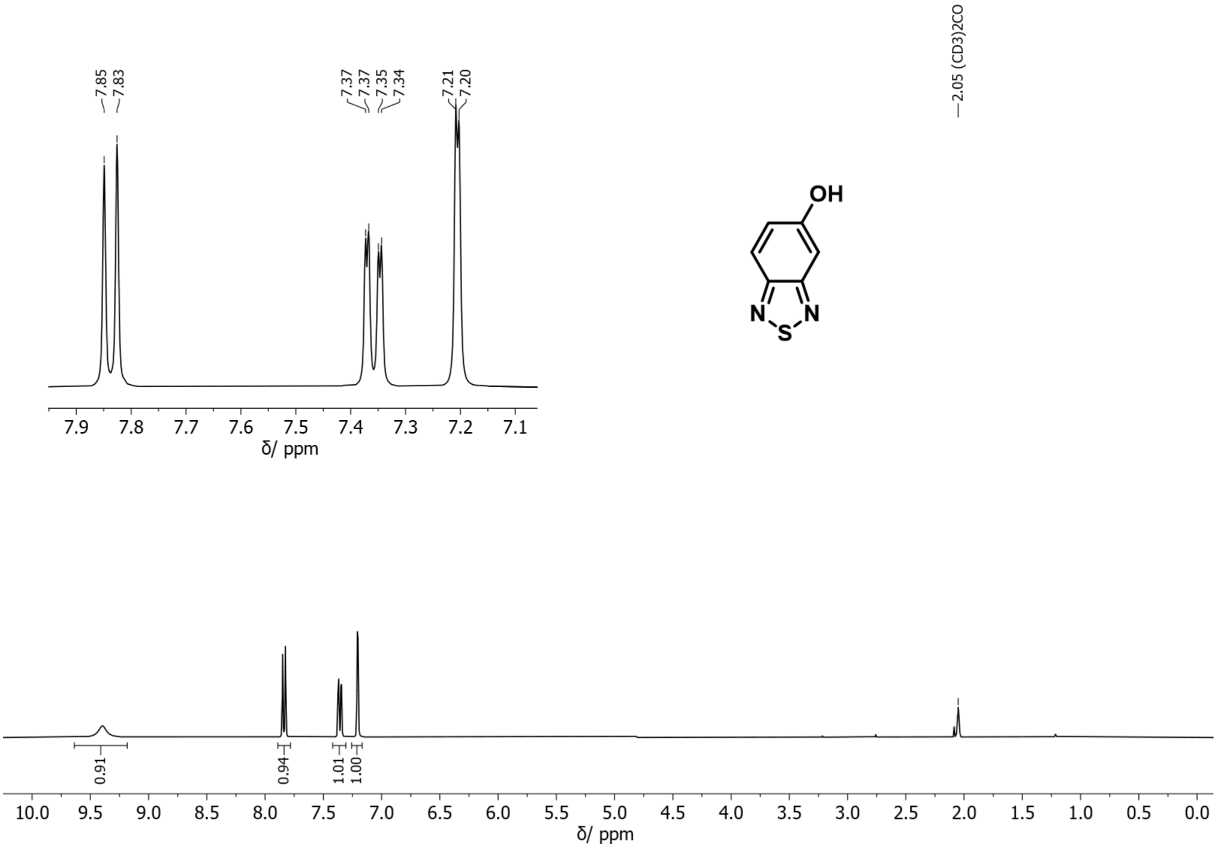

*Figure S45: ^{1}H NMR spectrum of **7** in acetone-d_6 (400 MHz).*

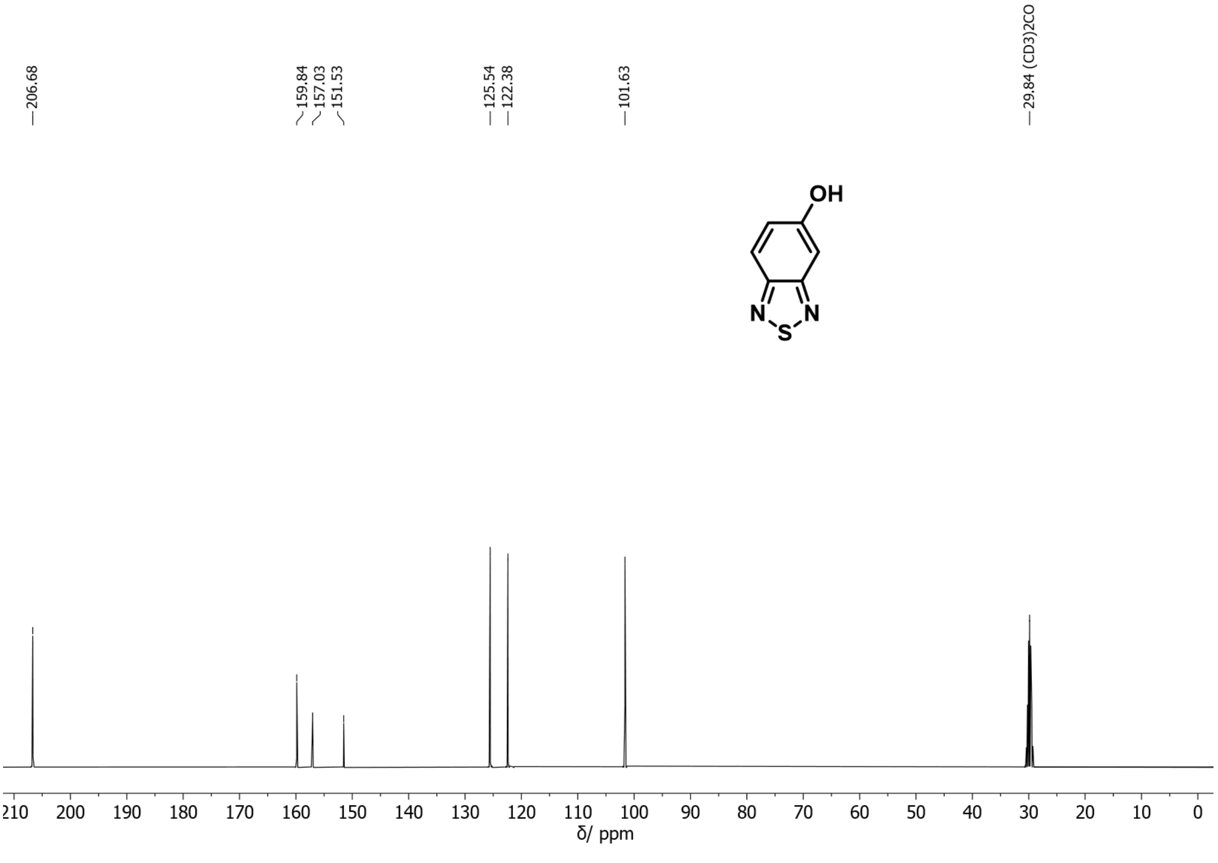

*Figure S46: $^{13}C\{^{1}H\}$ NMR spectrum of **7** in acetone-d_6 (101 MHz).*

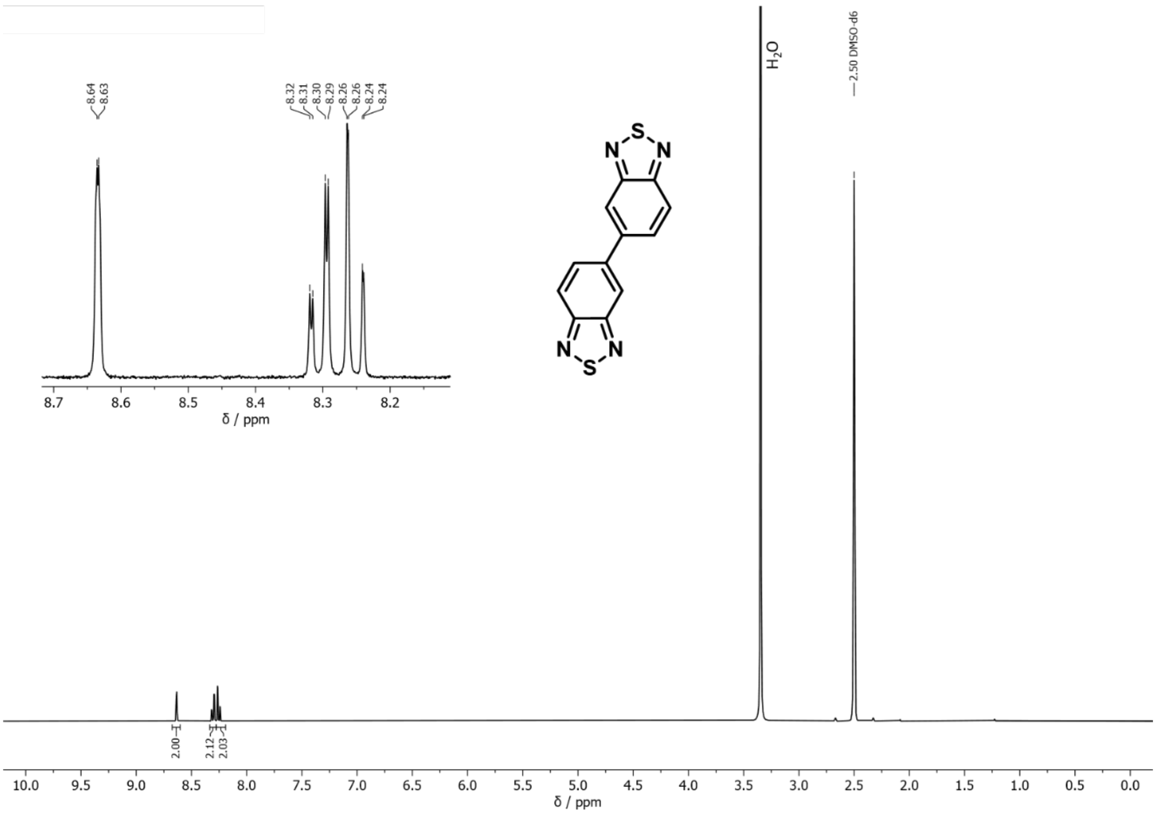

*Figure S47: ^{1}H NMR spectrum of **8** in DMSO-d_6 (400 MHz).*

^{13}C NMR spectrum could not be measured due to the poor solubility of **8**.

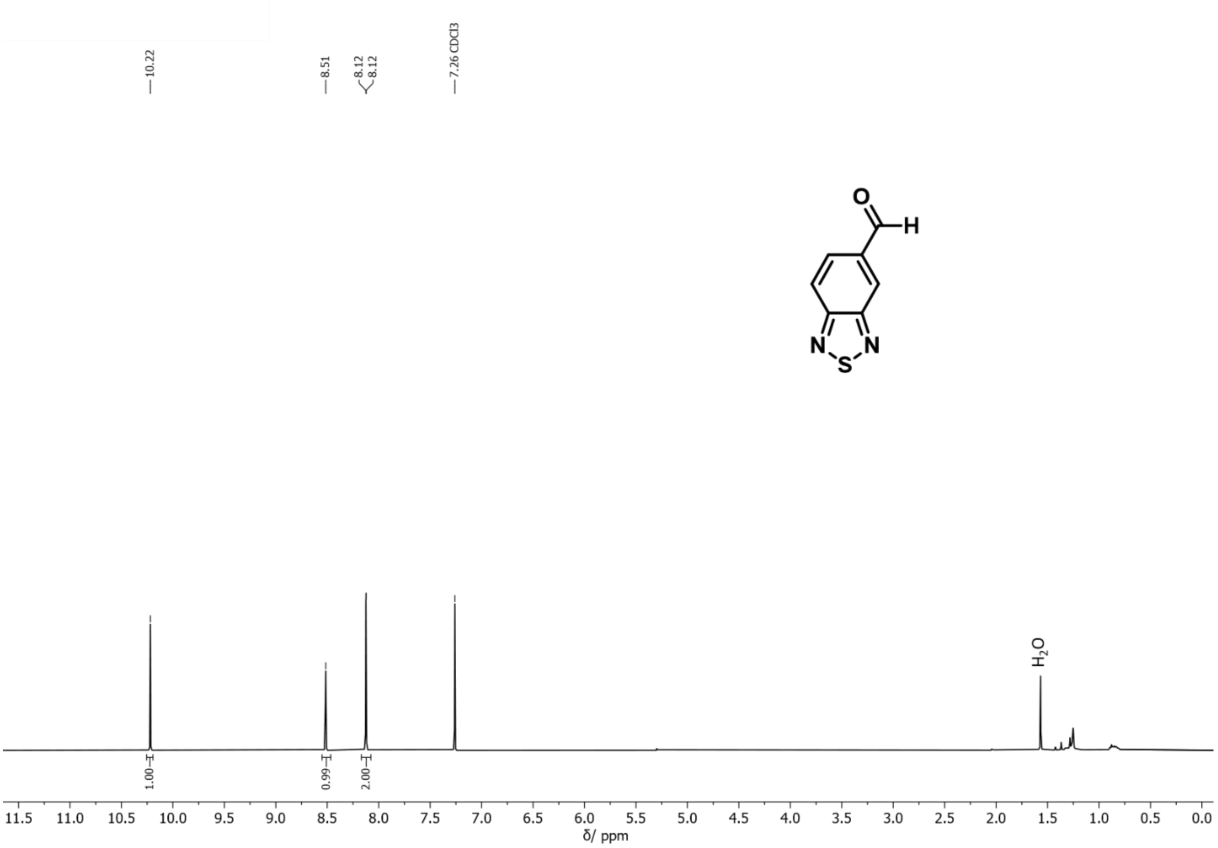

*Figure S98: ¹H NMR spectrum of **9** in $CDCl_3$ (400 MHz).*

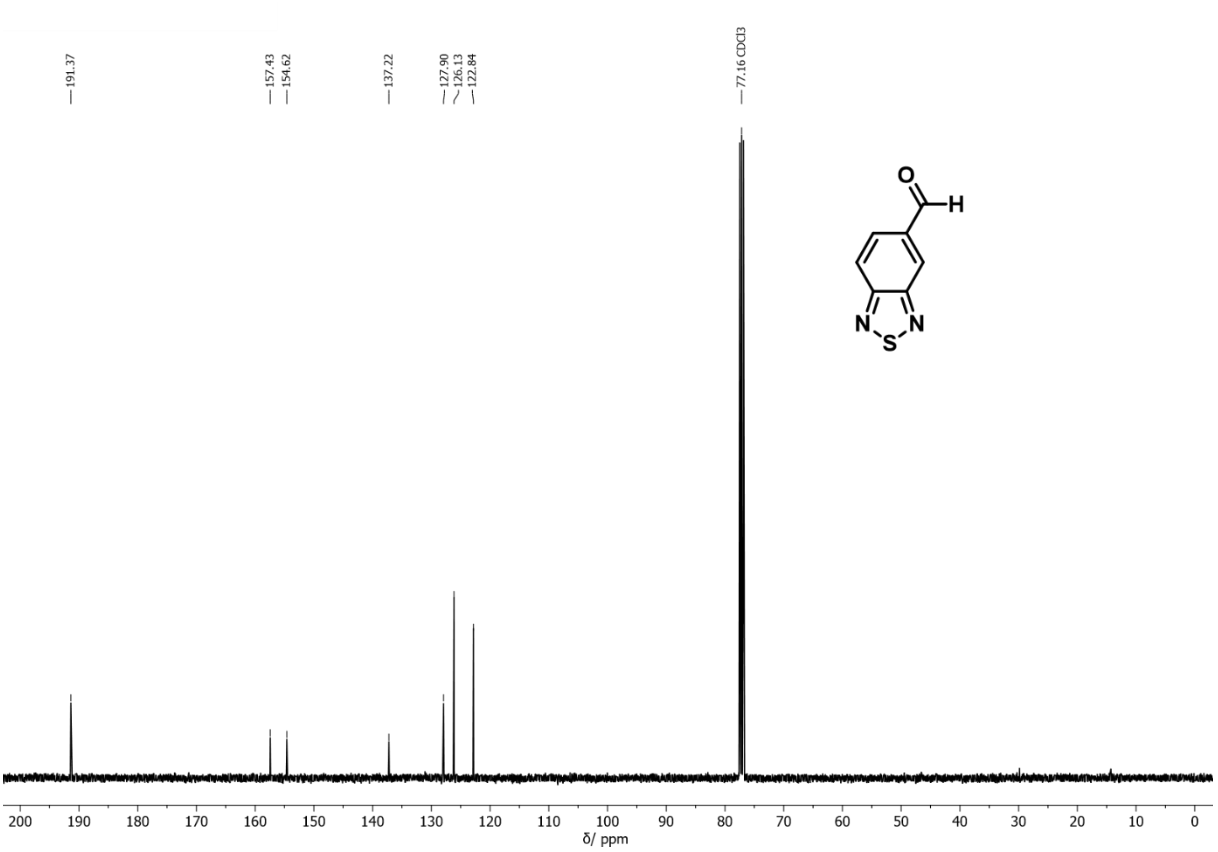

*Figure S10: $^{13}C\{^1H\}$ NMR spectrum of **9** in $CDCl_3$ (101 MHz).*

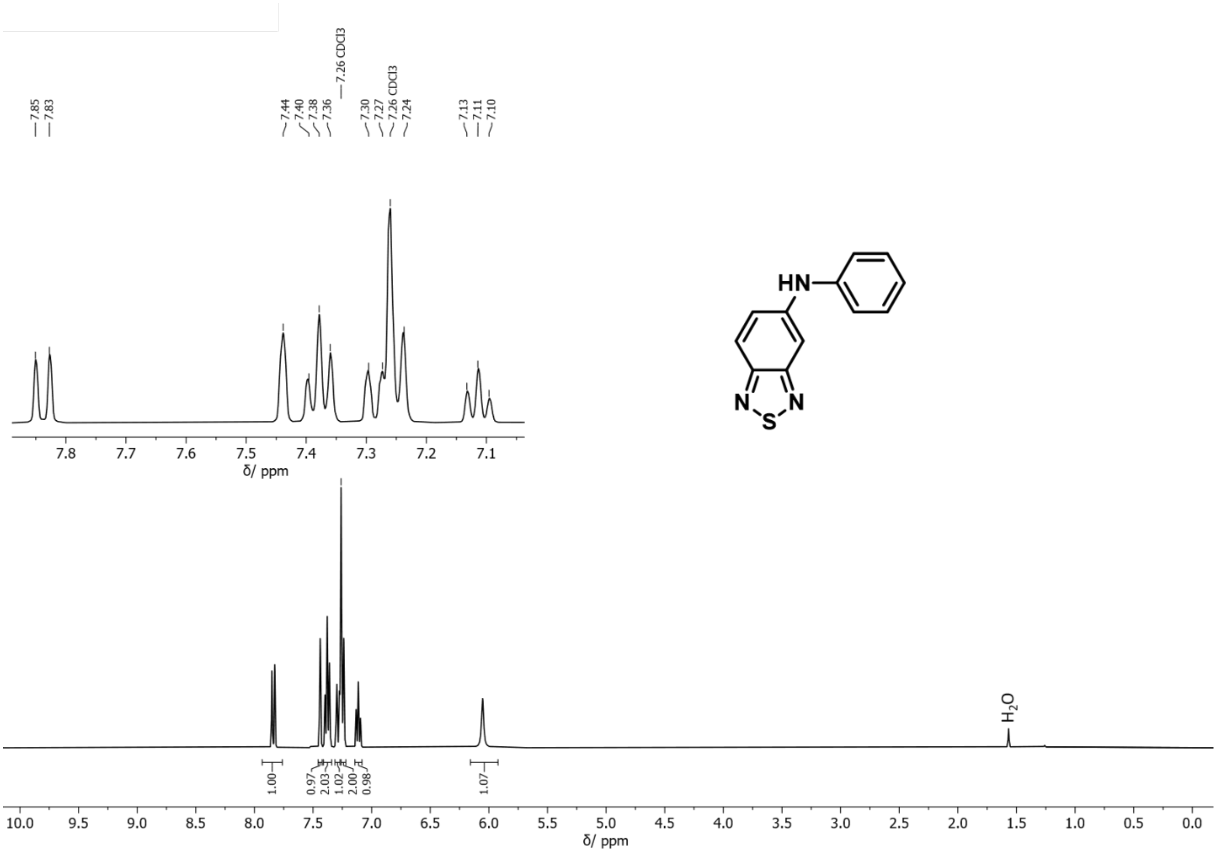

*Figure S50: ^{1}H NMR spectrum of **10a** in $CDCl_3$ (400 MHz).*

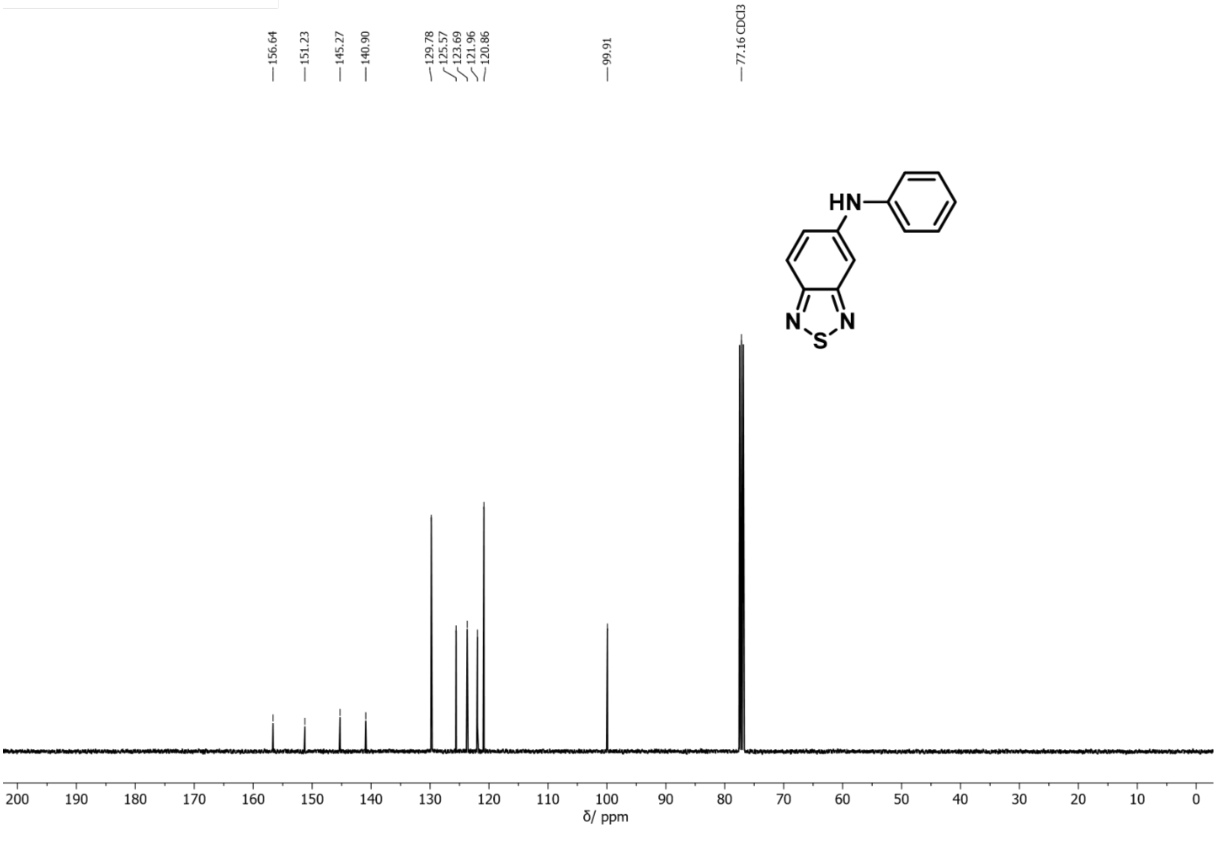

*Figure S51: $^{13}C\{^{1}H\}$ NMR spectrum of **10a** in $CDCl_3$ (101 MHz).*

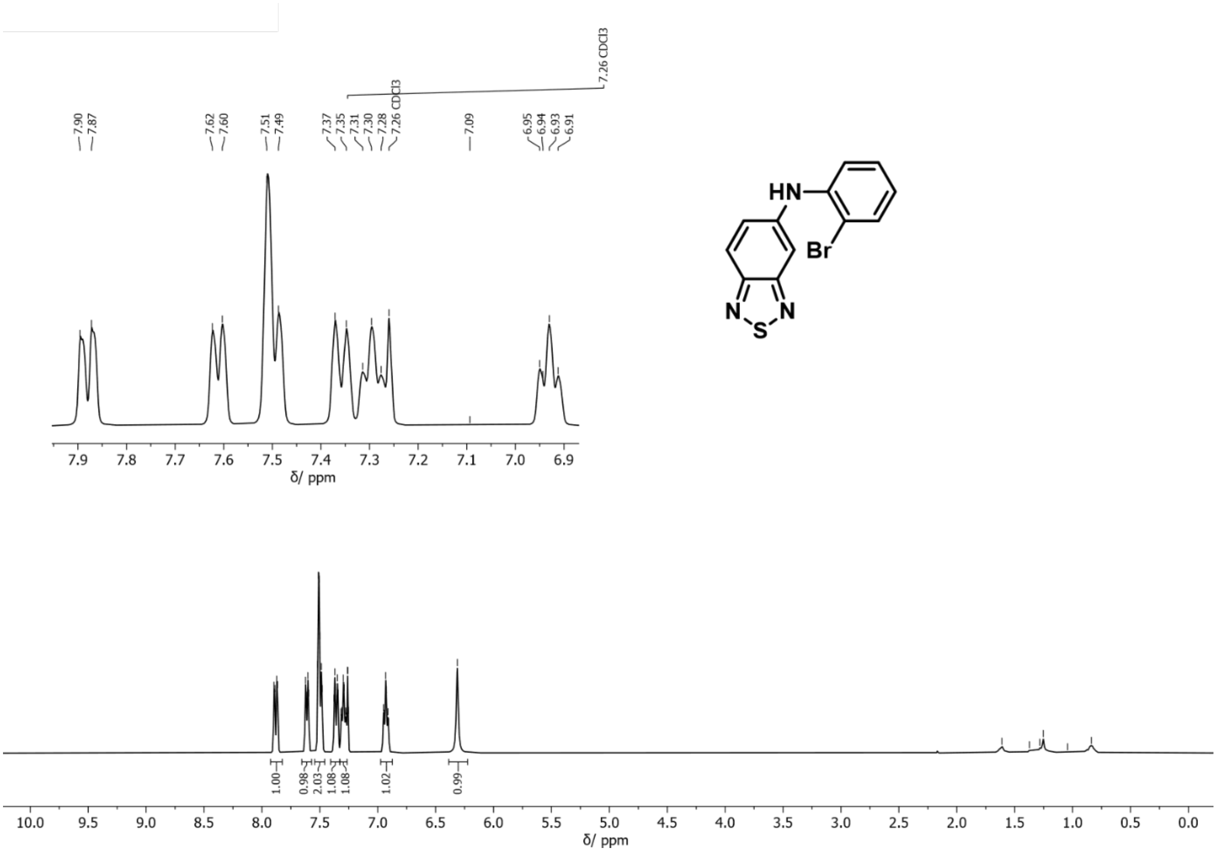

*Figure S52: ^{1}H NMR spectrum of **10b** in $CDCl_3$ (400 MHz).*

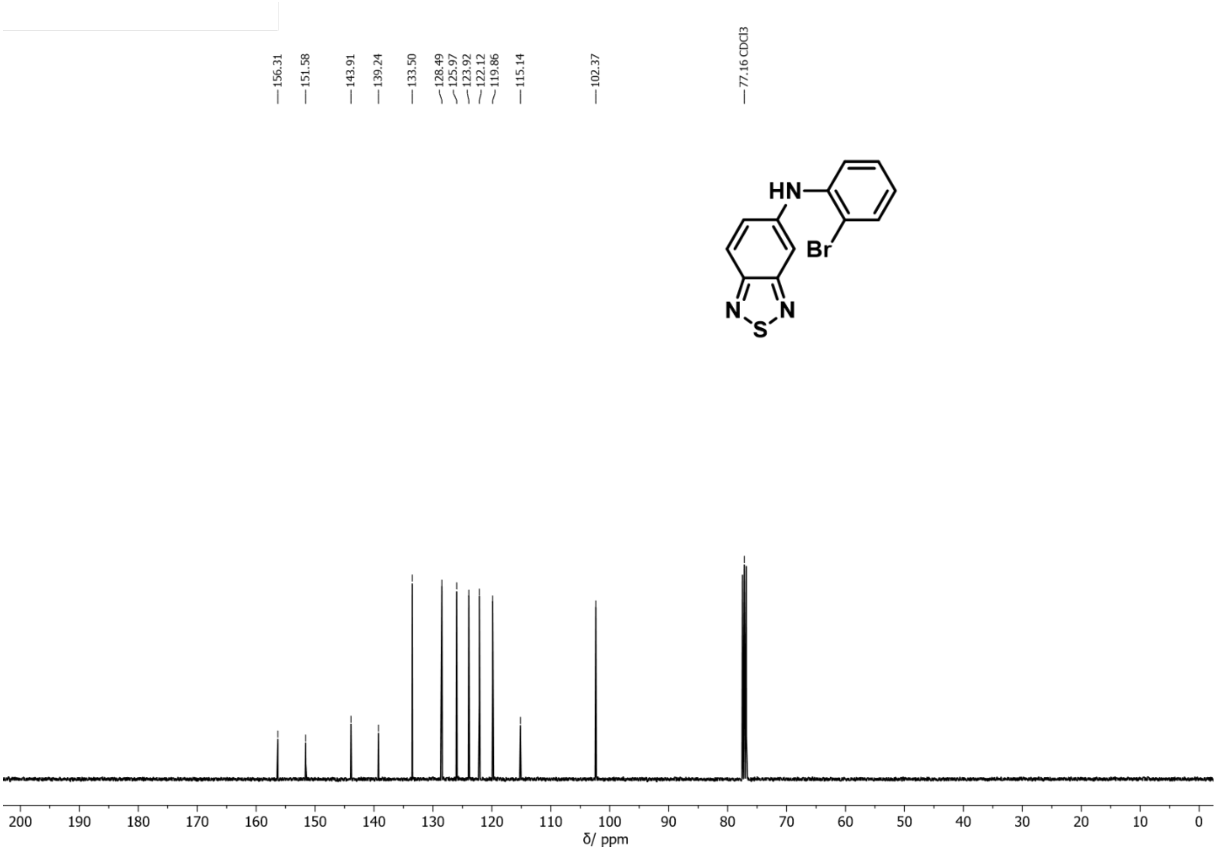

*Figure S53: $^{13}C\{^{1}H\}$ NMR spectrum of **10b** in $CDCl_3$ (101 MHz).*

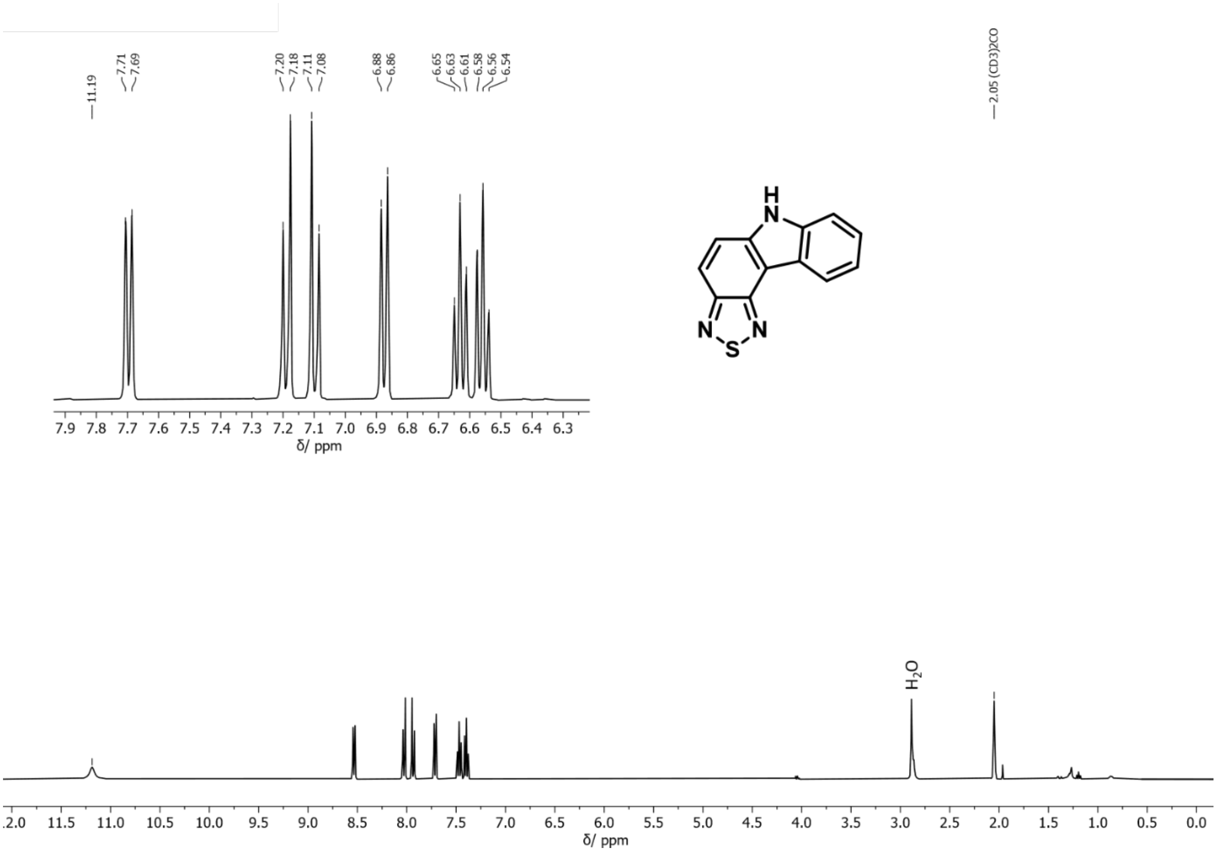

*Figure S54: ^{1}H NMR spectrum of **11a** in acetone-d$_6$ (400 MHz).*

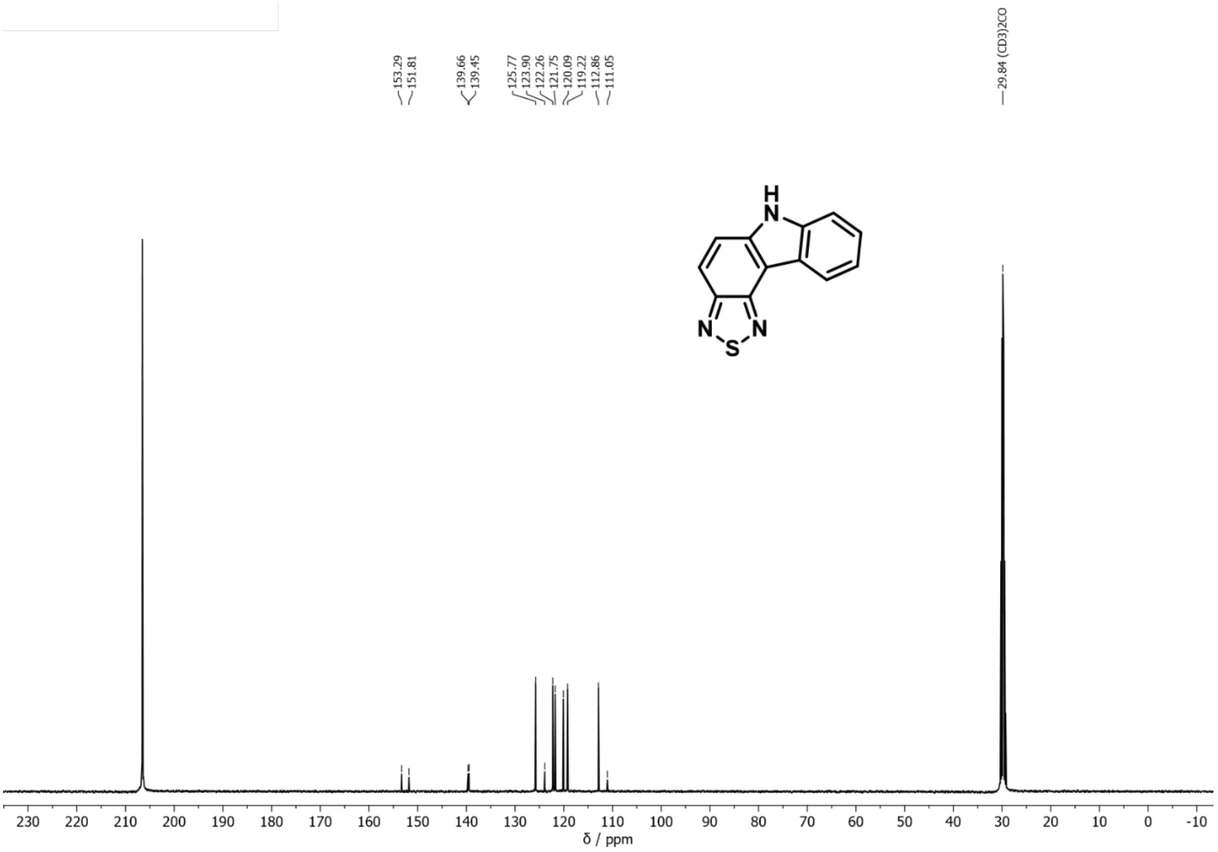

*Figure S55: ^{13}C{^{1}H} NMR spectrum of **11a** in acetone-d$_6$ (101 MHz).*

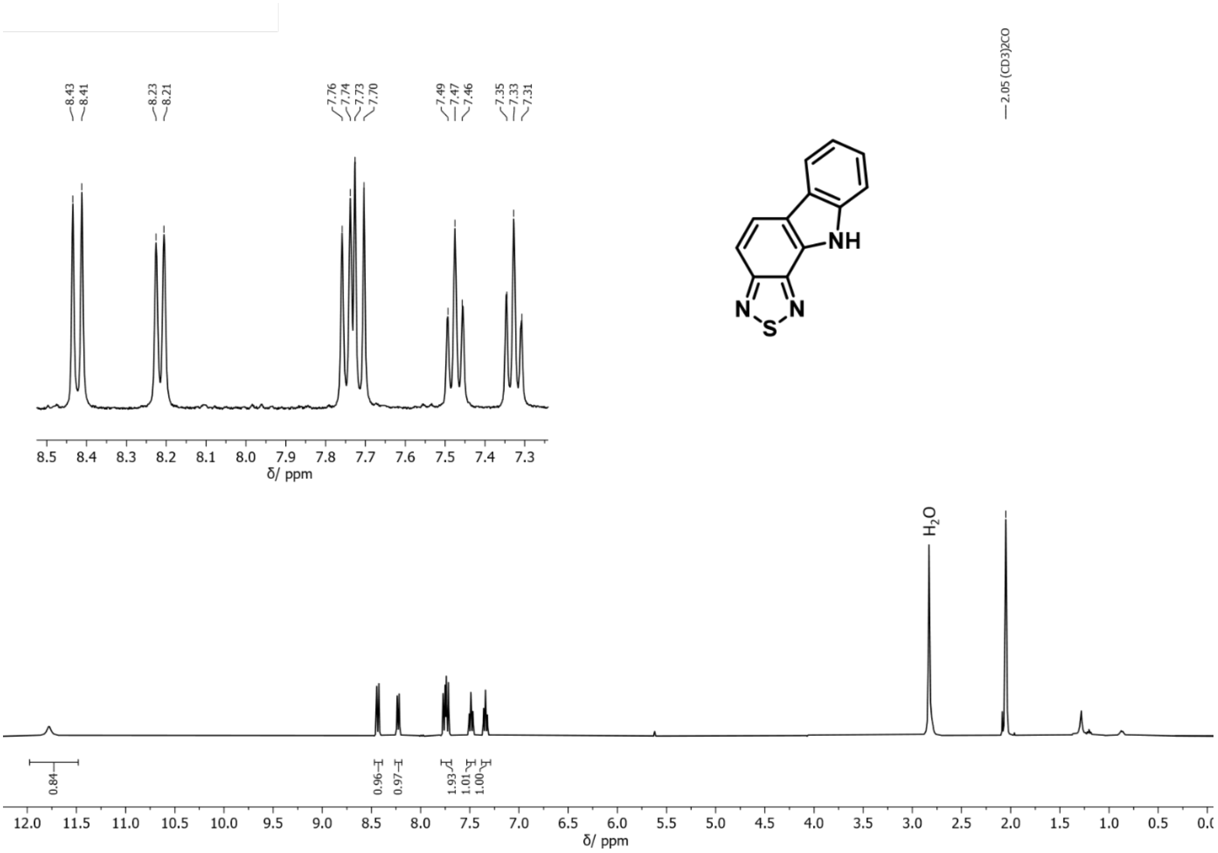

*Figure S56: ^{1}H NMR spectrum of **11b** in acetone-d_6 (400 MHz).*

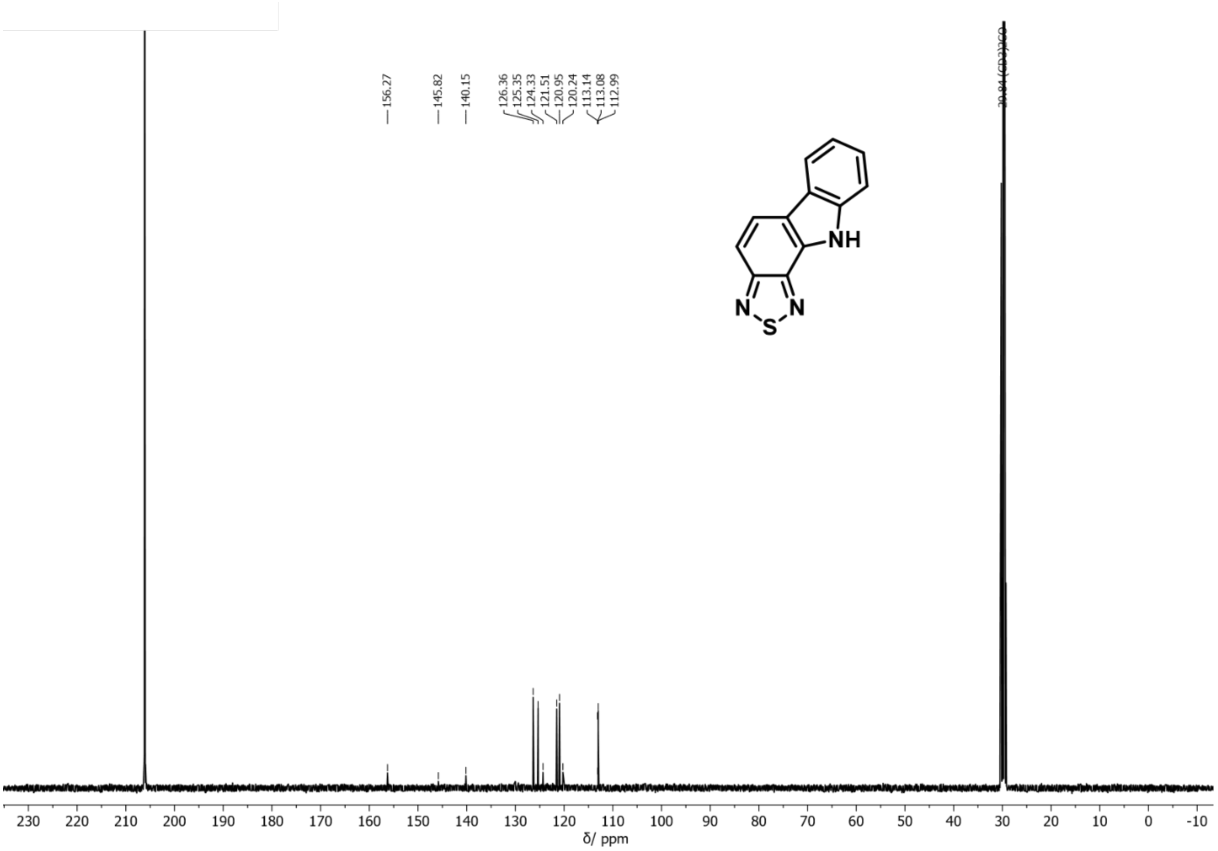

*Figure S11: $^{13}C\{^{1}H\}$ NMR spectrum of **11b** in acetone-d_6 (101 MHz).*

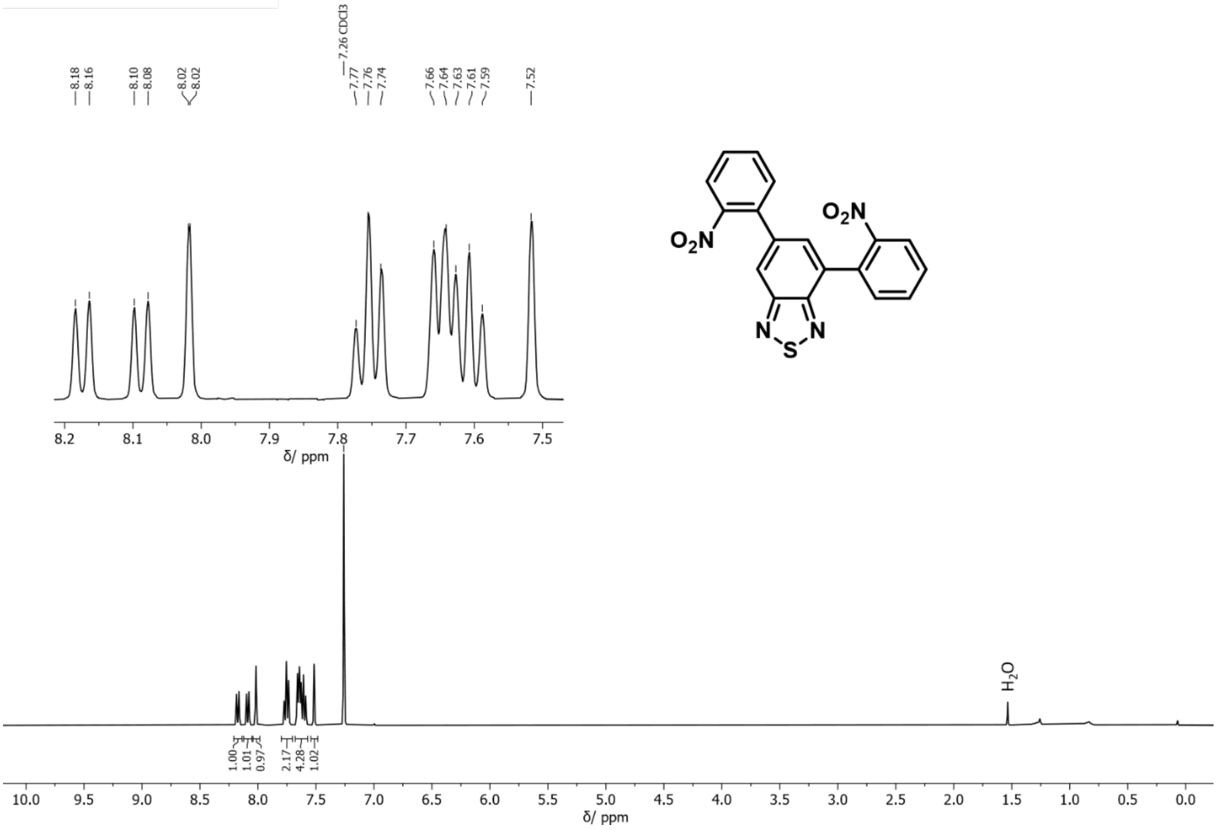

*Figure S58: ^{1}H NMR spectrum of **4i** in $CDCl_3$ (400 MHz).*

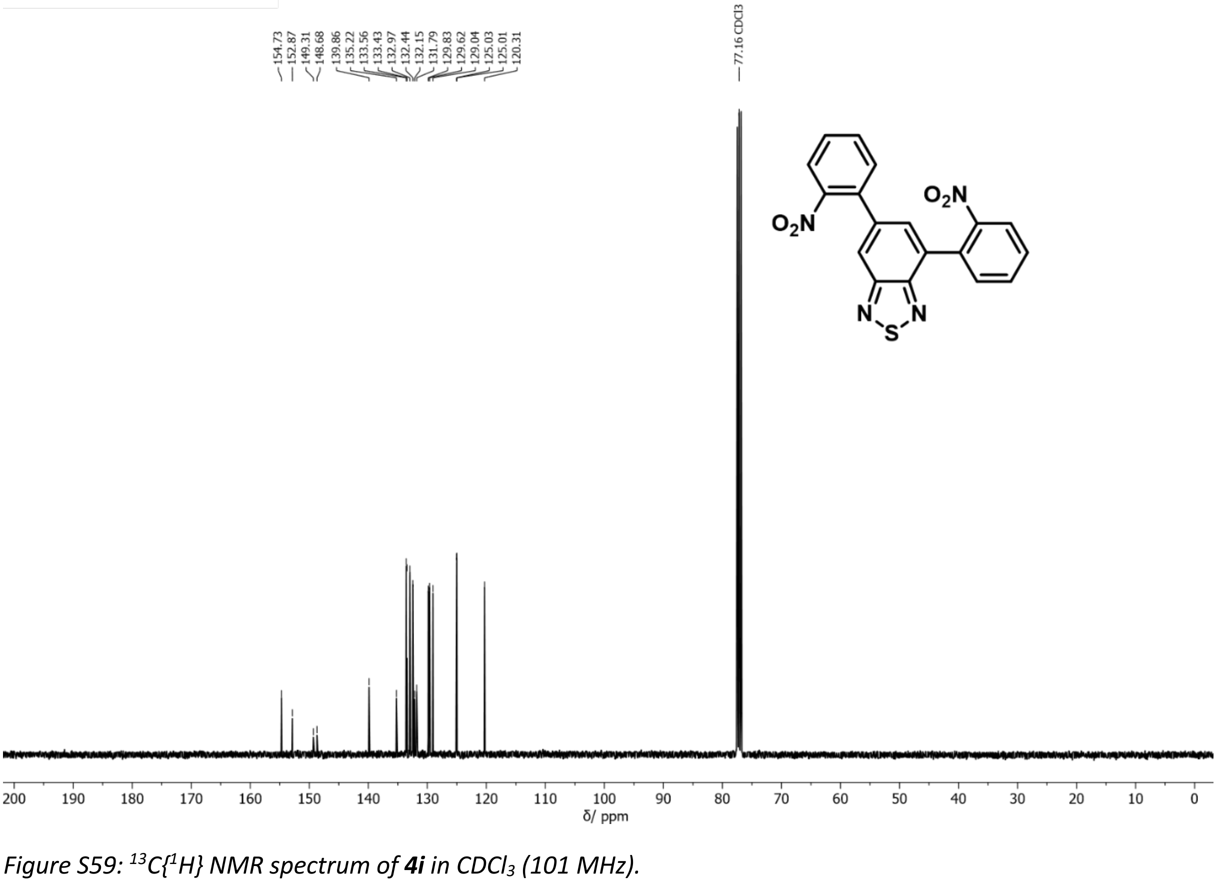

*Figure S59: $^{13}C\{^{1}H\}$ NMR spectrum of **4i** in $CDCl_3$ (101 MHz).*

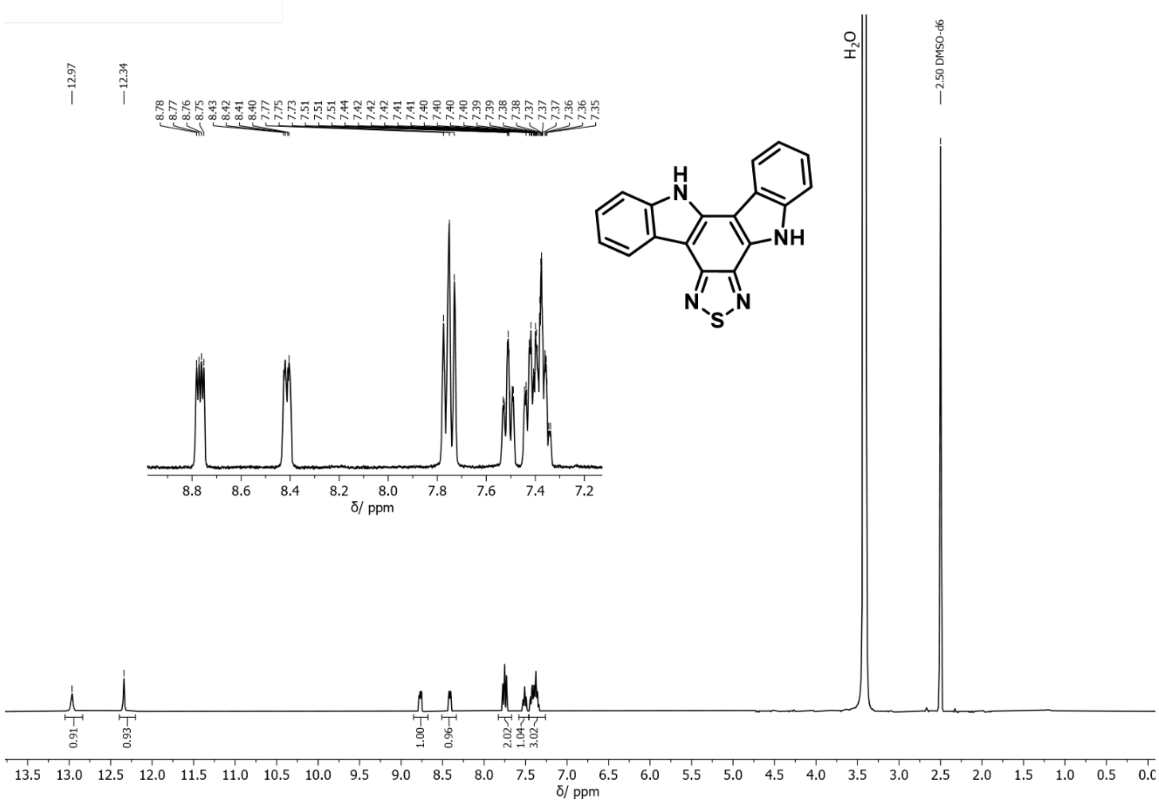

*Figure S60: ^{1}H NMR spectrum of **11c** in DMSO-d_6 (400 MHz).*

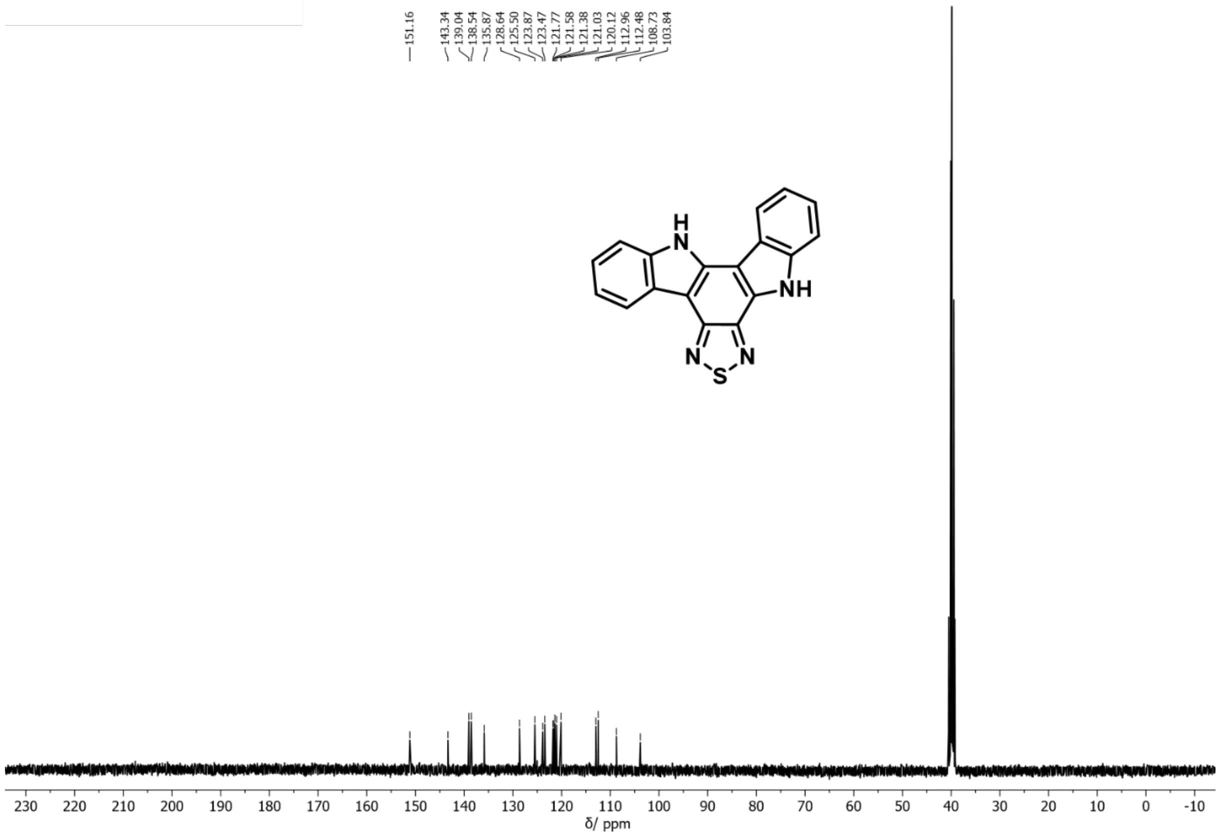

*Figure S61: ^{13}C{^{1}H} NMR spectrum of **11c** in DMSO-d_6 (101 MHz).*

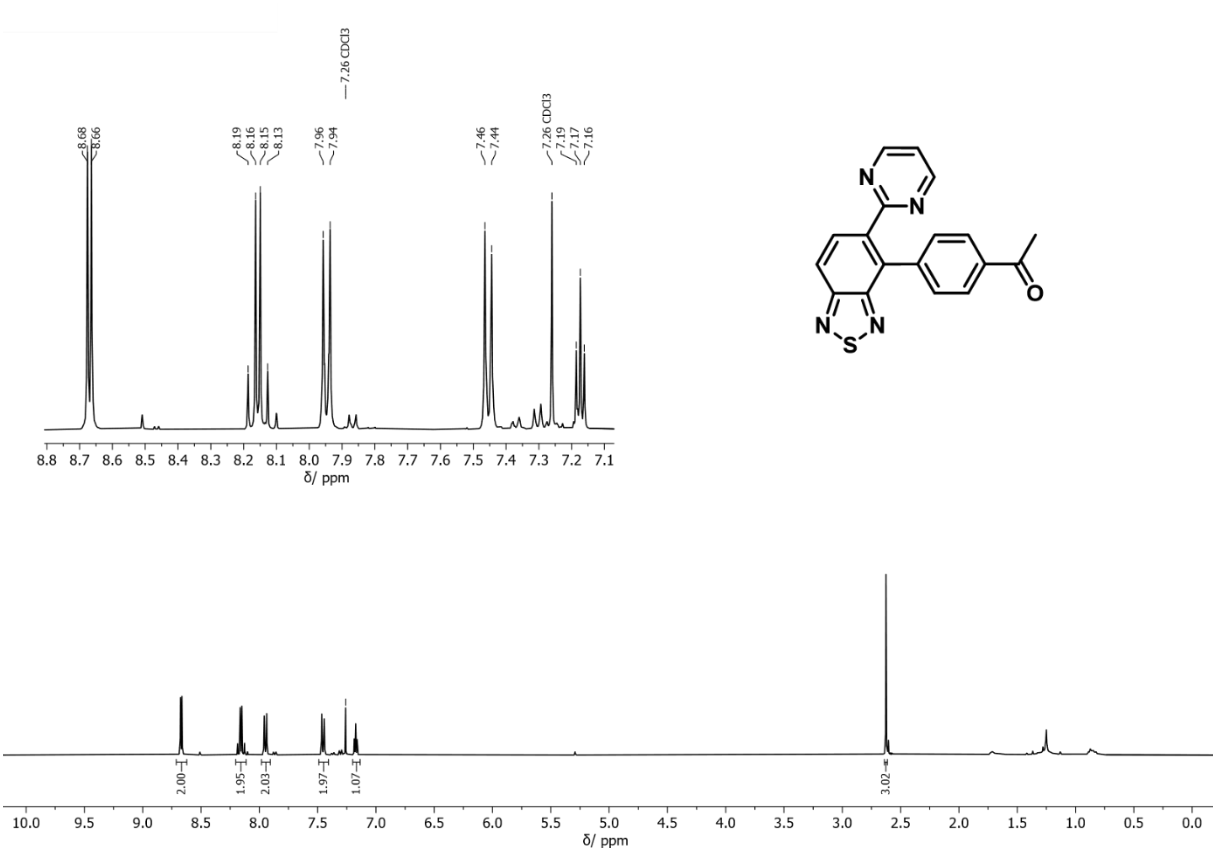

*Figure S62: ^{1}H NMR spectrum of **12a** (and presumably its isomer, ratio approximately 15:1) in $CDCl_3$ (400 MHz).*

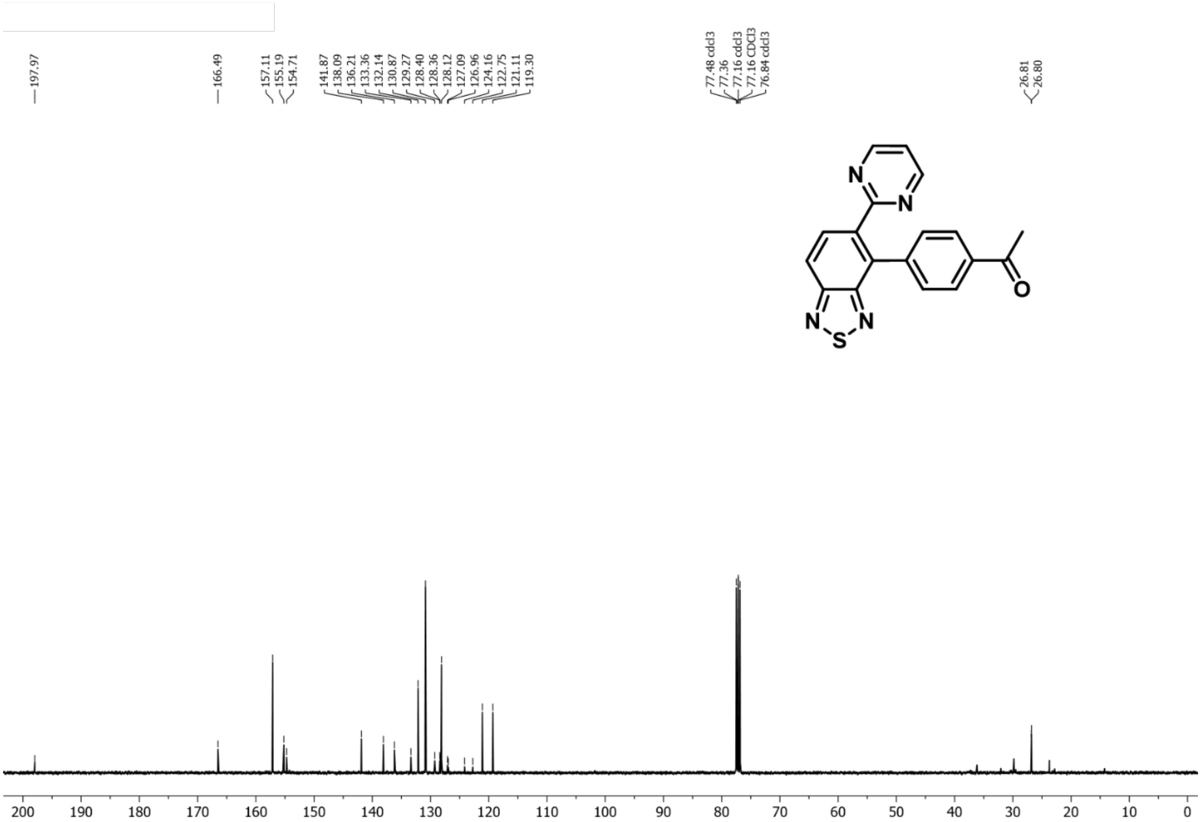

*Figure S63: $^{13}C\{^{1}H\}$ NMR spectrum of **12a** (and presumably its isomer, ratio approximately 15:1) in $CDCl_3$ (101 MHz).*

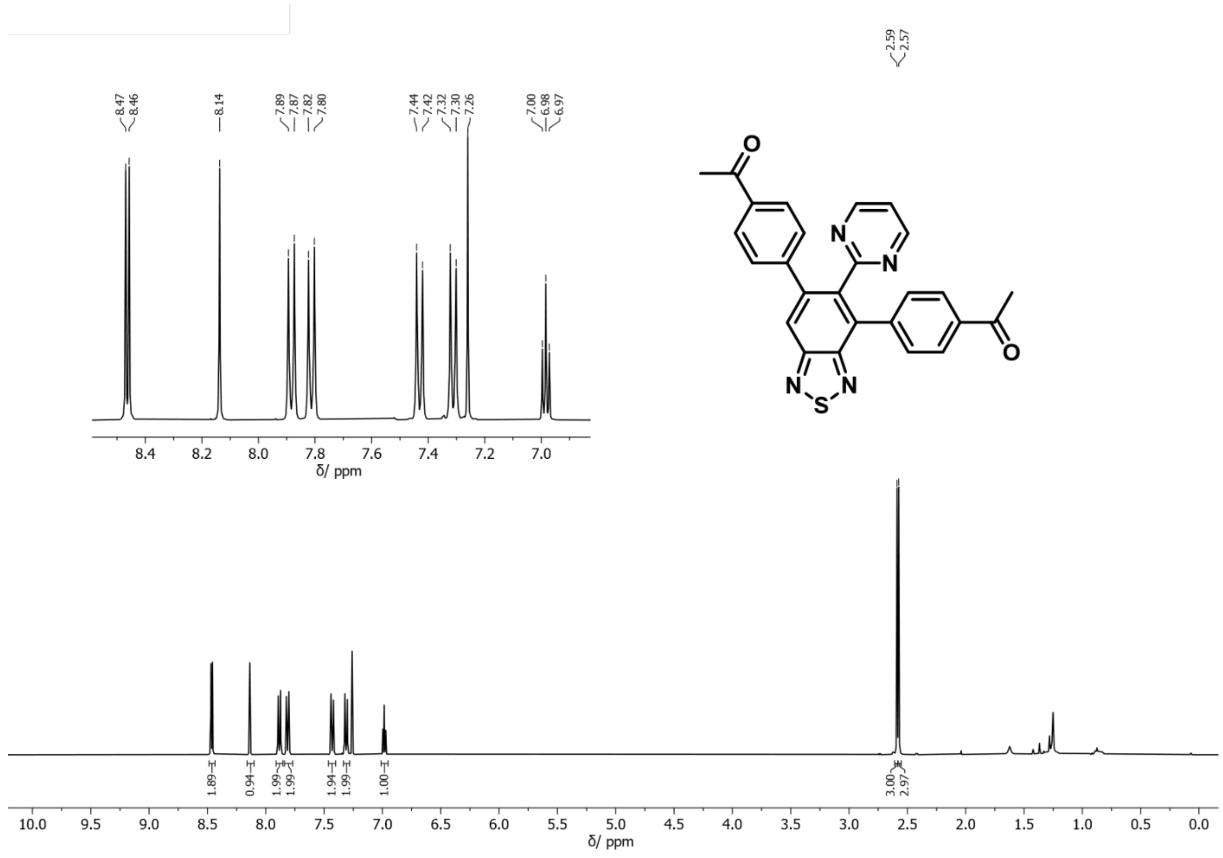

*Figure S64: ^{1}H NMR spectrum of **12b** in $CDCl_3$ (400 MHz).*

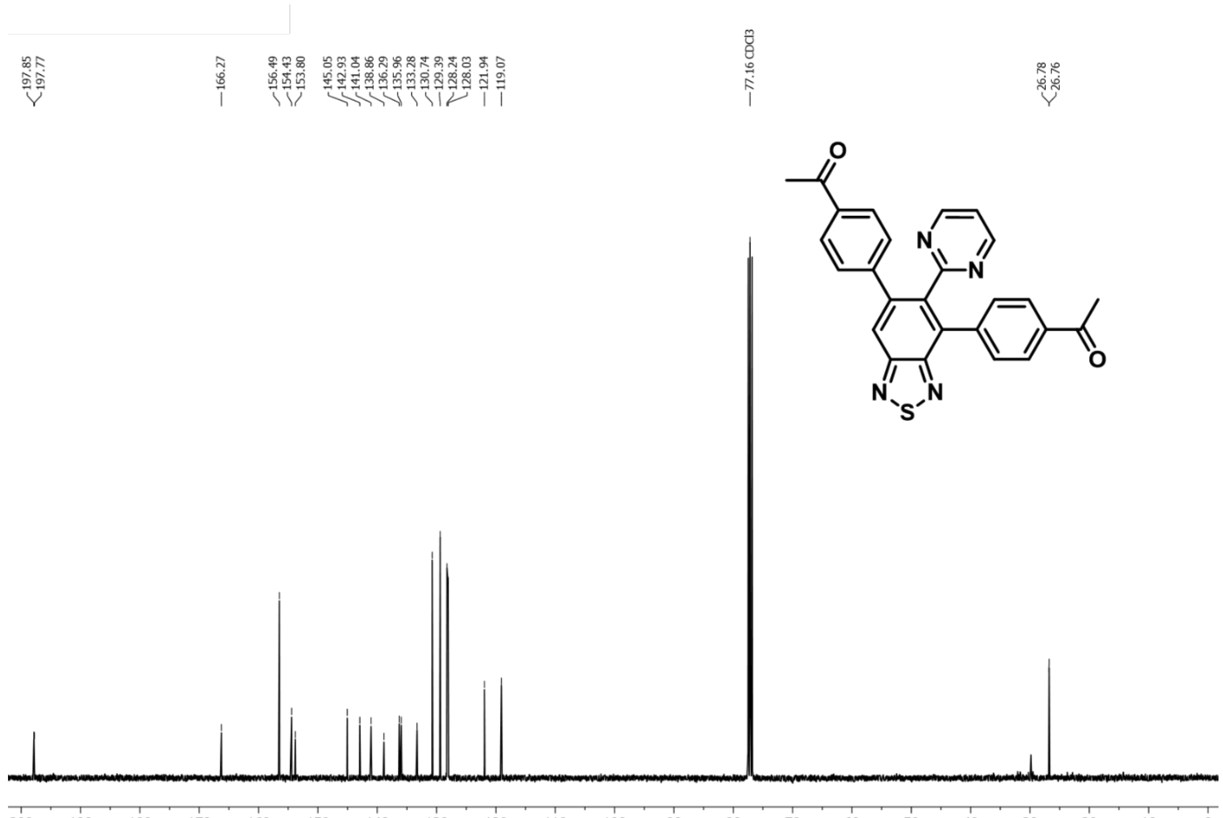

*Figure S65: $^{13}C\{^{1}H\}$ NMR spectrum of **12b** in $CDCl_3$ (101 MHz).*

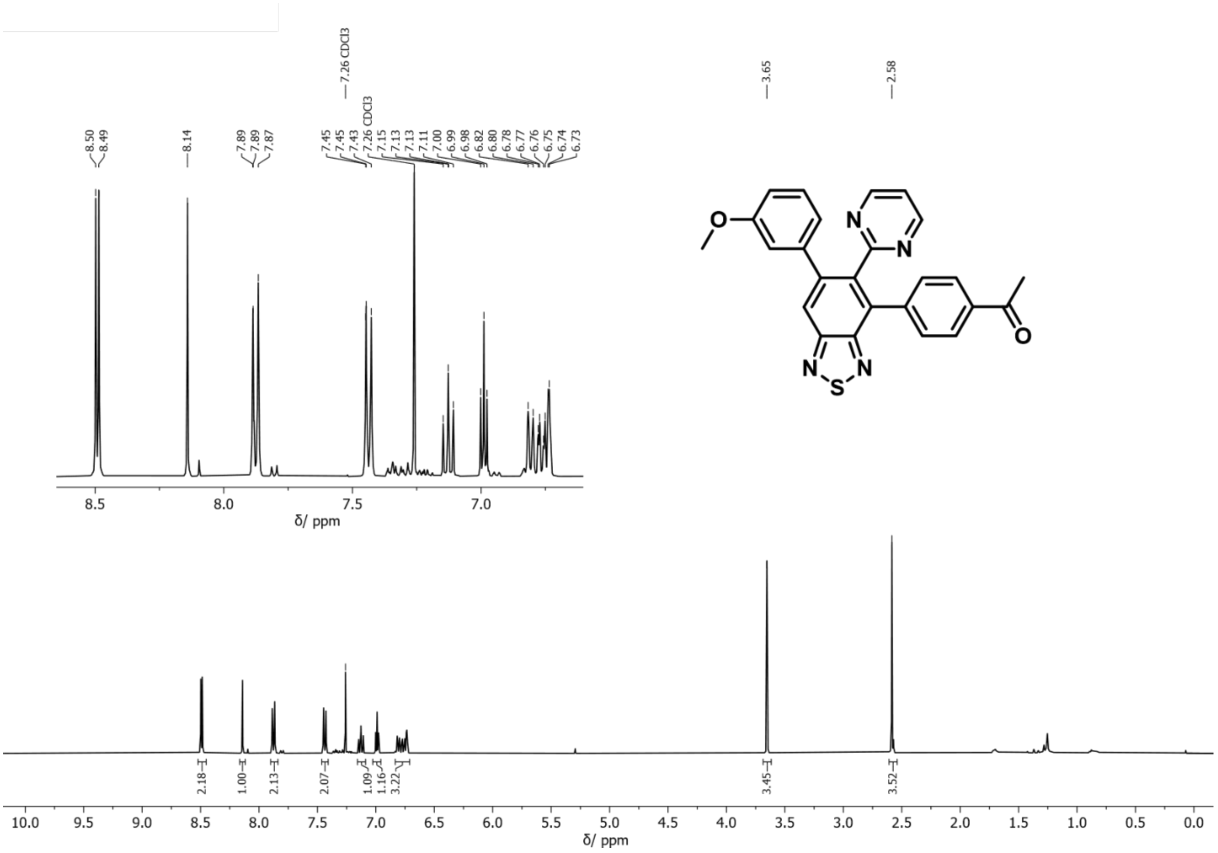

*Figure S12: ^{1}H NMR spectrum of **12c** (and presumably its isomer, ratio approximately 17:1) in $CDCl_3$ (400 MHz).*

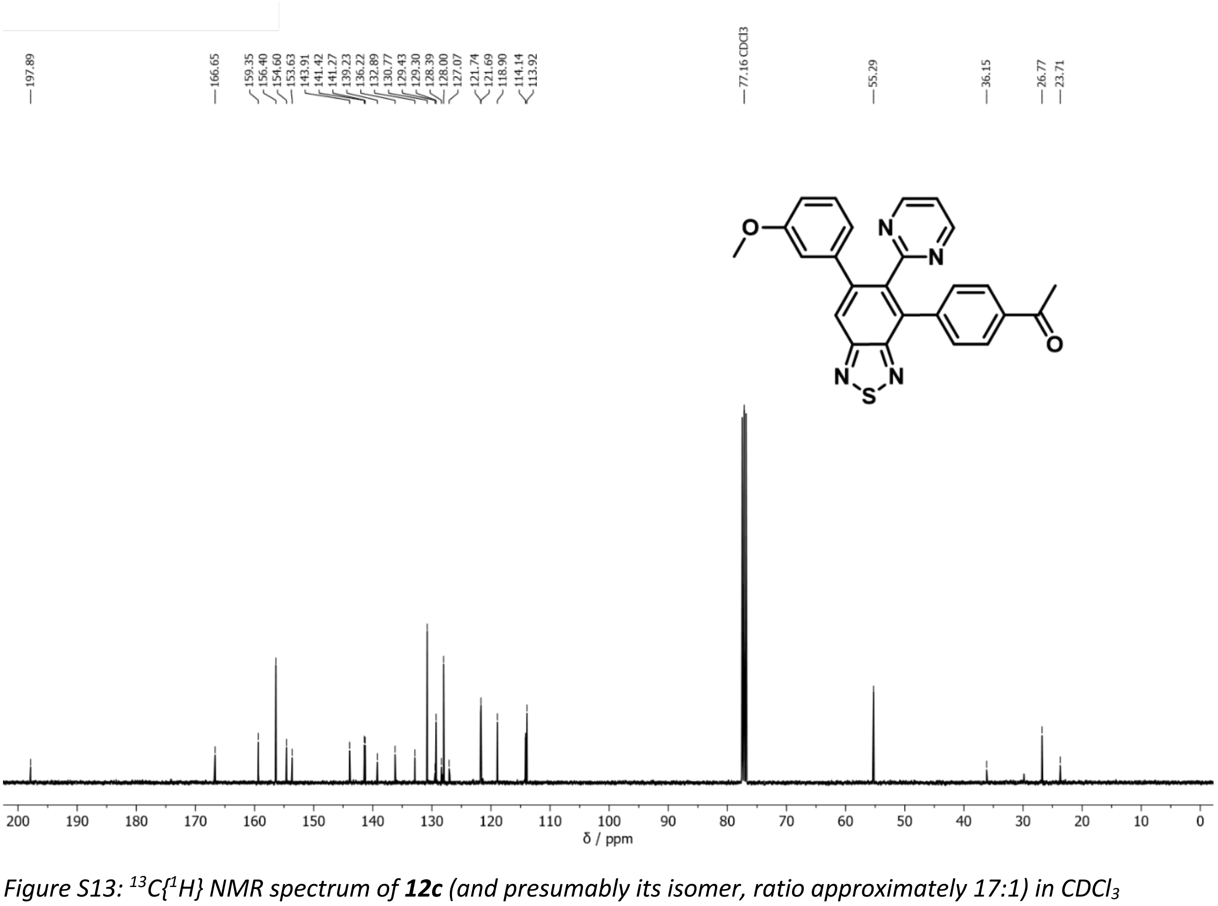

*Figure S13: $^{13}C\{^{1}H\}$ NMR spectrum of **12c** (and presumably its isomer, ratio approximately 17:1) in $CDCl_3$ (101 MHz).*

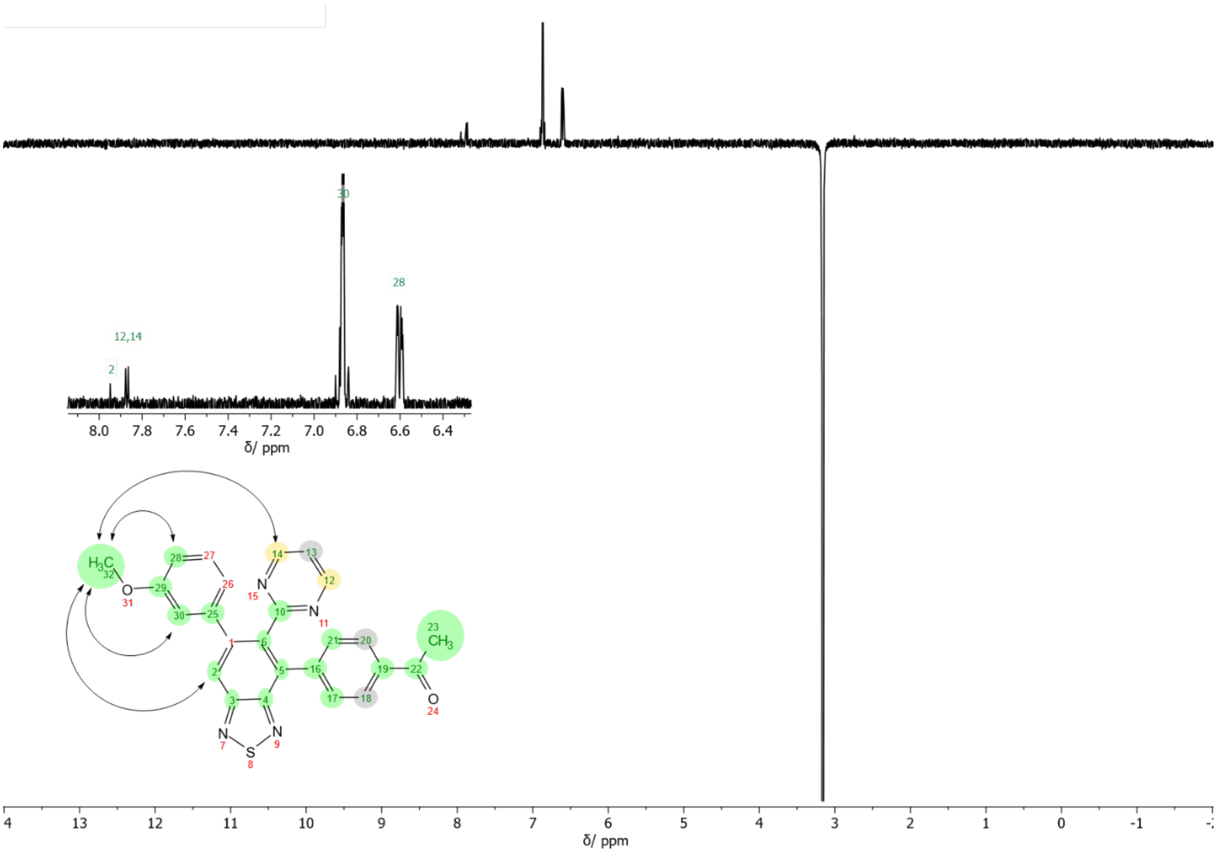

*Figure S68: 1D-NOESY-spectrum of **12c** in benzene-d$_6$ (excitation at 3.15 ppm (methoxy group labelled as 32).*

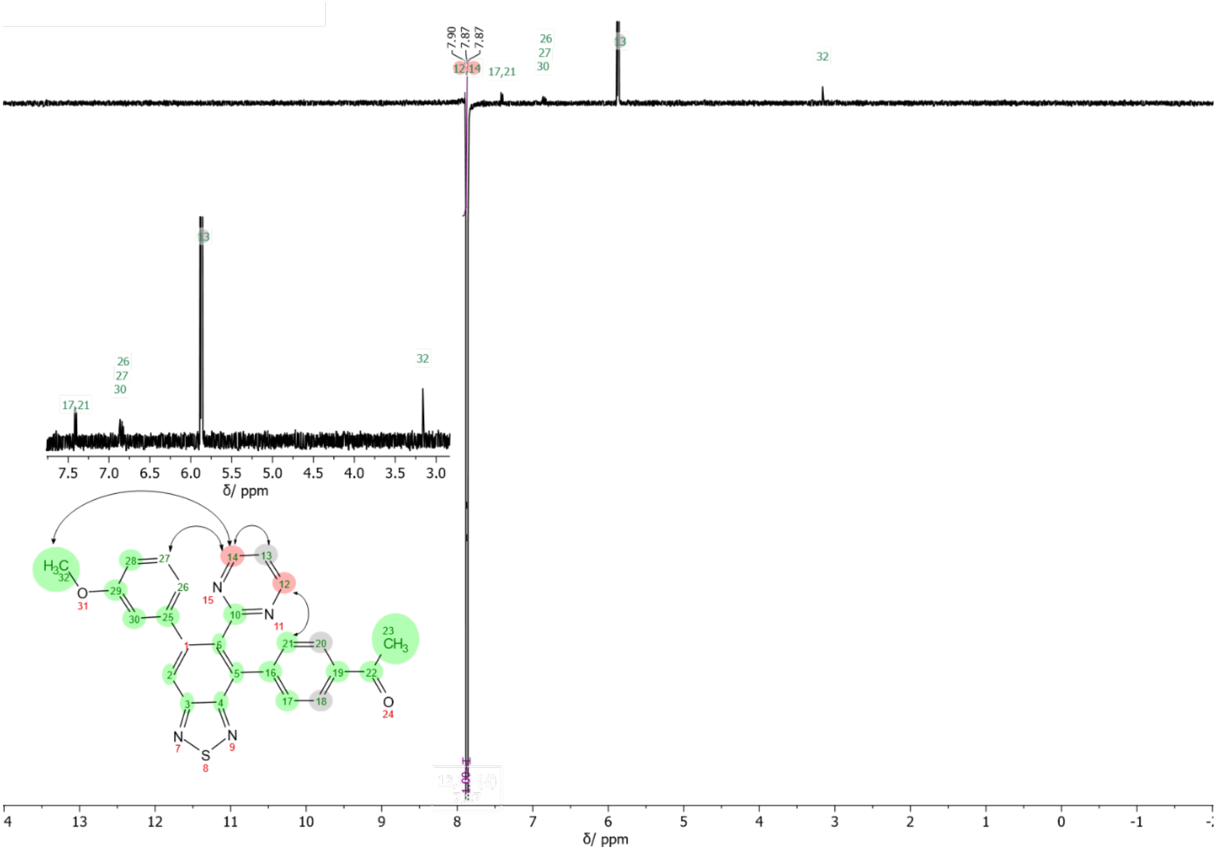

*Figure S69: 1D-NOESY-spectrum of **12c** in benzene-d$_6$ (excitation at 7.87 ppm (pyrimidyl-C-H labelled as 12 & 14).*

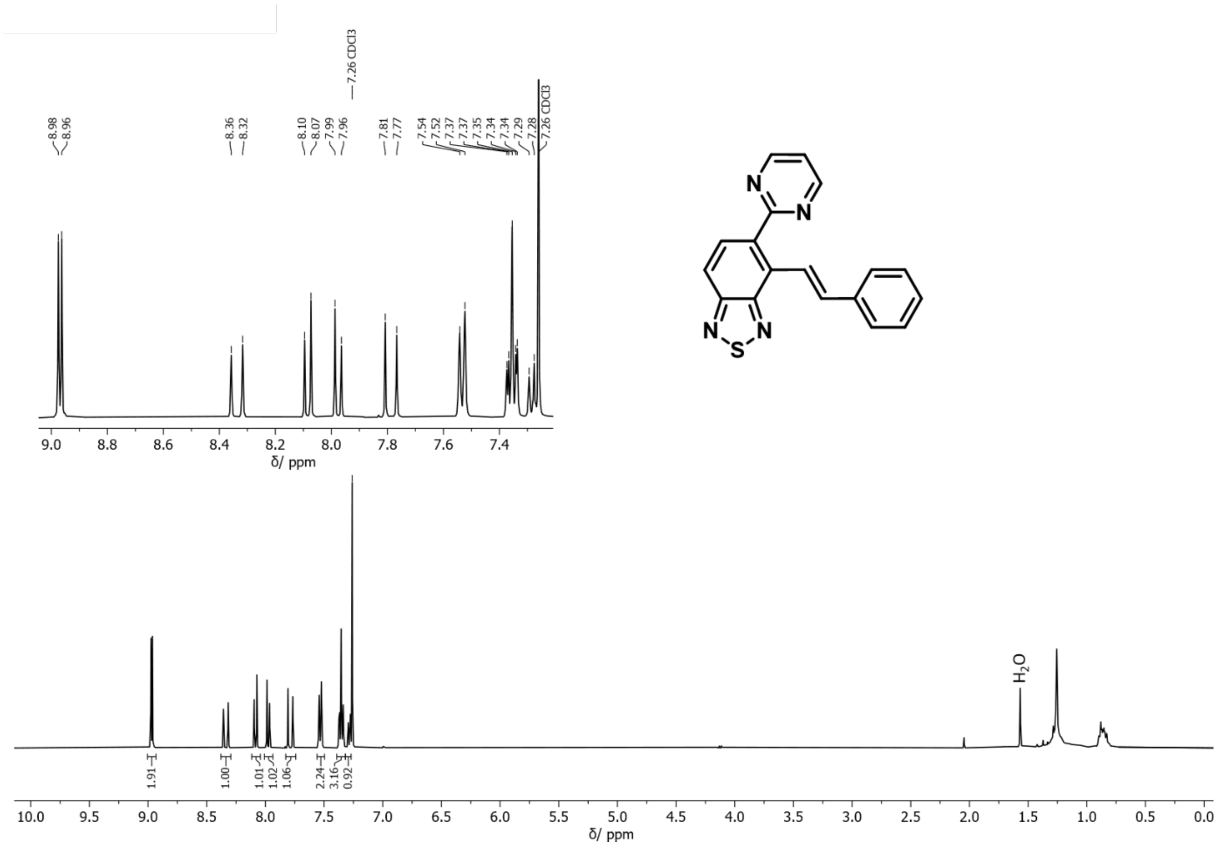

*Figure S70: ^{1}H NMR spectrum of **13a** in $CDCl_3$ (400 MHz).*

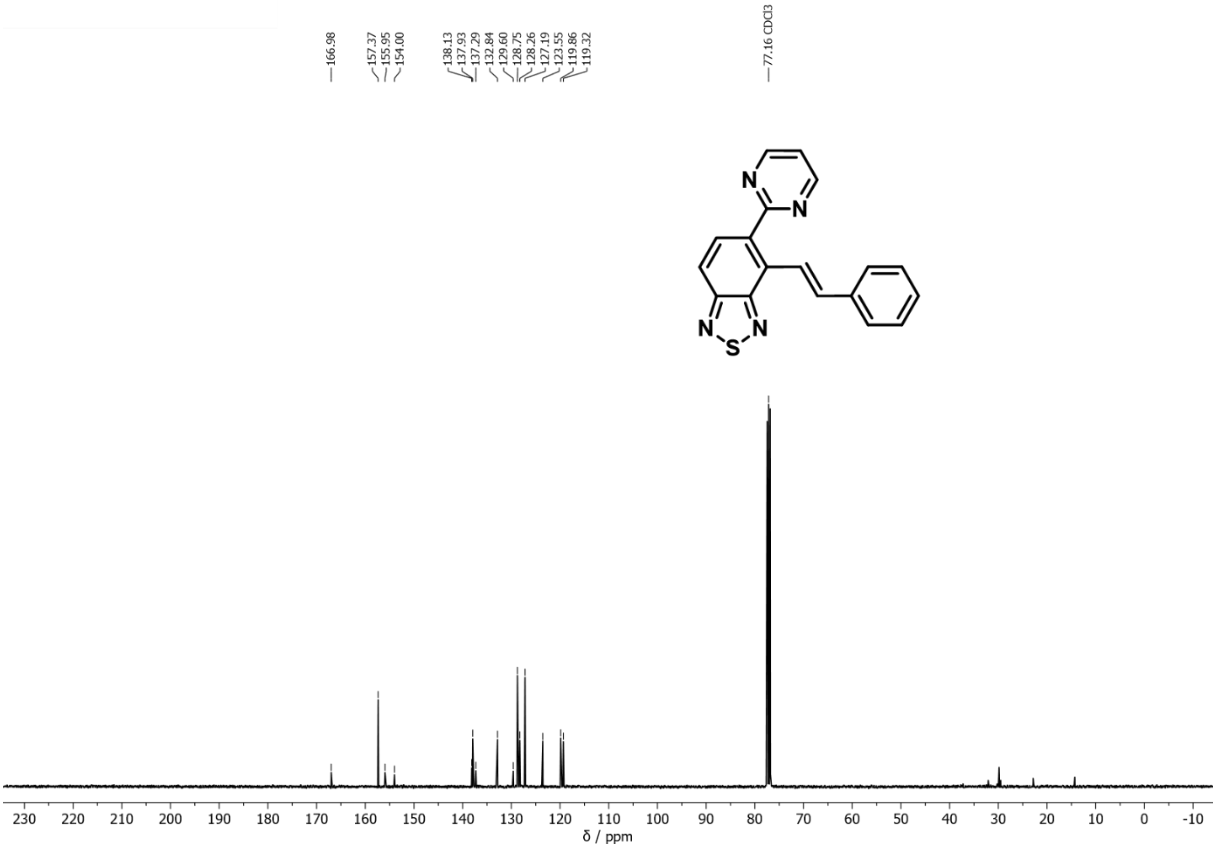

*Figure S71: $^{13}C\{^{1}H\}$ NMR spectrum of **13a** in $CDCl_3$ (101 MHz).*

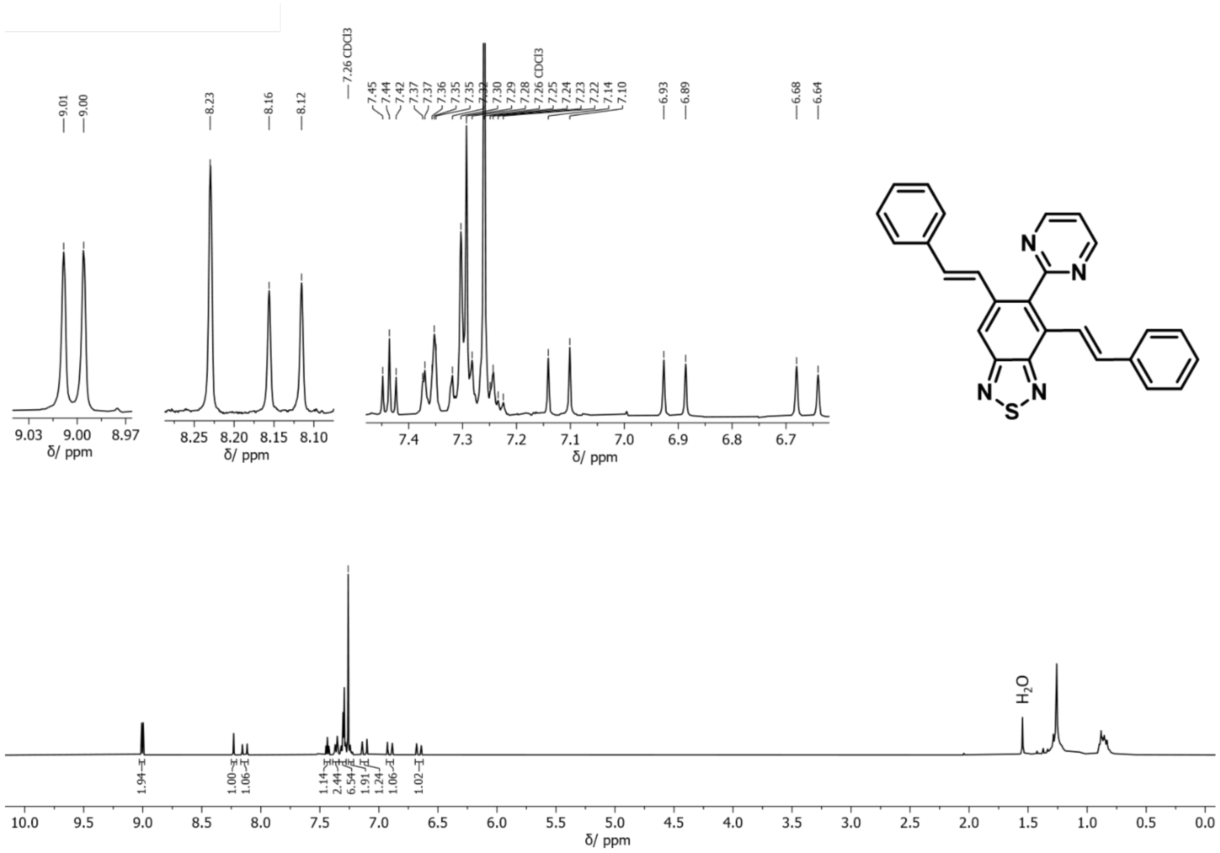

*Figure S72: ^{1}H NMR spectrum of **13b** in $CDCl_3$ (400 MHz).*

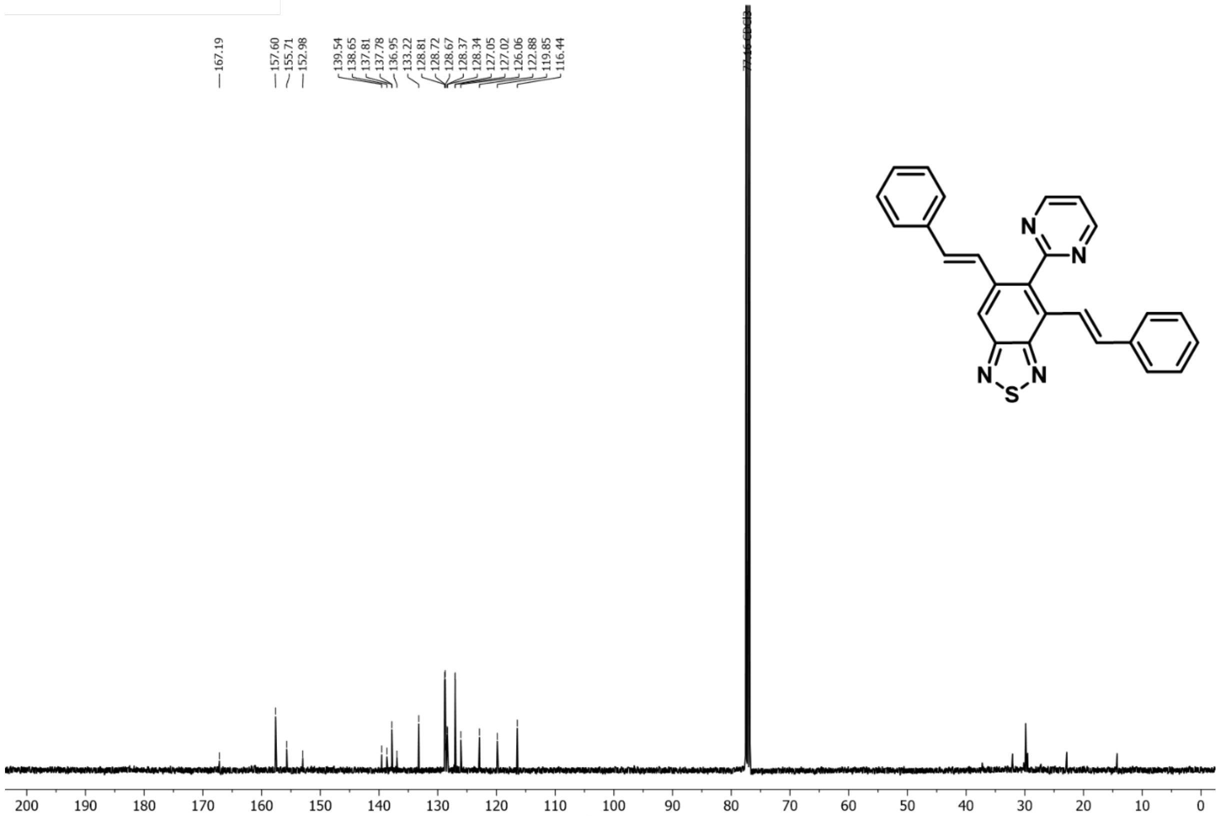

*Figure S73: $^{13}C\{^{1}H\}$ NMR spectrum of **13b** in $CDCl_3$ (101 MHz).*

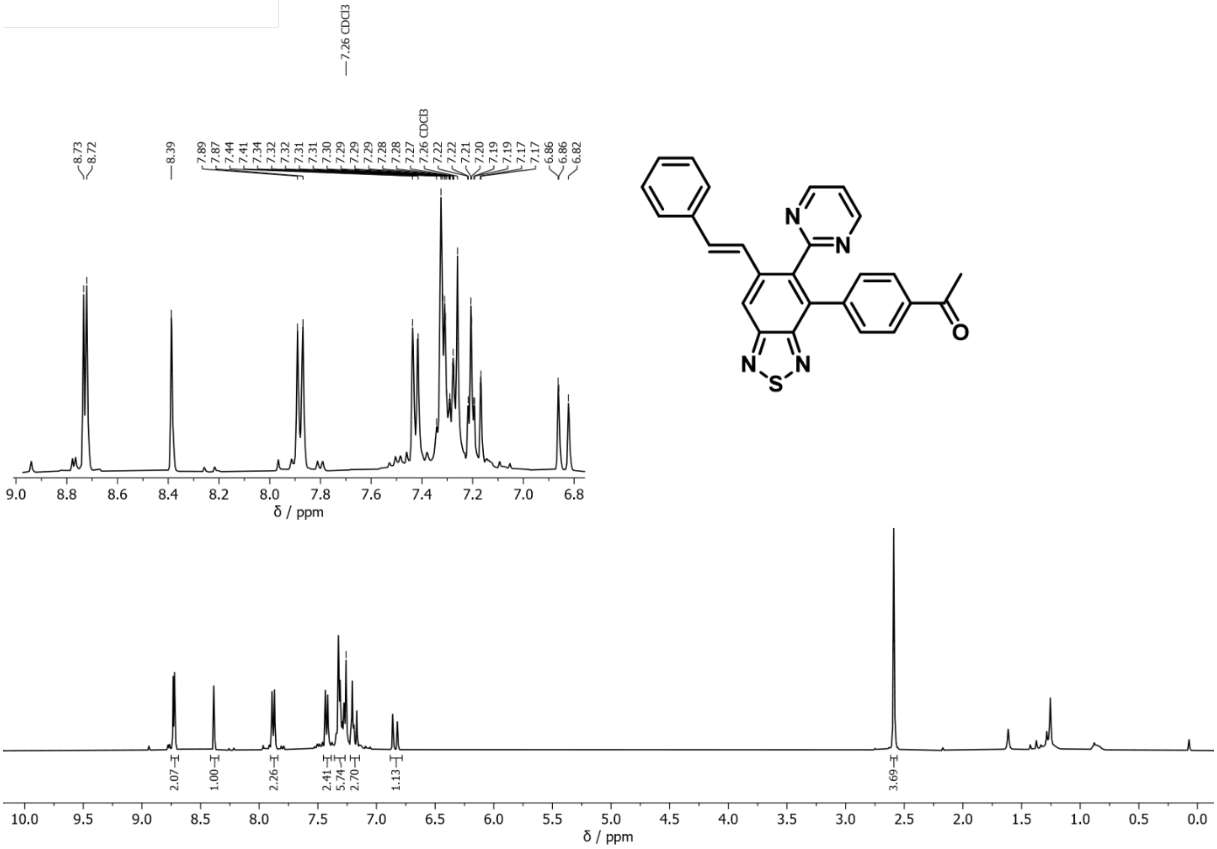

*Figure S74: ^{1}H NMR spectrum of **13c** (and presumably its isomer, ratio approximately 15:1) in $CDCl_3$ (400 MHz).*

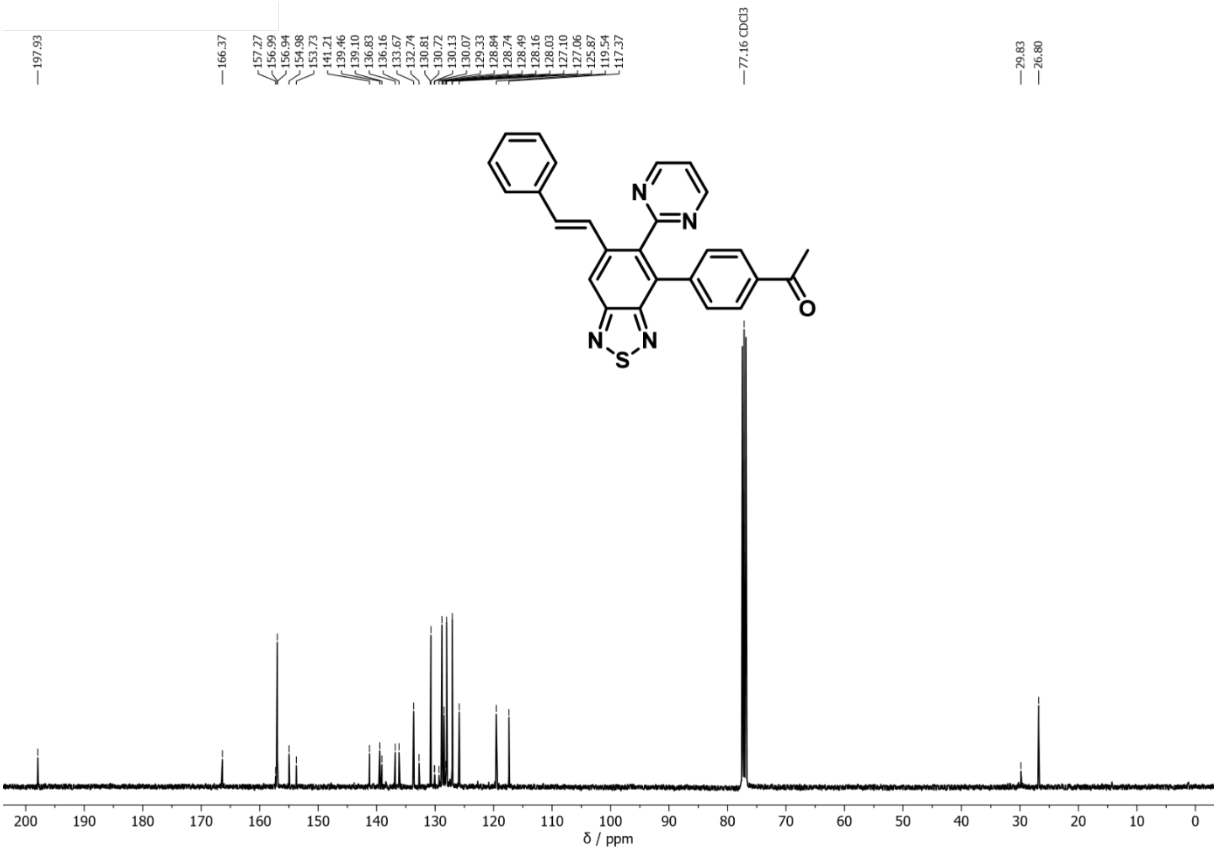

*Figure S75: $^{13}C\{^{1}H\}$ NMR spectrum of **13c** (and presumably its isomer, ratio approximately 15:1) in $CDCl_3$ (101 MHz).*

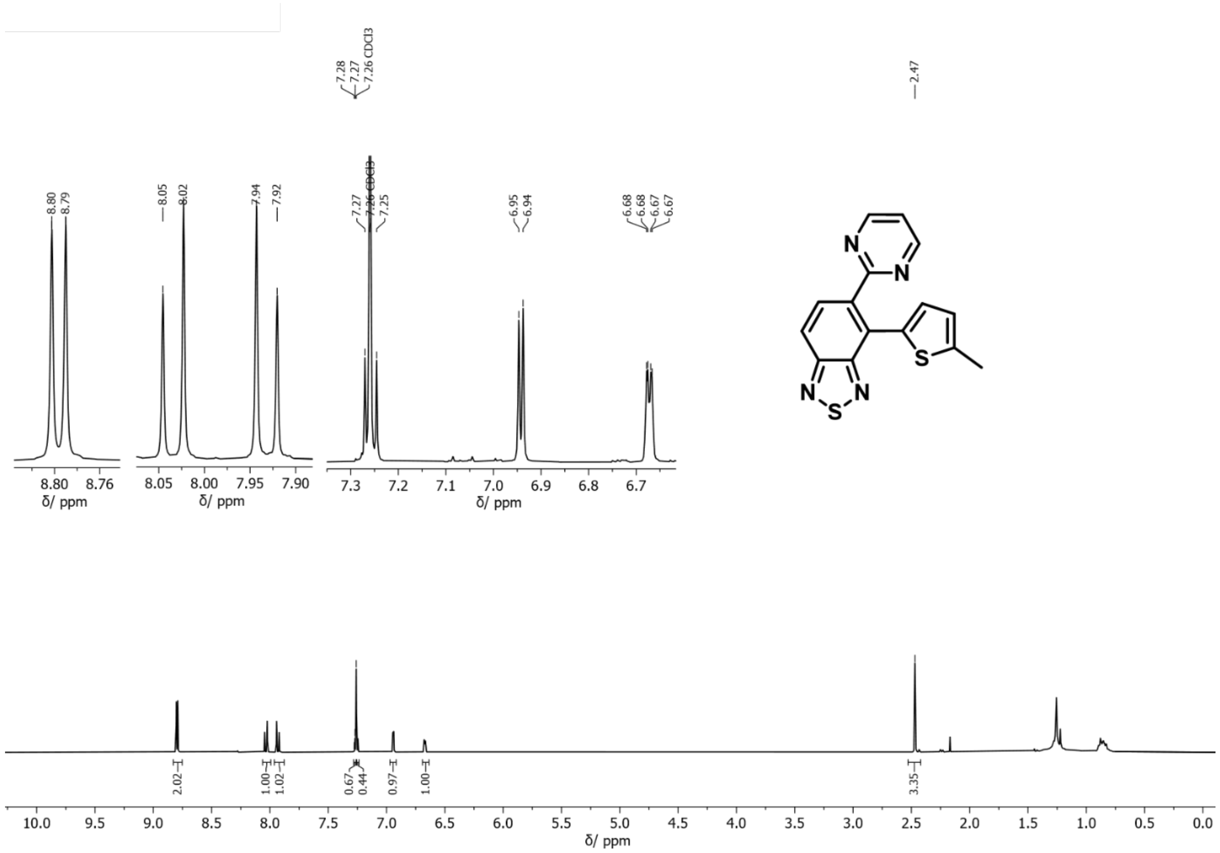

*Figure S14: ^{1}H NMR spectrum of **14** in $CDCl_3$ (400 MHz).*

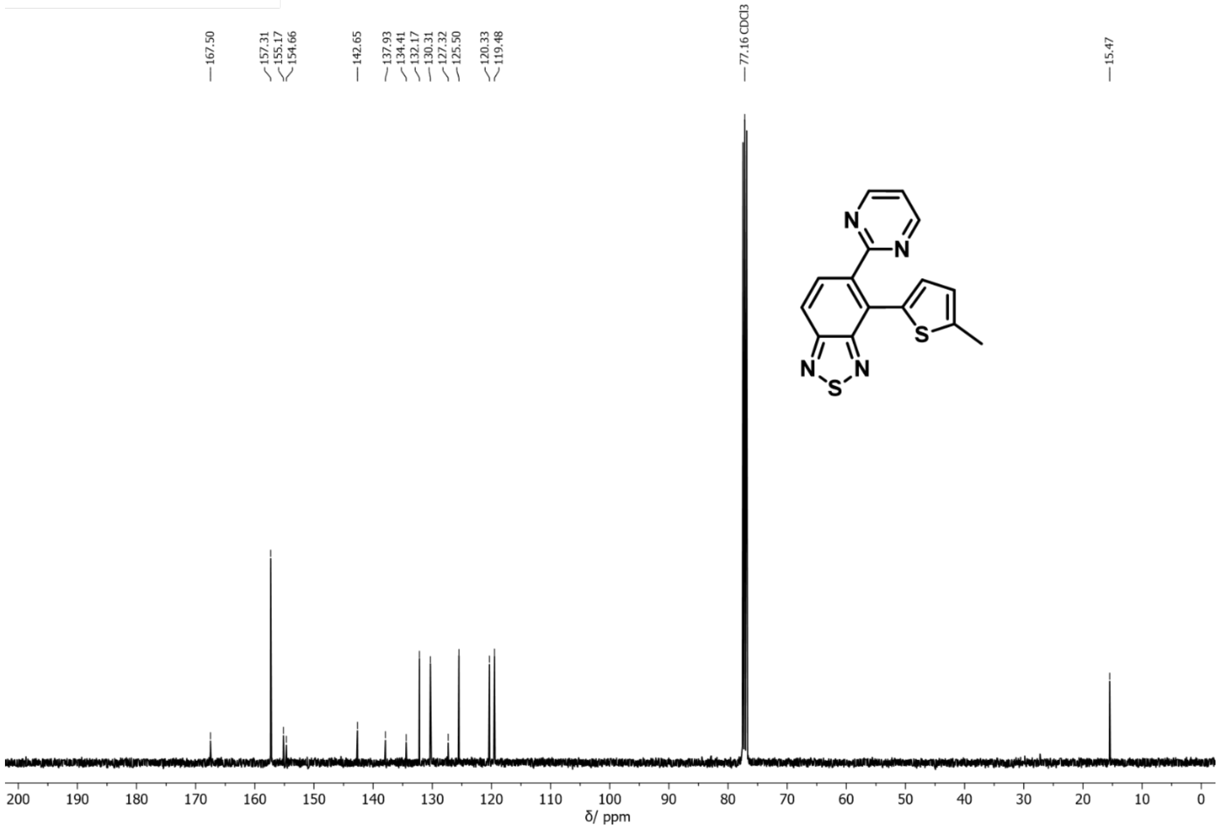

*Figure S15: $^{13}C\{^{1}H\}$ NMR spectrum of **14** in $CDCl_3$ (101 MHz).*

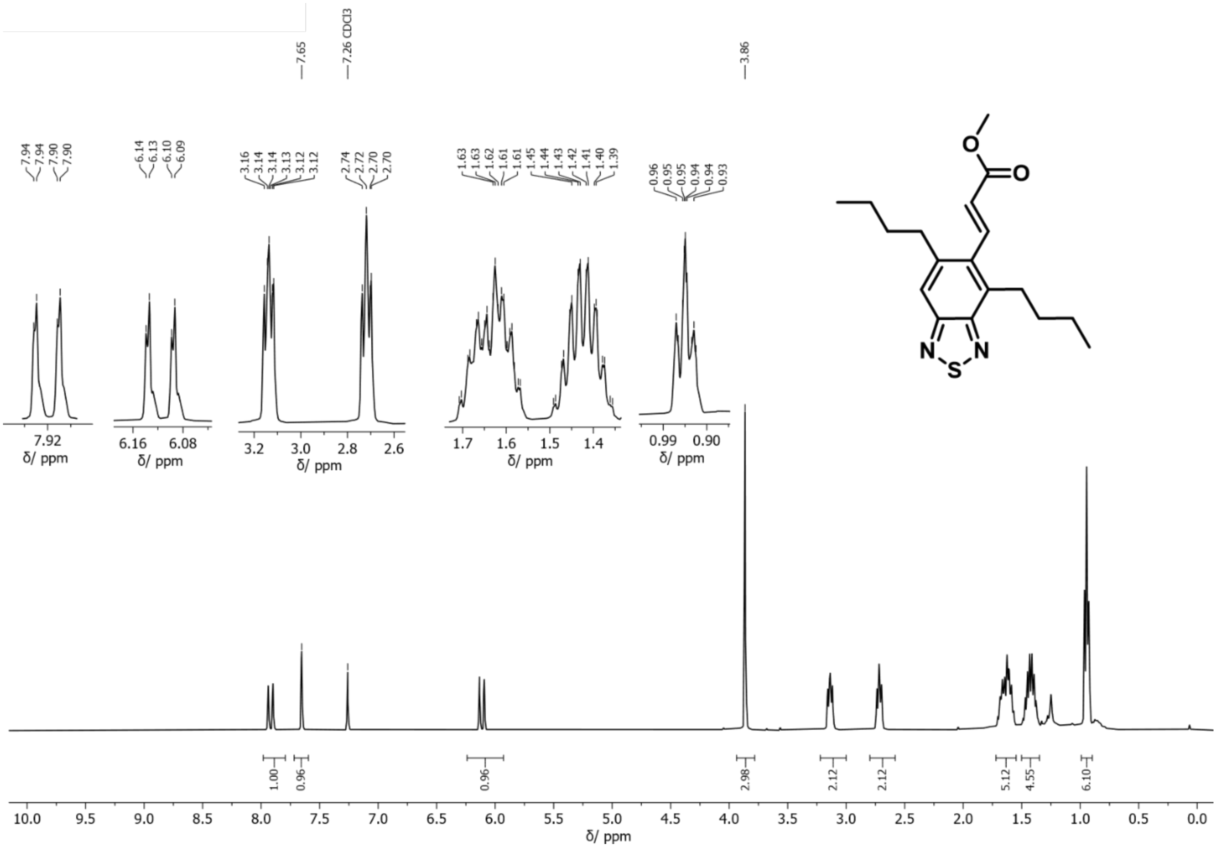

*Figure S78: ^{1}H NMR spectrum of **15** in $CDCl_3$ (400 MHz).*

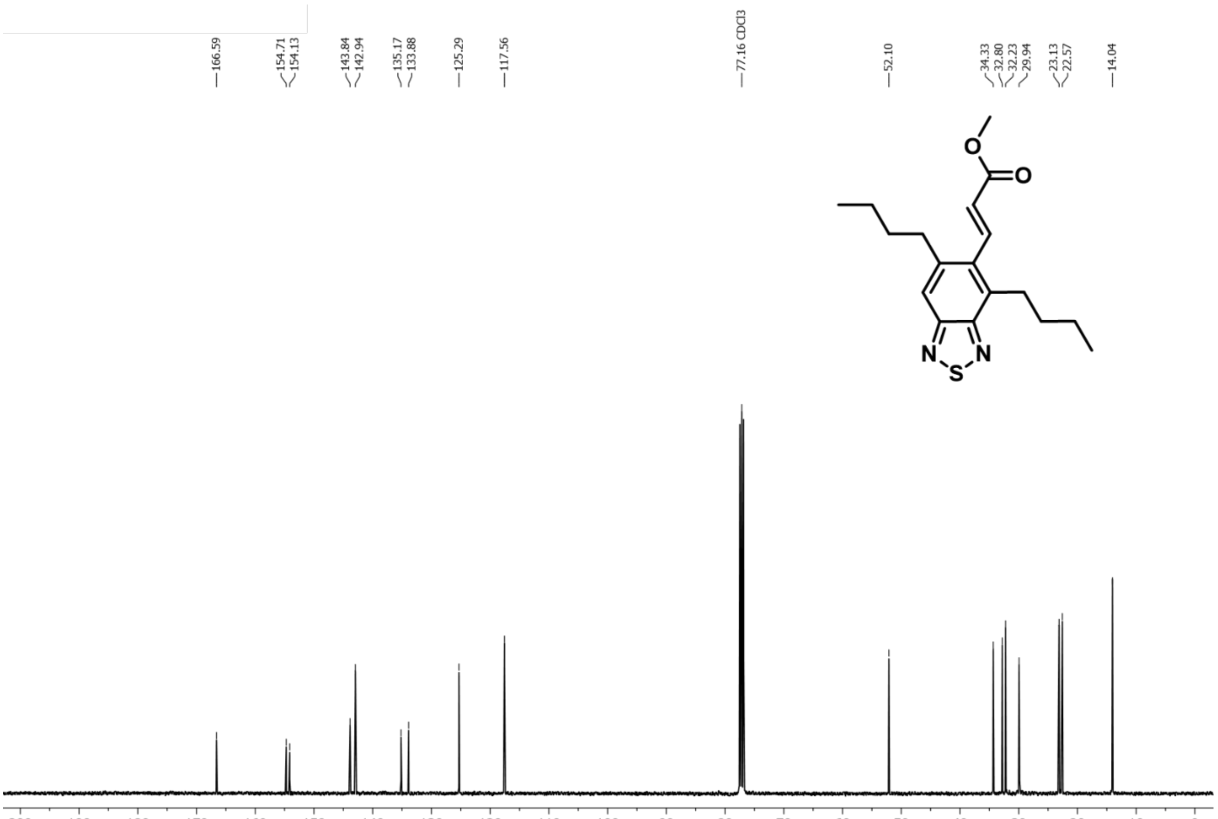

*Figure S79: $^{13}C\{^{1}H\}$ NMR spectrum of **15** in $CDCl_3$ (101 MHz).*

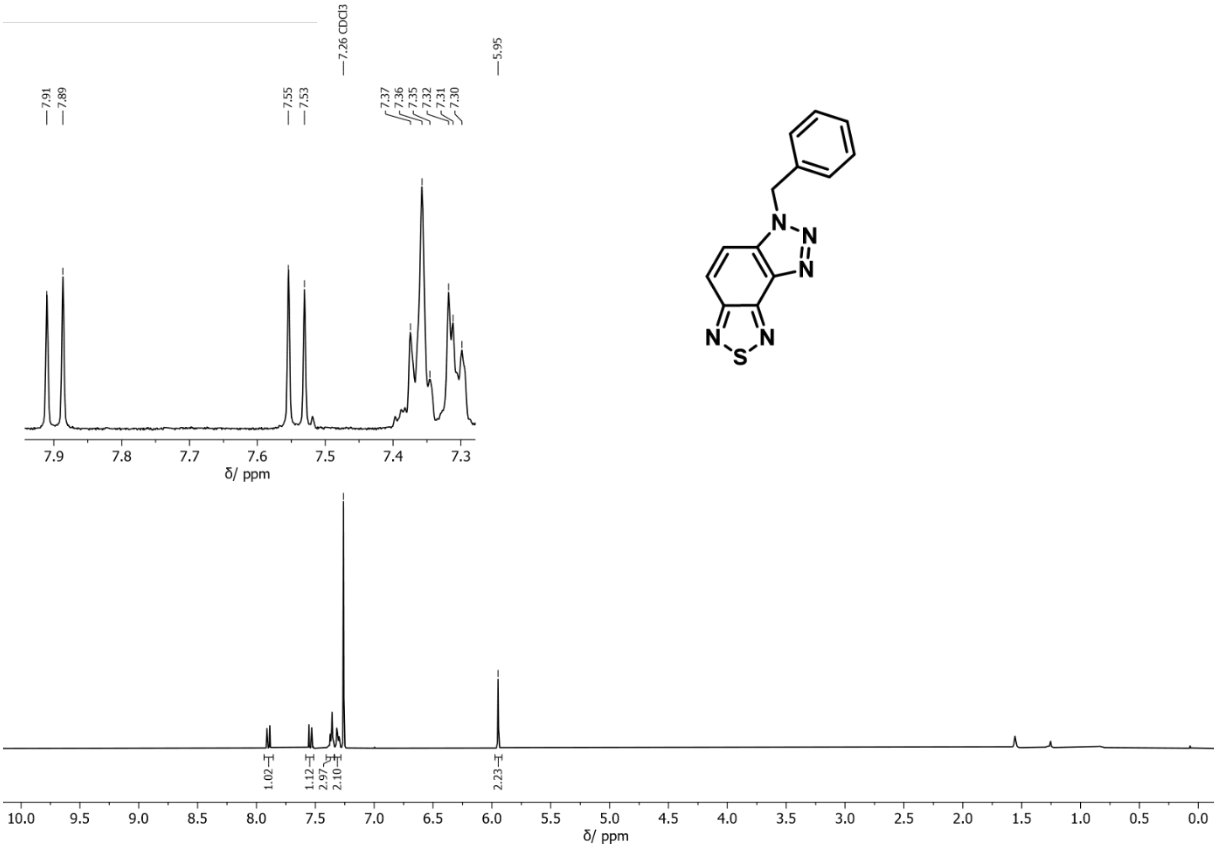

*Figure S80: ^{1}H NMR spectrum of **18a** in $CDCl_3$ (400 MHz).*

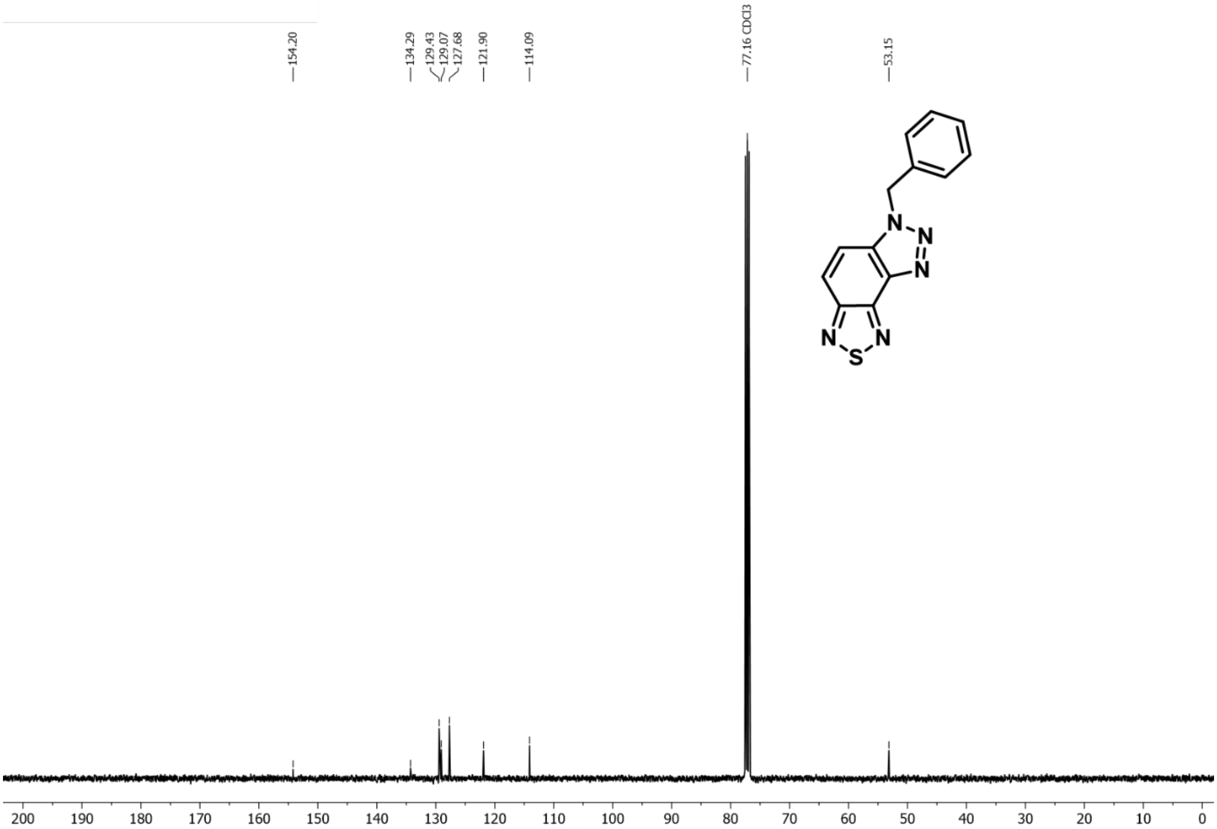

*Figure S81: $^{13}C\{^{1}H\}$ NMR spectrum of **18a** in $CDCl_3$ (101 MHz).*

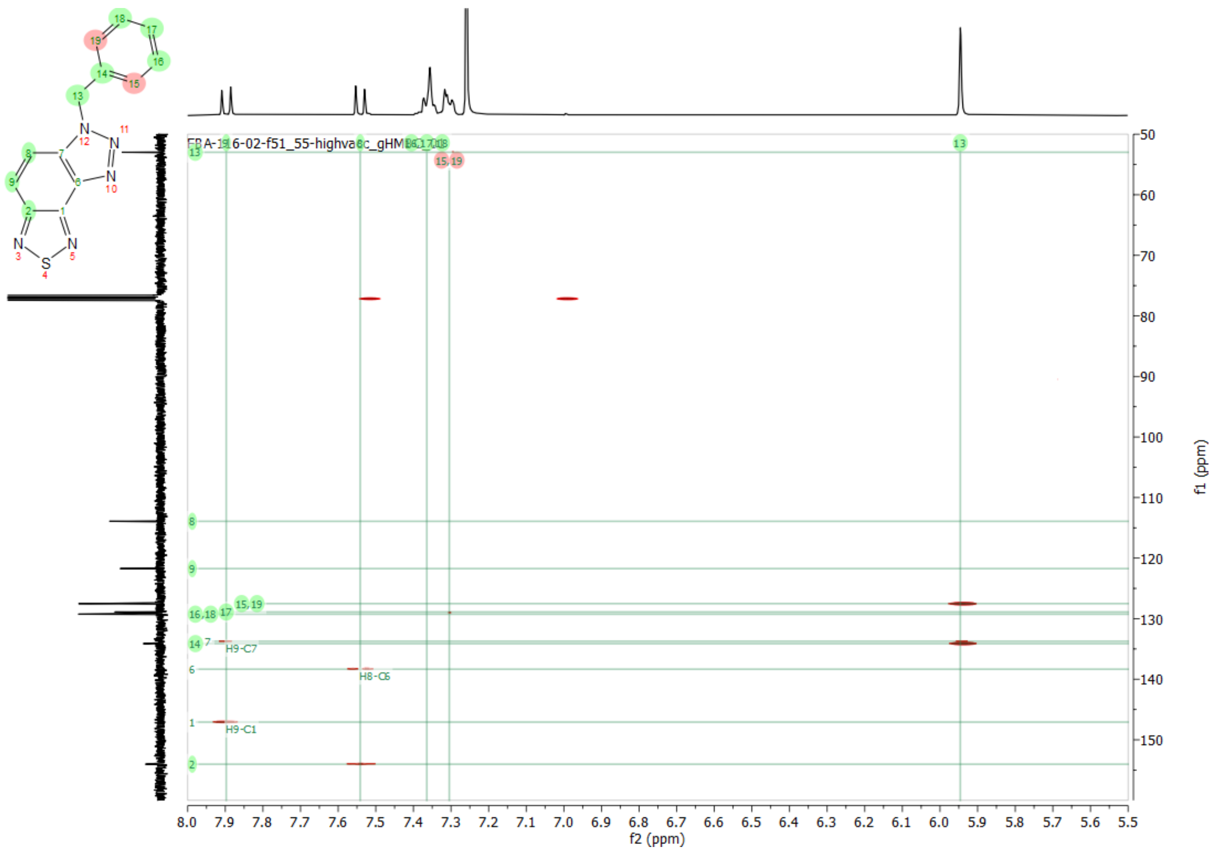

*Figure S82: HMBC NMR spectrum of **18a** in CDCl$_3$.*

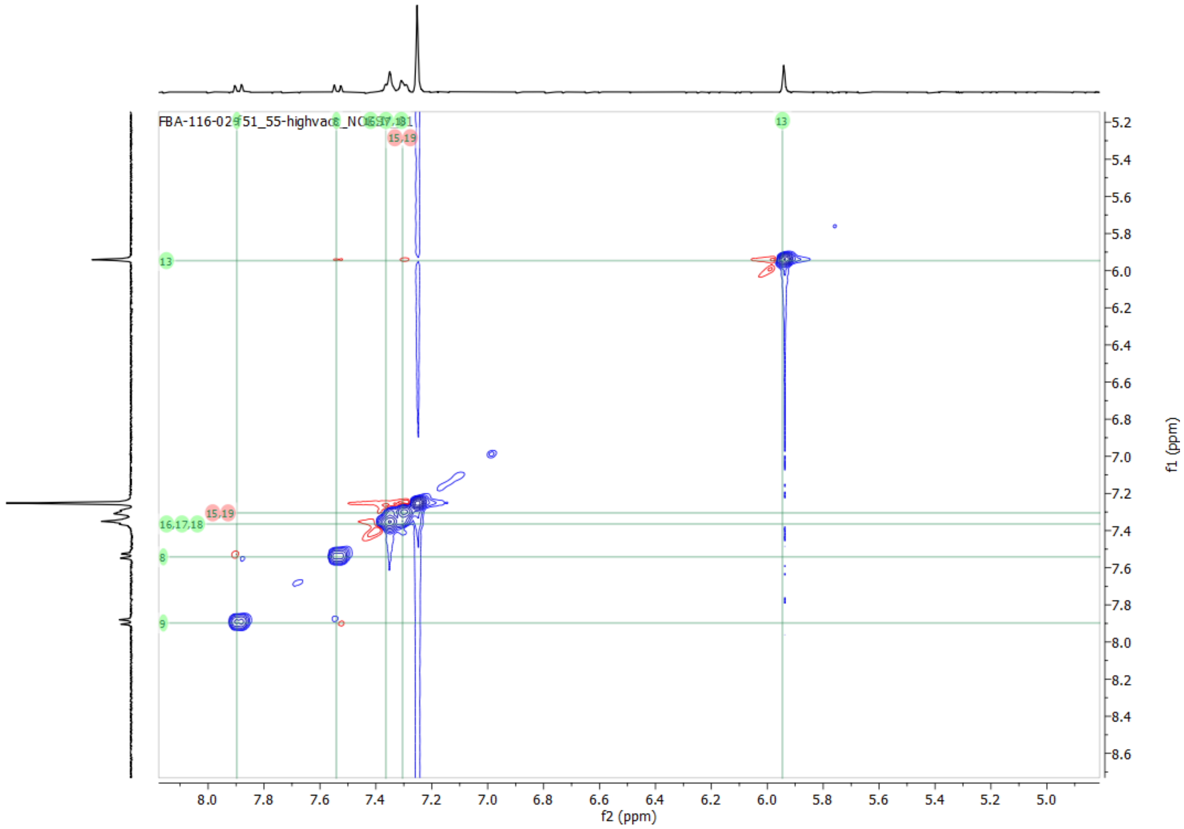

*Figure S83: NOESY NMR spectrum of **18a** in CDCl$_3$.*

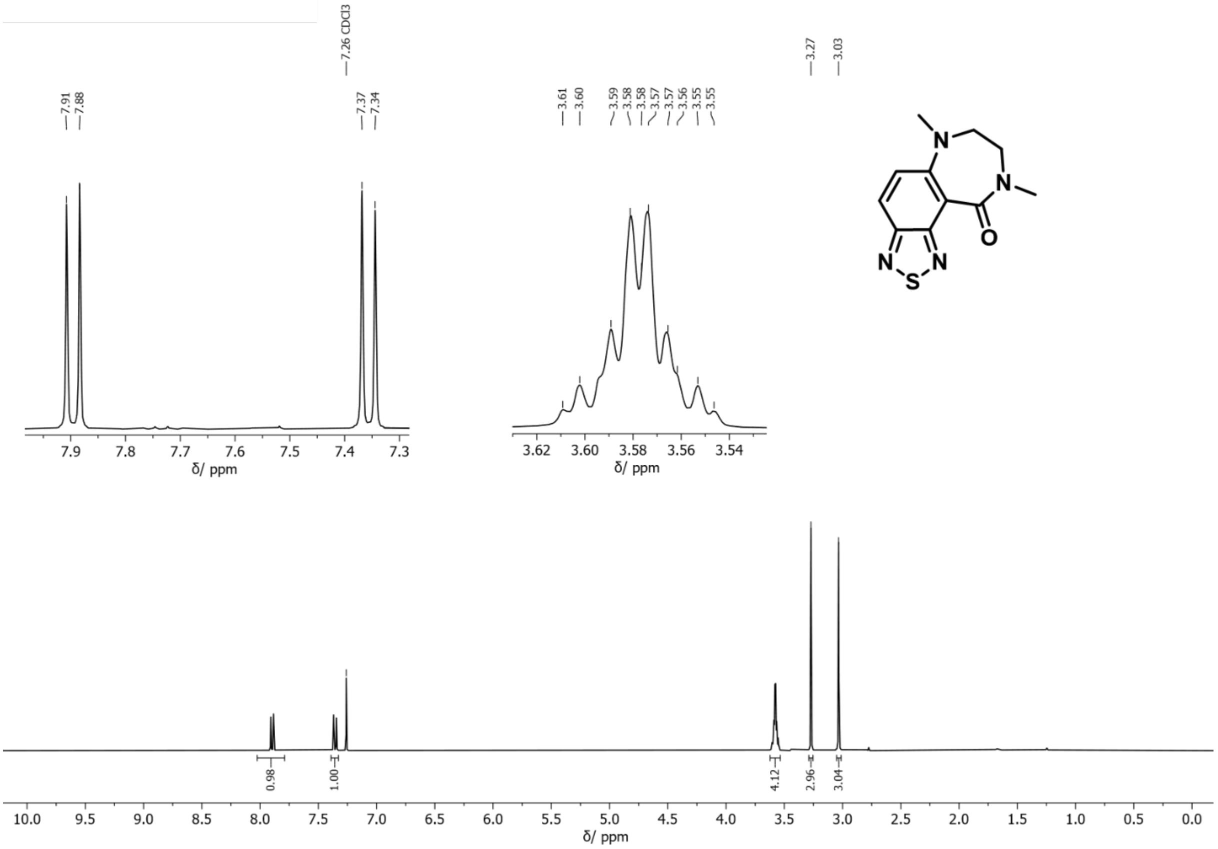

*Figure S84: ^{1}H NMR spectrum of **18b** in $CDCl_3$ (400 MHz).*

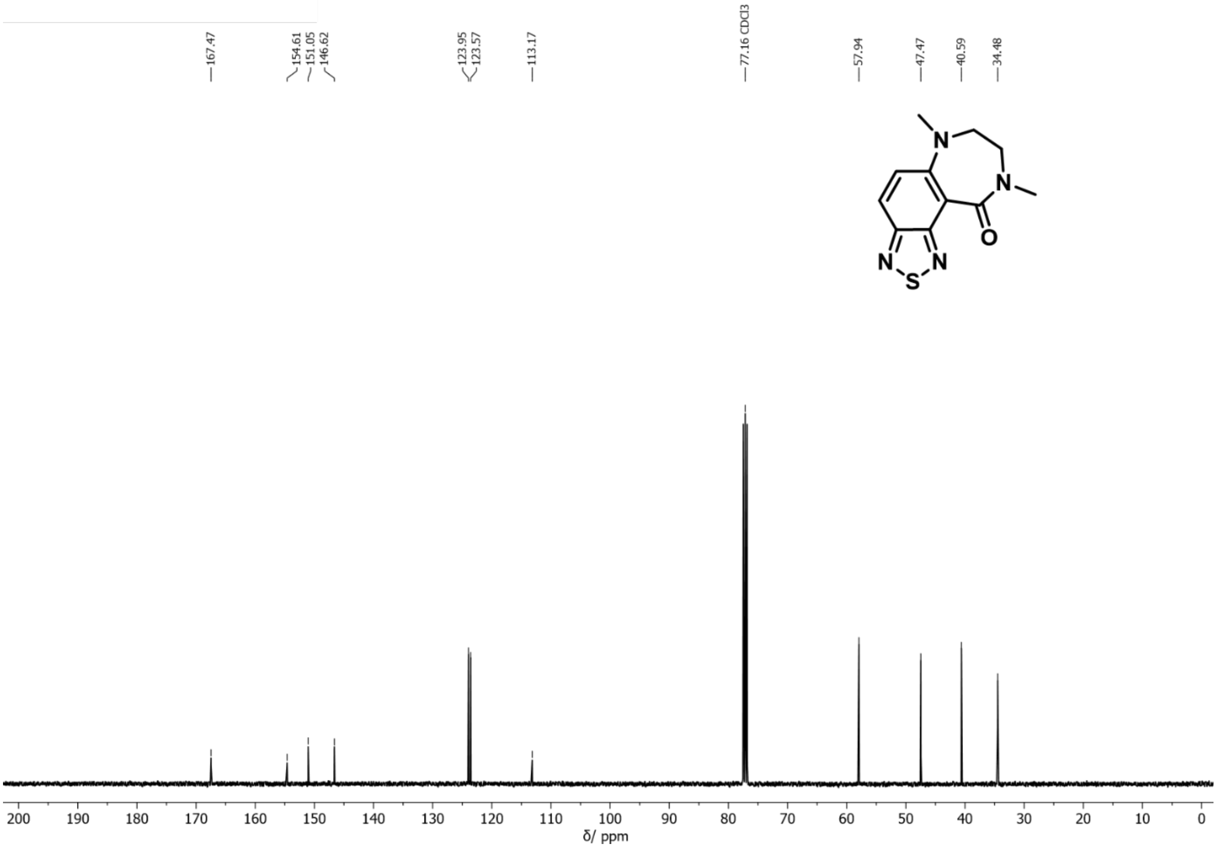

*Figure S85: ^{13}C{^{1}H} NMR spectrum of **18b** in $CDCl_3$ (101 MHz).*

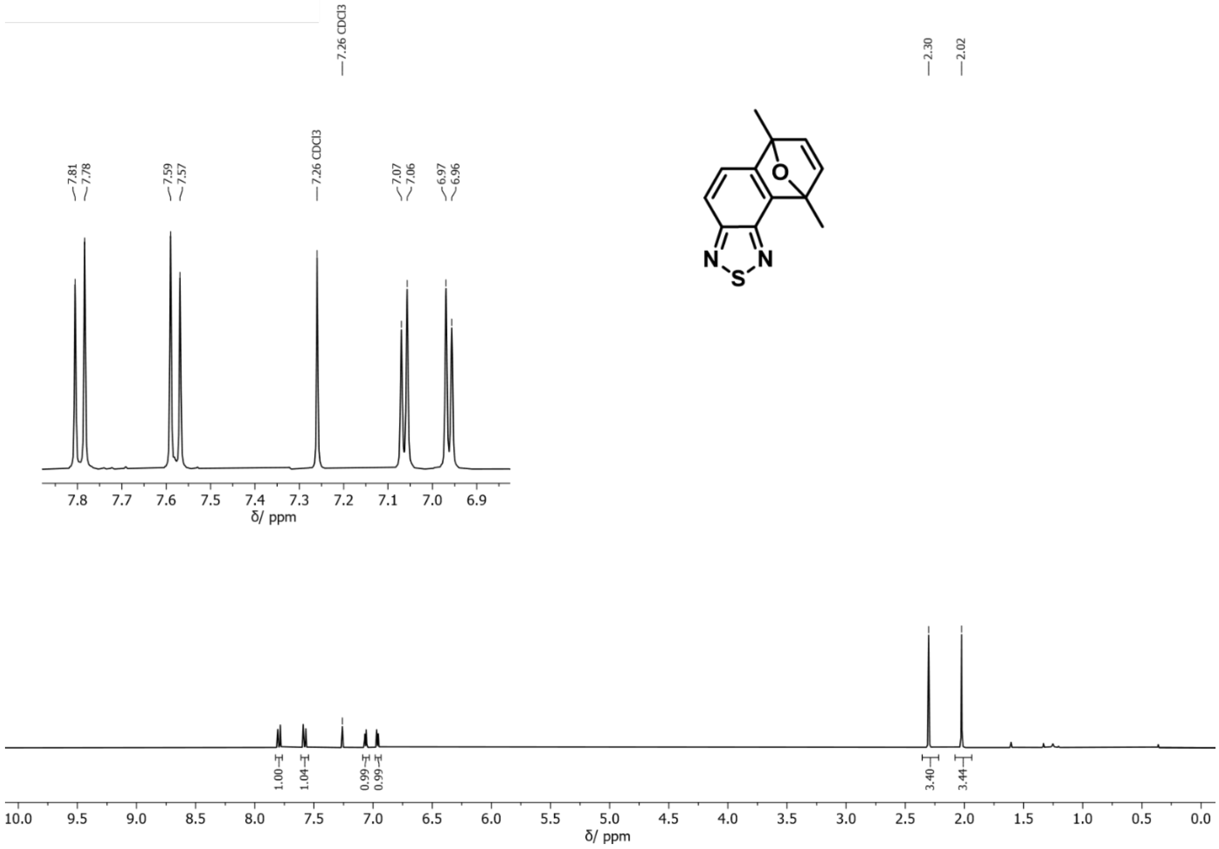

*Figure S86: ^{1}H NMR spectrum of **18c** in $CDCl_3$ (400 MHz).*

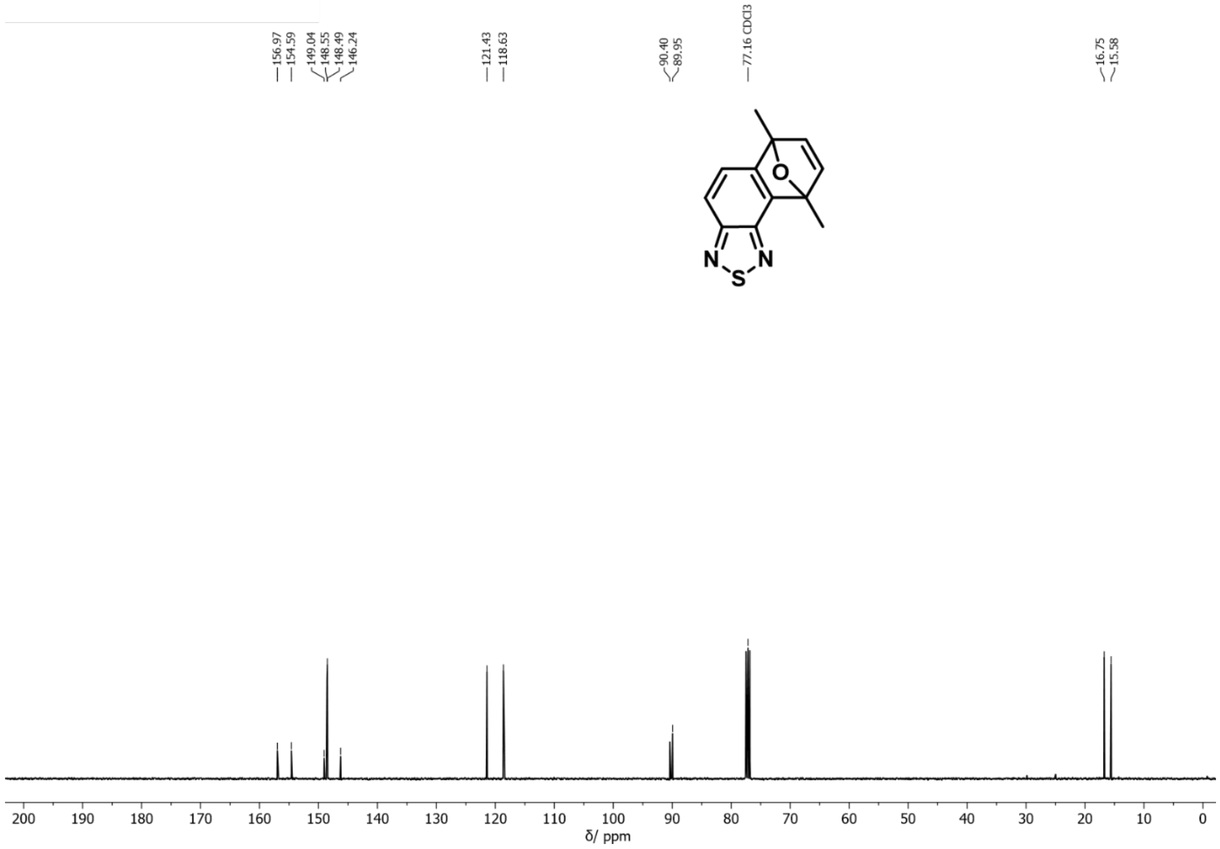

*Figure S87: $^{13}C\{^{1}H\}$ NMR spectrum of **18c** in $CDCl_3$ (101 MHz).*
